# Defluoroalkylation of Trifluoromethylarenes with Hydrazones: Rapid Access to Benzylic Difluoroarylethylamines

**DOI:** 10.1021/acs.orglett.3c00126

**Published:** 2023-02-27

**Authors:** Cecilia M. Hendy, Cameron J. Pratt, Nathan T. Jui, Simon B. Blakey

**Affiliations:** † Department of Chemistry, Emory University, Atlanta, Georgia 30322, United States; ‡ Loxo Oncology, Boulder, Colorado 80301, United States

## Abstract

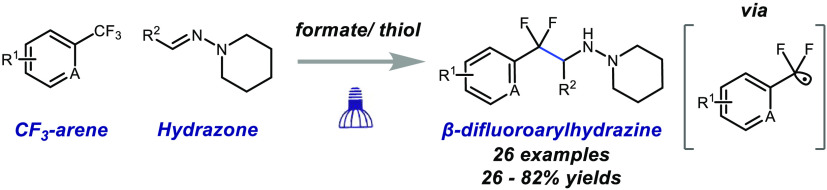

Here, we report an efficient and modular approach toward the formation of difluorinated arylethylamines from simple aldehyde-derived *N,N*-dialkylhydrazones and trifluoromethylarenes (CF_3_-arenes). This method relies on selective C–F bond cleavage via reduction of the CF_3_-arene. We show that a diverse set of CF_3_-arenes and CF_3_-heteroarenes react smoothly with a range of aryl and alkyl hydrazones. The β-difluorobenzylic hydrazine product can be selectively cleaved to form the corresponding benzylic difluoroarylethylamines.

Arylethylamines are an important class of biologically active molecules found across endogenous neurotransmitters (e.g., dopamine), natural products, pharmaceuticals, and agrochemicals (Figure 1A).^1^ The introduction of fluorine atoms into small molecules is well-known to have large impacts on biological properties such as potency, bioavailability, and metabolic stability.^2^ In particular, the α,α-difluorobenzylic motif^3^ has been recognized as an excellent bioisostere for aryl ethers and ketones,^4^ and its introduction into arylethylamines would provide significant opportunity to tune biological activity. Indeed, this has been showcased by a series of thrombin inhibitors developed by Merck that contain an arylethylamine core.^5^ They found that substitution of the benzylic hydrogen atoms with fluorine significantly enhanced the potency of the inhibitor (Figure 1A). However, the synthesis of the β-difluoroarylethylamine component of the inhibitor required a five-step sequence.^6^ Beyond this example, the evaluation of the benzylic difluorination of arylethylamines in other bioactive small molecules is underexplored, likely due to the current lack of convenient synthetic methods to access these compounds. In this manuscript we describe a modular reaction for the formation of benzylic difluoroarylethylamines from readily accessible components. We anticipate this methodology will serve as a valuable tool for the synthesis and evaluation of new difluorinated arylethylamine analogues in the development of novel pharmaceuticals and agrochemicals.

**Figure 1:**
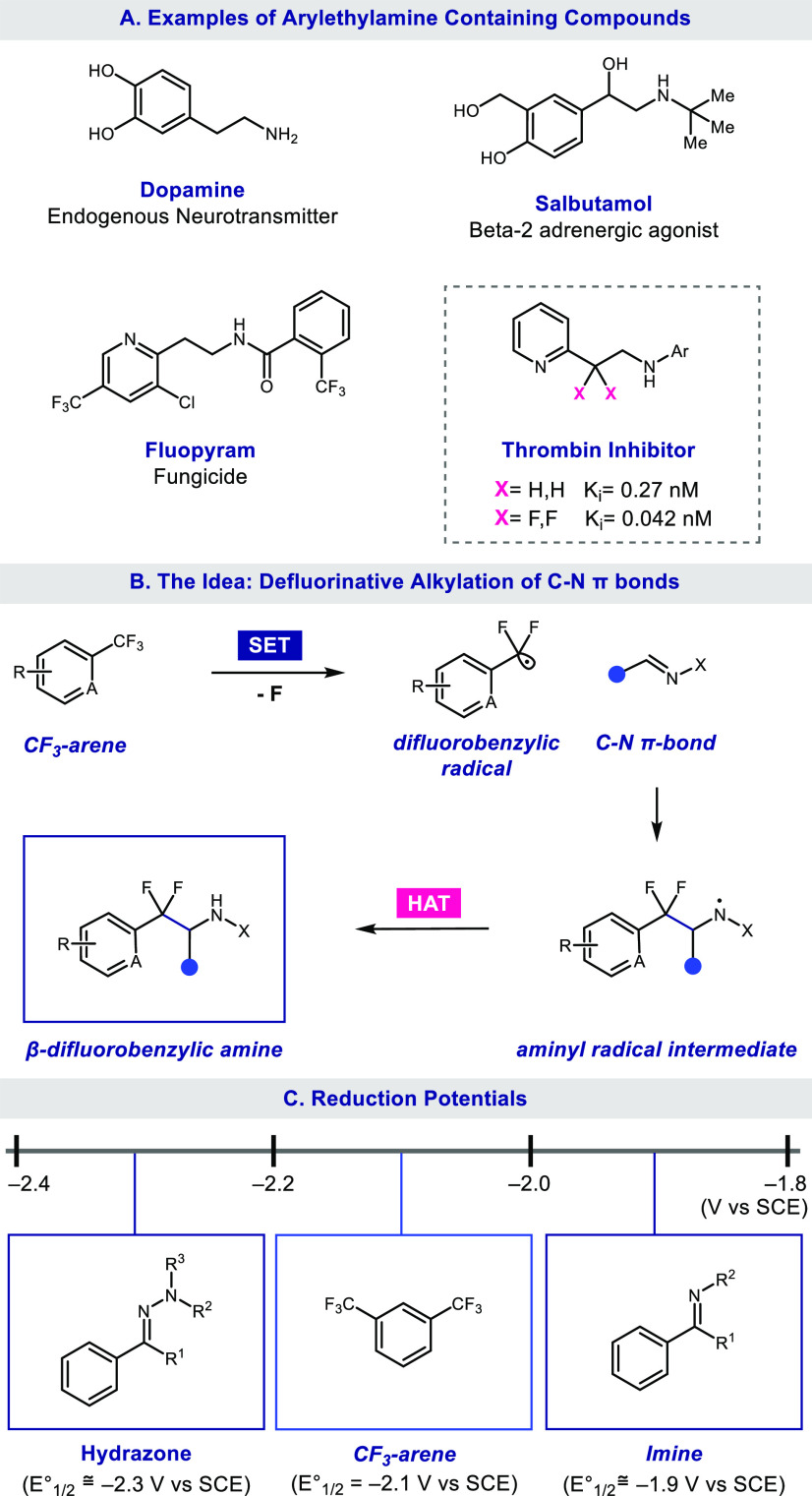
Approach toward the formation of benzylic difluoro arylethylamines.

Our lab^7^ and others^8^ have developed chemistry to selectively activate trifluoromethylarenes (CF_3_-arenes) through a reductive C–F cleavage that is induced through single-electron transfer to afford the difluorobenzylic radical. We envisioned engaging a difluorobenzylic radical with a C–N π-bond and capturing the resulting aminyl radical by hydrogen atom transfer (HAT) to deliver the desired difluorobenzylic arylethylamine scaffold (Figure 1B).

However, the key to unlocking this methodology would lie in the selection of the appropriate C–N π-bond type (Figure 1C). Although imines would serve as a straightforward building block for direct access to the desired product, two significant problems preclude their use. The reaction of an electrophilic difluorobenzylic radical with an electron-poor imine would result in a high-energy transition state due to unfavorable polar interactions.^9^ Additionally, the CF_3_-arene (*E*_1/2_^°^ = −2.07 V vs SCE for bis(1,3-trifluoromethyl)benzene) presents a more challenging reduction than typical imines (*E*_1/2_^°^ = −1.91 V vs SCE for *N*-benzylideneaniline),^10^ which would lead to competitive reduction of the two species. Conversely, *N*,*N*-dialkylhydrazones have been demonstrated to display umpolung reactivity compared to their imine counterparts. This can be attributed to the nucleophilic aza-enamine character of *N*,*N*-dialkylhydrazones due to the conjugation of the terminal nitrogen lone pair with the with the C–N π-bond.^11^ In fact, it has been demonstrated that trifluoromethyl radicals^12^ and difluoro radicals derived from difluorobromoacetate^13^ and perfluoroalkyl bromides^14^ can efficiently undergo addition to aldehyde-derived *N*,*N*-dialkylhydrazones. However, in all these cases the resulting aminyl radical undergoes oxidation and deprotonation to deliver the corresponding fluorinated hydrazone. Furthermore, hydrazones (*E*_1/2_^°^ = −2.30 V vs SCE for benzophenone phenylhydrazone)^15^ are more challenging to reduce than CF_3_-arenes, minimizing complications from competitive reduction.

Building from our previously disclosed method for the reductive defluoroalkylation of unactivated olefins using CO_2_^•–^ as the reductant,^16^ we began our investigation with the reaction of CF_3_-arene **1** and hydrazone **2**. Upon treating 1.0 equiv of **1** and 3.0 equiv of **2** in the presence of 4CzIPN (1 mol %), sodium formate (4 equiv), and mesna (20 mol %) in DMSO, we observed the desired product **3** in a 39% yield (Table 1, entry 1). The reversal of the stoichiometry to 3.0 equiv of **1** and 1.0 equiv of **2** led to an increased yield of **3** (54%, Table 1, entry 2). Upon heating the reaction to 80 °C, we were pleased to observe a 99% yield of **3** (Table 1, entry 3). Control experiments demonstrated that photocatalyst, sodium formate, light, and mesna were crucial for the observed reactivity (Table 1, entries 4–7).

**Table 1:**
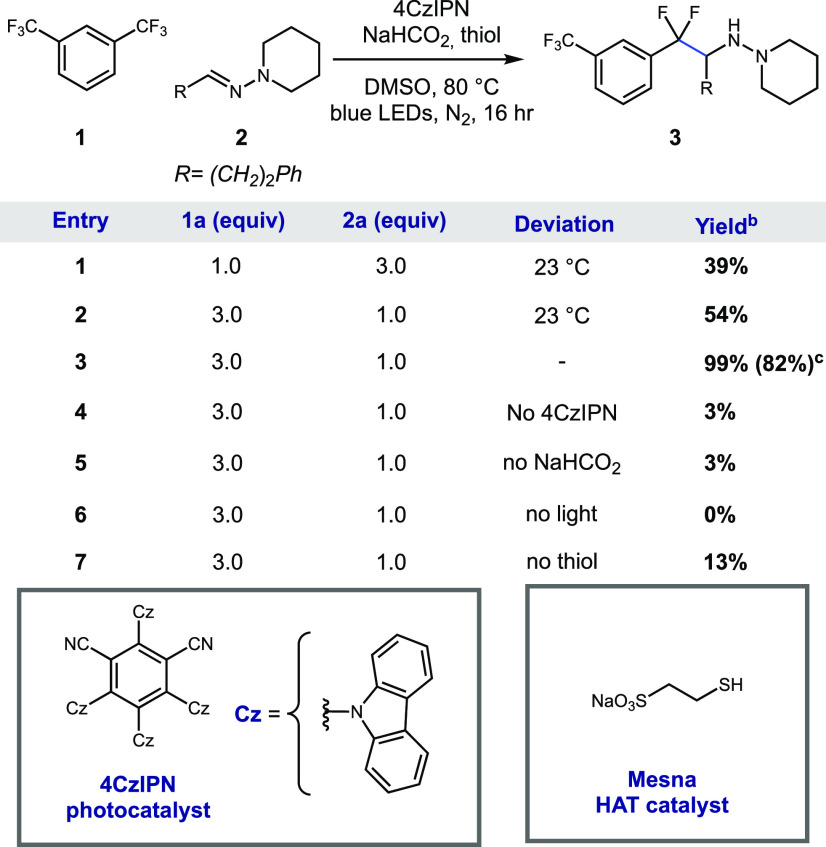
Optimization of the Defluoroalkylation of CF_3_-Arenes with Hydrazones^a^

a Conditions are as follows: **1** (0.3 mmol), **2** (0.1 mmol), 4CzIPN (1 mol %), NaHCO_2_ (0.4 mmol), mesna (20 mol %), DMSO (1 mL), blue LEDs, and N_2_ at 80 °C for 16 h.

b Yields determined by ^19^F NMR with 2-(trifluoromethyl)pyridine as the internal standard.

c Isolated yield shown.

We next turned our attention to understanding the scope of this transformation (Scheme 1). During this study, we found that certain substrates gave higher yields in the presence of 3.0 equiv of formic acid (noted in Scheme 1 accordingly). We attribute the enhanced yields with formic acid to the protonation of the hydrazine product, which prevents undesired oxidation of the nitrogen lone pair by the photocatalyst that can lead to degradation products.^17^

**Scheme 1:**
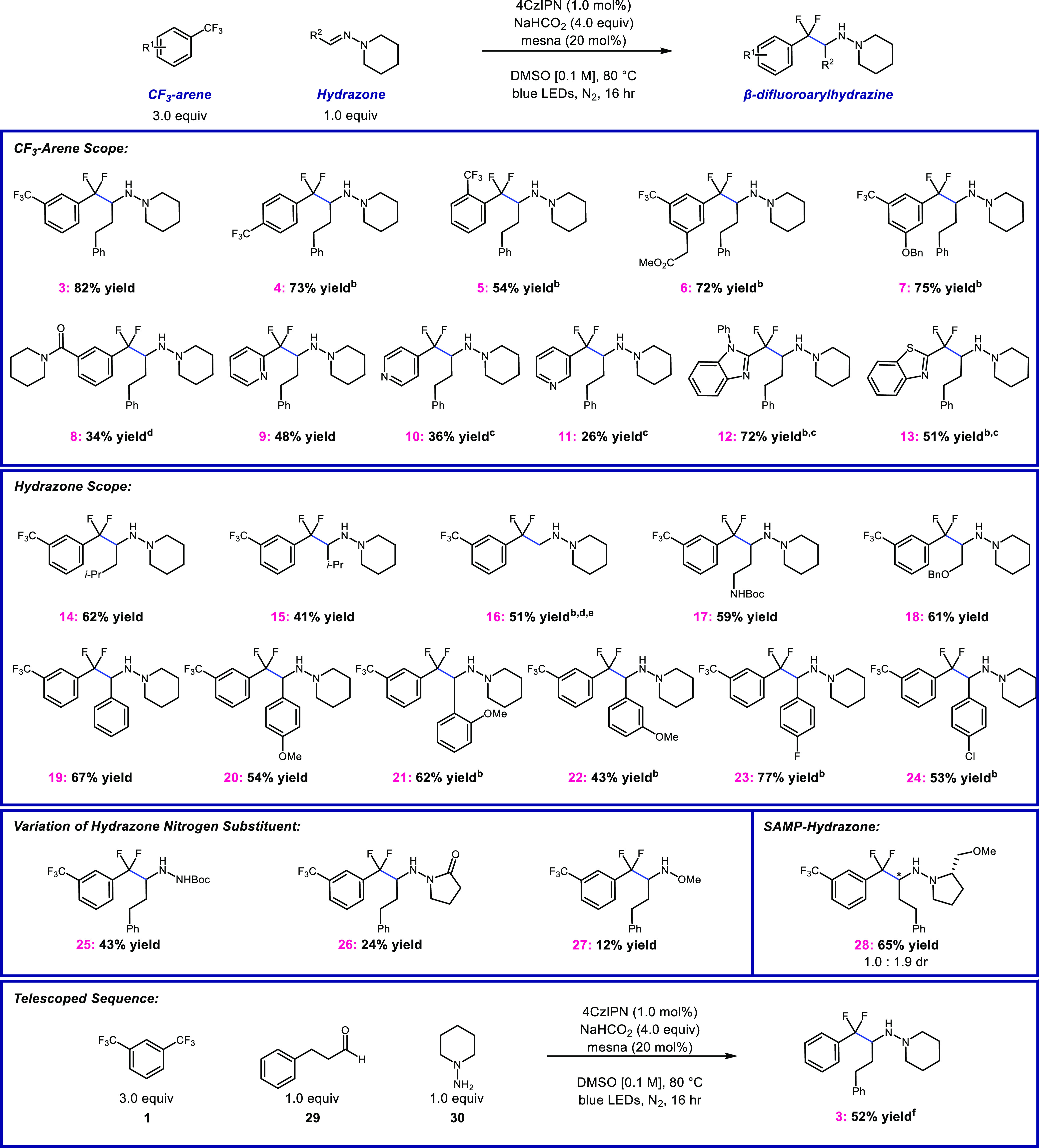
Scope of the Defluoroalkylation of CF_3_-Arenes with Hydrazones Conditions are as folows: CF_3_-arene (3 equiv), hydrazone (1 equiv), 4CzIPN (1 mol %), NaHCO_2_ (4 equiv), mesna (20 mol %), DMSO [0.1 M], blue LEDs, and N_2_ at 80 °C for 16 h. Isolated yields are shown. Reactions were performed on a 0.1- 0.5 mmol scale (see the Supporting Information). 3.0 equiv of HCOOH was added to the reaction mixture. Yields determined by ^1^H NMR with dibromomethane as the internal standard. Yields determind by ^19^F NMR using 2-(trifluoromethyl)pyridine as the internal standard. The reaction was performed with CF_3_-arene (1 equiv), hydrazone (3 equiv), and NaHCO_2_(8 equiv). Conditions are as follows: CF_3_-arene (3 equiv), aldehyde (1 equiv), hydrazine (1 equiv), 4CzIPN (1 mol %), NaHCO_2_ (4 equiv), mesna (20 mol %), DMSO [0.1 M], blue LEDs, and N_2_ at 80 °C for 16 h. The isolated yield is shown. The reaction was performed on a 0.5 mmol scale.

Our investigation began with the evaluation of a variety of CF_3_-arenes using hydrazone **2**. Bis(trifluoromethyl)benzenes (**3**–**5**) were excellent substrates irrespective to the substitution pattern (54–82% yield). This reaction allows the selective activation of only one of the C–F bonds because the defluoroalkylation products (ArCF_2_R) are harder to reduce than the starting materials (ArCF_3_). Additionally, 5-substituted 1,3-bis(trifluoromethyl)benzene substrates (**6** and **7**) gave good yields (72–75% yield). Nonfluorinated withdrawing groups on the arene, such as amide **8** (34% yield), were also tolerated. Importantly, this reactivity extended to a variety of nitrogen-containing heterocycles. We examined a series of (trifluoromethyl)pyridines with variation of the position of the trifluoromethyl group around the pyridine. While 2-(trifluoromethyl)pyridine **9** gave a moderate yield (48%), both 4- and 3-trifluoromethyl-substituted pyridine (**10** and **11**, respectively) gave lower yields (36% and 26%, respectively). In addition to pyridines, benzimidazole **12** (72% yield) and benzothiazole **13** (51% yield) reacted smoothly under these conditions. The current limitation of this chemistry regarding the CF_3_-arene scope is that CF_3_-arenes that do not bear an electron-withdrawing group are unreactive because their reduction potentials lie outside the range of the CO_2_^•–^ reductant (see the Supporting Information).

The hydrazone scope was evaluated using CF_3_-arene **1**. A variety of alkyl hydrazones reacted smoothly under these conditions. We first examined the steric tolerance of the reaction. The β-isopropyl hydrazone **14** reacted smoothly (62% yield); however, the α-isopropyl group **15** gave slightly diminished reactivity (41% yield). The corresponding α-*tert*-butyl hydrazone gave no product (see the Supporting Information). Nonsubstituted hydrazone **16** gave a moderate yield (51%) under slightly modified conditions (1.0 equiv of CF_3_-arene and 3.0 equiv of hydrazone). Hydrazones containing a Boc-protected amine (**17**, 59% yield) and a benzyl-protected alcohol (**18**, 61% yield) were tolerated. Aryl-derived hydrazones were also suitable reaction partners. Benzaldehyde-derived hydrazone **19** reacted in a good yield (67%). We next examined substitution around the benzene ring with a methoxy group (**20**–**22**) and found it was tolerated in all positions (43–62%). While electron-rich benzaldehyde hydrazones reacted well, electron-deficient hydrazones (e.g., cyano) gave no desired product and led to a complex mixture of unidentified products. However, we were pleased to find that hydrazones bearing an aryl fluoride (**23**) and chloride (**24**) reacted well (77% and 53% yields, respectively) with no detectable hydrodehalogenation side products.

Furthermore, we evaluated a small series of hydrazones with various substitutions on the hydrazone nitrogen. The NHBoc hydrazone **25** gave a moderate yield (43%), whereas the *N*-acyl hydrazone **26** gave a low yield (24%). This reactivity trend is consistent with more favorable polar effects between the electrophilic difluorobenzylic radical and the relatively electron-rich *N*,*N*-dialkylhydrazone compared to the electron-poor *N*-acyl hydrazone. Intriguingly, other types of C–N π-systems such as oximes reacted under these conditions, albeit in low yields (12% of **27**). We also evaluated a SAMP-hydrazone to probe the possibility of stereoselectivity in the radical addition. While this reaction proceeded in a good yield (65% yield of **28**), it gave relatively low diastereoselectivity (1.0:1.9). For operational simplicity, we also show that this reaction can be performed in a telescoped sequence where the hydrazone is formed *in situ*, with aldehyde **29** and hydrazine **30** giving the expected product **3** in a 52% yield.

We propose this transformation operates through a CO_2_^•–^ chain mechanism (Figure 2A). The reaction begins with a photocatalytic oxidation of mesna (*E*_1/2_^°^ = +1.12 V vs SCE)^16^ by the excited state of the photocatalyst, 4CzIPN* (*E*_1/2_^°^ = +1.43 V vs SCE).^18^ The resulting thiyl radical undergoes HAT with sodium formate to deliver CO_2_^•–^ (*E*_1/2_^°^ = −2.21 V vs SCE).^19^ This undergoes single-electron transfer with the CF_3_-arene **I**. The resulting CF_3_-arene radical anion expels the benzylic fluoride through mesolytic cleavage, generating the difluorobenzylic radical **II**. The reaction of **II** with the hydrazone **III** results in aminyl radical **IV**. Due to the electrophilic nature of nitrogen-centered radicals, we propose that HAT occurs with nucleophilic formate, leading to the propagation of the chain mechanism and the delivery of the desired product **V**. However, despite thiol being an electrophilic H atom donor, we cannot rule out HAT from mesna, as typical deuteration experiments cannot be performed due to the exchangeable nature of the hydrogen atoms under this system. To confirm the presence of a radical chain, we subjected **1** and **2** to conditions using methyl disulfide (20 mol %) as an alternative initiator in place of photocatalyst and thiol (Figure 2B). We observed a 59% yield of **3**, which is consistent with our previous findings of radical chain reduction via CO_2_^•–^.^16^

**Figure 2:**
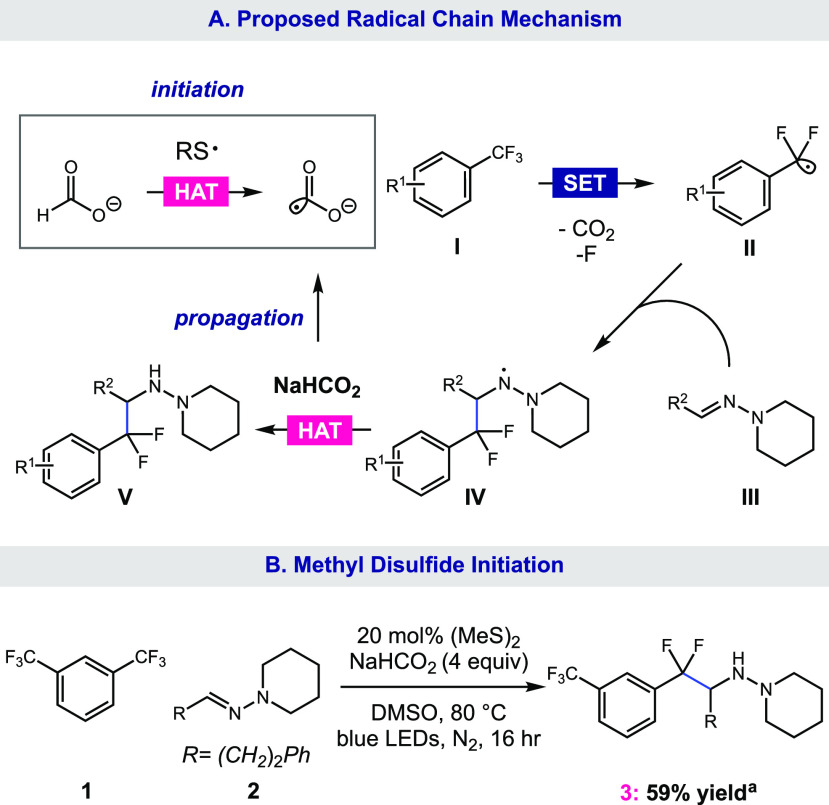
(A) Proposed radical chain mechanism and (B) alternative initiation using methyl disulfide. ^a^Yield determined by ^19^F NMR using 2-(trifluoromethyl)pyridine as the internal standard.

Finally, we demonstrated the efficient cleavage of the hydrazine bond to afford the valuable benzylic difluoroarylethylamine scaffold (Scheme 2). While the reductive cleavage of N–N σ-bonds has been well established,^20^ it was important show that the hydrazine could be chemoselectively cleaved in the presence of the relatively weak benzylic fluorine bonds. When using the hydrogenation catalyst palladium on carbon, we observed no N–N reduction, even when high pressures were applied. However, the use of Raney nickel under an atmosphere of hydrogen resulted in the formation of amine **31** in an excellent yield (79%), with only trace formation of benzylic defluorination observed by ^1^H NMR and LCMS.

**Scheme 2:**
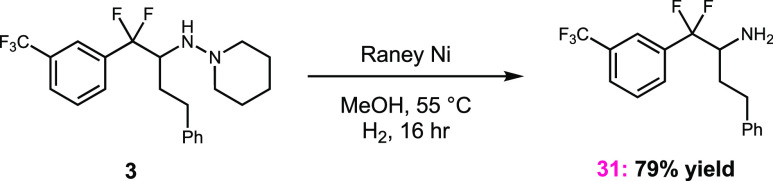
Hydrazine Bond Cleavage Using Raney Nickel Conditions are as follows: **3** (0.1 mmol), Raney nickel (0.310 g), MeOH (2.5 mL), and H_2_ (balloon) at 55 °C for 16 h. The isolated yield is shown.

In summary, we have developed a method for the formation of β-difluorobenzylic hydrazines using a diverse array of CF_3_-arenes and hydrazones utilizing inexpensive and benign reagents. Reactivity is enabled through CO_2_^•–^ reduction and operates through an efficient radical chain mechanism. The hydrazine products can be selectivity cleaved to form the corresponding pharmaceutically relevant benzylic difluoroarylethylamine scaffold. Further investigations toward stereoselective difluorobenzylic radical addition to hydrazones are underway in our lab.

## Data Availability

The data underlying this study are available in the published article and its Supporting Information.

## Supporting Information Available

Experimental procedures and compound characterization (PDF)
