# Supplementary material for: Defluoroalkylation of Trifluoromethylarenes with Hydrazones: Rapid Access to Benzylic Difluoroarylethylamines
Source: Org Lett. 2023 Feb 27;25(9):1397–402. doi: 10.1021/acs.orglett.3c00126 (PMC10012270; doi:10.1021/acs.orglett.3c00126)
Supplement: Supplementary file 1 — ol3c00126_si_001.pdf [file ol3c00126_si_001.pdf]

Supporting Information

Defluoroalkylation of Trifluoromethylarenes with Hydrazones- Rapid Access to Benzylic Difluoroarylethylamines

Cecilia M. Hendy,[1] Cameron J. Pratt,[1] Nathan T. Jui,[2] Simon B. Blakey*[1]

*Corresponding author: sblakey@emory.edu

1. Department of Chemistry, Emory University, Atlanta, Georgia, 30322, United States
2. Loxo Oncology, Boulder, Colorado 80301, United States

I. General Information..........2
- a. **General Reaction and Reagent Information**..........2
- b. **General Analytical Information**..........2
- c. **Abbreviations**..........2
- d. **General Photoredox Setup**..........3

II. Optimization Details..........4

III. Preparation of Starting Materials..........6
- a. **Preparation of CF_3-Arenes**..........6
- b. **Preparation of Hydrazones**..........7

IV. Preparation of Products from Substrate Table..........13
- a. **General Procedures**..........13
- b. **Isolated Products from Substrate Table**..........14
- c. **1.0 mmol Scale Isolation**..........29
- d. **Telescoped Reaction**..........29
- e. **Unsuccessful Substrates**..........30

V. Nitrogen-Nitrogen Bond Cleavage..........31

VI. Alternative Initiator..........32

VII. References..........33

VIII. NMR Spectra..........34

I. General Information

I-A. General Reaction and Reagent Information

All reactions were set up on the bench top and conducted under nitrogen atmosphere while subject to irradiation from blue LEDs (HydroFarm powerPAR LED Grow Light Blue 15 Watt/440 nm, available at www.1000bulbs.com). Flash chromatography was carried out using Siliaflash® P60 silica gel obtained from Silicycle. Thin-layer chromatography (TLC) was performed on 250 μm SiliCycle silica gel F-254 plates. Visualization of the developed chromatogram was performed by fluorescence quenching or staining using $KMnO_4$ or ninhydrin stains. Anhydrous sodium formate was purchased from Sigma Aldrich and stored in a desiccator. Raney Nickel (W.R. Grace and Co. Raney® 2400, slurry, in H_2O, active catalyst) was purchased from Sigma Aldrich. Drisolv® DMSO was purchased from VWR and dried over 4Å molecular sieves. Photoredox catalyst 4CzIPN was prepared according to literature procedures.[1] All other reagents were purchased from Sigma-Aldrich, Alfa Aesar, Acros Organics, Combi-Blocks, Oakwood Chemicals, Ambeed, and TCI America and used as received.

I-B. General Analytical Information.

Unless otherwise noted, all yields refer to chromatographically and spectroscopically (^{1}H NMR) homogenous materials. New compounds were characterized by NMR and HRMS. ^{1}H and ^{13}C NMR spectra were obtained from the Emory University NMR facility and recorded on a Bruker 400 (400 MHz), Bruker 600 (600 MHz), INOVA 600 (600 MHz), INOVA 500 (500 MHz), or VNMR 400 (400 MHz), and are internally referenced to residual protio solvent signals. Data for ^{1}H NMR are reported as follows: chemical shift (ppm), multiplicity (s = singlet, d = doublet, t = triplet, q = quartet, p = pentet, m = multiplet, dd = doublet of doublets, dt = doublet of triplets, ddd= doublet of doublet of doublets, dtd= doublet of triplet of doublets, br s = broad singlet) coupling constant (Hz), integration, and assignment, when applicable. Data for decoupled ^{13}C NMR are reported in terms of chemical shift and multiplicity when applicable. High Resolution mass spectra were obtained from the Emory University Mass Spectral facility using a Thermo Scientific Extractive Plus with an orbitrap mass analyzer.

I-C. Abbreviations.

DCM = dichloromethane
DMA = dimethylacetamide
DMF = dimethylformamide
DMSO = dimethyl sulfoxide
DMA = dichloromethane
EtOAc = ethyl acetate
MeOH = methanol
PTFE = polytetrafluoroethylene
RBF= Round Bottom Flask
TLC = thin layer chromatography

I-D. General Photoredox Reaction Setup

The reactions were ran in a shallow oil bath at elevated temperatures (Photo 1). A 15 W LED array lamp was used as the light source. It was positioned approximately 6 inches above the reaction vials (Photo 2).

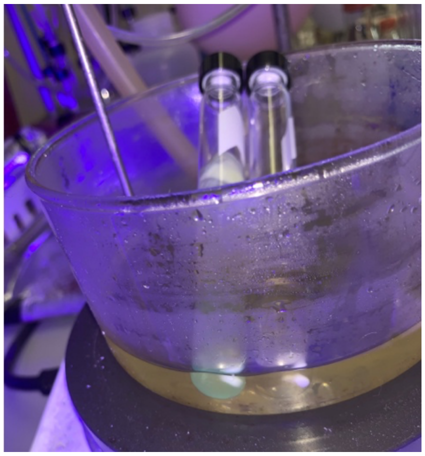

Photo 1.

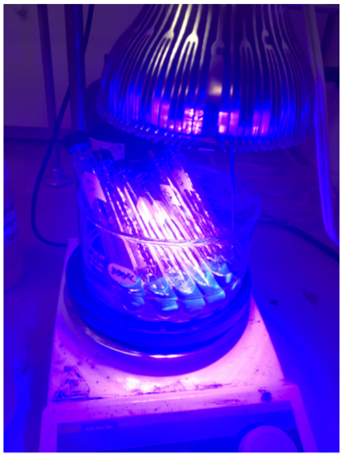

Photo 2.

II. Optimization Details

Optimization Procedure

An 8 mL oven-dried screw-top test tube was charged with 4CzIPN (1 mol%, 0.001 mol), sodium formate (4.0 equiv, 0.4 mmol), mesna (20 mol%, 0.02 mol), and (*E*)-3-phenyl-*N*-(piperidin-1-yl)propan-1-imine **S6** (1.0 equiv, 0.1 mmol). The tube was equipped with an oven-dried stir bar and sealed with a PTFE/silicon septum. Under nitrogen atmosphere, separately degassed DMSO (1 mL) was added via syringe, followed by 1,3-bis(trifluoromethyl)benzene (3.0 equiv, 0.3 mmol). The resulting mixture was heated to 80 °C in an oil bath and stirred at 700 RPM for 16 h under irradiation by blue LEDs. The reaction was cooled to room temperature and 2-(trifluoromethyl)pyridine (0.1 mmol) was added as the internal standard. A small aliquot of the reaction mixture was diluted in d_6-DMSO and the sample was analyzed by ^{19}F NMR and the integral values were used to calculate the data given in Table S1.

Note: Due to the slight hygroscopic nature of formate, we stored sodium formate in the desiccator for consistent results. Bottles of formate that had been sitting out on benchtop for longer periods of time gave less consistent results.

Table S1.

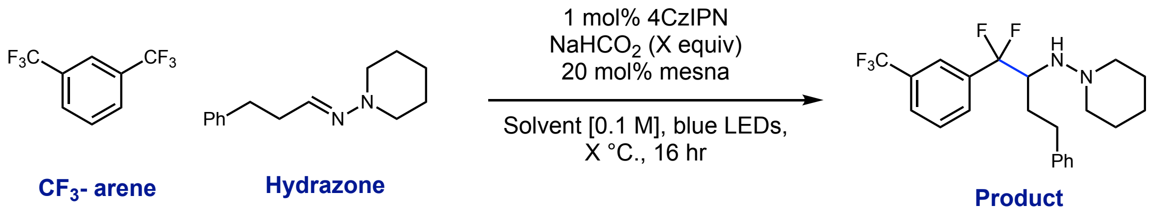

Entry	CF_3 arene	Hydrazone	Formate	Solvent	Temp.	Other	Product*
1	3.0 equiv	1.0 equiv	4.0 equiv	DMSO	80 °C	-	**99%**
2	1.0 equiv	3.0 equiv	4.0 equiv	DMSO	80 °C	-	**74%**
3	3.0 equiv	1.0 equiv	4.0 equiv	DMSO	23 °C	-	**54%**
4	1.0 equiv	3.0 equiv	4.0 equiv	DMSO	23 °C	-	**39%**
5	3.0 equiv	1.0 equiv	4.0 equiv	20% H_2O/DMSO	80 °C	-	**38%**
6	3.0 equiv	1.0 equiv	3.0 equiv	DMSO	80 °C	-	**55%**
7	3.0 equiv	1.0 equiv	5.0 equiv	DMSO	80 °C	-	**97%**
8	3.0 equiv	1.0 equiv	4.0 equiv	DMSO	80 °C	open to air	**89%**
9	3.0 equiv	1.0 equiv	4.0 equiv	DMSO	80 °C	no 4CzIPN	**3%**
10	3.0 equiv	1.0 equiv	4.0 equiv	DMSO	80 °C	no light	**0%**
11	3.0 equiv	1.0 equiv	none	DMSO	80 °C	no formate	**3%**
12	3.0 equiv	1.0 equiv	4.0 equiv	DMSO	80 °C	no mesna	**13%**
13	3.0 equiv	1.0 equiv	4.0 equiv	DMF	80 °C	-	**93%**
14	3.0 equiv	1.0 equiv	4.0 equiv	DMA	80 °C	-	**91%**
15	3.0 equiv	1.0 equiv	4.0 equiv	MeCN	80 °C	-	**0%**

*Yields determined by ^{19}F NMR using 2-(trifluoromethyl)pyridine as internal standard

III. Preparation of Starting Materials

III-A. Preparation of CF_3-Arenes

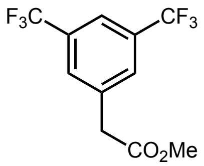

methyl 2-(3,5-bis(trifluoromethyl)phenyl)acetate (**S1**): To a solution of 2-(3,5-bis(trifluoromethyl)phenyl)acetic acid (5.00 mmol, 1.36 g, 1.0 equiv) dissolved in MeOH (12 mL) was added a few drops of conc. HCl. The reaction was stirred overnight under reflux using a heating mantle. The reaction was allowed to cool to temperature. The reaction was concentrated *in vacuo*, washed with sat. $NaHCO_3$ (3x) and extracted with EtOAc (2x), dried over Na_2SO_4 and concentrated *in vacuo*. The reaction was passed with through a plug of silica gel (20-30% EtOAc/hexanes as eluent) to afford the title compound as a clear oil (1.22 g, 85% yield). The physical properties and spectral data were consistent with reported values.[2]
^{1}H NMR (600 MHz, $CDCl_3$) δ 7.81 (s, 1H), 7.75 (s, 2H), 3.77 (s, 2H), 3.74 (s, 3H).

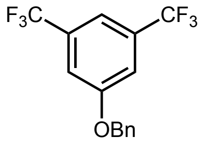

1-(benzyloxy)-3,5-bis(trifluoromethyl)benzene (S2): An oven-dried round bottom flask was charged with 3,5-bis(trifluoromethyl)phenol (7.14 mmol. 1.64 g, 1.02 equiv), benzyl bromide (7.00 mmol, 0.831 mL, 1.0 equiv) and potassium carbonate (21.0 mmol, 2.90 g, 3.0 equiv) and dissolved in acetone (30 mL). The reaction was stirred overnight under reflux using a heating mantle. The reaction was allowed to cool to room temperature. The reaction was filtered and concentrated *in vacuo*. The reaction was purified via silica chromatography (5-10% EtOAc/hexanes as eluent) to afford the title compound as a clear oil (1.92 g, 86% yield). The physical properties and spectral data were consistent with reported values.[3]
^{1}H NMR (500 MHz, $CDCl_3$) δ 7.48 (br s, 1H), 7.46 – 7.35 (m, 7H), 5.14 (s, 2H).

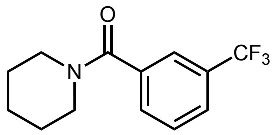

piperidin-1-yl(3-(trifluoromethyl)phenyl)methanone (S3): To a solution of piperidine (5.50 mmol, 0.543 mL, 1.1 equiv) and triethylamine (5.00 mmol, 0.697 mL, 1.0 equiv) in an RBF was added 3-(trifluoromethyl)benzoyl chloride (5.00 mmol, 0.834 mL, 1.0 equiv) at 0 °C. The reaction was warmed to room temperature and stirred overnight. The reaction mixture was washed with 1 M HCl (3x), dried over Na_2SO_4 and concentrated *in vacuo*. The reaction was pushed through a plug of silica gel (30% EtOAc/hexanes as eluent) to afford the title compound as a clear oil (1.30 g, quantitative). The physical properties and spectral data were consistent with reported values.[4]

^{1}H NMR (400 MHz, CDCl$_3$) δ 7.71 – 7.62 (m, 2H), 7.62 – 7.49 (m, 2H), 3.72 (br s, 2H), 3.32 (br s, 2H), 1.76 – 1.44 (m, 6H).

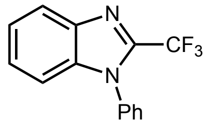

1-phenyl-2-(trifluoromethyl)-1H-benzo[d]imidazole (S4): The title compound was prepared according to literature procedure. The physical properties and spectral data were consistent with reported values.[5]
^{1}H NMR (500 MHz, CDCl$_3$) δ 8.04 (d, *J* = 8.1 Hz, 1H), 7.67 – 7.57 (m, 3H), 7.52 – 7.40 (m, 4H), 7.19 (d, *J* = 8.1 Hz, 1H).

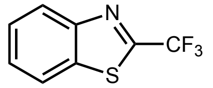

2-(trifluoromethyl)benzo[*d*]thiazole (S5): The title compound was prepared according to literature procedure. The physical properties and spectral data were consistent with reported values.[5]
^{1}H NMR (500 MHz, CDCl$_3$) δ 8.21 (d, *J* = 8.2 Hz, 1H), 8.00 (d, *J* = 8.1 Hz, 1H), 7.64- 7.61 (m, 1H), 7.61 – 7.48 (m, 1H).

III-B. Preparation of Hydrazones

General Procedure SA

In a screw-top test tube equipped with a stirbar was added a solution of aldehyde (1 equiv) dissolved in DCM. The hydrazine (1.1 equiv) was added to the reaction dropwise followed by the addition of sodium sulfate and stirred overnight. The reaction was filtered and concentrated *in vacuo.* The crude reaction mixture was purified by silica chromatography to afford the corresponding hydrazone product.

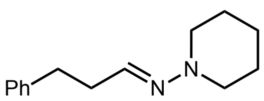

(*E*)-3-phenyl-*N*-(piperidin-1-yl)propan-1-imine (S6): Prepared according to general procedure SA (addition of hydrazine at 0 °C) using 3-phenylpropanal (25.0 mmol, 3.32 mL, 1.0 equiv), N-aminopiperidine (30.0 mmol, 3.24 mL, 1.1 equiv) and DCM (20 mL). The crude reaction was purified by silica chromatography (5-30% EtOAc/hexanes as eluent) to afford the title compound as a yellow oil. (3.36 g, 62% yield). The physical properties and spectral data were consistent with the reported values.[6]
^{1}H NMR (400 MHz, CDCl$_3$) δ 7.31 – 7.26 (m, 2H), 7.24 – 7.14 (m, 3H), 6.95 (t, *J* = 5.4 Hz, 1H), 2.91 (t, J= 5.7 Hz, 4H), 2.84- 2.78 (m, 2H), 2.61 – 2.51 (m, 2H), 1.69 (p, *J* = 5.8 Hz, 4H), 1.52 – 1.42 (m, 2H).

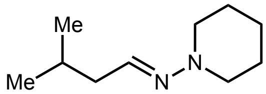

(*E*)-3-methyl-*N*-(piperidin-1-yl)butan-1-imine (S7): Prepared according to general procedure SA using 3-methylbutanal (4.00 mmol, 0.439 mL, 1.0 equiv), N-aminopiperidine (4.40 mmol, 0.475 mL, 1.1 equiv) and DCM (3 mL). The crude reaction was purified by silica chromatography (10-30% EtOAc/hexanes as eluent) to afford the title compound as a clear oil. (507 mg, 75% yield).The physical properties and spectral data were consistent with the reported values.[6]

^{1}H NMR (500 MHz, $CDCl_3$) δ 6.96 (t, *J* = 5.9 Hz, 1H), 2.91 (t, J=5.5Hz, 4H), 2.13-2.11 (m, 2H) 1.83-1.75 (m1H), 1.69 (p, *J* = 5.8 Hz, 4H), 1.51 – 1.42 (m, 2H), 0.93 (d, *J* = 6.7 Hz, 6H).

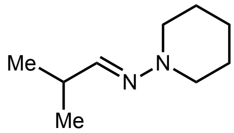

(*E*)-2-methyl-*N*-(piperidin-1-yl)propan-1-imine (**S8**): Prepared according to general procedure SA using isobutyraldehyde (5.00 mmol, 0.460 mL, 1.0 equiv), N-aminopiperidine (5.50 mmol, 0.594 mL, 1.1 equiv) and DCM (5 mL). The crude reaction was purified by silica chromatography (10-20% EtOAc/hexanes as eluent) to afford the title compound as a clear oil. (370 mg, 48% yield).The physical properties and spectral data were consistent with the reported values.[6]

^{1}H NMR (500 MHz, $CDCl_3$) δ 6.80 (s, 1H), 2.89 (t, *J* = 5.6 Hz, 4H), 2.53 – 2.42 (m, 1H), 1.69 (p, *J* = 5.9 Hz, 4H), 1.52 – 1.42 (m, 2H), 1.05 (d, *J* = 6.8 Hz, 6H).

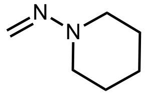

***N*-(piperidin-1-yl)methanimine (S9):** Prepared according to a slightly modified version of general procedure SA using paraformaldehyde (15.0 mmol, 450 mg, 1.5 equiv), N-aminopiperidine (10.0 mmol, 1.08 mL, 1.0 equiv) and Et_2O (10 mL). The crude reaction was purified by silica chromatography (70% Et_2O/hexanes as eluent) to afford the title compound as a clear oil (599 mg, 53% yield). The physical properties and spectral data were consistent with the reported values.[7]

^{1}H NMR (500 MHz, $CDCl_3$) δ 6.46 (d, *J* = 11.1 Hz, 1H), 6.28 (d, *J* = 11.1 Hz, 1H), 2.99 (t, *J* = 5.7 Hz, 3H), 1.70 (p, *J* = 5.8 Hz, 2H), 1.54 – 1.46 (m, 1H).

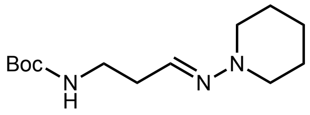

***tert*-butyl (*E*)-(3-(piperidin-1-ylimino)propyl)carbamate (S10):** Prepared according to general procedure SA using *tert*-butyl (3-oxopropyl)carbamate (4.00 mmol, 0.690 g, 1.0 equiv), N-aminopiperidine (4.40 mmol, 0.470 mL, 1.1 equiv) and DCM (4 mL). The crude reaction was purified via silica chromatography (20-50% EtOAc/hexanes as eluent) to afford the title compound as a clear oil (0.629 g, 62% yield).

R_f= 0.34 (60% EtOAc/hexanes)

^{1}H NMR (500 MHz, CDCl$_3$) δ 6.90 (t, *J* = 4.8 Hz, 1H), 3.34 (q, *J* = 6.3 Hz, 2H), 2.95 – 2.87 (t, J= 5.7 Hz, 4H), 2.40 (td, *J* = 6.3, 4.6 Hz, 2H), 1.73 – 1.65 (p, J= 5.7 Hz, 4H), 1.51 – 1.44 (m, 2H), 1.42 (s, 9H).
^{13}C NMR (101 MHz, CDCl$_3$) δ 156.0, 136.9, 79.2, 52.7, 38.1, 33.7, 28.6, 25.4, 24.3.
HRMS (APCI) *m/z*: [M+H] calcd. for $C_{13}H_{26}O_2N_3$, 256.2019; found 256.2018.

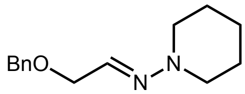

(*E*)-2-(benzyloxy)-*N*-(piperidin-1-yl)ethan-1-imine (S11): Prepared according to general procedure SA using 2-(benzyloxy)acetaldehyde (5.00 mmol, 0.702 mL, 1.0 equiv), N-aminopiperidine (5.50 mmol, 0.594 mL, 1.1 equiv) and DCM (5 mL). The crude reaction was purified by silica chromatography (10-20% EtOAc/hexanes as eluent) to afford the title compound as a yellow oil (767 mg, 66% yield).
R_f= 0.48 (20% EtOAc/hexanes)
^{1}H NMR (500 MHz, CDCl$_3$) δ 7.38 – 7.32 (m, 4H), 7.30 – 7.26 (m, 1H), 6.90 (t, *J* = 5.2 Hz, 1H), 4.54 (s, 2H), 4.17 (d, *J* = 5.3 Hz, 2H), 3.02 – 2.96 (t, J=5.6 Hz, 4H), 1.69 (p, *J* = 5.9 Hz, 4H), 1.53 – 1.45 (m, 2H).
^{13}C NMR (101 MHz, CDCl$_3$) δ 138.3, 134.0, 128.5, 128.0, 127.7, 72.5, 71.0, 52.0, 25.2, 24.1.
HRMS (APCI) *m/z*: [M+H] calcd. for $C_{14}H_{21}ON_2$, 233.16484; found 233.16529.

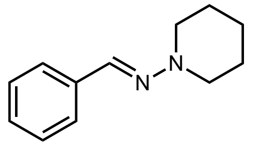

(*E*)-1-phenyl-*N*-(piperidin-1-yl)methanimine (S12): Prepared according to general procedure SA using benzaldehyde (10.0 mmol, 1.00 mL, 1.0 equiv), N-aminopiperidine (12.0 mmol, 1.30 mL, 1.2 equiv) and DCM (6 mL). The reaction was concentrated *in vacuo* without further purification to afford the title compound as a colorless solid. (1.76 g, 93% yield). The physical properties and spectral data were consistent with the reported values.[8]
^{1}H NMR (500 MHz, CDCl$_3$) δ 7.61 – 7.57 (m, 2H), 7.55 (s, 1H), 7.33 (t, *J* = 7.6 Hz, 2H), 7.26 – 7.21 (m, 1H), 3.18 (t, J= 5.6 Hz, 4H), 1.75 (p, J= 5.7 Hz, 4H), 1.64 – 0.88 (m, 2H).

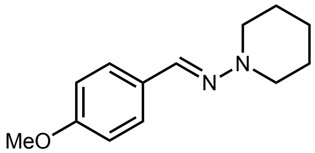

(*E*)-1-(4-methoxyphenyl)-*N*-(piperidin-1-yl)methanimine (S13): Prepared according to general procedure SA using 4-methoxybenzaldehyde (4.00 mmol, 0.486 mL, 1.0 equiv), N-aminopiperidine (4.80 mmol, 0.518 mL, 1.2 equiv) and DCM (2.5 mL). The crude reaction was purified by silica chromatography (5-20% EtOAc/hexanes as eluent) to afford the title compound as a clear oil. (672 mg, 78% yield). The physical properties and spectral data were consistent with the reported values.[9]
^{1}H NMR (500 MHz, CDCl$_3$) δ ,7.55 (s, 1H), 7.53 (d, *J* = 9.0 Hz, 2H), 6.87 (d, *J* = 9.0 Hz, 2H), 3.81 (s, 3H), 3.12 (t, J=5.6 Hz, 4H), 1.75 (p, *J* = 5.8 Hz, 4H), 1.57 – 1.49 (m, 2H).

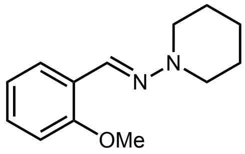

(*E*)-1-(2-methoxyphenyl)-*N*-(piperidin-1-yl)methanimine (S14): Prepared according to general procedure SA using 2-methoxybenzaldehyde (5.00 mmol, 0.602 mL, 1.0 equiv), N-aminopiperidine (5.50 mmol, 0.594 mL, 1.2 equiv) and DCM (5 mL). The crude reaction was purified by silica chromatography (0-20% EtOAc/hexanes as eluent) to afford the title compound as a yellow oil (1.05 g, 96% yield).
R_f= 0.62 (20% EtOAc/hexanes)
^{1}H NMR (500 MHz, $CDCl_3$) δ 7.92 (s, 1H), 7.87 (dd, *J* = 7.7, 1.7 Hz, 1H), 7.24 (t, J= 7.7 Hz, 1H), 6.94 (t, *J* = 7.5 Hz, 1H), 6.86 (d, *J* = 8.3 Hz, 1H), 3.85 (s, 3H), 3.16 (t, *J* = 5.7 Hz, 4H), 1.76 (p, *J* = 5.5 Hz, 4H), 1.54 (m, 3H).
^{13}C NMR (101 MHz, $CDCl_3$) δ 157.0, 130.8, 128.9, 125.4, 125.4, 121.0, 110.9, 55.6, 52.4, 25.4, 24.4.
HRMS (APCI) *m/z*: [M+H] calcd. for $C_{13}H_{19}ON_2$, 219.1492; found 219.1497.

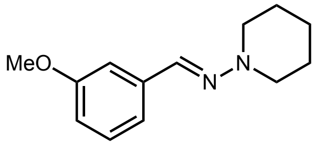

(*E*)-1-(3-methoxyphenyl)-*N*-(piperidin-1-yl)methanimine (S15): Prepared according to general procedure SA using 3-methoxybenzaldehyde (5.00 mmol, 0.582 mL, 1.0 equiv), N-aminopiperidine (5.50 mmol, 0.594 mL, 1.2 equiv) and DCM (5 mL). The crude reaction was pushed through a silica plug (20% EtOAc/hexanes as eluent) to afford the title compound as a yellow oil (1.01 g, 93% yield).
R_f= 0.58 (20% EtOAc/hexanes)
^{1}H NMR (500 MHz, $CDCl_3$) δ 7.52 (s, 1H), 7.24 (t, *J* = 8.1 Hz, 1H), 7.22 – 7.20 (m, 1H), 7.12 (d, *J* = 7.6 Hz, 1H), 6.81 (dd, *J* = 8.2, 2.7 Hz, 1H), 3.83 (s, 3H), 3.19 – 3.13 (m, 4H), 1.76 (p, *J* = 5.7 Hz, 4H), 1.60 – 1.50 (m, 2H).
^{13}C NMR (101 MHz, $CDCl_3$) δ 160.0, 138.3, 134.5, 129.5, 119.3, 114.4, 110.1, 55.4, 52.2, 25.3, 24.3.
HRMS (APCI) *m/z*: [M+H] calcd. for $C_{13}H_{19}ON_2$, 219.1492; found 219.1495.

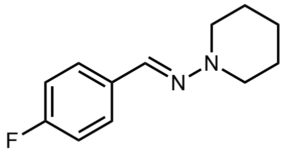

(*E*)-1-(4-fluorophenyl)-*N*-(piperidin-1-yl)methanimine (S16): Prepared according to general procedure SA using 4-fluorobenzaldehyde (5.00 mmol, 0.535 mL, 1.0 equiv), N-aminopiperidine (5.50 mmol, 0.594 mL, 1.1 equiv) and DCM (5 mL). The crude reaction was purified by silica chromatography (0-20% EtOAc/hexanes as eluent) to afford the title compound as a colorless solid (937 mg, 91% yield).
R_f= 0.78 (20% EtOAc/hexanes)

^{1}H NMR (500 MHz, CDCl$_3$) δ 7.60 – 7.52 (m, 2H), 7.51 (s, 1H), 7.06 – 6.97 (m, 2H), 3.14 (t, J=5.6 Hz, 4H), 1.75 (p, *J* = 5.8 Hz, 4H), 1.60 – 1.50 (m, 2H).
^{13}C NMR (101 MHz, CDCl$_3$) δ 162.7 (d, *J* = 246.8 Hz), 133.6 (d, *J* = 1.0 Hz), 133.1 (d, *J* = 3.2 Hz), 127.6 (d, *J* = 8.0 Hz), 115.6 (d, *J* = 21.7 Hz), 52.3, 25.4, 24.5.
^{19}F NMR (376 MHz, CDCl$_3$) δ -114.19 (tt, *J* = 8.7, 5.5 Hz).
HRMS (APCI) *m/z*: [M+H] calcd. for $C_{12}H_{16}N_2F$, 207.1292; found 207.1297.

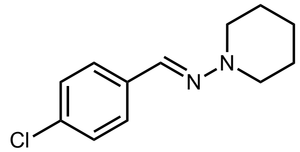

(*E*)-1-(4-chlorophenyl)-*N*-(piperidin-1-yl)methanimine (S17): Prepared according to general procedure SA using 4-chlorobenzaldehyde (5.00 mmol, 0.582 mL, 1.0 equiv), N-aminopiperidine (5.50 mmol, 0.594 mL, 1.1 equiv) and DCM (5 mL). The crude reaction was purified via silica chromatography (10-20% EtOAc/hexanes as eluent) to afford the title compound as a colorless solid (1.03 g, 92% yield).
R_f= 0.73 (20% EtOAc/hexanes)
^{1}H NMR (500 MHz, CDCl$_3$) δ 7.51 (d, *J* = 8.4 Hz, 2H), 7.47 (s, 1H), 7.29 (d, *J* = 8.6 Hz, 2H), 3.20 – 3.09 (t, J= 5.6 Hz, 4H), 1.81 – 1.71 (p, J= 5.8 Hz, 4H), 1.61 – 1.40 (m, 2H).
^{13}C NMR (101 MHz, CDCl$_3$) δ 135.5, 133.3, 133.0, 128.8, 127.2, 52.1, 25.3, 24.2.
HRMS (APCI) *m/z*: [M+H] calcd. for $C_{12}H_{16}N_2Cl$, 223.0997; found 223.0994.

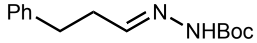

***tert*-butyl (*E*)-2-(3-phenylpropylidene)hydrazine-1-carboxylate (S18):** Prepared according to general procedure SA using 3-phenylpropanal (3.00 mmol, 0.395 mL, 1.0 equiv), tert-butyl hydrazinecarboxylate (3.0 mmol, 396 mg, 1.0 equiv) and DCM (3 mL). The crude reaction was purified via silica chromatography (10-50% EtOAc/hexanes as eluent) to afford the title compound as a colorless solid (739 mg, 99% yield). The physical properties and spectral data were consistent with the reported values.[10]
^{1}H NMR (500 MHz, CDCl$_3$) δ 7.33 – 7.27 (m, 2H), 7.23 – 7.17 (m, 3H), 7.15 (br s, 1H), 2.88 – 2.81 (m, 2H), 2.67 – 2.59 (m, 2H), 1.50 (s, 9H).

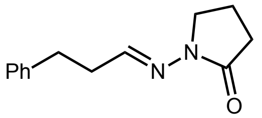

(*E*)-1-((3-phenylpropylidene)amino)pyrrolidin-2-one (S19): Prepared according to general procedure SA using 3-phenylpropanal (3.00 mmol, 0.395 mL, 1.0 equiv), 1-Aminopyrrolidin-2-one hydrochloride (3.30 mmol, 450 mg, 1.1 equiv) and DCM (3 mL). The crude reaction was purified via silica chromatography (50-100% EtOAc/hexanes as eluent) to afford the title compound as an off white solid (383 mg, 59% yield).
R_f= 0.07 (100% EtOAc/hexanes)
^{1}H NMR (500 MHz, CDCl$_3$) δ 7.29 (t, *J* = 6.5 Hz, 2H), 7.24 – 7.18 (m, 3H), 7.18- 7.13 (m, 1H), 3.60 – 3.49 (m, 2H), 2.87 (t, J= 7.6 Hz, 2H), 2.78 – 2.69 (m, 2H), 2.58 – 2.50 (m, 2H), 2.20 – 2.08 (m, 2H).

^{13}C NMR (101 MHz, CDCl$_3$) δ 171.5, 148.5, 140.8, 128.7, 128.5, 126.4, 44.7, 34.6, 33.4, 30.2, 16.2.
HRMS (APCI) *m/z*: [M+H] calcd. for $C_{13}H_{17}ON_2$, 217.1335; found 217.1335.

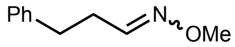

3-phenylpropanal *O*-methyl oxime (S20): An RBF was charged with sodium acetate (7.50 mmol, 615 mg, 1.5 equiv), methoxyamine hydrochloride (7.50 mmol, 627 mg, 1.5 equiv) and dissolved in MeOH/H_2O (10 mL, 9:1). 3-phenylpropanal (5.00 mmol, 0.658 mL, 1.0 equiv) was added dropwise to the mixture. The reaction was stirred overnight at room temperature. The reaction was concentrated, diluted in H_2O and extracted with EtOAc (3x). The combined organic phase was washed with H_2O (2x) and brine (1x), dried over Na_2SO_4 and concentrated *in vacuo*. The reaction was purified by silica chromatography (5-20% EtOAc/hexanes) to afford the title compound as a clear oil (572 mg, 70% yield, 1.0 : 1.4 dr). The physical properties and spectral data were consistent with the reported values.[5] (#denotes major diastereomer, *denotes minor diastereomer)
^{1}H NMR (500 MHz, CDCl$_3$) δ 7.41 (t, *J* = 6.0 Hz, 1H, #), 7.34 – 7.27 (m, 2H, #*), 7.23 – 7.13 (m, 3H, #*), 6.72 – 6.63 (t, J=5.36 Hz, 1 H, *), 3.87 (s, 3H, *), 3.82 (s, 3H, #), 2.85 – 2.77 (m, 2H, *#), 2.69 – 2.61 (m, 2H, *), 2.55 – 2.47 (m, 2H, #).

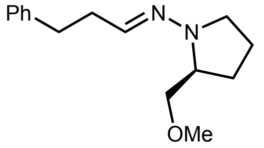

(*S*,*E*)-*N*-(2-(methoxymethyl)pyrrolidin-1-yl)-3-phenylpropan-1-imine (S21): Prepared according to general procedure SA using 3-phenylpropanal (3.00 mmol, 0.395 mL, 1.0 equiv), (*S*)-2-(methoxymethyl)pyrrolidin-1-amine (3.30 mmol, 0.440 mL, 1.1 equiv) and DCM (5 mL). The crude reaction was purified via silica chromatography (20-30% EtOAc/hexanes as eluent) to afford the title compound as a clear oil (680 mg, 92% yield). The physical properties and spectral data were consistent with the reported values.[11]
^{1}H NMR (500 MHz, CDCl$_3$) δ 7.31 – 7.26 (m, 2H), 7.23 – 7.14 (m, 3H), 6.66 (t, J= 5.4 Hz, 1H), 3.61 – 3.50 (m, 1H), 3.44 – 3.39 (m, 2H), 3.38 (s, 3H), 3.36 – 3.31 (m, 1H), 2.80 (dd, *J* = 9.2, 6.7 Hz, 2H), 2.71 (q, *J* = 8.4 Hz, 1H), 2.54 (dd, *J* = 13.1, 7.8 Hz, 2H), 2.00 – 1.84 (m, 3H), 1.84 – 1.73 (m, 1H).

IV. Preparation of Products from Substrate Table

IV-A. General Procedures

General Procedure A

A 15 mL oven-dried screw-top test tube was charged with 4CzIPN (1 mol%), sodium formate (4.0 equiv), mesna (20 mol%) hydrazone (1.0 equiv, *if solid or oil*) and CF_3-arene (3.0 equiv, *if solid or oil*). The tube was equipped with a stir bar and was sealed with a PTFE/silicon septum. The atmosphere was exchanged by applying vacuum and backfilling with nitrogen (this process was conducted a total of three times). Under nitrogen atmosphere, the degassed solvent (DMSO, 0.1 M) was added via syringe followed by hydrazone (1.0 equiv, *if liquid*) and CF_3-arene (3.0 equiv, *if liquid*) The resulting mixture was stirred for 16 h under irradiation by blue LEDs at 80 °C in an oil bath. Upon completion, the reaction was diluted with H_2O and extracted with EtOAc (3x). The combined organic layers were passed through silica to remove excess DMSO and concentrated *in vacuo*. The residue was then purified by silica chromatography using the indicated solvent mixture as the eluent to afford the title compound.

General Procedure B

A 15 mL oven-dried screw-top test tube was charged with 4CzIPN (1 mol%), sodium formate (4.0 equiv), mesna (20 mol%), hydrazone (1.0 equiv, *if solid or oil*) and CF_3-arene (3.0 equiv, *if solid or oil*). The tube was equipped with a stir bar and was sealed with a PTFE/silicon septum. The atmosphere was exchanged by applying vacuum and backfilling with nitrogen (this process was conducted a total of three times). Under nitrogen atmosphere, the degassed solvent (DMSO, 0.1 M) was added via syringe followed by hydrazone (1.0 equiv, *if liquid*), CF_3-arene (3.0 equiv, *if liquid*) and formic acid (3.0 equiv). The resulting mixture was stirred for 16 h under irradiation by blue LEDs at 80 °C in an oil bath. Upon completion, the reaction was diluted with H_2O and extracted with EtOAc (3x). The combined organic layers were passed through silica to remove excess DMSO and concentrated *in vacuo*. The residue was then purified by silica chromatography using the indicated solvent mixture as the eluent to afford the title compound.

IV-B. Isolated Products from Substrate Table

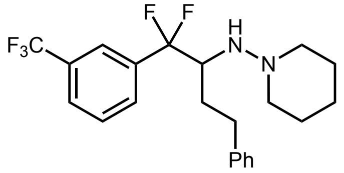

***N*-(1,1-difluoro-4-phenyl-1-(3-(trifluoromethyl)phenyl)butan-2-yl)piperidin-1-amine (3):** Prepared according to general procedure A using (E)-3-phenyl-N-(piperidin-1-yl)propan-1-imine **S6** (0.500 mmol, 108 mg, 1.0 equiv), 1,3-bis(trifluoromethyl)benzene (1.50 mmol, 0.232 mL, 3.0 equiv), 4CzIPN (5.00 μmol, 3.93 mg, 1 mol%), sodium formate (2.00 mmol, 136 mg, 4.0 equiv), mesna (0.100 mmol, 16.4 mg, 20 mol%) in DMSO (5 mL). After 16 hours the reaction was worked up and purified by silica chromatography (10% EtOAc/hexanes as eluent) to afford the title compound as yellow oil (169 mg, 82% yield).

R_f= 0.70 (20% EtOAc/hexanes)

^{1}H NMR (600 MHz, $CDCl_3$) δ 7.78 (s, 1H), 7.67 (t, *J* = 8.9 Hz, 2H), 7.50 (t, *J* = 7.8 Hz, 1H), 7.33 – 7.24 (m, 2H), 7.21 (t, *J* = 7.4 Hz, 1H), 7.17 (d, *J* = 6.9 Hz, 2H), 3.39 – 3.29 (m, 1H), 2.84 – 2.76 (m, 1H), 2.76 – 2.67 (m, 1H), 2.59 – 2.02 (m, 5H), 1.68 – 1.57 (m, 1H), 1.29 (m, 6H).

^{13}C NMR (151 MHz, $CDCl_3$) δ 141.3, 137.8- 137.4 (m), 130.4 (q, *J* = 32.6 Hz), 129.6 (t, *J* = 6.5 Hz), 128.7, 128.5, 128.4, 126.3, 126.3 (m), 124.1 (q, *J* = 272.0 Hz), 123.6 (dt, *J* = 8.8, 4.1 Hz), 122.1 (dd, *J* = 249.1, 245.2 Hz), 62.7 (dd, *J* = 29.3, 26.0 Hz), 57.2, 32.7, 29.7, 26.1, 23.8.

^{19}F NMR (376 MHz, $CDCl_3$) δ -62.69 (s, 3F), -97.89 (dd, *J* = 246.9, 7.6 Hz, 1F), -111.71 (dd, *J* = 247.3, 16.3 Hz, 1F).

HRMS (APCI) *m/z*: [M+H] calcd. for $C_{22}H_{26}N_2F_5$, 413.2011; found 413.2012.

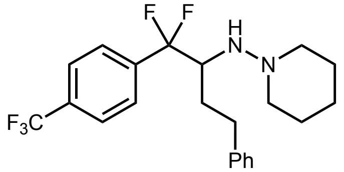

***N*-(1,1-difluoro-4-phenyl-1-(4-(trifluoromethyl)phenyl)butan-2-yl)piperidin-1-amine (4):** Prepared according to general procedure B using (E)-3-phenyl-N-(piperidin-1-yl)propan-1-imine **S6** (0.250 mmol, 54.1 mg, 1.0 equiv), 1,4-Bis(trifluoromethyl)benzene (0.750 mmol, 0.116 mL, 3.0 equiv), 4CzIPN (2.50 μmol, 2.0 mg, 1 mol%), sodium formate (1.00 mmol, 68.0 mg, 4.0 equiv), mesna (0.05 mmol, 8.20 mg, 20 mol%), formic acid (0.750 mmol, 28.0 μL, 3.0 equiv) in DMSO (2.5 mL). After 16 hours the reaction was worked up and purified by silica chromatography (0-10% EtOAc/hexanes as eluent with 5% triethylamine) to afford the title compound as yellow oil (75.2 mg, 73% yield).

R_f= 0.61 (10% EtOAc/ hexanes)

^{1}H NMR (500 MHz, $CDCl_3$) δ 7.74 – 7.50 (m, 4H), 7.28 (m, 2H), 7.23 – 7.18 (m, 1H), 7.18 – 7.14 (m, 2H), 3.39 – 3.29 (m, 1H), 2.80 (m, 1H), 2.75 – 2.65 (m, 1H), 2.58 – 2.12 (m, 4H), 2.11 – 1.99 (m, 1H), 1.66 – 1.55 (m, 1H), 1.44 – 1.19 (m, 6H).

^{13}C NMR (151 MHz, $CDCl_3$) δ 141.3, 140.0 (t, *J* = 26.4 Hz), 131.7 (q, *J* = 32.5 Hz), 128.6, 128.5, 126.9 (dd, *J* = 7.6, 5.4 Hz), 126.3, 124.8 (q, *J* = 3.8 Hz), 124.0 (q, *J* = 272.6 Hz), 122.2 (dd, *J* = 248.8, 245.5 Hz), 62.8 (t, *J* = 27.4 Hz), 57.3, 32.7, 29.8, 26.1, 23.8.

^{19}F NMR (376 MHz, $CDCl_3$) δ -62.72 (s, 3F), -99.27 (dd, *J* = 247.3, 8.7 Hz, 1F), -110.34 (dd, *J* = 247.3, 14.9 Hz, 1F).

HRMS (APCI) *m/z*: [M+H] calcd. for $C_{22}H_{26}N_2F_5$, 413.2011; found 413.2013.

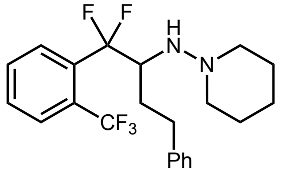

***N*-(1,1-difluoro-4-phenyl-1-(2-(trifluoromethyl)phenyl)butan-2-yl)piperidin-1-amine (5):** Prepared according to general procedure B using (E)-3-phenyl-N-(piperidin-1-yl)propan-1-imine **S6** (0.500 mmol, 54.1 mg, 1.0 equiv), 1,2-bis(trifluoromethyl)benzene (1.50 mmol, 0.233 mL, 3.0 equiv), 4CzIPN (5.00 µmol, 3.90 mg, 1 mol%), sodium formate (2.00 mmol, 0.140 g, 4.0 equiv), mesna (0.100 mmol, 16.4 mg, 20 mol%), formic acid (1.50 mmol, 56.0 µL, 3.0 equiv) in DMSO (5 mL). After 16 hours the reaction was worked up and purified by silica chromatography (0-10% EtOAc/hexanes as eluent). This was further purified by a second column (0-10% EtOAc/hexanes as eluent) to afford the title compound as a clear oil (110.3 mg, 54% yield).

^{1}H NMR (600 MHz, CDCl$_3$) δ 7.73 (t, *J* = 7.0 Hz, 2H), 7.56 (t, *J* = 7.6 Hz, 1H), 7.51 (t, *J* = 7.6 Hz, 1H), 7.35 – 7.24 (m, 2H), 7.23 – 7.17 (m, 3H), 3.58 – 3.49 (m, 1H), 2.84 – 2.76 (m, 1H), 2.75 – 2.67 (m, 1H), 2.57 – 1.84 (m, 5H), 1.80 – 1.71 (m, 1H), 1.41 – 0.85 (m, 6H).

^{13}C NMR (151 MHz, CDCl$_3$) δ 141.4, 135.4 (t, *J* = 27.1 Hz), 131.0, 129.47 (m), 129.45, 128.6, 128.5, 128.0 (qd, *J* = 32.1, 4.1 Hz), 127.1 (q, *J* = 6.6 Hz), 126.2, 124.0 (q, *J* = 273.4 Hz), 122.1 (dd, *J* = 250.4, 246.6 Hz), 62.1 (td, *J* = 27.9, 3.0 Hz), 56.7, 32.9, 29.3 (d, *J* = 5.5 Hz), 26.0, 23.8.

^{19}F NMR (376 MHz, CDCl$_3$) δ -57.30 (dd, *J* = 21.8, 7.3 Hz, 3F), -98.56 (dqd, *J* = 251.8, 21.2, 5.9 Hz, 1F), -105.05 (ddq, *J* = 251.8, 20.8, 7.3 Hz, 1F).

HRMS (APCI) *m/z*: [M+H] calcd. for $C_{22}H_{26}N_2F_5$, 413.2011; found 413.2012.

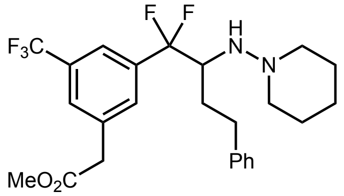

methyl 2-(3-(1,1-difluoro-4-phenyl-2-(piperidin-1-ylamino)butyl)-5 (trifluoromethyl)phenyl)acetate (**6**): Prepared according to general procedure B using (E)-3-phenyl-N-(piperidin-1-yl)propan-1-imine **S6** (0.100 mmol, 21.8 mg, 1.0 equiv), methyl 2-(3,5-bis(trifluoromethyl)phenyl)acetate **S1** (0.300 mmol, 85.9 mg, 3.0 equiv), 4CzIPN (1.00 µmol, 0.787 mg, 1 mol%), sodium formate (0.400 mmol, 27.2 mg, 4.0 equiv), mesna (20.0 µmol, 3.28 mg, 20 mol%), formic acid (0.300 mmol, 11.0 µL, 3.0 equiv) in DMSO (1 mL). After 16 hours the reaction was worked up and purified by silica prep plate (10% EtOAc/hexanes as eluent) to afford the title compound as a clear oil (35.0 mg, 72% yield).

R_f= 0.48 (20% EtOAc/hexanes)

^{1}H NMR (600 MHz, CDCl$_3$) δ 7.69 (s, 1H), 7.59 (s, 2H), 7.29 (t, *J* = 7.5 Hz, 2H), 7.20 (dd, *J* = 19.4, 7.5 Hz, 3H), 3.71 (s, 2H), 3.69 (s, 3H), 3.39 – 3.25 (m, 1H), 2.85 – 2.67 (m, 2H), 2.62 – 2.04 (m, 5H), 1.68 – 1.56 (m, 1H), 1.37 – 1.16 (m, 6H).

^{13}C NMR (151 MHz, CDCl$_3$) δ 170.9, 141.2, 138.0 (t, *J* = 26.8 Hz), 134.7, 130.7 (q, *J* = 32.6 Hz), 130.6 – 130.4 (m), 128.7, 128.5, 127.2 (m), 126.3, 123.8 (q, J= 272.5 Hz), 126.7 – 120.9

(m), 121.9 (dd, J=244.71, 248.93 Hz), 62.7 (dd, *J* = 29.3, 26.0 Hz), 57.3, 52.4, 40.9, 32.7, 29.5, 26.0, 23.8.

^{19}F NMR (376 MHz, CDCl$_3$) δ -62.61 (s, 3F), -97.79 (dd, *J* = 247.1, 7.5 Hz, 1F), -112.08 (dd, *J* = 246.7, 16.5 Hz, 1F).

HRMS (APCI) *m/z*: [M+H] calcd. for $C_{25}H_{30}O_2N_2F_5$, 485.2222; found 485.2222.

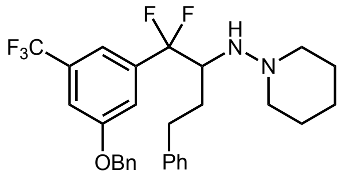

***N*-(1-(3-(benzyloxy)-5-(trifluoromethyl)phenyl)-1,1-difluoro-4-phenylbutan-2-yl)piperidin-1-amine (7):** Prepared according to general procedure B using (E)-3-phenyl-N-(piperidin-1-yl)propan-1-imine **S6** (0.500 mmol, 108 mg, 1.0 equiv), 1-(benzyloxy)-3,5-bis(trifluoromethyl)benzene **S2** (1.50 mmol, 0.480 g, 3.0 equiv), 4CzIPN (5.00 µmol, 3.90 mg, 1 mol%), sodium formate (2.00 mmol, 0.136 g, 4.0 equiv), mesna (0.100 mmol, 16.4 mg, 20 mol%), formic acid (1.50 mmol, 56 µL, 3.0 equiv) in DMSO (5 mL). After 16 hours the reaction was worked up and purified by silica chromatography (0-12% EtOAc/hexanes as eluent) to afford the title compound as a yellow oil (195 mg, 75% yield).

R$_f$=0.41 (20% EtOAc/hexanes)

^{1}H NMR (600 MHz, CDCl$_3$) δ 7.43 – 7.33 (m, 6H), 7.31 – 7.27 (m, 3H), 7.25 (s, 1H), 7.20 (t, *J* = 7.5 Hz, 1H), 7.16 (d, *J* = 7.8 Hz, 2H), 5.10 (s, 2H), 3.40 – 3.20 (m, 1H), 2.81 – 2.65 (m, 2H), 2.59 – 2.12 (m, 4H), 2.10 – 2.01 (m, 1H), 1.67 – 1.57 (m, 1H), 1.41 – 1.16 (m, 6H).

^{13}C NMR (101 MHz, CDCl$_3$) δ 158.6, 141.3, 139.1 (t, *J* = 26.9 Hz), 136.1, 131.6 (q, *J* = 32.6 Hz), 128.9, 128.6, 128.5, 127.7, 126.3, 123.8 (q, J= 272.9 Hz), 121.9 (dd, J= 246.0, 246.1 Hz), 116.2 (t, J=6.4 Hz), 116.1-115.8 (m), 112.9, 112.8, 70.6, 62.6 (dd, J= 28.37, 26.2 Hz), 57.3, 32.7, 29.7, 26.1, 23.8.

^{19}F NMR (376 MHz, CDCl$_3$) δ -62.72 (s, 3F), -98.65 (dd, *J* = 245.9, 9.0 Hz, 1F), -110.46 (dd, *J* = 246.2, 16.0 Hz, 1F).

HRMS (APCI) *m/z*: [M+H] calcd. for $C_{29}H_{32}ON_2F_5$, 519.2429; found 519.2432.

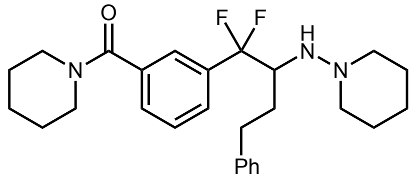

(3-(1,1-difluoro-4-phenyl-2-(piperidin-1-ylamino)butyl)phenyl)(piperidin-1-yl)methanone (8): Prepared according to general procedure A using ((E)-3-phenyl-N-(piperidin-1-yl)propan-1-imine **S6** (0.100 mmol, 216 mg, 1.0 equiv), piperidin-1-yl(3-(trifluoromethyl)phenyl)methanone **S3** (0.300 mmol, 771 mg, 3.0 equiv), 4CzIPN (1.00 µmol, 0.787 mg, 1 mol%), sodium formate (0.400 mmol, 27.2 mg, 4.0 equiv), and mesna (20.0 µmol, 3.28 mg, 20 mol%) in DMSO (1 mL). After 16 hours the reaction was worked up and purified by silica chromatography (2-30% EtOAc/DCM as eluent) followed by further purification by silica prep plate (5% EtOAc/DCM as eluent) to afford the title compound as clear oil (8.40 mg, 18% yield).

R$_f$= 0.41 (20% EtOAc/DCM)

^{1}H NMR (500 MHz, CDCl$_3$) δ 7.57 – 7.49 (m, 2H), 7.47 – 7.39 (m, 2H), 7.32 – 7.22 (m, 2H), 7.21 – 7.12 (m, 3H), 3.86 – 3.59 (m, 2H), 3.49 – 3.14 (m, 3H), 2.84 – 2.74 (m, 1H), 2.74 – 2.64 (m, 1H), 2.58 – 2.18 (m, 5H), 2.06 – 1.92 (m, 1H), 1.74 – 1.56 (m, 6H), 1.50 – 1.37 (m, 6H).
^{13}C NMR (101 MHz, CDCl$_3$) δ 169.7, 141.7, 136.6 (t, J= 26.5 Hz), 136.39, 129.0, 128.6, 128.5, 128.3, 128.2, 126.1, 124.8 (t, J= 6.7 Hz), 122.5 (dd, J= 246.01, 247.49 Hz), 62.8 (t, J= 27.0 Hz), 57.6, 32.8, 30.4, 30.1, 29.8, 26.2, 24.7, 23.9.
HRMS (APCI) *m/z*: [M+H] calcd. for $C_{27}H_{36}ON_3F_2$, 456.2821; found 456.2820.

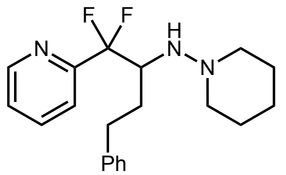

***N*-(1,1-difluoro-4-phenyl-1-(pyridin-2-yl)butan-2-yl)piperidin-1-amine (9):** Prepared according to general procedure A using (E)-3-phenyl-N-(piperidin-1-yl)propan-1-imine **S6** (0.500 mmol, 108 mg, 1.0 equiv), 2-(trifluoromethyl)pyridine (1.50 mmol, 0.173 mL, 3.0 equiv), 4CzIPN (5.00 µmol, 3.93 mg, 1 mol%), sodium formate (2.00 mmol, 136 mg, 4.0 equiv), mesna (0.100 mmol, 16.4 mg, 20 mol%) in DMSO (5 mL). After 16 hours the reaction was worked up and purified by silica chromatography (0-12% acetone/hexanes as eluent) to afford the title compound as yellow oil (82.2 mg, 48% yield).
R$_f$= 0.12 (20% Acetone/hexanes)
^{1}H NMR (500 MHz, CDCl$_3$) δ 8.64 (d, *J* = 4.2 Hz, 1H), 7.75 (td, *J* = 7.8, 2.0 Hz, 1H), 7.61 (d, *J* = 7.8 Hz, 1H), 7.33 (dd, *J* = 7.6, 4.8 Hz, 1H), 7.30 – 7.23 (m, 2H), 7.19 (d, *J* = 7.2 Hz, 3H), 3.78 – 3.67 (m, 1H), 2.86 – 2.69 (m, 2H), 2.56 – 2.09 (m, 5H), 1.81 – 1.71 (m, 1H), 1.24 (m, 6H).
^{13}C NMR (151 MHz, CDCl$_3$) δ 155.1 (dd, *J* = 30.4, 26.0 Hz), 149.0, 141.8, 136.5, 128.5, 126.0, 124.2, 120.6 (t, *J* = 5.3 Hz), 120.5 (dd, *J* = 248.8, 244.9 Hz), 60.9 (dd, *J* = 27.6, 23.2 Hz), 57.0, 32.8, 29.6, 26.0, 23.8.
^{19}F NMR (376 MHz, CDCl$_3$) δ -103.68 (dd, *J* = 249.0, 8.3 Hz, 1F), -116.18 (dd, *J* = 249.0, 17.3 Hz, 1F).
HRMS (APCI) *m/z*: [M+H] calcd. for $C_{20}H_{26}N_3F_2$, 346.2089; found 346.2089.

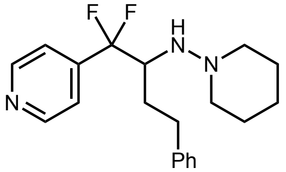

***N*-(1,1-difluoro-4-phenyl-1-(pyridin-4-yl)butan-2-yl)piperidin-1-amine (10):** Prepared according to general procedure A using (E)-3-phenyl-N-(piperidin-1-yl)propan-1-imine **S6** (0.500 mmol, 108 mg, 1.0 equiv), 4-(trifluoromethyl)pyridine (1.50 mmol, 0.173 mL, 3.0 equiv), 4CzIPN (5.00 µmol, 3.93 mg, 1 mol%), sodium formate (2.00 mmol, 136 mg, 4.0 equiv), mesna (0.100 mmol, 16.4 mg, 20 mol%) in DMSO (5 mL). After 16 hours the reaction was worked up and purified by silica chromatography (0-30% EtOAc/hexanes as eluent). This was further purified by silica prep plate (60% EtOAc/hexanes as eluent) to afford the title compound as a yellow oil (19.0 mg, 11% yield).
R$_f$= 0.67 (60% EtOAc/hexanes)

^{1}H NMR (500 MHz, CDCl$_3$) δ 8.65 (d, *J* = 4.8 Hz, 2H), 7.41 (d, *J* = 5.1 Hz, 2H), 7.29 (t, *J* = 7.9 Hz, 2H), 7.21 (t, *J* = 7.4 Hz, 1H), 7.17 (d, *J* = 8.3 Hz, 2H), 3.35 (m, 1H), 2.88 – 2.64 (m, 2H), 2.35 (m, 4H), 2.12 – 2.02 (m, 1H), 1.66 – 1.55 (m, 1H), 1.45 – 1.13 (m, 6H).
^{13}C NMR (151 MHz, CDCl$_3$) δ 149.6, 144.7 (t, *J* = 27.6 Hz), 141.1, 128.7, 128.5, 126.3, 121.4 (dd, *J* = 248.8, 245.1 Hz), 121.0 (dd, *J* = 7.1, 5.1 Hz), 62.4 (dd, *J* = 27.9, 25.9 Hz), 57.3, 32.7, 29.6, 26.0, 23.9.
^{19}F NMR (376 MHz, CDCl$_3$) δ -100.54 (d, *J* = 245.5 Hz, 1F), -113.28 (d, *J* = 246.9 Hz, 1F).
HRMS (APCI) *m/z*: [M+H] calcd. for $C_{20}H_{26}N_3F_2$, 346.2089; found 346.2097.

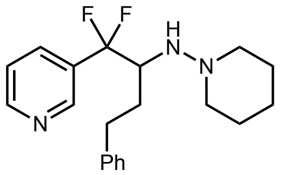

***N*-(1,1-difluoro-4-phenyl-1-(pyridin-3-yl)butan-2-yl)piperidin-1-amine (11):** Prepared according to general procedure A using (E)-3-phenyl-N-(piperidin-1-yl)propan-1-imine **S6** (0.500 mmol, 108 mg, 1.0 equiv), 3-(trifluoromethyl)pyridine (1.50 mmol, 0.173 mL, 3.0 equiv), 4CzIPN (5.00 μmol, 3.93 mg, 1 mol%), sodium formate (2.00 mmol, 136 mg, 4.0 equiv), mesna (0.100 mmol, 16.4 mg, 20 mol%) in DMSO (5 mL). After 16 hours the reaction was worked up and purified by silica chromatography (5-25% acetone/hexanes as eluent) to afford the title compound as a yellow oil (30.0 mg, 17% yield).
R_f= 0.29 (50% EtOAc/hexanes)
^{1}H NMR (500 MHz, CDCl$_3$) δ 8.76 (s, 1H), 8.64 (d, *J* = 4.9 Hz, 1H), 7.79 (dd, *J* = 8.0, 2.2 Hz, 1H), 7.32 – 7.24 (m, 3H), 7.26 – 7.10 (m, 3H), 3.47 – 3.24 (m, 1H), 2.92 – 2.63 (m, 2H), 2.57 – 2.00 (m, 5H), 1.66 – 1.54 (m, 1H), 1.43 – 1.14 (m, 6H).
^{13}C NMR (151 MHz, CDCl$_3$) δ 150.5, 148.0 (dd, *J* = 8.3, 5.5 Hz), 141.2, 134.0 (dd, *J* = 7.7, 5.0 Hz), 132.2 (t, *J* = 26.5 Hz), 128.7, 128.5, 126.3, 122.6, 121.9 (dd, *J* = 249.1, 245.2 Hz), 62.8 (dd, *J* = 29.3, 26.5 Hz), 57.3, 32.7, 29.7, 26.1, 23.8.
^{19}F NMR (376 MHz, CDCl$_3$) δ -97.72 (dd, *J* = 251.3, 7.5 Hz), -112.34 (dd, *J* = 251.1, 16.0 Hz).
HRMS (APCI) *m/z*: [M+H] calcd. for $C_{20}H_{26}N_3F_2$, 346.2089; found 346.2094.

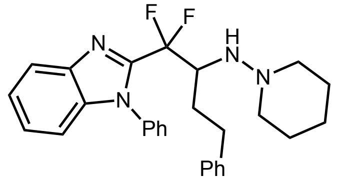

***N*-(1,1-difluoro-4-phenyl-1-(1-phenyl-1*H*-benzo[*d*]imidazol-2-yl)butan-2-yl)piperidin-1-amine (12):** Prepared according to general procedure A using (E)-3-phenyl-N-(piperidin-1-yl)propan-1-imine **S6** (0.250 mmol, 54.1 mg, 1.0 equiv), 1-phenyl-2-(trifluoromethyl)-1H-benzo[d]imidazole **S4** (0.750 mmol, 0.197 g, 3.0 equiv), 4CzIPN (2.50 μmol, 2.0 mg, 1 mol%), sodium formate (1.00 mmol, 68.0 mg, 4.0 equiv), mesna (0.05 mmol, 8.20 mg, 20 mol%), formic acid (0.750 mmol, 28.0 μL, 3.0 equiv) in DMSO (2.5 mL). After 16 hours the reaction was worked up and purified by silica chromatography (0-20% EtOAc/hexanes as eluent) followed by further purification by silica prep plate (20% EtOAc/hexanes as eluent) to afford the title compound as clear oil (44.0 mg, 38% yield).

R_f= 0.29 (20% EtOAc/hexanes)
^{1}H NMR (400 MHz, CDCl$_3$) δ 7.87 (d, J = 8.0 Hz, 1H), 7.53- 7.46 (m, 3H), 7.44 – 7.26 (m, 4H), 7.22 – 7.08 (m, 5H), 7.04 (d, J = 8.0 Hz, 1H), 3.51 – 3.39 (m, 1H), 2.83-2.61 (m, 3H), 2.38 (br s, 3H), 2.16- 2.08 (m, 1H), 1.89 – 1.77 (m, 1H), 1.40 – 1.10 (m, 6H).
^{13}C NMR (151 MHz, CDCl$_3$) δ 146.9 (t, J = 30.5 Hz), 141.6, 141.3, 137.7, 136.0, 129.6-129.0 (m), 129.3, 128.6- 127.9 (m), 128.5, 128.5, 126.0, 124.5, 123.2, 120.8, 118.6 (dd, J = 249.6, 244.1 Hz), 110.9, 61.4 (dd, J = 26.4, 22.1 Hz), 57.5, 32.5, 29.1, 26.0, 23.8.
^{19}F NMR (376 MHz, CDCl$_3$) δ -97.86 (dd, J = 269.1, 8.7 Hz, 1F), -106.91 (dd, J = 269.6, 17.2 Hz, 1F).
HRMS (APCI) *m/z*: [M+H] calcd. for $C_{28}H_{31}N_4F_2$, 461.2511; found 461.2510.

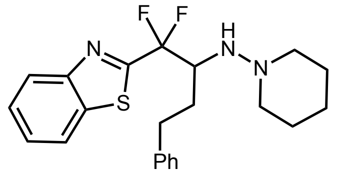

***N*-(1-(benzo[*d*]thiazol-2-yl)-1,1-difluoro-4-phenylbutan-2-yl)piperidin-1-amine (13):**
Prepared according to general procedure B using ((E)-3-phenyl-N-(piperidin-1-yl)propan-1-imine **S6** (0.100 mmol, 216 mg, 1.0 equiv), 2-(trifluoromethyl)benzo[d]thiazole **S5** (0.300 mmol, 0.0609, 3.0 equiv), 4CzIPN (1.00 μmol, 0.787 mg, 1 mol%), sodium formate (0.400 mmol, 27.2 mg, 4.0 equiv), mesna (20.0 μmol, 3.28 mg, 20 mol%), formic acid (0.300 mmol, 11 μL, 3.0 equiv) in DMSO (1 mL). After 16 hours the reaction was worked up and purified by silica chromatography (0-12% EtOAc/hexanes as eluent) followed by further purification by silica prep plate (10% Acetone/hexanes as eluent) to afford the title compound as clear oil (15.0 mg, 37% yield).
R_f= 0.63 (20% EtOAc/hexanes)
^{1}H NMR (500 MHz, CDCl$_3$) δ 8.11 (d, J = 8.3 Hz, 1H), 7.94 (d, J = 8.1 Hz, 1H), 7.57 – 7.48 (m, 1H), 7.49 – 7.41 (m, 1H), 7.31 – 7.26 (m, 2H), 7.20 (m, 3H), 3.83 – 3.72 (m, 1H), 2.92 – 2.72 (m, 2H) 2.64 – 2.14 (m, 5H), 1.87 – 1.75 (m, 1H), 1.34 – 1.01 (m, 6H).
^{13}C NMR (101 MHz, CDCl$_3$) δ 165.0 (dd, J = 34.0, 31.1 Hz), 152.8, 141.3, 135.3, 128.6, 128.6, 126.6, 126.3, 126.4, 124.2, 121.9, 119.5 (dd, J = 249.3, 246.0 Hz), 61.7 (dd, J = 26.6, 23.5 Hz), 57.2, 32.8, 29.4 (t, J = 2.5 Hz), 25.9, 23.8.
^{19}F NMR (376 MHz, CDCl$_3$) δ -95.08 (dd, J = 259.7, 7.6 Hz, 1F), -107.07 (dd, J = 259.7, 16.0 Hz, 1F).
HRMS (APCI) *m/z*: [M+H] calcd. for $C_{22}H_{26}N_3F_2S$, 402.1810; found 402.1812.

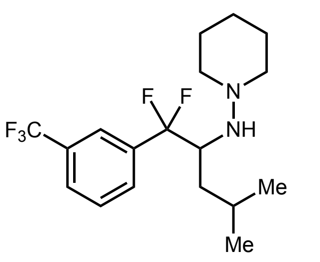

***N*-(1,1-difluoro-4-methyl-1-(3-(trifluoromethyl)phenyl)pentan-2-yl)piperidin-1-amine (14):**
Prepared according to general procedure A using (*E*)-3-methyl-*N*-(piperidin-1-yl)butan-1-imine **S7** (0.500 mmol, 97 μL, 1.0 equiv), 1,3-bis(trifluoromethyl)benzene (1.50 mmol, 0.232 mL, 3.0

equiv), 4CzIPN (5.00 μmol, 3.93 mg, 1 mol%), sodium formate (2.00 mmol, 136 mg, 4.0 equiv), mesna (0.100 mmol, 16.4 mg, 20 mol%) in DMSO (5 mL). After 16 hours the reaction was worked up and purified by silica chromatography (0-20% EtOAc/hexanes as eluent) to afford the title compound as yellow oil (112 mg, 62% yield).
R_f= 0.71 (20% EtOAc/hexanes)
^{1}H NMR (500 MHz, $CDCl_3$) δ 7.79 (s, 1H), 7.70 (d, *J* = 7.9 Hz, 1H), 7.66 (d, *J* = 7.8 Hz, 1H), 7.51 (t, *J* = 7.8 Hz, 1H), 3.39 – 3.27 (m, 1H), 2.59 – 1.89 (m, 4H), 1.67 (m, 1H), 1.50 (m, 1H), 1.35 – 1.14 (m, 7H), 0.96 (t, *J* = 6.9 Hz, 6H).
^{13}C NMR (151 MHz, $CDCl_3$) δ 137.9 (t, *J* = 26.8 Hz), 130.2 (q, *J* = 32.6 Hz), 129.7 (t, *J* = 6.4 Hz), 128.2, 126.0, 124.2 (q, *J* = 272.2 Hz), 127.1 – 121.4 (m), 122.1 (dd, J= 248.2, 244.2 Hz), 61.2 (dd, *J* = 29.8, 26.6 Hz), 57.1, 37.1, 26.1, 24.7, 24.0, 23.8, 22.0.
^{19}F NMR (376 MHz, $CDCl_3$) δ -62.69 (s, 3F), -96.89 (dd, *J* = 246.0, 6.8 Hz, 1F), -113.69 (dd, *J* = 245.5, 16.3 Hz, 1F).
HRMS (APCI) *m/z*: [M+H] calcd. for $C_{18}H_{26}N_2F_5$, 365.2011; found 365.2011.

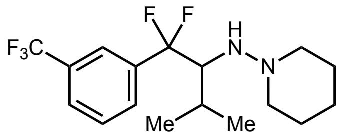

***N*-(1,1-difluoro-3-methyl-1-(3-(trifluoromethyl)phenyl)butan-2-yl)piperidin-1-amine (15):** Prepared according to general procedure A using (E)-2-methyl-N-(piperidin-1-yl)propan-1-imine **S8** (0.500 mmol, 88 μL, 1.0 equiv), 1,3-bis(trifluoromethyl)benzene (1.50 mmol, 0.232 mL, 3.0 equiv), 4CzIPN (5.00 μmol, 3.93 mg, 1 mol%), sodium formate (2.00 mmol, 140 mg, 4.0 equiv), mesna (0.100 mmol, 16.4 mg, 20 mol%) in DMSO (5 mL). After 16 hours the reaction was worked up and purified by silica chromatography (0-10% EtOAc/hexanes as eluent) to afford the title compound as clear oil (72.3 mg, 41% yield).
R_f= 0.52 (10% EtOAc/hexanes)
^{1}H NMR (500 MHz, $CDCl_3$) δ 7.78 (s, 1H), 7.70 (d, *J* = 7.8 Hz, 1H), 7.65 (d, *J* = 7.8 Hz, 1H), 7.50 (t, *J* = 7.8 Hz, 1H), 3.19 – 3.10 (m, 1H), 2.68 – 1.85 (m, 5H), 1.37 – 1.13 (m, 6H), 1.09 (d, *J* = 7.0 Hz, 3H), 0.95 (d, *J* = 6.8 Hz, 3H).
^{13}C NMR (151 MHz, $CDCl_3$) δ 138.6 (qt, *J* = 26.7, 7.3 Hz), 130.3 (q, *J* = 32.5 Hz), 129.5 (t, *J* = 6.6 Hz), 128.3, 126.0, 124.2 (q, *J* = 272.3 Hz), 127.3 – 120.9 (m), 122.8 (dd, *J* = 252.1, 244.9 Hz), 68.0 – 66.7 (m), 56.9, 27.1, 26.1, 23.8, 21.7, 17.8.
^{19}F NMR (376 MHz, $CDCl_3$) δ -62.68 (s, 3F), -96.27 (dd, *J* = 245.5, 8.3 Hz, 1F), -110.95 (dd, *J* = 245.5, 20.8 Hz, 1F).
HRMS (APCI) *m/z*: [M+H] calcd. for $C_{17}H_{24}N_2F_5$, 351.1854; found 351.1862.

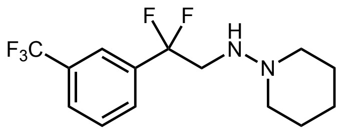

***N*-(2,2-difluoro-2-(3-(trifluoromethyl)phenyl)ethyl)piperidin-1-amine (16):** Prepared according to a slighty modified version of general procedure B using *N*-(piperidin-1-yl)methanimine **S9** (0.300 mmol, 34.7 μL, 1.0 equiv), 1,3-Bis(trifluoromethyl)benzene (0.100 mmol, 16.0 μL, 3.0 equiv), 4CzIPN (1.00 μmol, 0.787 mg, 1 mol%), sodium formate (0.800 mmol, 54.4 mg, 4.0 equiv), mesna (20.0 μmol, 3.28 mg, 20 mol%), formic acid (0.300 mmol,

11.0 µL, 3.0 equiv) in DMSO (1 mL). After 16 hours the reaction was worked up and purified by silica chromatography (5-50% EtOAc/hexanes as eluent) to afford the title compound as a yellow oil (8.2 mg, 27% yield). *^{13}C NMR was taken of the HCl salt of* ***16***
R_f=0.56 (40% EtOAc/hexanes)
^{1}H NMR (600 MHz, $CDCl_3$) δ 7.82 (s, 1H), 7.73 (d, *J* = 7.9 Hz, 1H), 7.69 (d, *J* = 7.8 Hz, 1H), 7.54 (t, *J* = 7.8 Hz, 1H), 3.45 (t, *J* = 13.5 Hz, 2H), 2.75 – 2.10 (m, 4H), 1.47 (p, *J* = 5.6 Hz, 4H), 1.37 – 1.11 (m, 2H).
^{13}C NMR (101 MHz, MeOD) δ 137.4 (t, J= 26.17), 132.1 (q, J= 32.86 Hz), 130.9, 130.7 (t, J=6.26 Hz), 128.6- 128.4 (m), 125.3 (q, J= 272.82), 123.9-123.2 (m), 120.8 (t, J=243.80 Hz), 56.7, 52.1 (t, J=31.4), 24.0, 22.3.
^{19}F NMR (376 MHz, $CDCl_3$) δ -62.78(s, 3F), -99.69 (t, *J* = 13.5 Hz, 2F).
HRMS (APCI) *m/z*: [M+H] calcd. for $C_{14}H_{18}N_2F_5$, 309.1385; found 309.1391

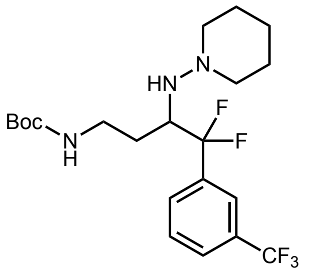

Tert-butyl (4,4-difluoro-3-(piperidin-1-ylamino)-4-(3-(trifluoromethyl)phenyl)butyl) carbamate (17): Prepared according to general procedure A *tert*-butyl (*E*)-(3-(piperidin-1-ylimino)propyl)carbamate **S10** (0.500 mmol, 128 mg, 1.0 equiv), 1,3-bis(trifluoromethyl)benzene (1.50 mmol, 0.232 mL, 3.0 equiv), 4CzIPN (5.00 µmol, 3.93 mg, 1 mol%), sodium formate (2.00 mmol, 140 mg, 4.0 equiv), mesna (0.100 mmol, 16.4 mg, 20 mol%) in DMSO (5 mL). After 16 hours the reaction was worked up and purified by silica chromatography (3-20% EtOAc/hexanes as eluent) to afford the title compound as clear oil (134 mg, 59% yield).
R_f=0.59 (30% EtOAc/hexanes)
^{1}H NMR (500 MHz, $CDCl_3$) δ 7.78 (s, 1H), 7.70 (d, *J* = 7.8 Hz, 2H), 7.55 (t, *J* = 7.9 Hz, 1H), 5.66 (br s, 1H), 3.43 – 3.30 (m, 1H), 3.21 (m, 2H), 2.42 (br s, 4H), 1.92 (m, 1H), 1.74 – 1.39 (m, 16H).
^{13}C NMR (101 MHz, $CDCl_3$) δ 156.1 136.79 (t, J= 25.61 Hz), 130.8 (q, J=32.88 Hz), 129.5 (t, J= 6.22 Hz), 128.8, 126.8, 124.0 (q, J= 273.06 Hz), 123.3 (m), 121.8 (dd, J= 246.03, 247.48 Hz), 79.2, 61.3 (t, J= 27.64 Hz), 57.2, 38.3, 29.5, 28.6, 26.0, 23.8.
^{19}F NMR (376 MHz, $CDCl_3$) δ -62.73 (s, 3F), -100.50 (dd, *J* = 249.0, 8.0 Hz, 1F), -108.28 (dd, *J* = 249.5, 14.0 Hz, 1F).
HRMS (APCI) *m/z*: [M+H] calcd. for $C_{21}H_{31}O_2N_3F_5$, 452.2331; found 452.2328

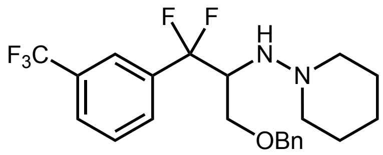

***N*-(3-(benzyloxy)-1,1-difluoro-1-(3-(trifluoromethyl)phenyl)propan-2-yl)piperidin-1-amine (18):** Prepared according to general procedure A (*E*)-2-(benzyloxy)-*N*-(piperidin-1-yl)ethan-1-imine **S11** (0.500 mmol, 116 mg, 1.0 equiv), 1,3-Bis(trifluoromethyl)benzene (1.50 mmol, 0.232 mL, 3.0 equiv), 4CzIPN (5.00 μmol, 3.93 mg, 1 mol%), sodium formate (2.00 mmol, 136 mg, 4.0 equiv), mesna (0.100 mmol, 16.4 mg, 20 mol%) in DMSO (5 mL). After 16 hours the reaction was worked up and purified by silica chromatography (0-12% EtOAc/hexanes as eluent) to afford the title compound as clear oil (130 mg, 61% yield).
R_f= 0.64 (20% EtOAc/hexanes)
^{1}H NMR (500 MHz, $CDCl_3$) δ 7.78 (s, 1H), 7.68 (dd, *J* = 13.9, 7.8 Hz, 2H), 7.51 (t, *J* = 7.8 Hz, 1H), 7.38 – 7.32 (m, 2H), 7.32 – 7.26 (m, 3H), 4.58 – 4.47 (dd, J= 15.24, 11.87 Hz, 2H), 3.86 (dd, *J* = 10.0, 3.4 Hz, 1H), 3.68 – 3.57 (m, 1H), 3.45 (t, *J* = 9.4 Hz, 1H), 2.73 – 1.80 (m, 4H), 1.37 – 1.07 (m, 6H).
^{13}C NMR (151 MHz, $CDCl_3$) δ 137.8, 137.7 – 137.2 (m), 130.3 (q, *J* = 32.6 Hz), 129.5 (t, *J* = 6.7 Hz), 128.6, 128.3, 127.9, 127.7, 129.5 (m), 124.0 (q, J= 271.92 Hz), 137.7 – 137.2 (m), 121.4 (dd, *J* = 249.9, 244.4 Hz), 73.5, 66.7 (dd, *J* = 5.3, 3.0 Hz), 63.6 (dd, *J* = 30.3, 24.8 Hz), 56.9, 26.0, 23.8.
^{19}F NMR (376 MHz, $CDCl_3$) δ -62.71 (s, 3F), -96.86 (dd, *J* = 247.2, 5.9 Hz), -113.28 (dd, *J* = 247.2, 18.6 Hz).
HRMS (APCI) *m/z*: [M+H] calcd. for $C_{22}H_{26}ON_2F_5$, 429.1960; found 429.1972.

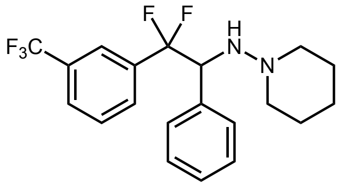

***N*-(2,2-difluoro-1-phenyl-2-(3-(trifluoromethyl)phenyl)ethyl)piperidin-1-amine (19):** Prepared according to general procedure A using (*E*)-1-phenyl-*N*-(piperidin-1-yl)methanimine **S12** (0.500 mmol, 94.1 mg, 1.0 equiv), 1,3-bis(trifluoromethyl)benzene (1.50 mmol, 0.233 mL, 3.0 equiv), 4CzIPN (5.00 μmol, 3.93 mg, 1 mol%), sodium formate (2.00 mmol, 136 mg, 4.0 equiv), mesna (0.100 mmol, 16.4 mg, 20 mol%) in DMSO (5 mL). After 16 hours the reaction was worked up and purified by silica chromatography (0-10% EtOAc/hexanes as eluent) to afford the title compound as yellow oil (128 mg, 67% yield).
R_f= 0.44 (10% EtOAc/ hexanes)
^{1}H NMR (500 MHz, $CDCl_3$) δ 7.64 (d, *J* = 7.7 Hz, 1H), 7.59 (s, 1H), 7.51 (d, *J* = 7.9 Hz, 1H), 7.45 (t, *J* = 7.8 Hz, 1H), 7.35 – 7.25 (m, 3H), 7.25 – 7.19 (m, 2H), 4.43 (dd, *J* = 14.4, 8.8 Hz, 1H), 2.73 – 2.16 (m, 4H), 1.55 – 1.01 (m, 6H).
^{13}C NMR (151 MHz, $CDCl_3$) δ 137.2-136.8 (m), 136.8, 130.2 (q, 32.71 Hz), 129.9-129.8 (m), 129.0, 128.4, 128.2, 128.2, 124.0 (q, *J* = 272.3 Hz), 126.3-126.2 (m), 123.8 (tq, *J* = 7.7, 3.9 Hz), 121.2 (dd, *J* = 249.9, 247.7 Hz), 68.4 (dd, *J* = 28.7, 26.0 Hz), 57.6, 26.0, 23.8.
^{19}F NMR (376 MHz, $CDCl_3$) δ -62.75(s, 3F), -98.66 (dd, *J* = 247.3, 8.7 Hz, 1F), -106.76 (dd, *J* = 247.3, 14.2 Hz, 1F).

HRMS (APCI) *m/z*: [M+H] calcd. for $C_{20}H_{22}N_2F_5$, 385.1698; found 385.1692.

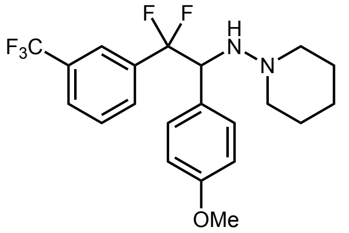

***N*-(2,2-difluoro-1-(4-methoxyphenyl)-2-(3-(trifluoromethyl)phenyl)ethyl)piperidin-1-amine (20):** Prepared according to general procedure A using (*E*)-1-(4-methoxyphenyl)-*N*-(piperidin-1-yl)methanimine **S13** (0.500 mmol, 94.1 mg, 1.0 equiv), 1,3-bis(trifluoromethyl)benzene (1.50 mmol, 0.233 mL, 3.0 equiv), 4CzIPN (5.00 μmol, 3.93 mg, 1 mol%), sodium formate (2.00 mmol, 140 mg, 4.0 equiv), mesna (0.100 mmol, 16.4 mg, 20 mol%) in DMSO (5 mL). After 16 hours the reaction was worked up and purified by silica chromatography (0-10% EtOAc/hexanes as eluent). Some of the product co-eluted with a byproduct, this was further purified by a silica gel prep plate (20% EtOAc/hexanes) to afford the title compound as yellow oil (111 mg, 54% yield).
R_f= 0.60 (20% EtOAc/hexanes)
^{1}H NMR (500 MHz, $CDCl_3$) δ 7.64 (d, *J* = 7.7 Hz, 1H), 7.60 (s, 1H), 7.50 (d, *J* = 7.9 Hz, 1H), 7.45 (t, *J* = 7.6 Hz, 1H), 7.13 (d, *J* = 8.6 Hz, 2H), 6.85 – 6.78 (m, 2H), 4.38 (dd, *J* = 14.3, 8.7 Hz, 1H), 3.79 (s, 3H), 2.63 – 2.17 (m, 4H), 1.45 – 1.16 (m, 6H).
^{13}C NMR (151 MHz, $CDCl_3$) δ 159.7, 137.1 (t, *J* = 26.9 Hz), 130.1 (q, 32.6 Hz), 130.1, 129.8 (t, *J* = 6.4 Hz), 128.8, 128.2, 128.2, 126.2 (q, *J* = 4.0 Hz), 124.1 (q, *J* = 272.6 Hz), 121.3 (dd, J=247.69, 249.24 Hz), 113.7, 67.6 (dd, *J* = 28.7, 26.0 Hz), 57.6, 55.3, 26.1, 23.9.
^{19}F NMR (376 MHz, $CDCl_3$) δ -62.72 (s, 3F), -98.57 (dd, *J* = 246.6, 9.4 Hz, 1F), -107.00 (dd, *J* = 246.6, 14.2 Hz, 1F).
HRMS (APCI) *m/z*: [M+H] calcd. for $C_{21}H_{24}ON_2F_5$, 415.1803; found 415.1806.

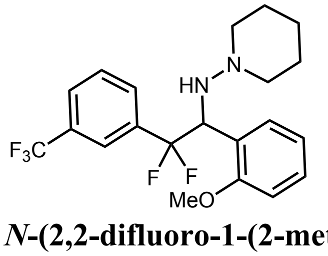

***N*-(2,2-difluoro-1-(2-methoxyphenyl)-2-(3-(trifluoromethyl)phenyl)ethyl)piperidin-1-amine (21):** Prepared according to general procedure B using (*E*)-1-(2-methoxyphenyl)-*N*-(piperidin-1-yl)methanimine **S14** (0.100 mmol, 21.8 mg, 1.0 equiv), 1,3-Bis(trifluoromethyl)benzene (0.300 mmol, 47 μL, 3.0 equiv), 4CzIPN (1.00 μmol, 0.787 mg, 1 mol%), sodium formate (0.400 mmol, 27.2 mg, 4.0 equiv), mesna (20.0 μmol, 3.28 mg, 20 mol%), formic acid (0.300 mmol, 11.0 μL, 3.0 equiv) in DMSO (1 mL). After 16 hours the reaction was worked up and purified by silica chromatography (0-12% EtOAc/hexanes as eluent) to afford the title compound as a clear oil (25.8 mg, 62% yield).
R_f= 0.54 (20% EtOAc/hexanes)
^{1}H NMR (500 MHz, $CCCl_3$) δ 7.64 (s, 1H), 7.61 (d, *J* = 7.8 Hz, 1H), 7.50 (d, *J* = 7.8 Hz, 1H), 7.44 – 7.35 (m, 2H), 7.28 – 7.21 (m, 1H), 6.94 (t, *J* = 7.5 Hz, 1H), 5.02 (t, *J* = 12.9 Hz, 1H), 2.83 – 2.10 (m, 4H), 1.40 (m, 4H), 1.29 – 1.18 (m, 2H).

^{13}C NMR (151 MHz, CDCl$_3$) δ 157.1, 137.7 (t, J = 26.8 Hz), 130.0 (q, J = 32.6 Hz), 137.7 (t, J = 26.8 Hz), 129.4, 129.3, 128.0, 126.0 (m), 125.6 (m), 124.1 (q, J = 272.1 Hz), 123.6 (td, J = 6.5, 3.3 Hz), 121.3 (dd, J=248.13, 250.20 Hz), 120.5, 110.5, 60.1 (t, J = 26.0 Hz), 57.6, 55.3, 26.0, 23.7.
^{19}F NMR (376 MHz, CDCl$_3$) δ -62.67 (s, 3F), -102.47 (d, J = 244.5 Hz, 1F), -106.44 (dd, J = 242.6, 13.4 Hz, 1F).
HRMS (APCI) *m/z*: [M+H] calcd. for $C_{21}H_{24}ON_2F_5$, 415.1803; found 415.1813.

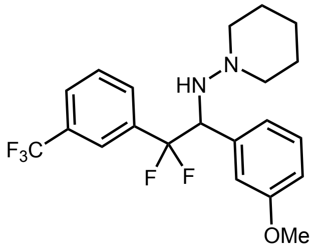

***N*-(2,2-difluoro-1-(3-methoxyphenyl)-2-(3-(trifluoromethyl)phenyl)ethyl)piperidin-1-amine (22):** Prepared according to general procedure B using (*E*)-1-(4-methoxyphenyl)-*N*-(piperidin-1-yl)methanimine **S15** (0.500 mmol, 94.1 mg, 1.0 equiv), 1,3-Bis(trifluoromethyl)benzene (1.50 mmol, 0.233 mL, 3.0 equiv), 4CzIPN (5.00 μmol, 3.93 mg, 1 mol%), sodium formate (2.00 mmol, 136 mg, 4.0 equiv), mesna (0.100 mmol, 16.4 mg, 20 mol%), formic acid (1.50 mmol, 56.0 μL, 3.0 equiv) in DMSO (5 mL). After 16 hours the reaction was worked up and purified by silica chromatography (0-12% EtOAc/hexanes as eluent) to afford the title compound as yellow oil (88.2 mg, 43% yield).
R$_f$= 0.60 (20% EtOAc/hexanes)
^{1}H NMR (400 MHz, CDCl$_3$) δ 7.64 (d, J = 7.7 Hz, 1H), 7.60 (s, 1H), 7.52 (d, J = 7.9 Hz, 1H), 7.45 (t, J = 7.7 Hz, 1H), 7.19 (t, J = 7.9 Hz, 1H), 6.88 – 6.74 (m, 3H), 4.40 (dd, J = 14.3, 8.8 Hz, 1H), 2.42 (m, 4H), 1.57 – 1.00 (m, 6H).
^{13}C NMR (151 MHz, CDCl$_3$) δ 159.5, 138.3 (t, J = 2.0 Hz), 137.0 (t, J = 26.8 Hz), 130.2 (q, J = 32.8 Hz), 129.8 (t, J = 6.2 Hz), 129.2, 128.2, 128.1, 126.3 (m), 124.0 (q, J= 273.1 Hz), 123.9 (m), 121.2 (dd, J= 247.8, 250.2), 114.5, 114.1, 68.4 (dd, J = 28.4, 26.3 Hz), 57.6, 55.3, 26.0, 23.8.
^{19}F NMR (376 MHz, CDCl$_3$) δ -62.74 (s, 3F), -98.81 (dd, J = 247.2, 8.8 Hz, 1F), -106.56 (dd, J = 247.2, 14.4 Hz, 1F).
HRMS (APCI) *m/z*: [M+H] calcd. for $C_{21}H_{24}ON_2F_5$, 415.1803; found 415.1804.

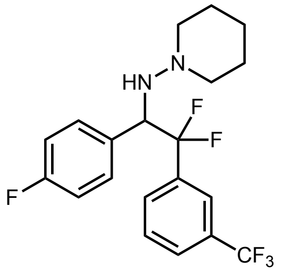

***N*-(2,2-difluoro-1-(4-fluorophenyl)-2-(3-(trifluoromethyl)phenyl)ethyl)piperidin-1-amine (23):** Prepared according to general procedure B using (*E*)-1-(4-fluorophenyl)-*N*-(piperidin-1-

yl)methanimine **S16** (0.100 mmol, 206 mg, 1.0 equiv), 1,3-Bis(trifluoromethyl)benzene (0.300 mmol, 47 µL, 3.0 equiv), 4CzIPN (1.00 µmol, 0.787 mg, 1 mol%), sodium formate (0.400 mmol, 27.2 mg, 4.0 equiv), mesna (20.0 µmol, 3.28 mg, 20 mol%), formic acid (0.300 mmol, 11.0 µL, 3.0 equiv) in DMSO (1 mL). After 16 hours the reaction was worked up and purified by silica chromatography (0-12% EtOAc/hexanes as eluent) to afford the title compound as a clear oil (31.1 mg, 77% yield).

R_f= 0.72 (20% EtOAc/hexanes)

^{1}H NMR (600 MHz, CDCl$_3$) δ 7.65 (m, 1H), 7.57 (s, 1H), 7.46 (m, 2H), 7.22 – 7.16 (m, 2H), 6.97 (t, *J* = 8.7 Hz, 2H), 4.43 (dd, *J* = 13.8, 9.1 Hz, 1H), 2.51 (m, 4H), 1.49 – 1.19 (m, 6H).

^{13}C NMR (151 MHz, CDCl$_3$) δ 162.8 (d, *J* = 247.1 Hz), 136.7 (t, *J* = 26.8 Hz), 132.5, 130.7 (d, *J* = 7.7 Hz), 130.3 (q, *J* = 32.6 Hz), 129.7 (t, *J* = 6.1 Hz), 128.3, 126.5 (m), 124.0 (q, *J* = 272.0 Hz), 123.7 (tq, *J* = 7.6, 4.1 Hz), 121.1 (dd, *J* = 248.77 Hz, 248.53), 115.1 (d, *J* = 21.6 Hz), 67.5 (t, *J* = 27.4 Hz), 57.5, 25.9. 23.8.

^{19}F NMR (376 MHz, CDCl$_3$) δ -62.77 (s, 3F), -99.35 (dd, *J* = 247.8, 8.8 Hz, 1F), -106.41 (dd, *J* = 247.8, 14.0 Hz, 1F), -113.89 (td, *J* = 8.7, 4.2 Hz, 1F).

HRMS (APCI) *m/z*: [M+H] calcd. for $C_{20}H_{21}N_2F_6$, 403.1603; found 403.1614.

.

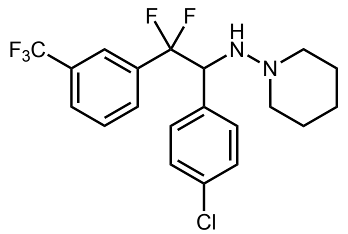

***N*-(1-(4-chlorophenyl)-2,2-difluoro-2-(3-(trifluoromethyl)phenyl)ethyl)piperidin-1-amine (24):** Prepared according to general procedure B using (*E*)-1-(4-chlorophenyl)-*N*-(piperidin-1-yl)methanimine **S17** (0.500 mmol, 111 mg, 1.0 equiv), 1,3- bis(trifluoromethyl)benzene (1.50 mmol, 0.233 mL, 3.0 equiv), 4CzIPN (5.00 µmol, 3.90 mg, 1 mol%), sodium formate (2.00 mmol, 0.136 g, 4.0 equiv), mesna (0.100 mmol, 16.4 mg, 20 mol%), formic acid (1.50 mmol, 56.0 µL, 3.0 equiv) in DMSO (5 mL). After 16 hours the reaction was worked up and purified by silica chromatography (3-10% EtOAc/hexanes as eluent) to afford the title compound as a yellow oil (111 mg, 53% yield).

R_f=0.46 (20% EtOAc/hexanes)

^{1}H NMR (500 MHz, CDCl$_3$) δ 7.69 – 7.61 (m, 1H), 7.59 (s, 1H), 7.45 (dt, *J* = 4.6, 1.3 Hz, 2H), 7.25 (d, *J* = 7.8 Hz, 2H), 7.16 (d, *J* = 8.1 Hz, 2H), 4.43 (dd, *J* = 13.9, 9.0 Hz, 1H), 2.60 (br s, 1H), 2.41 (br s, 3H), 1.36 (m, 4H), 1.28 – 1.17 (m, 2H).

^{13}C NMR (101 MHz, CDCl$_3$) δ 136.8 (t, J= 26.75 Hz), 135.2 (t, *J* = 2.0 Hz), 134.3, 130.5, 130.4 (q, J= 32.72 Hz), 129.7 (t, J= 5.96 Hz), 128.4, 128.4, 126.5, 124.0 (q, J= 271.09 Hz), 123.7 (m), 120.9 (dd, J= 248.52, 249.18 Hz), 67.6 (dd, *J* = 28.2, 26.4 Hz), 57.5, 25.9, 23.8.

^{19}F NMR (376 MHz, CDCl$_3$) δ -62.78 (s, 3F), -99.24 (dd, *J* = 248.5, 9.2 Hz, 1F), -106.25 (dd, *J* = 248.3, 13.9 Hz, 1F).

HRMS (APCI) *m/z*: [M+H] calcd. for $C_{20}H_{21}N_2ClF_5$, 419.1308; found 419.1305.

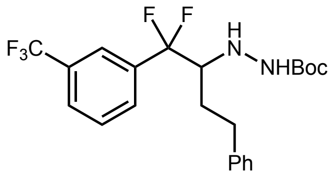

***tert*-butyl 2-(1,1-difluoro-4-phenyl-1-(3-(trifluoromethyl)phenyl)butan-2-yl)hydrazine-1-carboxylate (25):** Prepared according to general procedure A using *tert*-butyl (*E*)-2-(3-phenylpropylidene)hydrazine-1-carboxylate **S18** (0.500 mmol, 124 mg, 1.0 equiv), 1,3-Bis(trifluoromethyl)benzene (1.50 mmol, 0.232 mL, 3.0 equiv), 4CzIPN (5.00 μmol, 3.93 mg, 1 mol%), sodium formate (2.00 mmol, 136 mg, 4.0 equiv), mesna (0.100 mmol, 16.4 mg, 20 mol%) in DMSO (5 mL). After 16 hours the reaction was worked up and purified by silica chromatography (5-12% EtOAc/hexanes as eluent) to afford the title compound as yellow oil (95.5 mg, 43% yield).
R_f= 0.52 (20% EtOAc/hexanes)
^{1}H NMR (400 MHz, $CDCl_3$) δ 7.76 (s, 1H), 7.73 (d, *J* = 7.9 Hz, 1H), 7.67 (d, *J* = 7.9 Hz, 1H), 7.55 (t, *J* = 7.8 Hz, 1H), 7.29 – 7.23 (m, 2H), 7.22 - 7.13 (m, 3H), 6.09 (s, 1H), 4.11 (s, 1H), 3.45 – 3.31 (m, 1H), 3.03 – 2.93 (m, 1H), 2.89 – 2.76 (m, 1H), 1.86 – 1.75 (m, 1H), 1.65 – 1.55 (m, 1H), 1.51 (s, 9H).
^{13}C NMR (151 MHz, $CDCl_3$) δ 156.5, 141.2, 136.2 (t, *J* = 26.7 Hz), 131.0 (q, *J* = 32.8 Hz), 129.5 (t, *J* = 6.1 Hz), 129.0, 128.6, 128.5, 127.3 – 126.6 (m), 126.2, 123.8 (q, J= 272.80), 123.3 – 123.0 (m), 122.6 (t, J= 246.80 Hz), 80.9, 65.2 (t, *J* = 25.8 Hz), 32.1, 29.4, 28.4.
^{19}F NMR (376 MHz, $CDCl_3$) δ -62.65 (s, 3F), -99.65 – -108.98 (m, 2F).
HRMS (APCI) *m/z*: [M+Na] calcd. for $C_{22}H_{25}O_2N_2F_5Na$, 467.1728; found 467.1727.

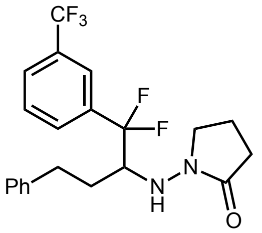

1-((1,1-difluoro-4-phenyl-1-(3-(trifluoromethyl)phenyl)butan-2-yl)amino)pyrrolidin-2-one (26): Prepared according to general procedure A using (*E*)-1-((3-phenylpropylidene)amino)pyrrolidin-2-one **S19** (0.500 mmol, 108 mg, 1.0 equiv), 1,3-Bis(trifluoromethyl)benzene (1.50 mmol, 0.232 mL, 3.0 equiv), 4CzIPN (5.00 μmol, 3.93 mg, 1 mol%), sodium formate (2.00 mmol, 136 mg, 4.0 equiv), mesna (0.100 mmol, 16.4 mg, 20 mol%) in DMSO (5 mL). After 16 hours the reaction was worked up and purified by silica chromatography (30-100% EtOAc/hexanes as eluent) to afford the title compound as a clear oil (50.0 mg, 24% yield).
R_f= 0.48 (75% EtOAc/hexanes)
^{1}H NMR (500 MHz, $CDCl_3$) δ 7.79 (s, 1H), 7.73 – 7.66 (m, 1H), 7.54 (t, *J* = 7.8 Hz, 1H), 7.28 – 7.25 (m, 2H), 7.19 (t, *J* = 7.3 Hz, 1H), 7.15 (d, *J* = 6.6 Hz, 1H), 4.64 (d, *J* = 4.6 Hz, 1H), 3.48 – 3.37 (m, 1H), 3.36 – 3.27 (m, 1H), 3.26 – 3.19 (m, 1H), 3.00 – 2.90 (m, 1H), 2.90 – 2.80 (m, 1H), 2.40 – 2.31 (m, 1H), 2.30 – 2.21 (m, 1H), 2.04 – 1.94 (m, 1H), 1.93 – 1.79 (m, 2H), 1.66 – 1.56 (m, 1H).
^{13}C NMR (101 MHz, $CDCl_3$) δ 173.6, 141.1, 136.3 (t, *J* = 26.9 Hz), 130.9 (q, *J* = 32.8 Hz), 129.6 (t, *J* = 6.3 Hz), 128.9, 128.6, 128.6, 127.1 – 126.6 (m), 126.3, 123.9 (q, J= 272.69 Hz),

123.5 – 123.2 (m), 121.6 (t, J=247.47 Hz), 63.2 (dd, J = 28.1, 26.9 Hz), 48.4 (t, J = 1.7 Hz), 32.1, 30.3 (t, J = 2.7 Hz), 29.1, 16.3.
^{19}F NMR (376 MHz, $CDCl_3$) δ -62.73 (s, 3F), -99.54 (dd, J = 253.9, 9.7 Hz, 1F), -106.46 (dd, J = 253.7, 12.7 Hz, 1F).
HRMS (APCI) *m/z*: [M+H] calcd. for $C_{21}H_{22}ON_2F_5$, 413.1647; found 413.1650.

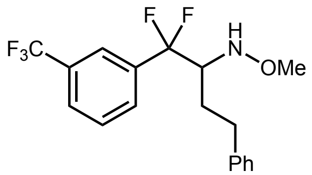

***N*-(1,1-difluoro-4-phenyl-1-(3-(trifluoromethyl)phenyl)butan-2-yl)-*O*-methylhydroxylamine (27):** Prepared according to general procedure A 3-phenylpropanal *O*-methyl oxime **S20** (0.500 mmol, 81.6 mg, 1.0 equiv), 1,3-bis(trifluoromethyl)benzene (1.50 mmol, 0.232 mL, 3.0 equiv), 4CzIPN (5.00 µmol, 3.93 mg, 1 mol%), sodium formate (2.00 mmol, 136 mg, 4.0 equiv), mesna (0.100 mmol, 16.4 mg, 20 mol%) in DMSO (5 mL). After 16 hours the reaction was worked up and purified by silica chromatography (2-12% EtOAc/hexanes as eluent) to afford the title compound as clear oil (22 mg, 12% yield).
R_f=0.68 (20% EtOAc/hexanes)
^{1}H NMR (500 MHz, $CDCl_3$) δ 7.77 (s, 1H), 7.70 (d, J = 7.8 Hz, 1H), 7.67 (d, J = 7.9 Hz, 1H), 7.55 (t, J = 7.8 Hz, 1H), 7.29 (t, J = 7.4 Hz, 2H), 7.21 (t, J = 7.4 Hz, 1H), 7.16 (d, J = 6.8 Hz, 2H), 5.62 (s, 1H), 3.39 (s, 3H), 3.37 – 3.28 (m, 1H), 2.93 – 2.83 (m, 1H), 2.78 – 2.68 (m, 1H), 2.03 – 1.92 (m, 1H), 1.86 – 1.73 (m, 1H).
^{13}C NMR (101 MHz, $CDCl_3$) δ 141.1, 136.8 (t, J = 26.9 Hz), 130.9 (q, J = 32.8 Hz), 129.4 (t, J = 6.2 Hz), 128.9, 128.7, 128.5, 126.9 – 126.7 (m), 126.4, 123.9 (q, J= 271.5 Hz), 123.3 – 123.0 (m), 121.9 (dd, J= 248.50, 249.3 Hz), 65.2 (dd, J = 27.7, 26.4 Hz), 62.2, 32.5, 27.8 (t, J = 2.6 Hz).
^{19}F NMR (376 MHz, $CDCl_3$) δ -62.72 (s, 3F), -100.81 (dd, J = 252.1, 11.1 Hz, 1F), -104.63 (dd, J = 252.3, 13.7 Hz, 1F).
HRMS (APCI) *m/z*: [M+H] calcd. for $C_{18}H_{19}ONF_5$, 360.1381; found 360.1366.

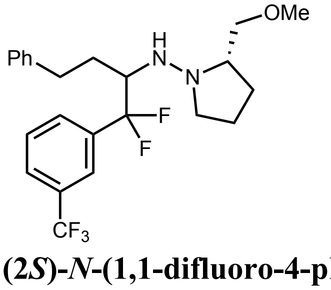

(2*S*)-*N*-(1,1-difluoro-4-phenyl-1-(3-(trifluoromethyl)phenyl)butan-2-yl)-2-(methoxymethyl)pyrrolidin-1-amine (28): Prepared according to general procedure A using (*S*,*E*)-*N*-(2-(methoxymethyl)pyrrolidin-1-yl)-3-phenylpropan-1-imine **S21** (0.100 mmol, 246 mg, 1.0 equiv), 1,3-Bis(trifluoromethyl)benzene (1.50 mmol, 47.0 µL, 3.0 equiv), 4CzIPN (1.00 µmol, 0.788 mg, 1 mol%), sodium formate (0.400 mmol, 27.2 mg, 4.0 equiv), mesna (0.020 mmol, 3.28 mg, 20 mol%) in DMSO (1 mL). Crude ^{19}F NMR analysis shows a 1.0 : 1.9 d.r.

After 16 hours the reaction was worked up and purified by silica chromatography (3-20% EtOAc/hexanes as eluent) to afford the title compound as a yellow oil (10.0 mg, 23% yield, diastereomer 1 and 18.6 mg, 42% yield, diastereomer 2).

Diastereomer 1:

R_f= 0.56 (30% EtOAc/hexanes)

^{1}H NMR (500 MHz, $CDCl_3$) δ 7.82 (s, 1H), 7.70 (t, *J* = 7.9 Hz, 2H), 7.53 (t, *J* = 7.8 Hz, 1H), 7.30 – 7.20 (m, 2H), 7.17 (t, *J* = 8.0 Hz, 1H), 7.12 (d, *J* = 8.2 Hz, 2H), 3.52 – 3.41 (m, 2H), 3.35 – 3.30 (m, 1H), 3.30 (s, 3H), 3.18 – 3.01 (m, 1H), 2.83 – 2.66 (m, 2H), 2.64 – 2.58 (m, 1H), 2.18 (q, *J* = 8.5 Hz, 1H), 1.92 – 1.47 (m, 6H).

^{13}C NMR (151 MHz, $CDCl_3$) δ 141.9, 137.0 (t, *J* = 27.1 Hz), 130.7 (q, *J* = 32.6 Hz), 129.5 (t, J= 5.5 Hz), 128.8, 128.7, 128.5, 126.7- 126.6 (m), 126.1, 124.0 (q, J= 272.4 Hz), 123.5- 123.3 (m), 122.4 (t, J=247.1), 75.3, 66.1, 63.6 (t, *J* = 25.7 Hz), 59.2, 57.0, 32.7, 30.9, 26.5, 21.1.

^{19}F NMR (376 MHz, $CDCl_3$) δ -62.75 (s, 3F), -100.87 (dd, *J* = 251.3, 12.0 Hz, 1F), -103.94 (dd, *J* = 251.8, 12.8 Hz, 1F).

HRMS (APCI) *m/z*: [M+H] calcd. for $C_{23}H_{28}ON_2F_5$, 443.2116; found 443.2117.

Diastereomer 2:

R_f= 0.52 (30% EtOAc/hexanes)

^{1}H NMR (500 MHz, $CDCl_3$) δ 7.83 (s, 1H), 7.74 – 7.64 (m, 2H), 7.53 (t, *J* = 7.8 Hz, 1H), 7.29 – 7.25 (m, 2H), 7.23 – 7.16 (m, 1H), 7.15 – 7.11 (m, 2H), 3.49 – 3.27 (m, 2H), 3.26 – 3.23 (m, 1H), 3.23 (s, 3H), 3.09 – 2.92 (m, 1H), 2.87 – 2.78 (m, 1H), 2.77 – 2.64 (m, 1H), 2.58- 2.52 (m, 1H), 2.24 – 2.12 (q, J= 8.7 Hz, 1H), 2.06 – 1.92 (m, 1H), 1.91 – 1.78 (m, 1H), 1.78 – 1.59 (m, 3H), 1.58 – 1.45 (m, 1H).

^{13}C NMR (101 MHz, $CDCl_3$) δ 141.4, 137.6 (t, *J* = 27.0 Hz), 130.7 (q, J= 32.2 Hz), 129.7 (t, J= 6.0 Hz), 128.6, 128.5, 128.5, 126.6- 126.3 (m), 126.3, 124.1 (q, J= 273.02 Hz), 123.7- 123.4 (m), 122.2 (t, J= 247.1 Hz), 74.8, 66.1, 64.2 (t, *J* = 26.4 Hz), 59.1, 58.2, 32.6, 30.9, 26.2, 21.1.

^{19}F NMR (376 MHz, $CDCl_3$) δ -62.67, -98.78 (dd, *J* = 251.3, 9.5 Hz), -107.15 (dd, *J* = 251.1, 14.9 Hz).

HRMS (APCI) *m/z*: [M+H] calcd. for $C_{23}H_{28}ON_2F_5$, 443.2116; found 443.2118.

IV-C. 1.0 mmol Scale Reaction

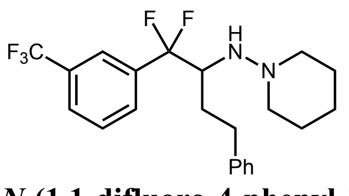

***N*-(1,1-difluoro-4-phenyl-1-(3-(trifluoromethyl)phenyl)butan-2-yl)piperidin-1-amine (3):** A 25 mL screw-top test tube was charged with 4CzIPN (10.0 µmol, 7.87 mg, 1 mol%), sodium formate (4.00 mmol, 0.270 g, 4.0 equiv), mesna (0.200 mmol, 32.8 mg, 20 mol%), and (*E*)-3-phenyl-*N*-(piperidin-1-yl)propan-1-imine **S6** (1.00 mmol, 216 mg, 1.0 equiv). The tube was equipped with a stir bar and was sealed with a PTFE/silicon septum. The atmosphere was exchanged by applying vacuum and backfilling with nitrogen (this process was conducted a total

of three times). Under nitrogen atmosphere, degassed DMSO (10 mL) was added via syringe followed by 1,3-bis(trifluoromethyl)benzene (3.00 mmol, 0.465 mL, 3.0 equiv) The resulting mixture was stirred for 16 h under irradiation by blue LEDs at 80 °C in an oil bath. Upon completion, the reaction was diluted with H_2O and extracted with EtOAc (3x). The combined organic layers were passed through silica to remove excess DMSO and concentrated *in vacuo*. The residue was then purified by silica chromatography (5-20% EtOAc/hexanes as the eluent) to afford the title compound as a yellow oil (316 mg, 77% yield).

IV-D. Telescoped Reaction Procedure

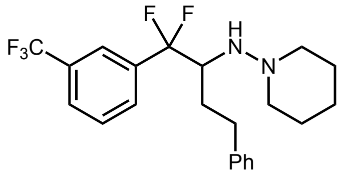

***N*-(1,1-difluoro-4-phenyl-1-(3-(trifluoromethyl)phenyl)butan-2-yl)piperidin-1-amine (3):** A 15 mL screw-top test tube was charged with 4CzIPN (5.00 μmol, 3.93 mg, *1* mol%), sodium formate (2.00 mmol, 136 mg, 4.0 equiv), mesna (0.100 mmol, 0.0164 g, 20 mol%). The tube was equipped with a stir bar and was sealed with a PTFE/silicon septum. The atmosphere was exchanged by applying vacuum and backfilling with nitrogen (this process was conducted a total of three times). Under nitrogen atmosphere, the degassed DMSO (5 mL) was added via syringe followed by 3-phenylpropanal (0.500 mmol, 65.8 μL, 1 equiv), N-aminopiperidine (0.500 mmol, 54.0 μL, 1.0 equiv) and 1,3-bis(trifluoromethyl)benzene (1.50 mmol, 0.233 mL, 3.0 equiv). The reaction was allowed to stir for 5 minutes before placing under lamps. The resulting mixture was stirred for 16 h under irradiation by blue LEDs at 80 °C in an oil bath. Upon completion, the reaction was diluted with H_2O and extracted with EtOAc (3x). The combined organic layers were passed through silica to remove excess DMSO and concentrated *in vacuo*. The residue was purified by silica chromatography (3-12% EtOAc/hexanes as the eluent) to afford the title compound as a yellow oil (107 mg, 52% yield).

IV-E. Unsuccessful Substrates

CF_3- Arenes:

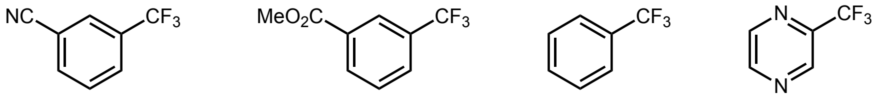

Hydrazones:

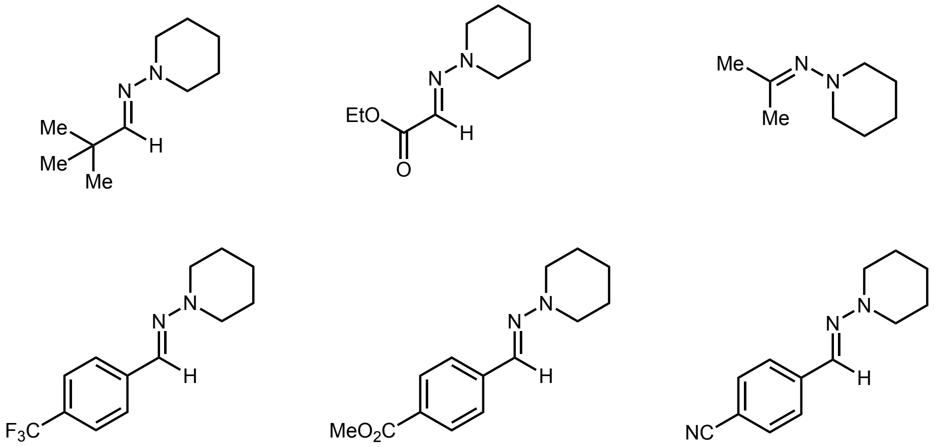

V. Nitrogen-Nitrogen Bond Cleavage

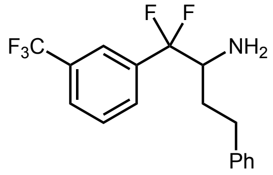

1,1-difluoro-4-phenyl-1-(3-(trifluoromethyl)phenyl)butan-2-amine (31): A 7-mL screw top vial equipped with a stir bar was charged with *N*-(1,1-difluoro-4-phenyl-1-(3-(trifluoromethyl)phenyl)butan-2-yl)piperidin-1-amine **3** (0.100 mmol, 412 mg, 1.0 equiv) and Raney nickel (0.310 g). The atmosphere was exchanged for N_2 (3x) and freshly degassed MeOH (2.5 mL) was added via syringe. The reactions atmosphere was sparged with H_2 gas. The reaction was heated to 55 °C using a heating mantle and stirred under a balloon of H_2 for 16 hours. The reaction was cooled to room temperature, filtered through celite and concentrated. The crude material was purified via silica chromatography (5- 30% EtOAc/hexanes as the eluent) to afford the title compound as a clear oil (25.9 mg, 79% yield).
R_f=0.36 (50% EtOAc/hexanes)
^{1}H NMR (600 MHz, $CDCl_3$) δ 7.71 (d, *J* = 8.5 Hz, 1H), 7.70 (s, 1H), 7.62 (d, *J* = 7.9 Hz, 1H), 7.55 (t, *J* = 7.6 Hz, 1H), 7.26 (t, *J* = 7.4 Hz, 2H), 7.20 (t, J= 7.4 Hz, 1H), 7.12 (d, J= 7.4 Hz, 2H), 3.21 – 3.12 (m, 1H), 2.93 – 2.85 (m, 1H), 2.69 – 2.61 (m, 1H), 1.90– 1.84 (m, 1H), 1.54 – 1.45 (m, 1fH), 1.29 (br s, 2H).
^{13}C NMR (101 MHz, $CDCl_3$) δ 141.3, 136.5 (t, *J* = 27.4 Hz), 131.2 (q, *J* = 32.8 Hz), 129.4 (t, *J* = 6.4 Hz), 129.1, 128.6, 128.5, 127.0- 126.8 (m), 126.2, 123.9 (q, J= 272.49), 123.1 – 122.8 (m), 122.5 (dd, J= 246.48, 247.16), 56.8 (t, *J* = 27.6 Hz), 32.3, 32.0 (t, *J* = 2.7 Hz).
^{19}F NMR (376 MHz, $CDCl_3$) δ -62.75 (s, 3F), -104.68 (dd, *J* = 246.2, 10.8 Hz, 1F), -109.63 (dd, *J* = 246.6, 13.5 Hz, 1F).
HRMS (APCI) *m/z*: [M+H] calcd. for $C_{17}H_{17}NF_5$, 330.1276; found 330.1276.

VI. Alternative Initiators

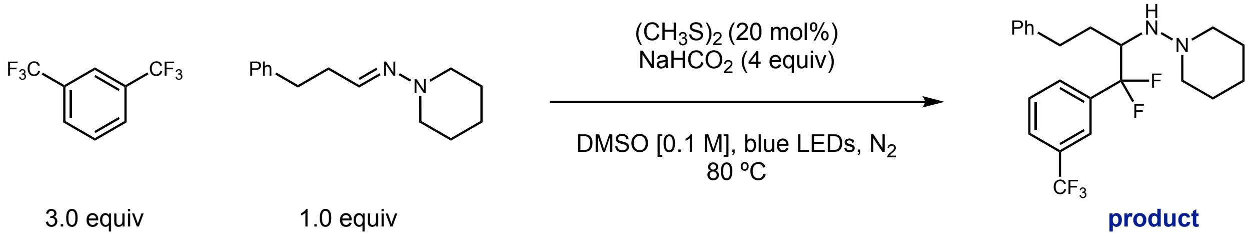

Procedure: An 8 mL screw-top test tube was charged with sodium formate (0.400 mmol, 27.2 mg),and (*E*)-3-phenyl-*N*-(piperidin-1-yl)propan-1-imine **S6** (0.100 mmol, 21.6 mg, 0.1 mmol). The tube was equipped with a stir bar and sealed with a PTFE/silicon septum. Under nitrogen atmosphere, separately degassed DMSO (1 mL) was added via syringe, followed by 1,3-bis(trifluoromethyl)benzene (0.300 mmol, 47.0 μL, 3.0 equiv) and dimethyl disulfide (0.020 mmol, 1.80 μL, 20 mol%). The resulting mixture was heated to 80 °C and stirred at 700 RPM for 16 h under irradiation by blue LEDs. The reaction was cooled to room temperature and 2-(trifluoromethyl)pyridine (0.1 mmol) was added as the internal standard. A small aliquot of the reaction mixture was diluted in d_6-DMSO and the sample was analyzed by ^{19}F NMR. The integral values were used to calculate the yield of the product (59% yield).

VII. References

(1) Luo, J.; Zhang, J. "Donor-Acceptor Fluorophores for Visible-Light-Promoted Organic Synthesis: Photoredox/ Ni Dual Catalytic C(sp^3)-C(sp^2) Cross- Coupling." *ACS. Catal.* **2016**, 6, 873–877.

(2) Hutchyby, M.; Houlden, C.; Haddow, M.; Tyler, S.; Lllyod-Jones, G.; Booker-Milburn, K. "Switching Pathways: Room-Termperature Neutral Solvolysis and Substitution of Amides." *Angew. Chem. Int. Ed.* 51, 548- 551.

(3) Wright, S.; Bandar, J. "A Base-Promoted Reductive Coupling Platform for the Divergent Defluorofunctionalization of Trifluoromethylarenes." *J. Am. Chem. Soc.* **2022**, 144, 13032-13038.

(4) Leow, D. "Phenazinium Salt-Catalyzed Aerobic Oxidative Amidation of Aromatic Aldehydes." *Org. Lett.* **2014**, 16, 5812-5815.

(5) Rene, O.; Souverneva, A.; Magnusion, S.; Fauber, B. "Efficient syntheses of 2-fluoroalkylbenzimidazoles and -benzothiazoles." *Tetrahedron Lett.* **2013**, 54, 201-204.

(6) Marqués-López, E.; Herrera, R.; Fernández, R.; Lassaletta, J. "Uncatalyzed Strecker-Type Reaction of N,N-Dialkylhydrazones in Pure Water." *Eur. J. Chem.* **2008**, 3457-3460.

(7) Monge, D.; Martín-Zamora, E.; Vázquez, H,; Alcarazo, M.; Álvarez, E.; Fernández, R.; Lassaletta, J. "Enantioselective Conjugate addition of N,N-Dialkylhydrazones to α- Hydroxy Enones." *Org. Lett.* **2007**, 9, 2867- 2870.

(8) Panda, A.; Das, U.; Dimmock, J. "Synthesis of Bicyclic N-Methylpyrazoline and Pyrazole Derivatives from α, β-Unsaturated Ketones and N,N-Dimethylhydrazine: An Illustration of Reductive Cyclization." J. *Heterocycl. Chem.* **2014**, 5, 219- 223.

(9) Vega, J.; Alonso, J.; Méndez, G.; Ciordia, M.; Delgado, F.; Trabanco, A. "Continuous Flow α -Arylation of N,N-Dialkylhydrazones under Visible Light Photoredox Catalysis." *Org. Lett.* **2017**, 19, 938–941.

(10) Zhang, W.; Mo, J.; He, W.; Kennepohl, P.; Sammis, G. "Regiocontrolled and Stereoselective Synthesis of Tetrahydrophthalazine Derivatives using Radical Cyclizations." *Chem. Eur. J.* **2019**, 25, 976-980.

(11) Demark, S.E.; Edwards, J.P.; Weber, T.; Piotrowski, D.W. "Organocerium Additions to Proline-Derived Hydrazones: Synthesis of Enantiomerically Enriched Amines." *Tetrahedron Asymmetry.* **2010**, 21, 1278-1302.

VIII. Spectra

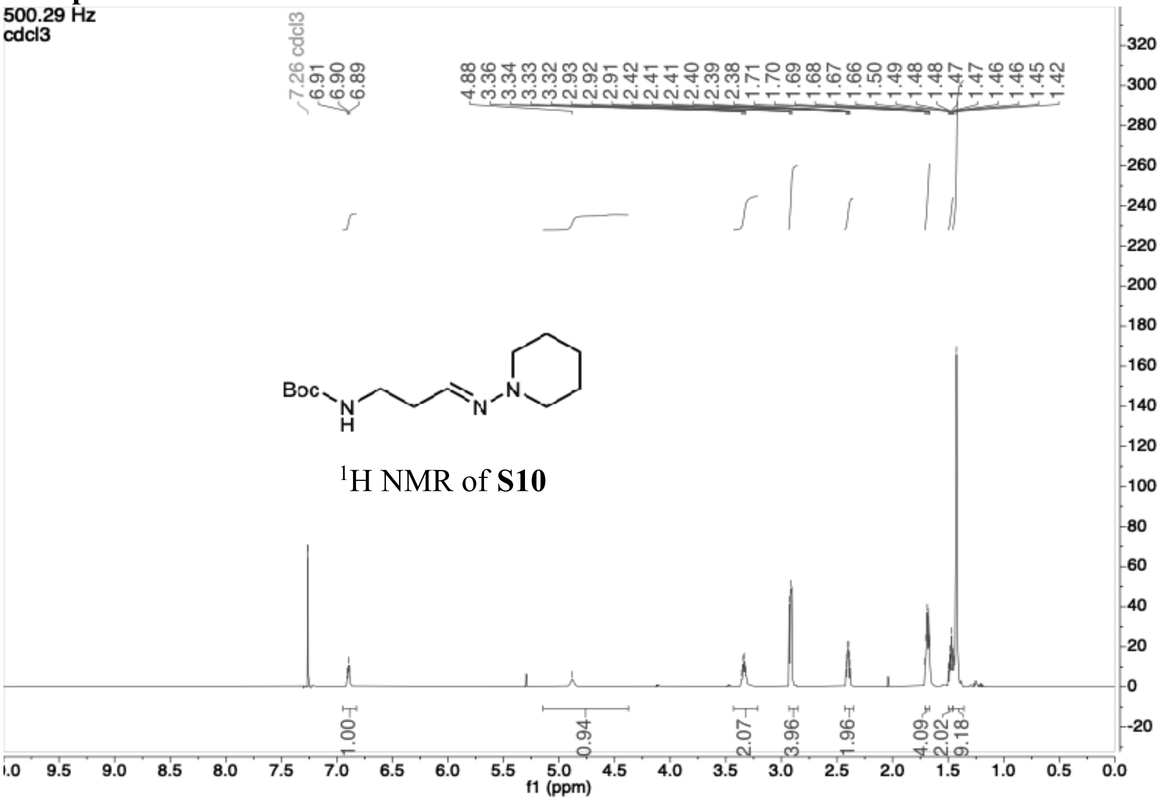

^{1}H NMR of **S10**

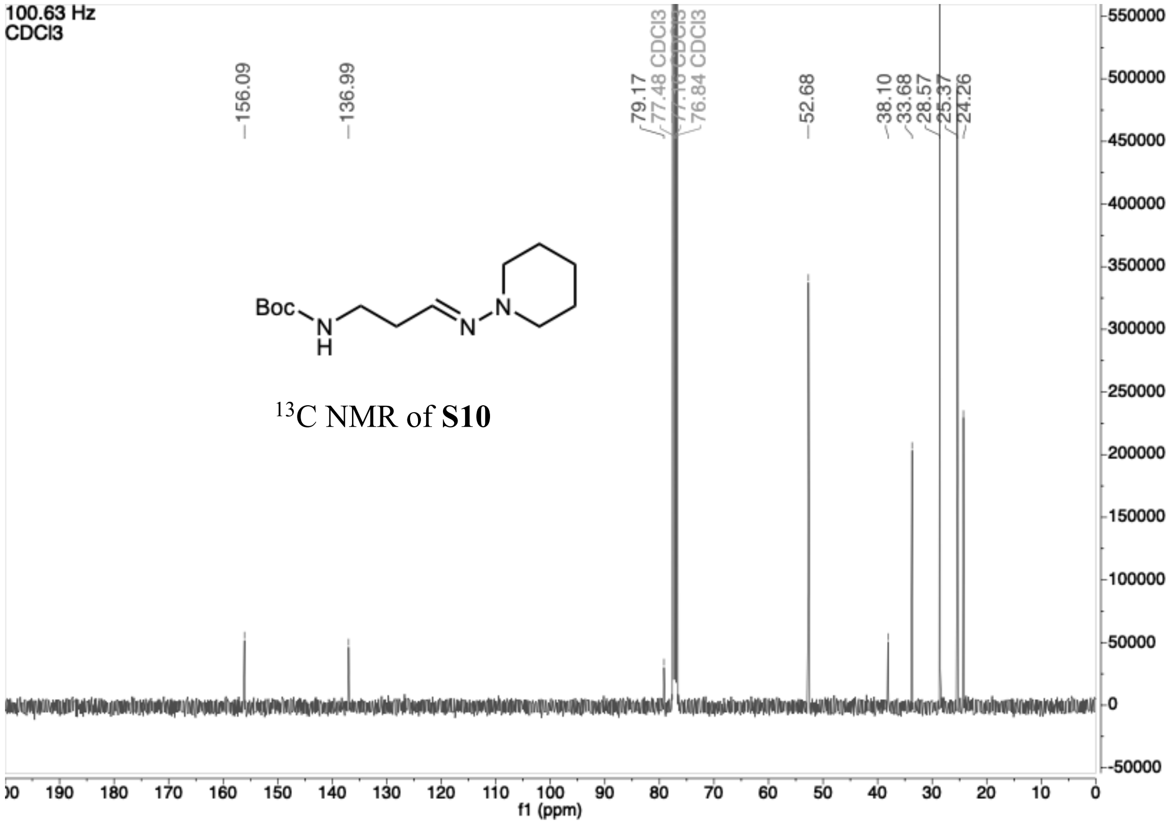

^{13}C NMR of **S10**

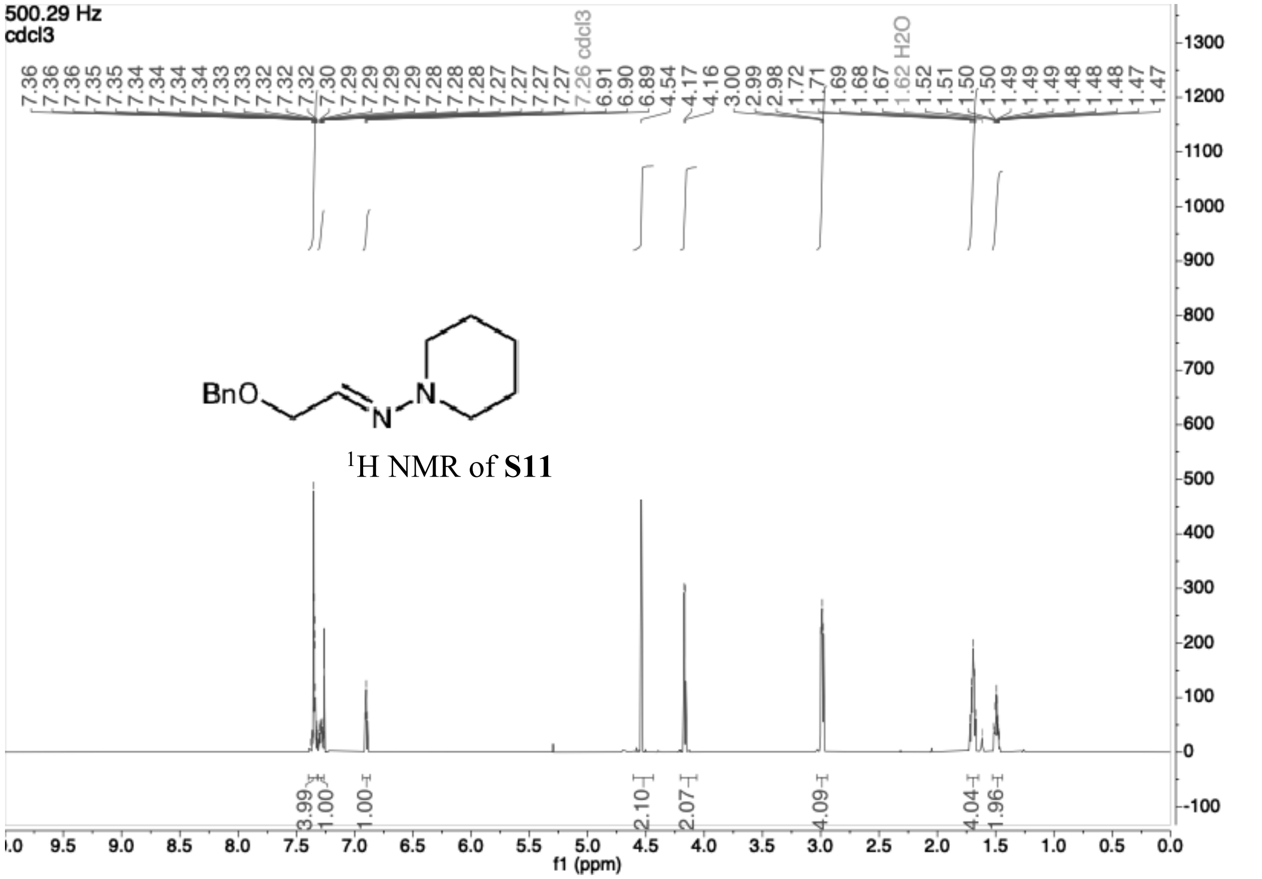

^{1}H NMR of **S11**

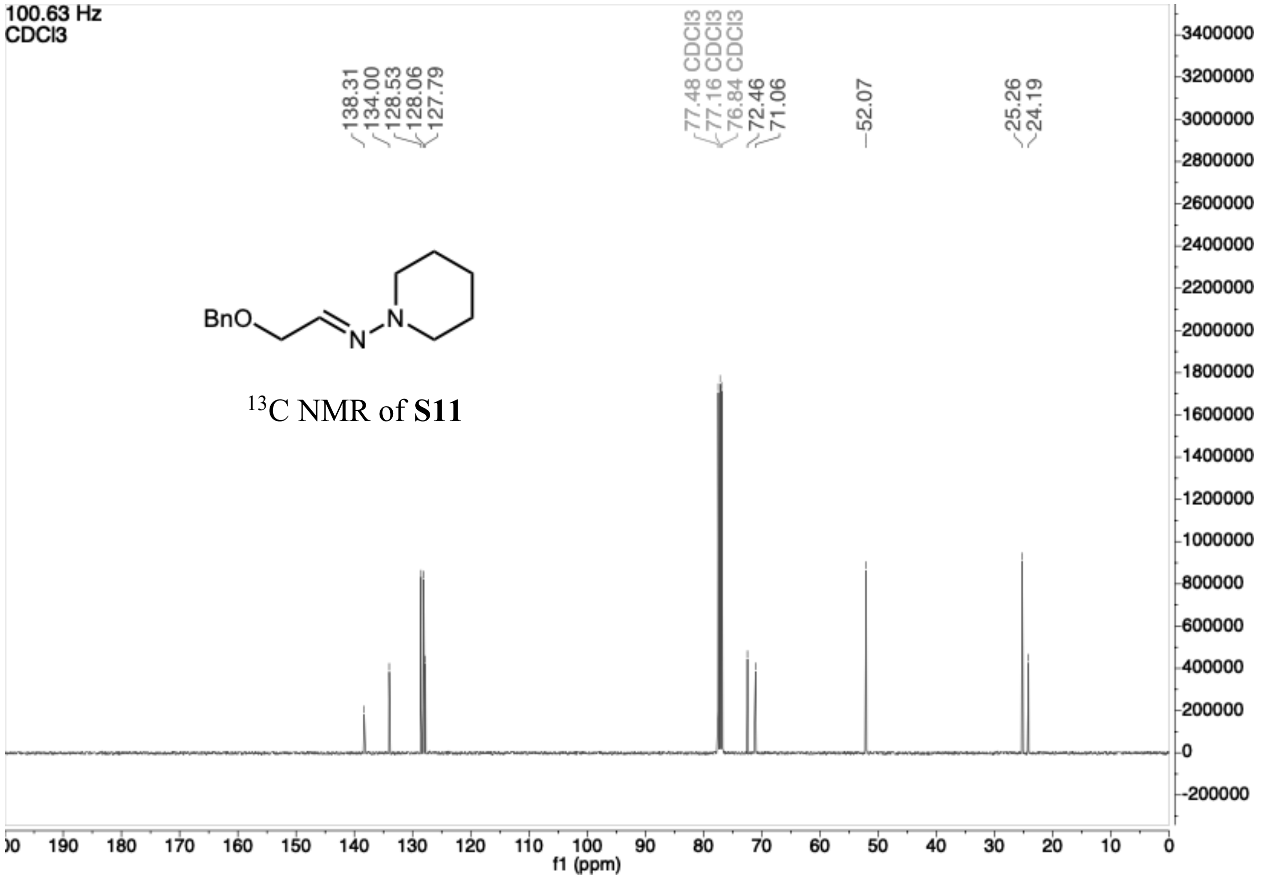

^{13}C NMR of **S11**

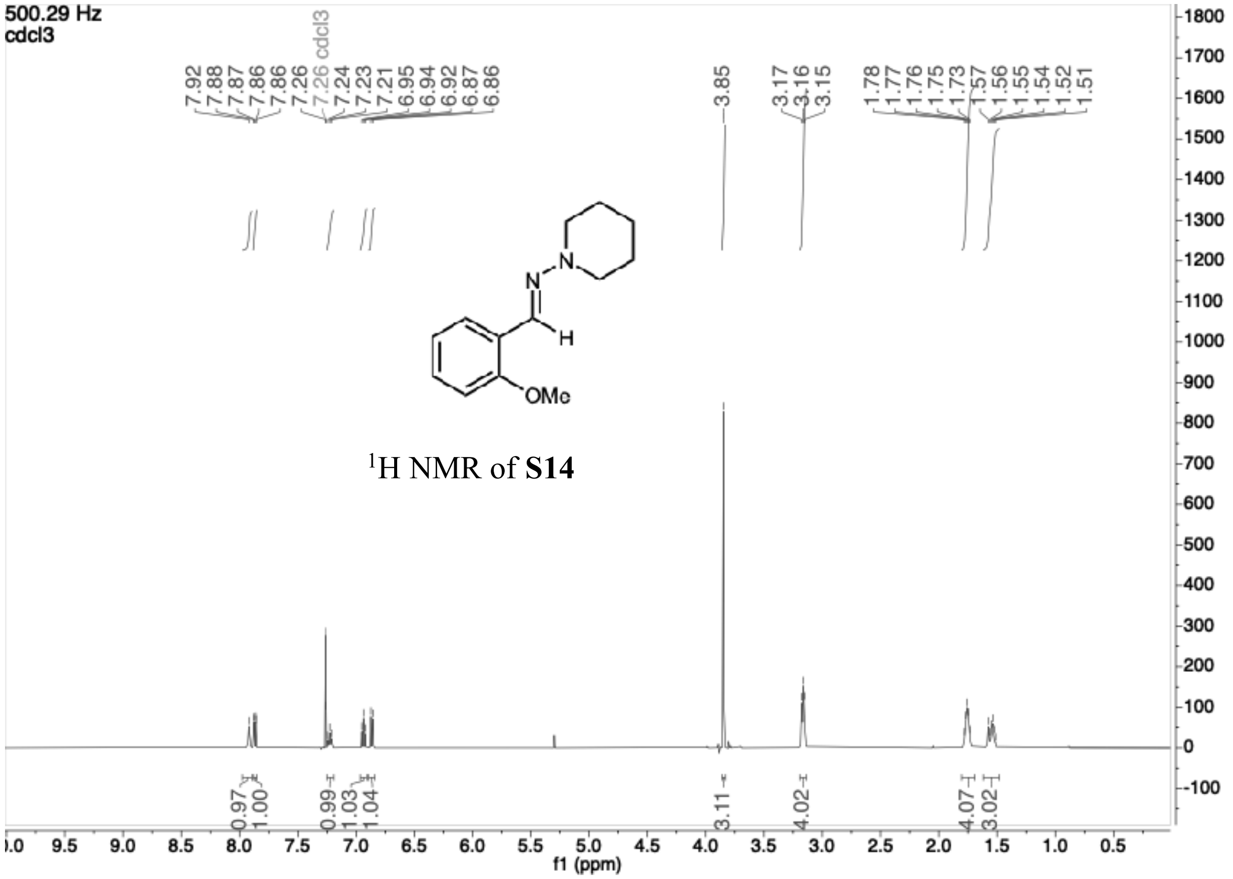
500.29 Hz
cdcl3
7.92 7.88 7.87 7.86 7.86 7.26 cdcl3 7.24 7.23 7.21 6.95 6.94 6.92 6.87 6.86
3.85
3.17 3.16 3.15
1.78 1.77 1.76 1.75 1.73 1.57 1.56 1.55 1.54 1.52 1.51
N
N
H
OMe
^{1}H NMR of **S14**
0.97 1.00 0.99 1.03 1.04 3.11 4.02 4.07 3.02
f1 (ppm)

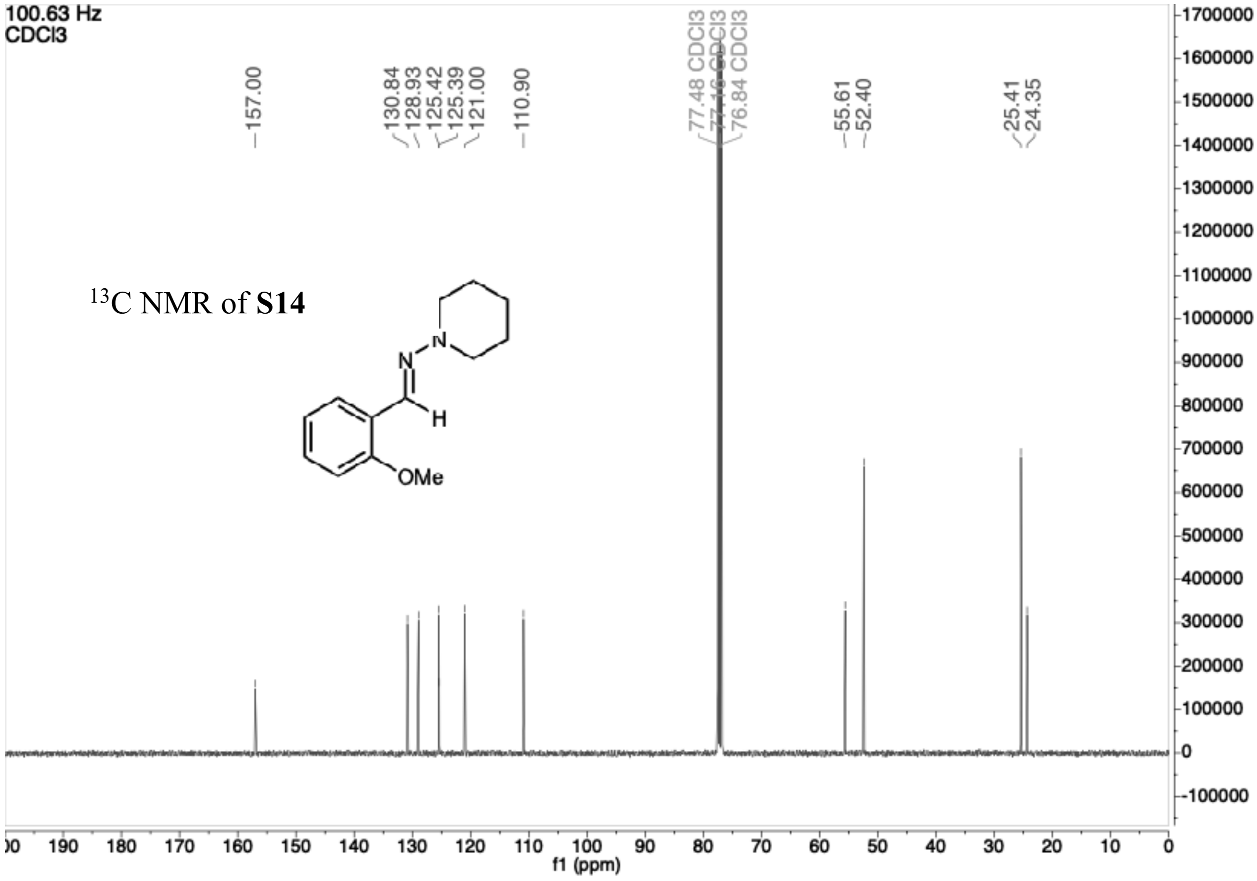
100.63 Hz
CDCl3
157.00
130.84 128.93 125.42 125.39 121.00
110.90
77.48 CDCl3 77.16 CDCl3 76.84 CDCl3
55.61 52.40
25.41 24.35
^{13}C NMR of **S14**
N
N
H
OMe
f1 (ppm)

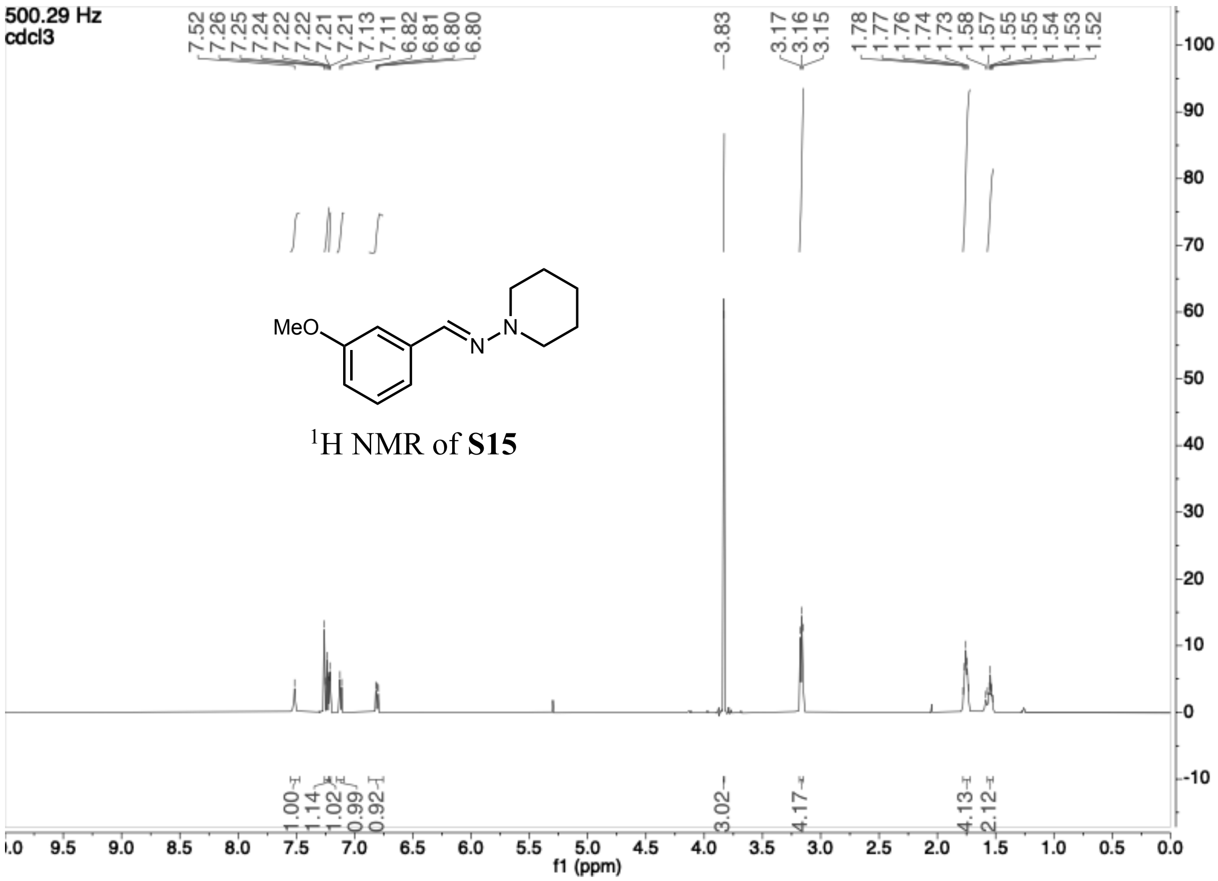

^{1}H NMR of **S15**

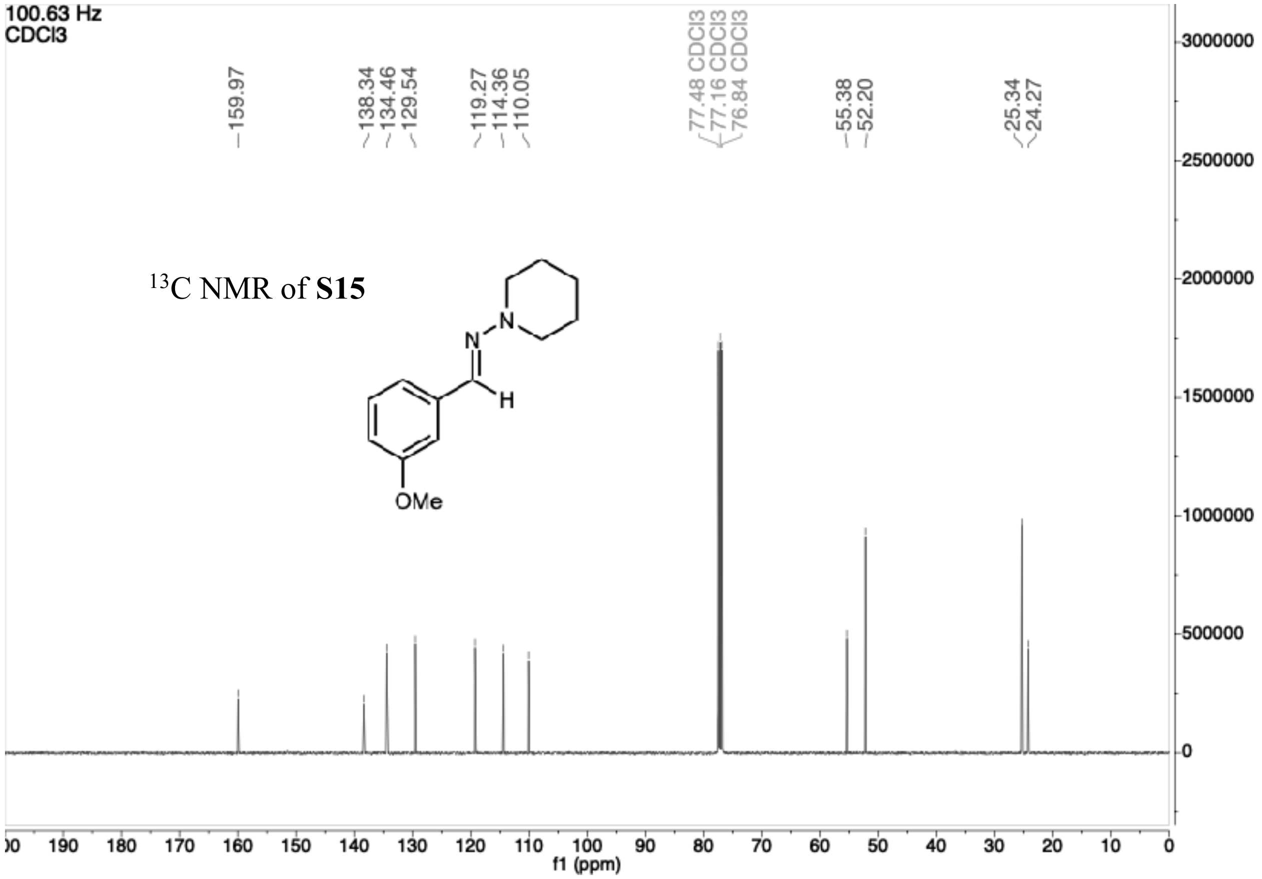

^{13}C NMR of **S15**

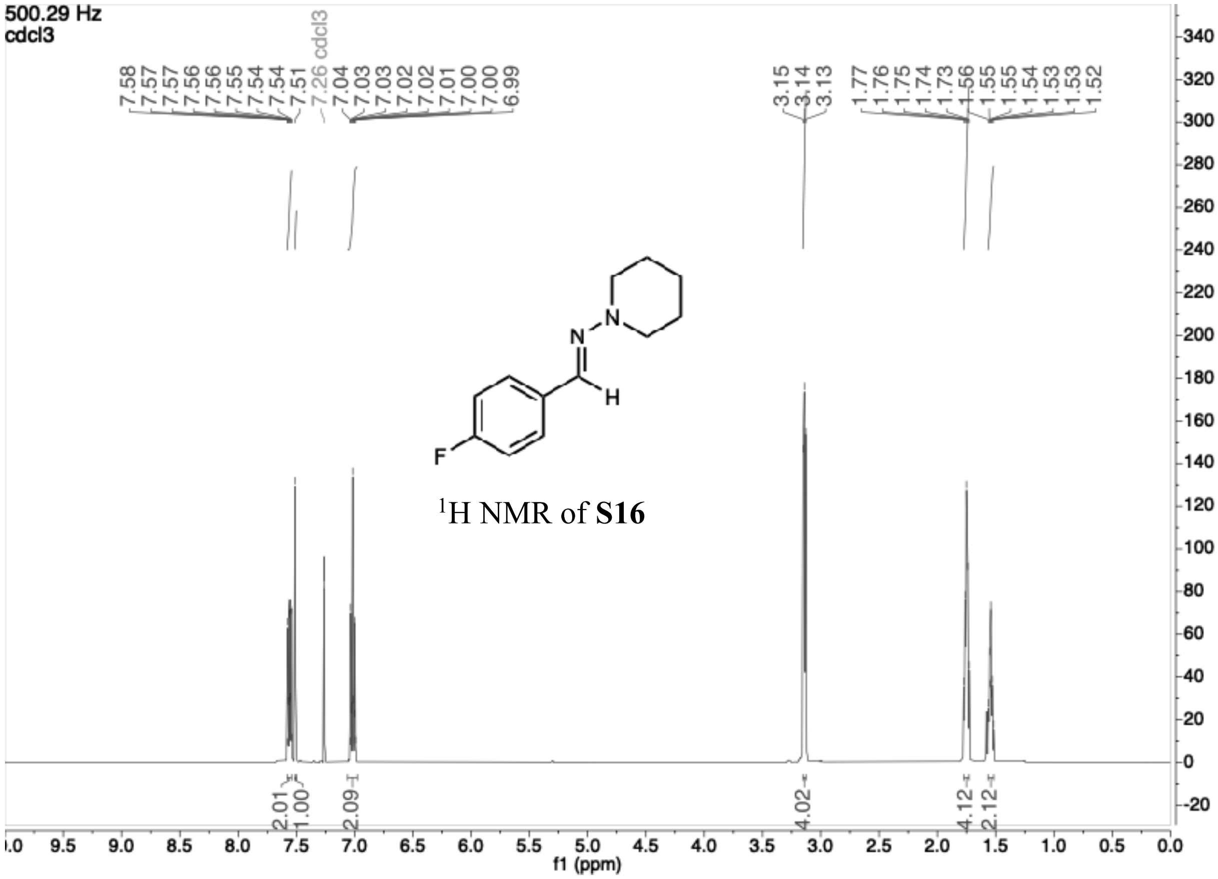

^{1}H NMR of **S16**

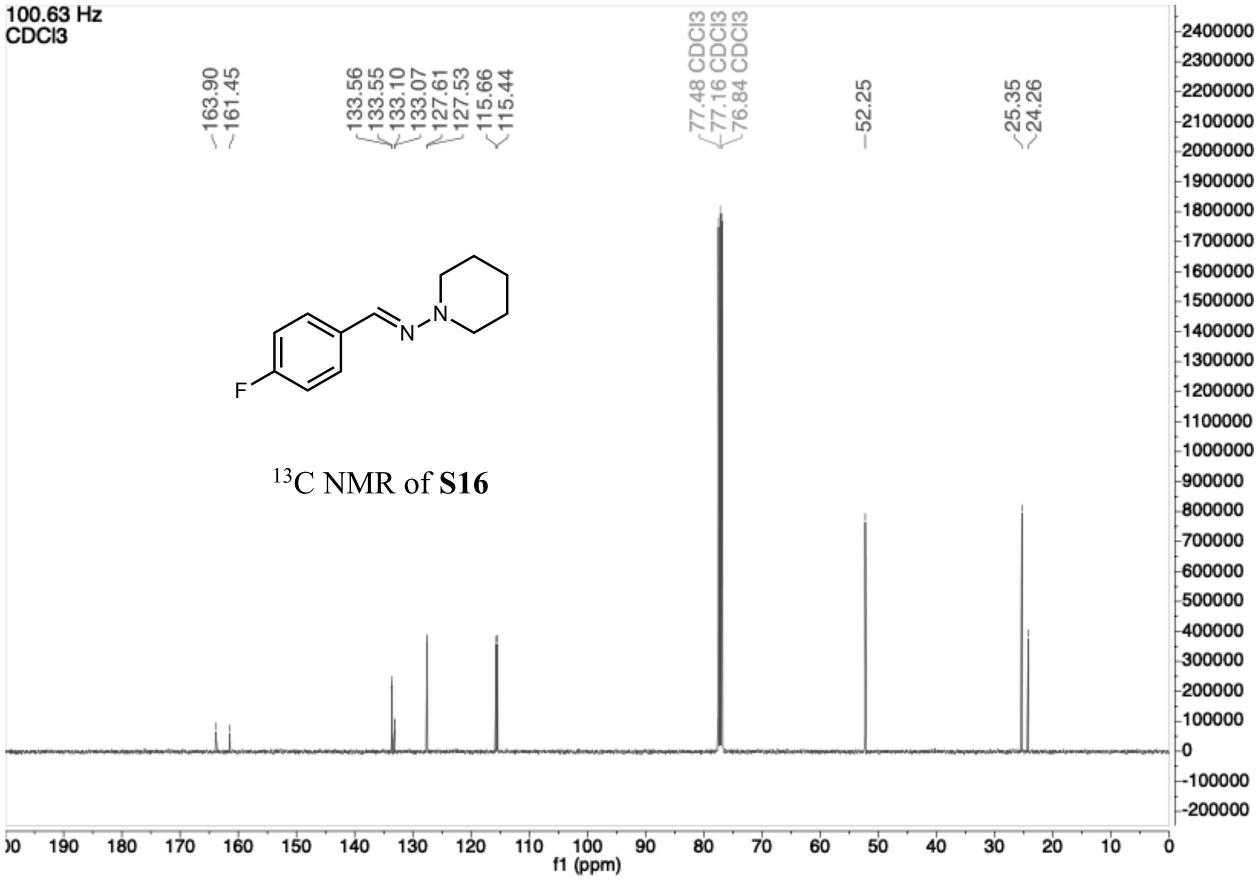

^{13}C NMR of **S16**

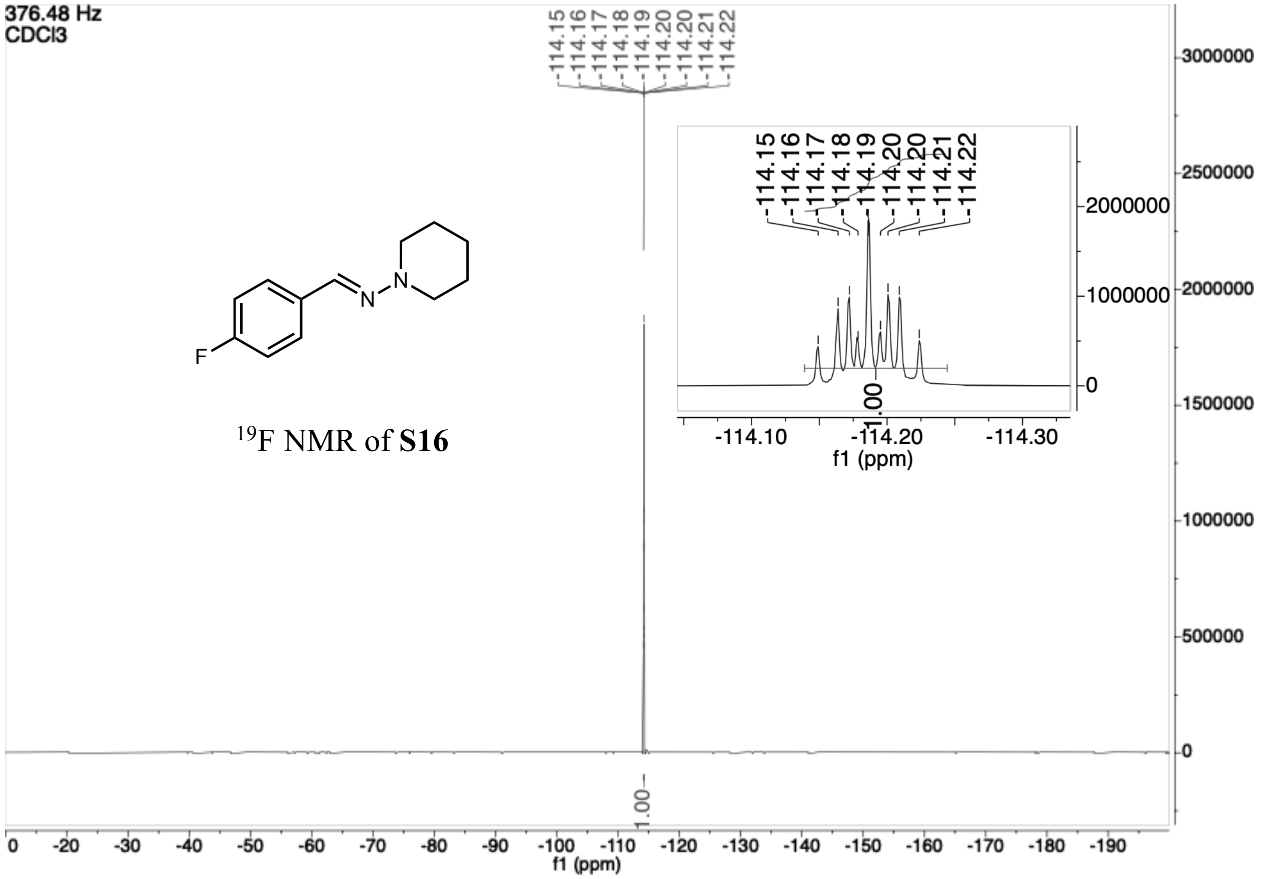

^{19}F NMR of **S16**

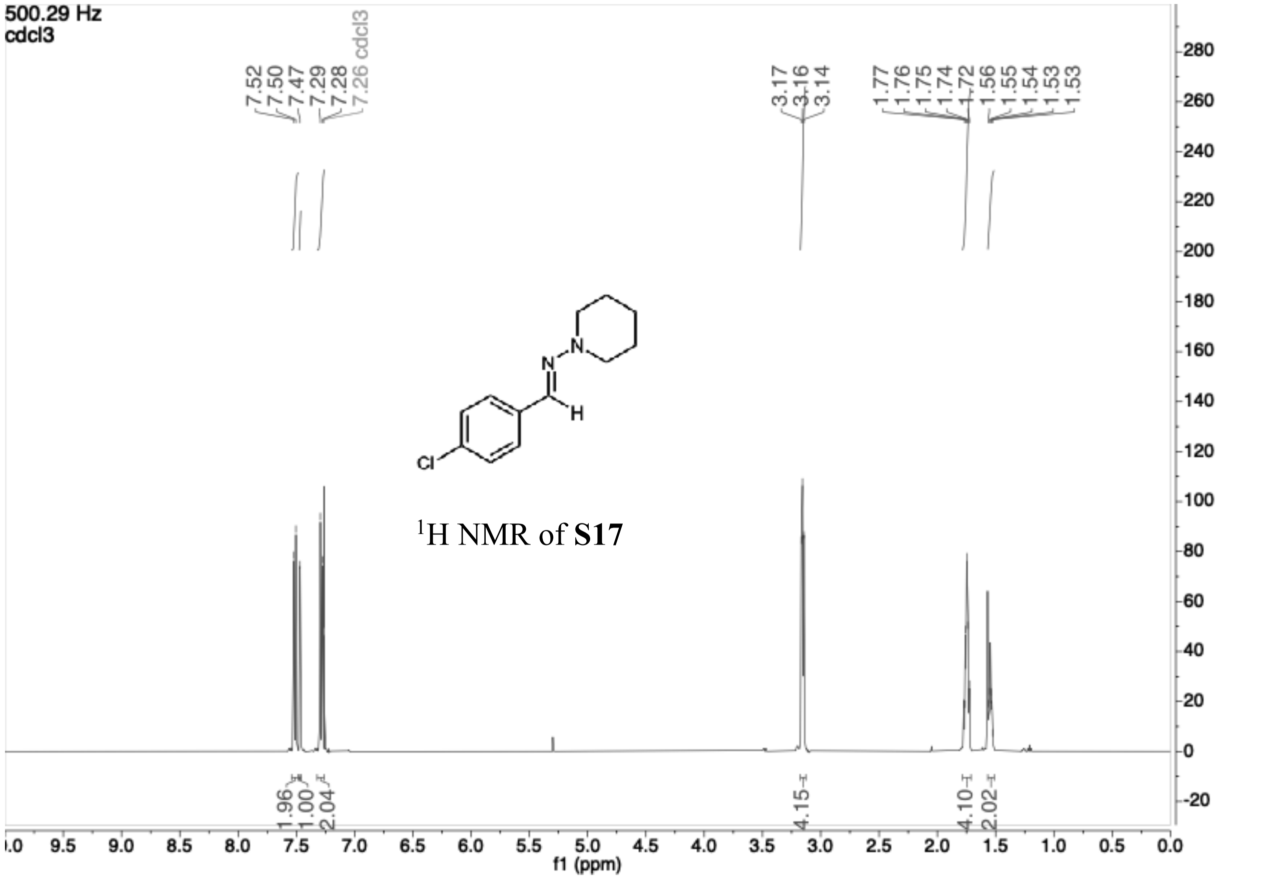

^{1}H NMR of **S17**

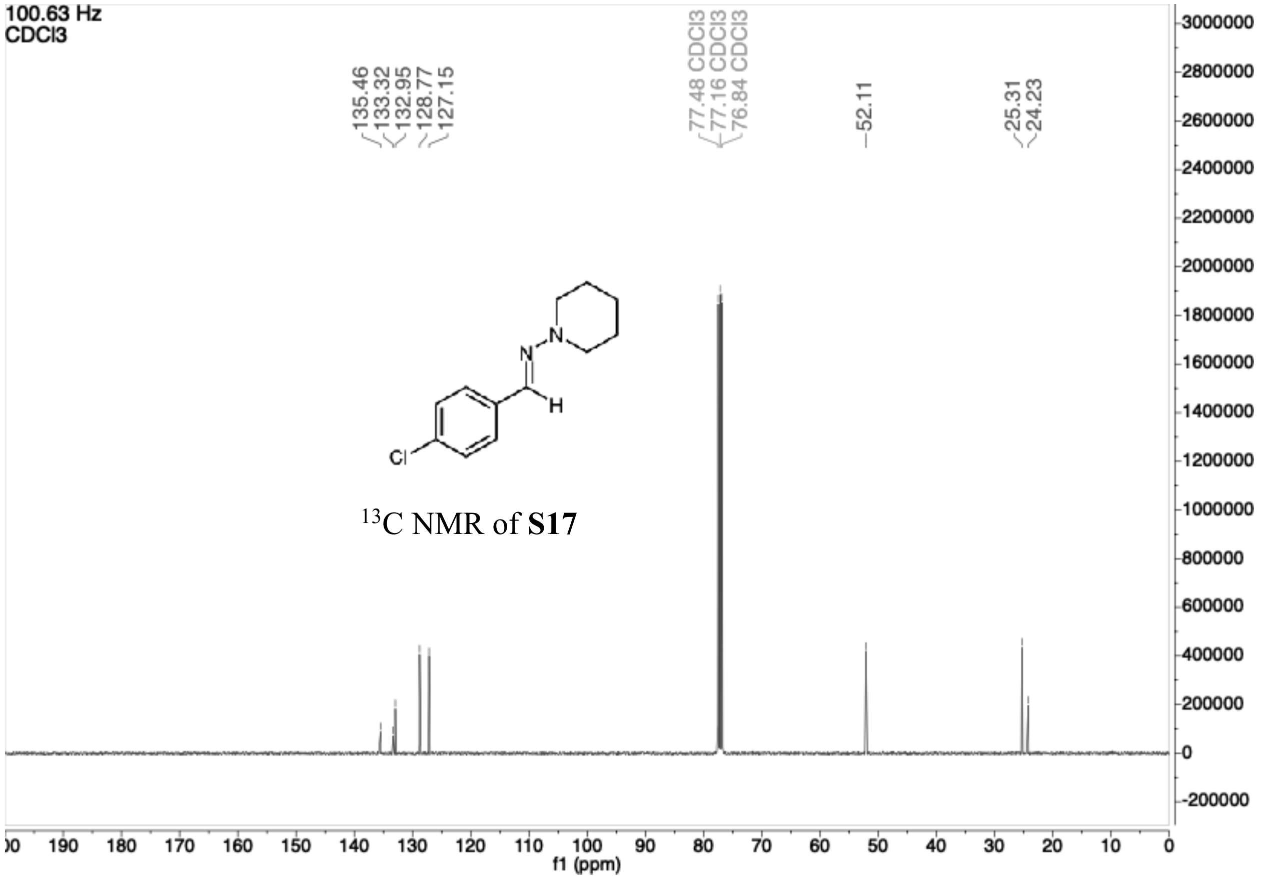

^{13}C NMR of **S17**

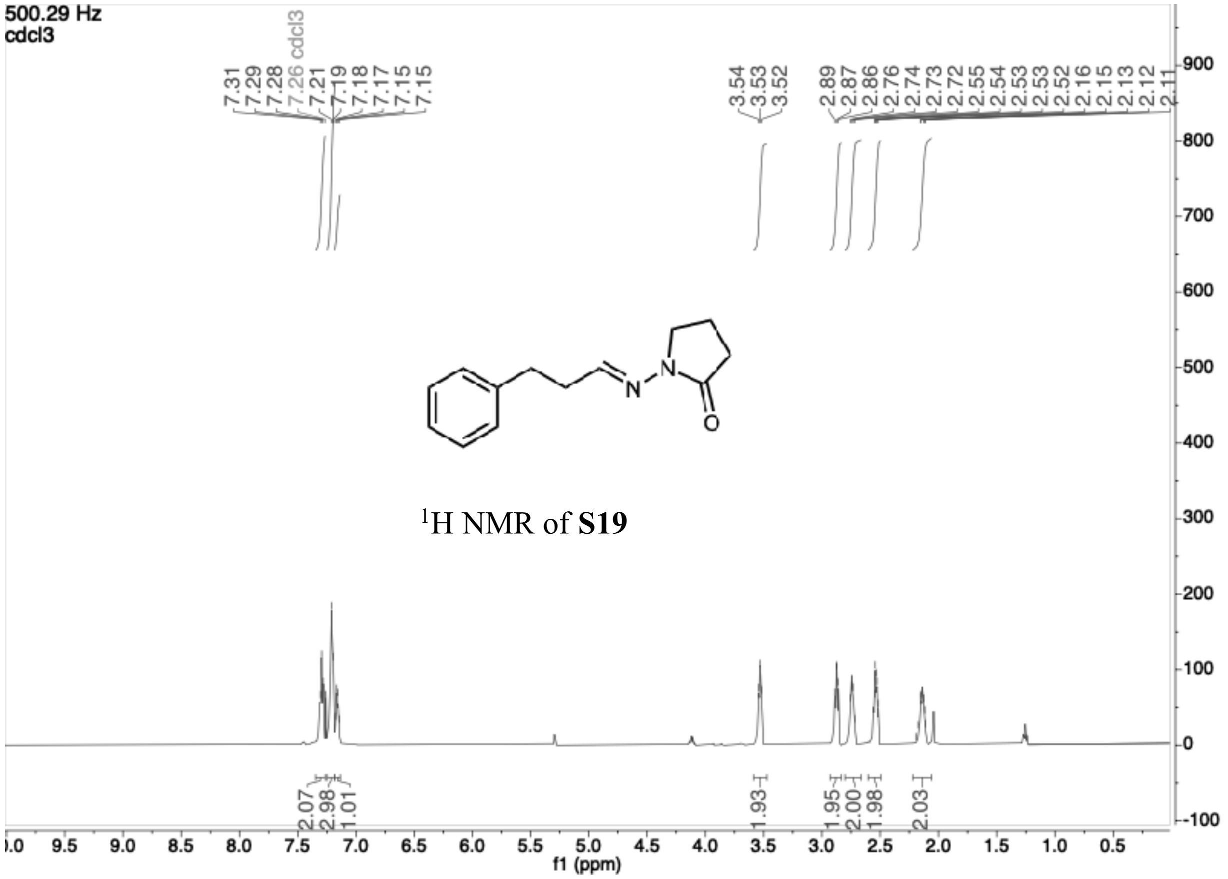

^{1}H NMR of **S19**

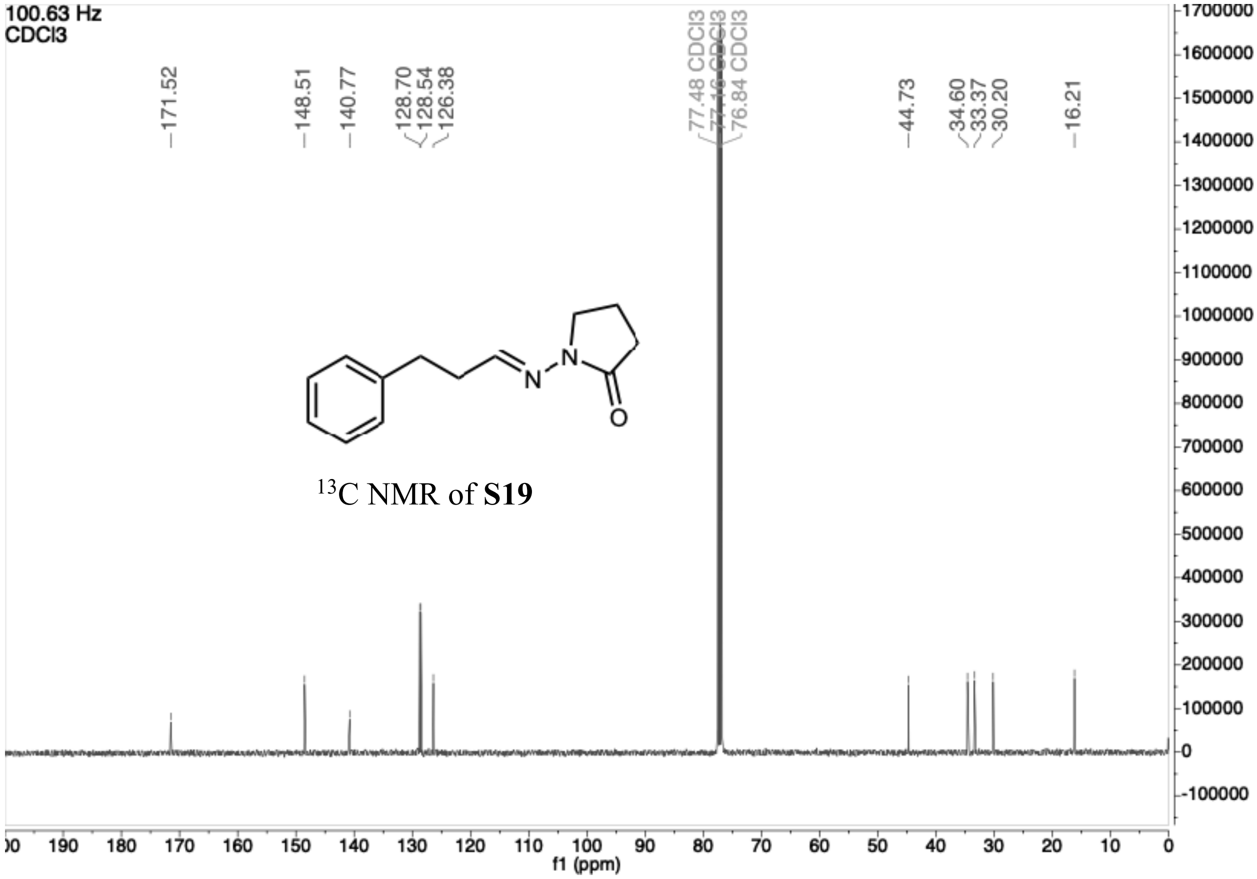

^{13}C NMR of **S19**

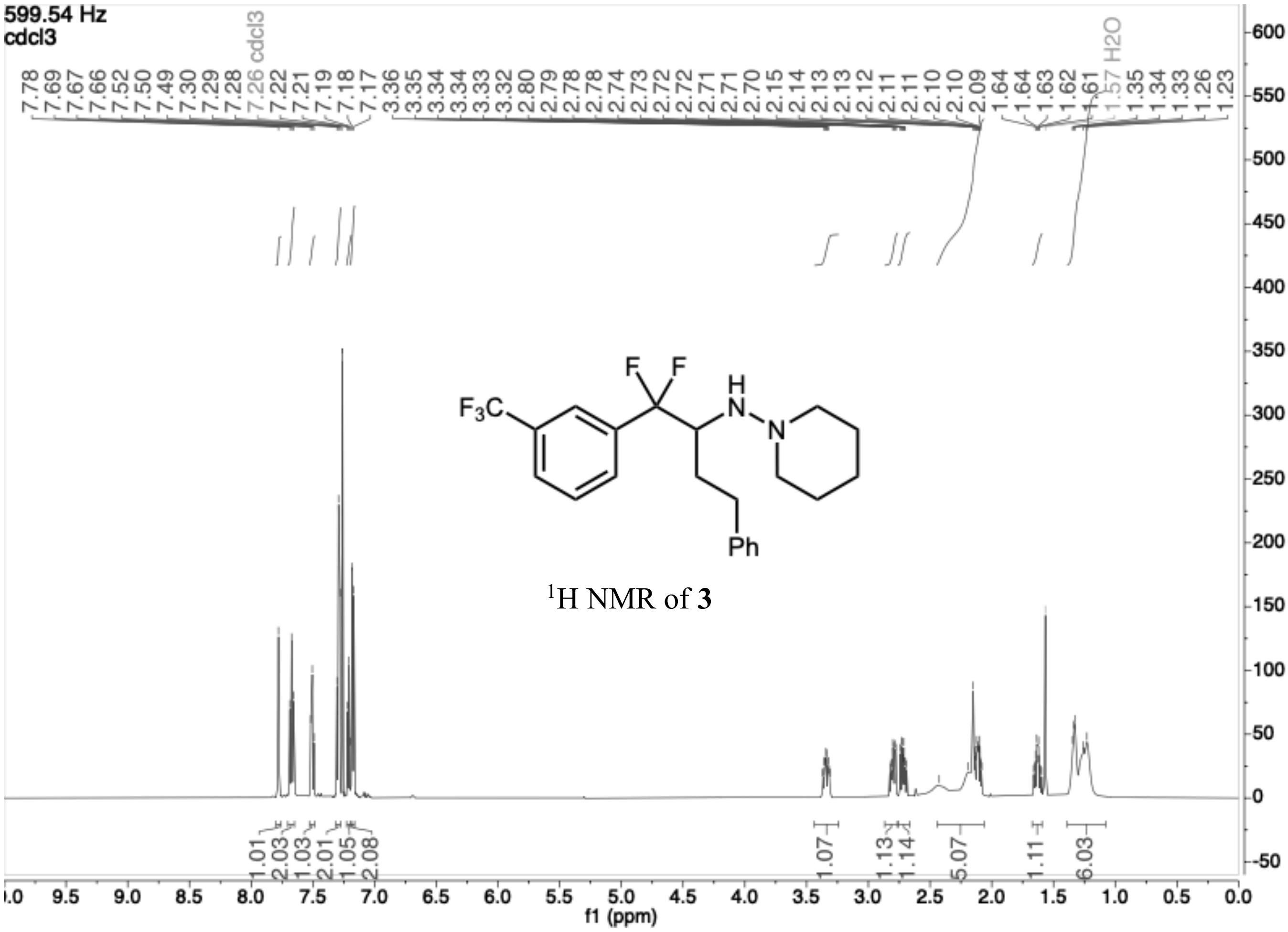

^{1}H NMR of **3**

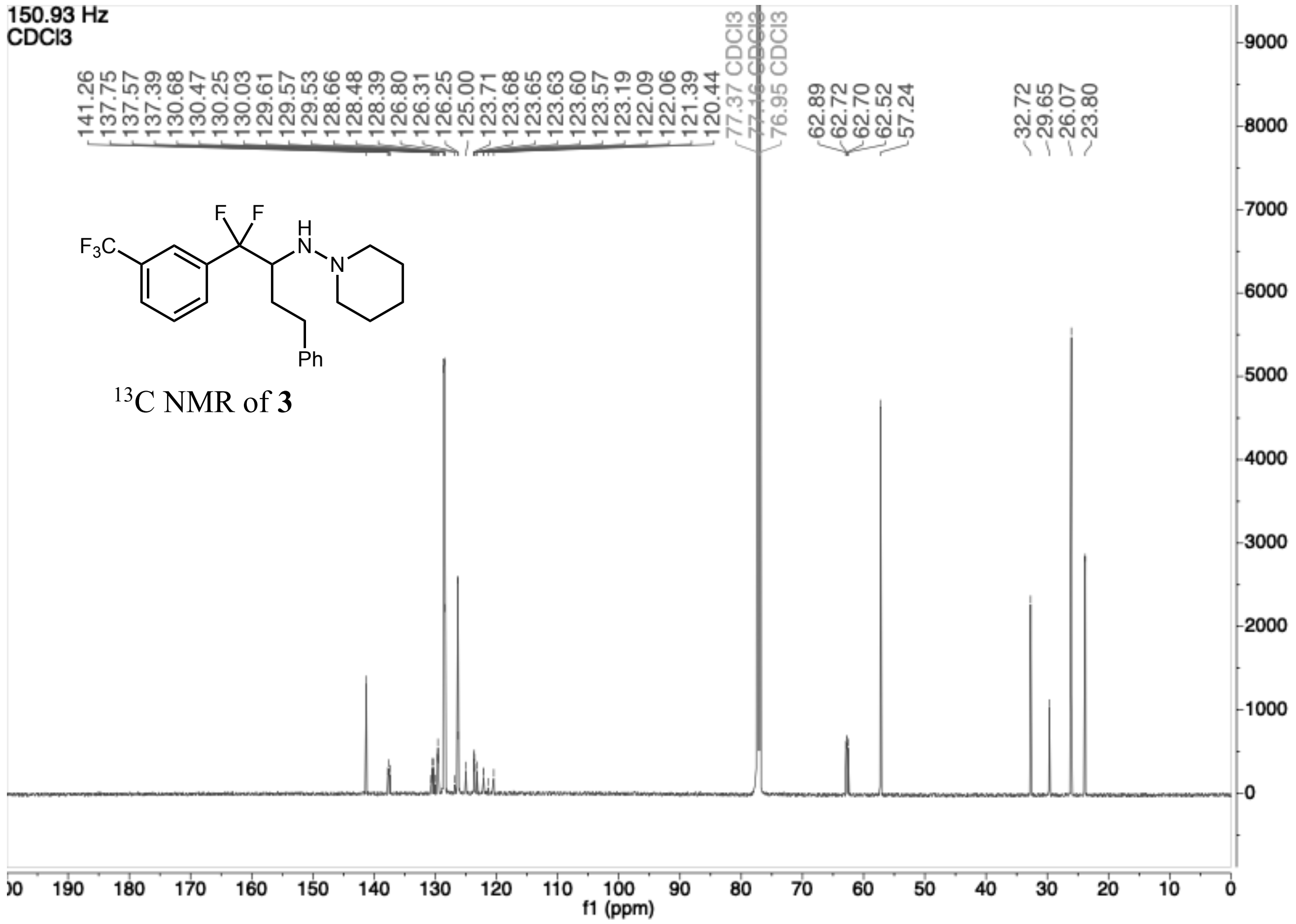
150.93 Hz
CDCl3
141.26
137.75
137.57
137.39
130.68
130.47
130.25
130.03
129.61
129.57
129.53
128.66
128.48
128.39
126.80
126.31
126.25
125.00
123.71
123.68
123.65
123.63
123.60
123.57
123.19
122.09
122.06
121.39
120.44
77.37 CDCl3
77.16 CDCl3
76.95 CDCl3
62.89
62.72
62.70
62.52
57.24
32.72
29.65
26.07
23.80
13C NMR of 3
f1 (ppm)

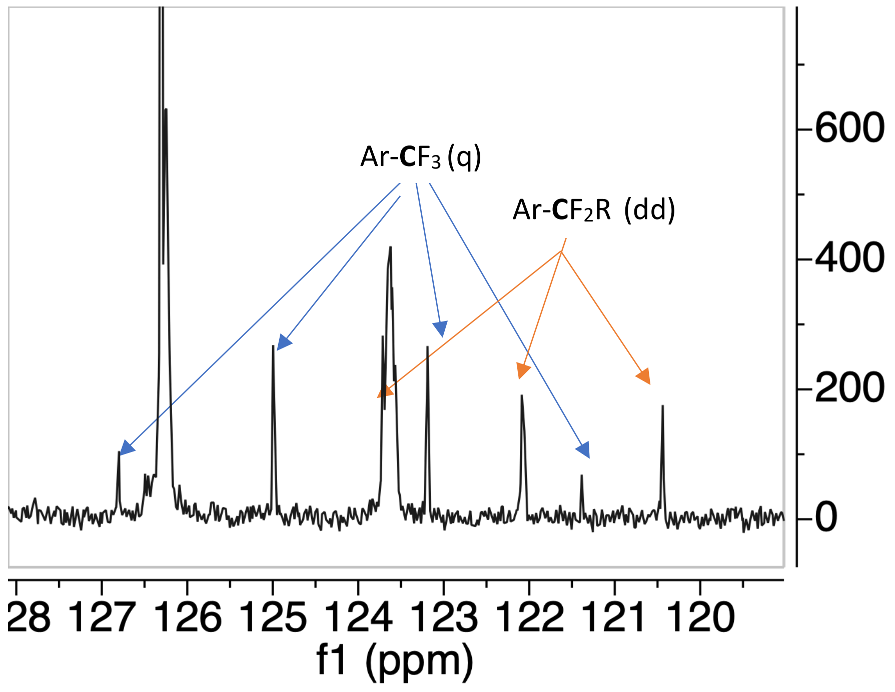
Ar-CF3 (q)
Ar-CF2R (dd)
f1 (ppm)

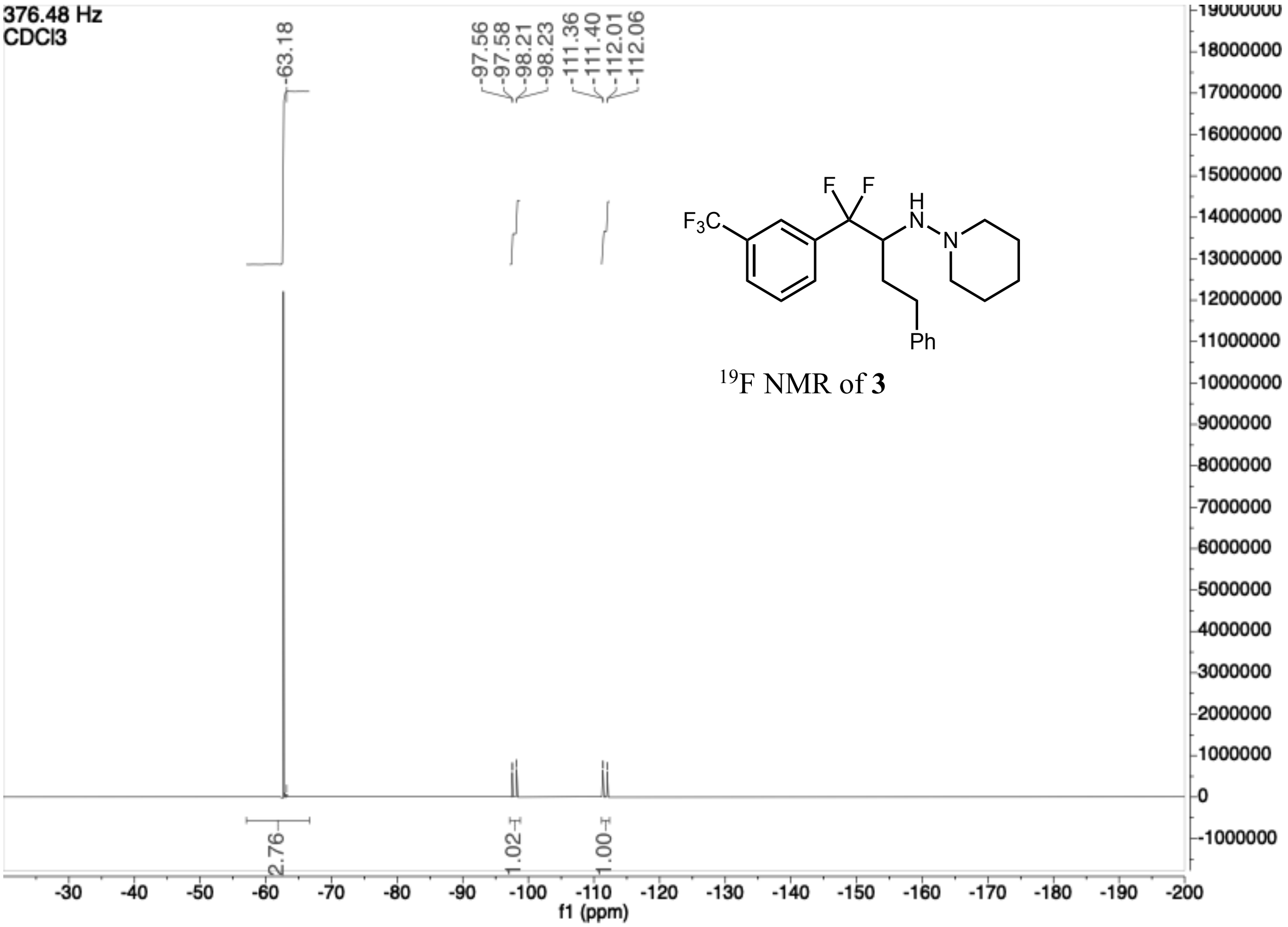
376.48 Hz
CDCl3
-63.18
-97.56
-97.58
-98.21
-98.23
-111.36
-111.40
-112.01
-112.06
2.76
1.02
1.00
f1 (ppm)
F_3C
F F
H
N
N
Ph

^{19}F NMR of **3**

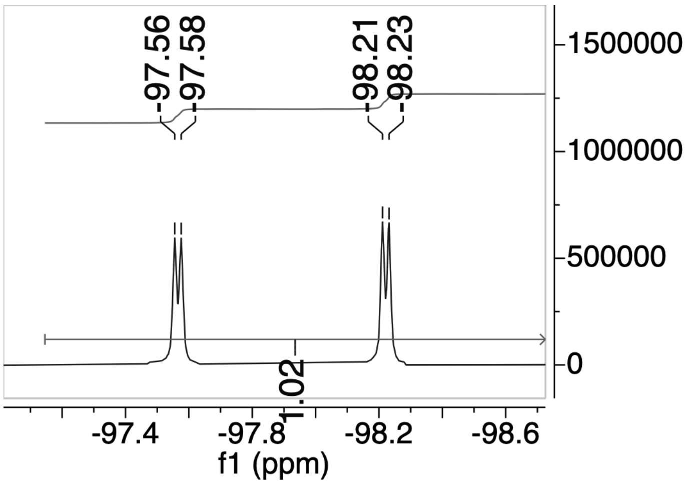
-97.56
-97.58
-98.21
-98.23
1.02
f1 (ppm)

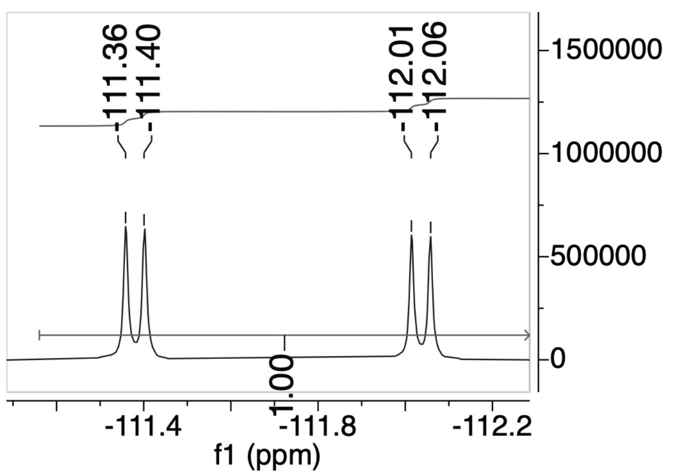
-111.36
-111.40
-112.01
-112.06
1.00
f1 (ppm)

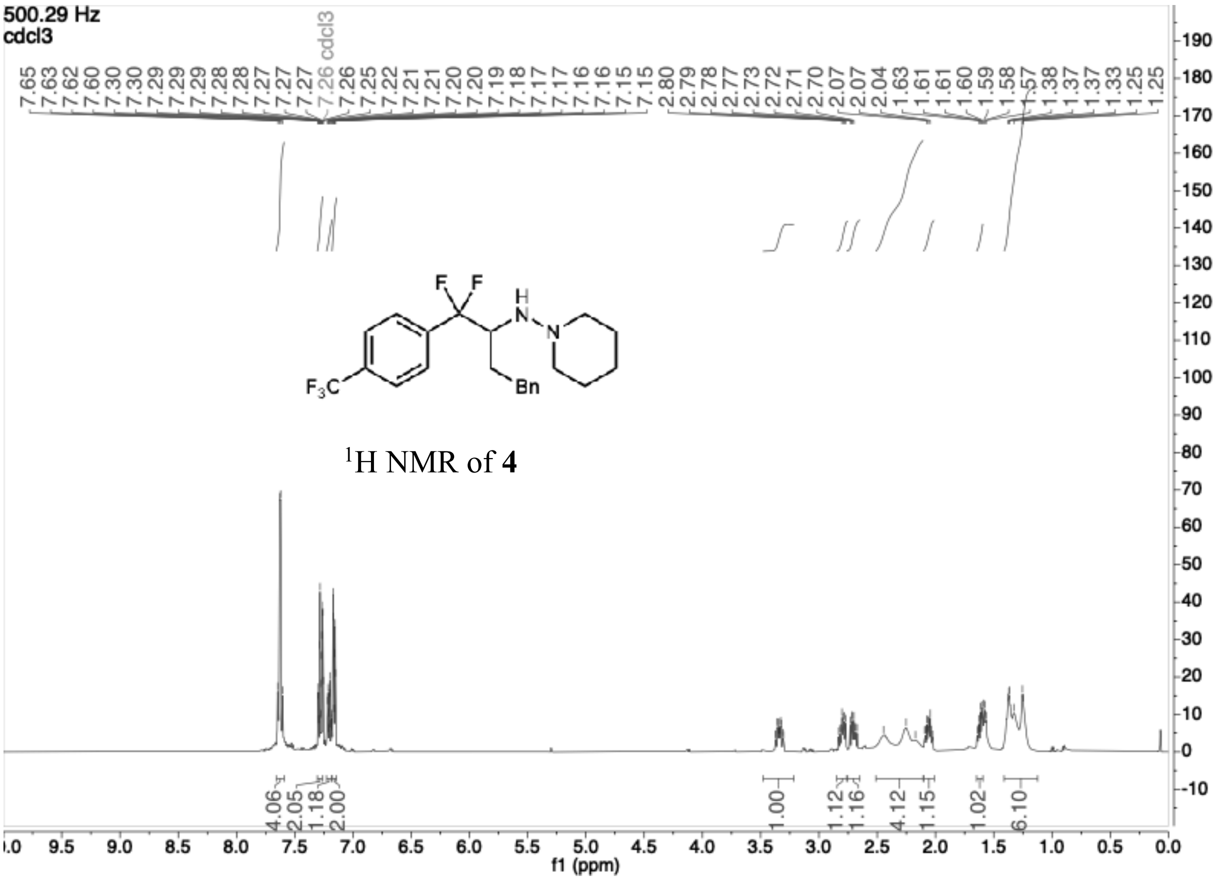

^{1}H NMR of **4**

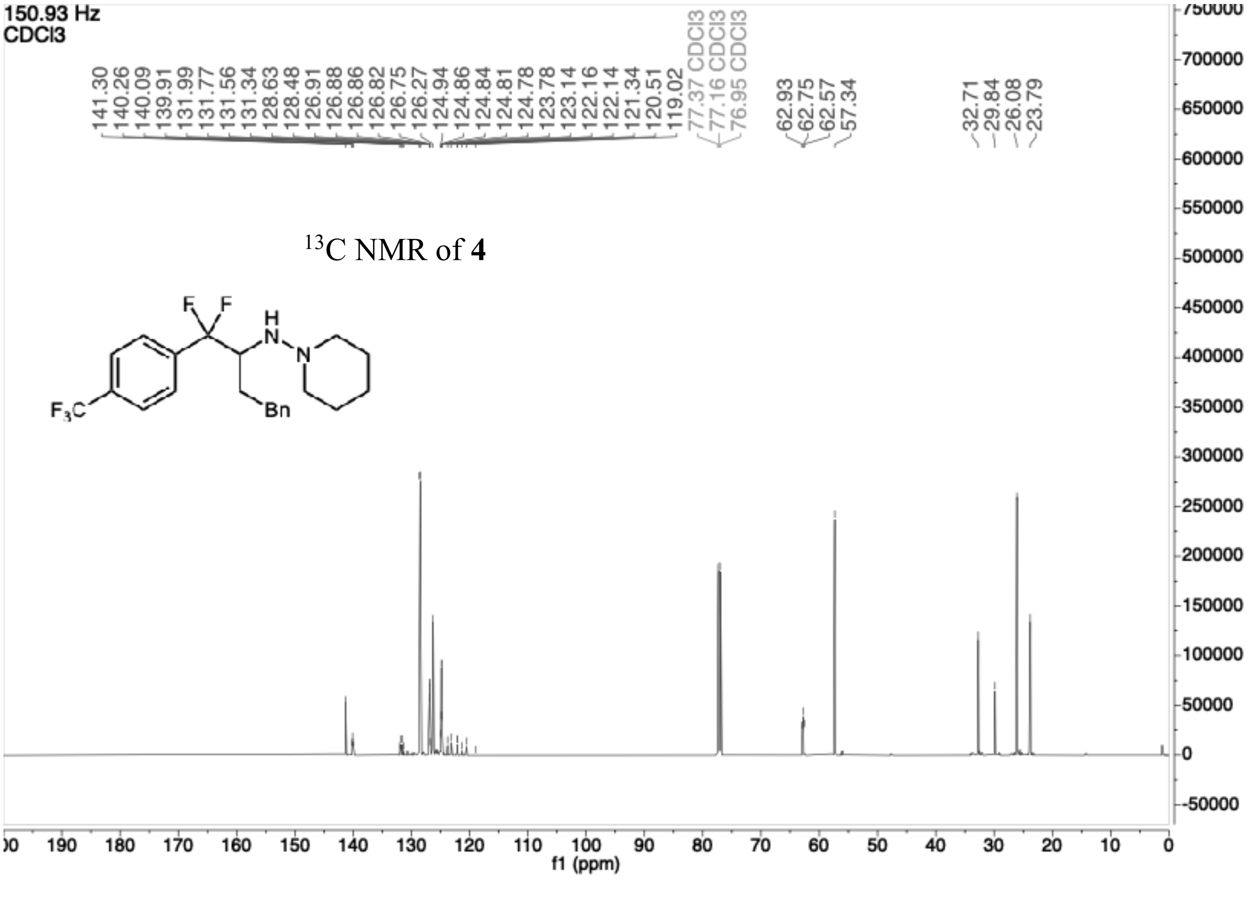

^{13}C NMR of **4**

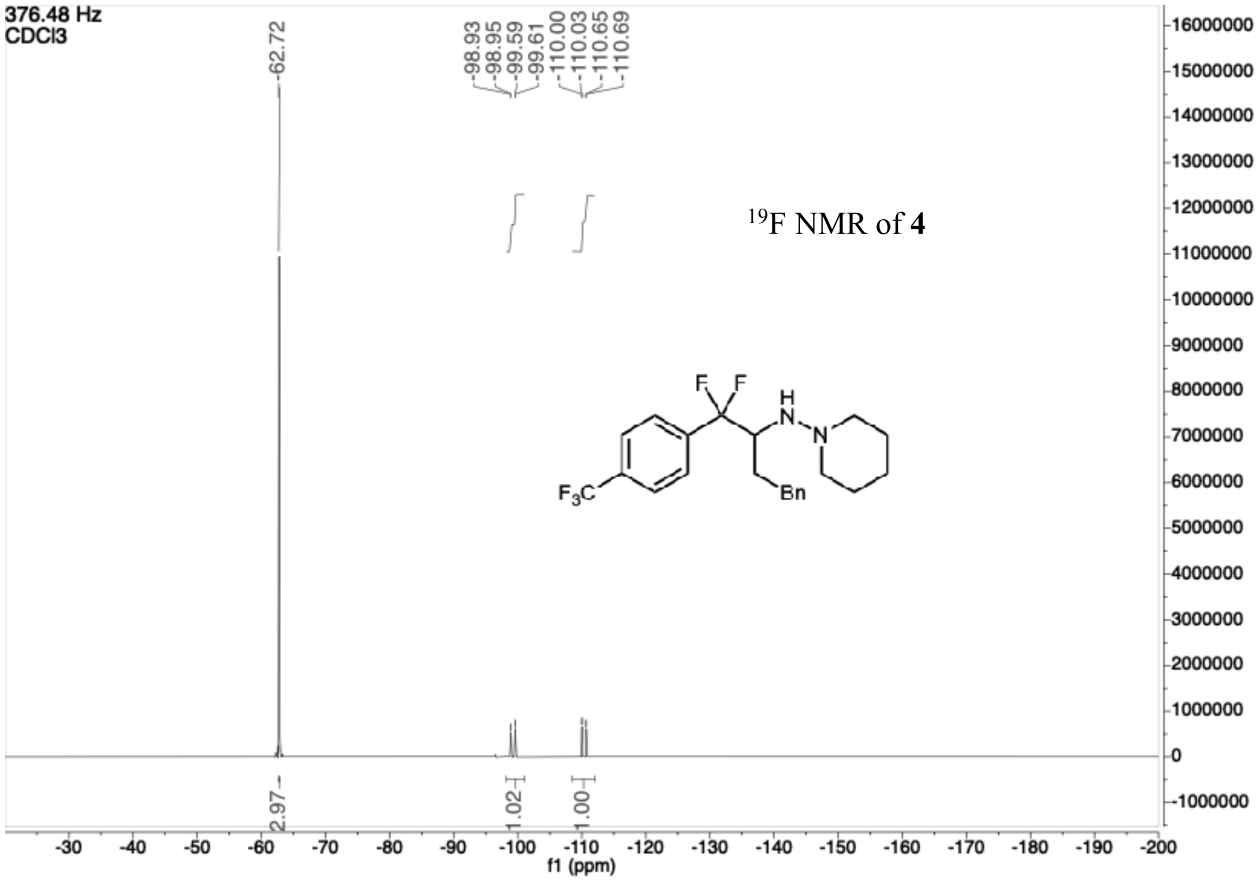
376.48 Hz
CDCl3
-62.72
-98.93
-98.95
-99.59
-99.61
-110.00
-110.03
-110.65
-110.69
19F NMR of 4
F
F
H
N
N
F3C
Bn
2.97
1.02
1.00
-30 -40 -50 -60 -70 -80 -90 -100 -110 -120 -130 -140 -150 -160 -170 -180 -190 -200
f1 (ppm)
16000000
15000000
14000000
13000000
12000000
11000000
10000000
9000000
8000000
7000000
6000000
5000000
4000000
3000000
2000000
1000000
0
-1000000

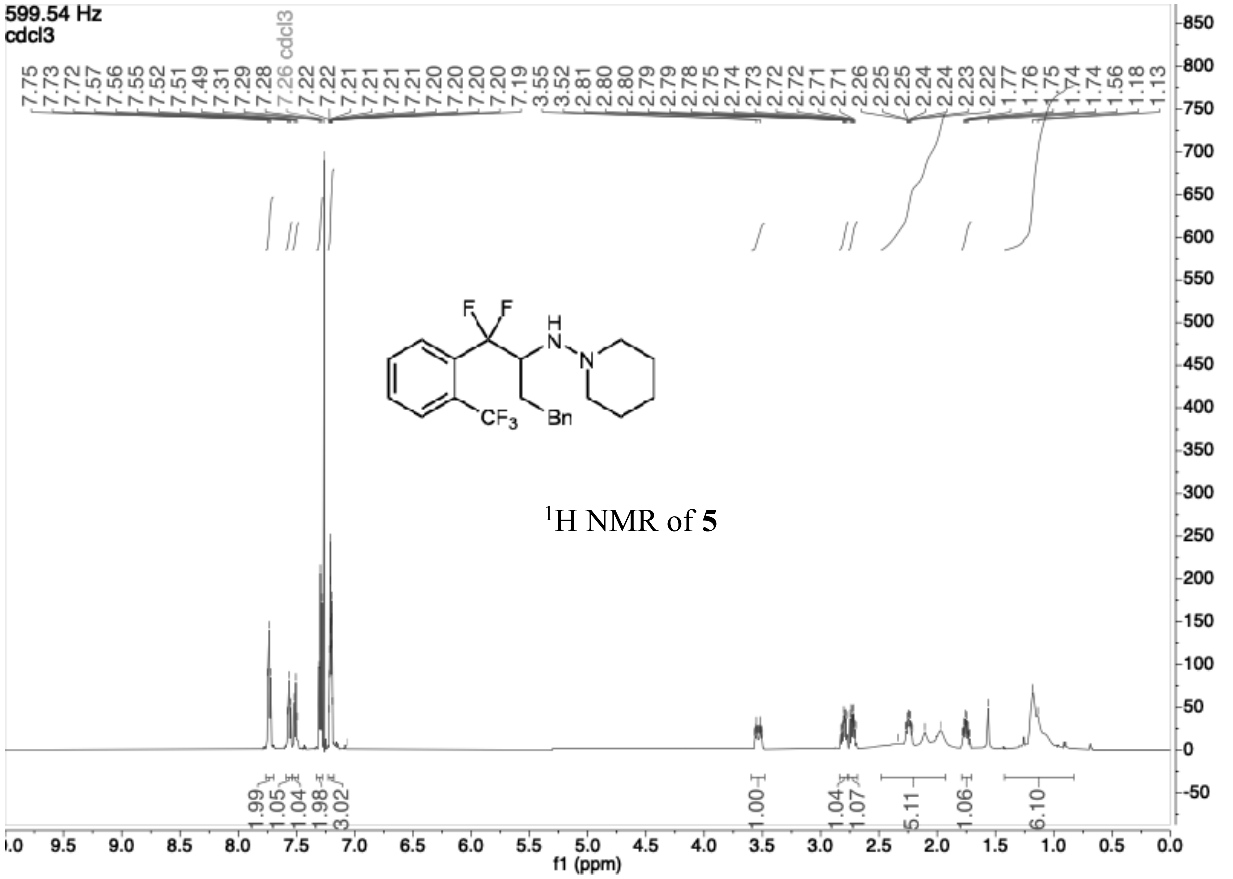

^{1}H NMR of **5**

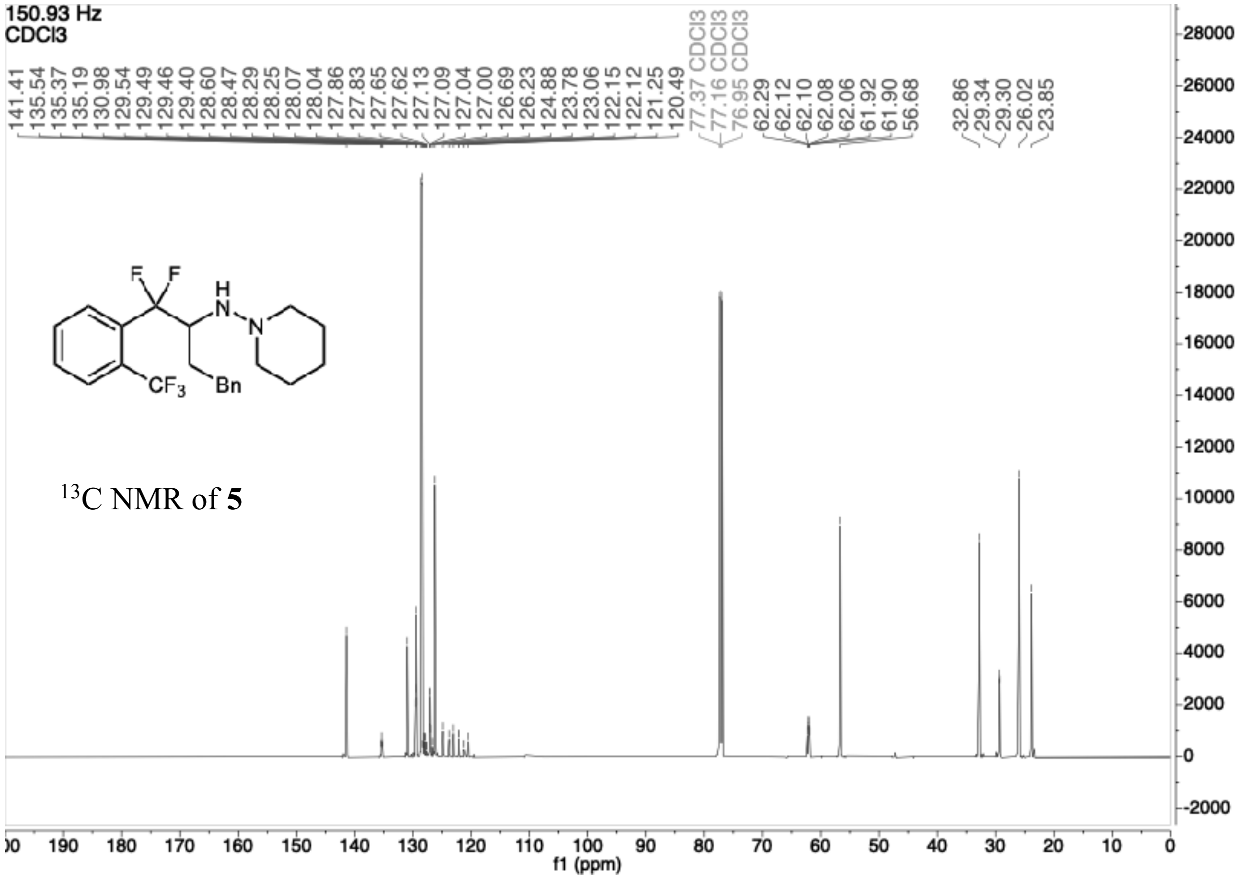

^{13}C NMR of **5**

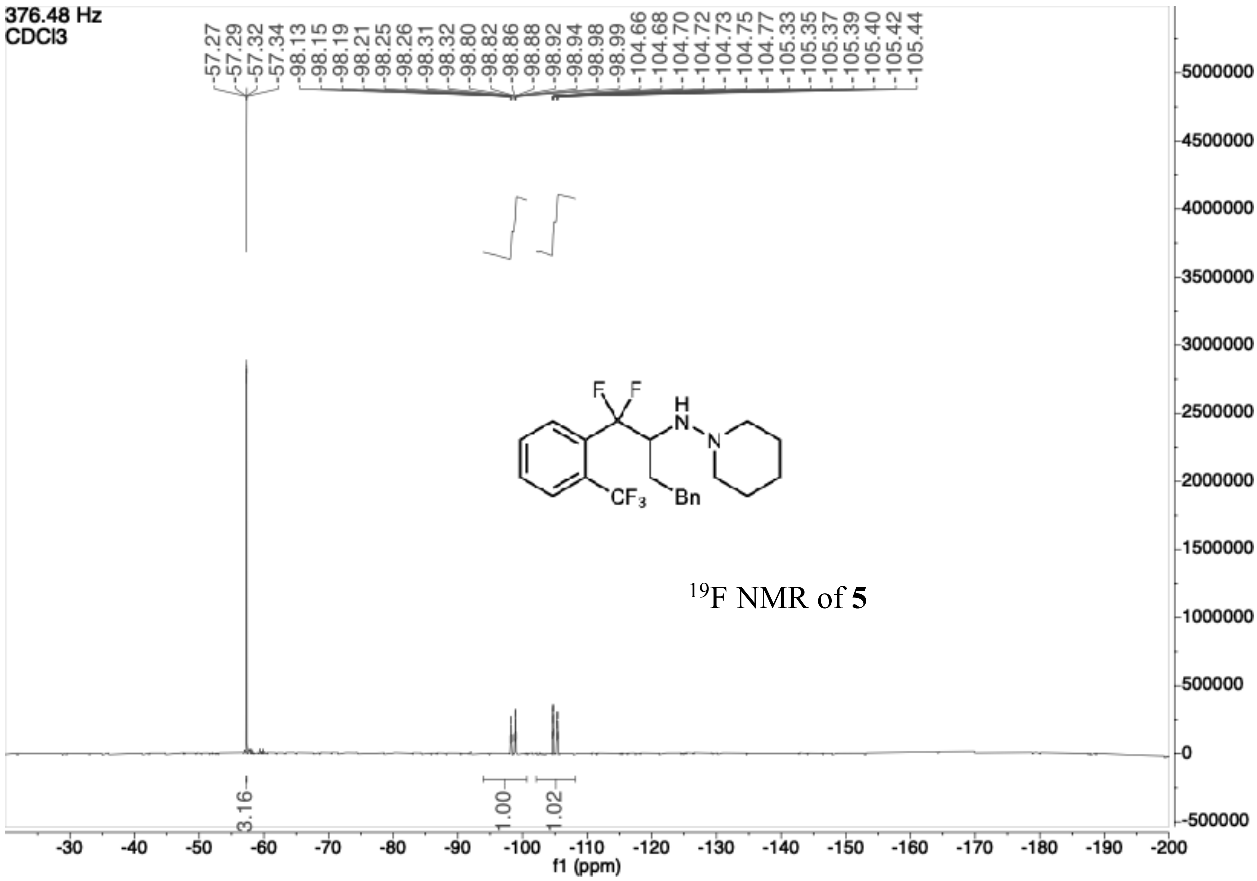

^{19}F NMR of **5**

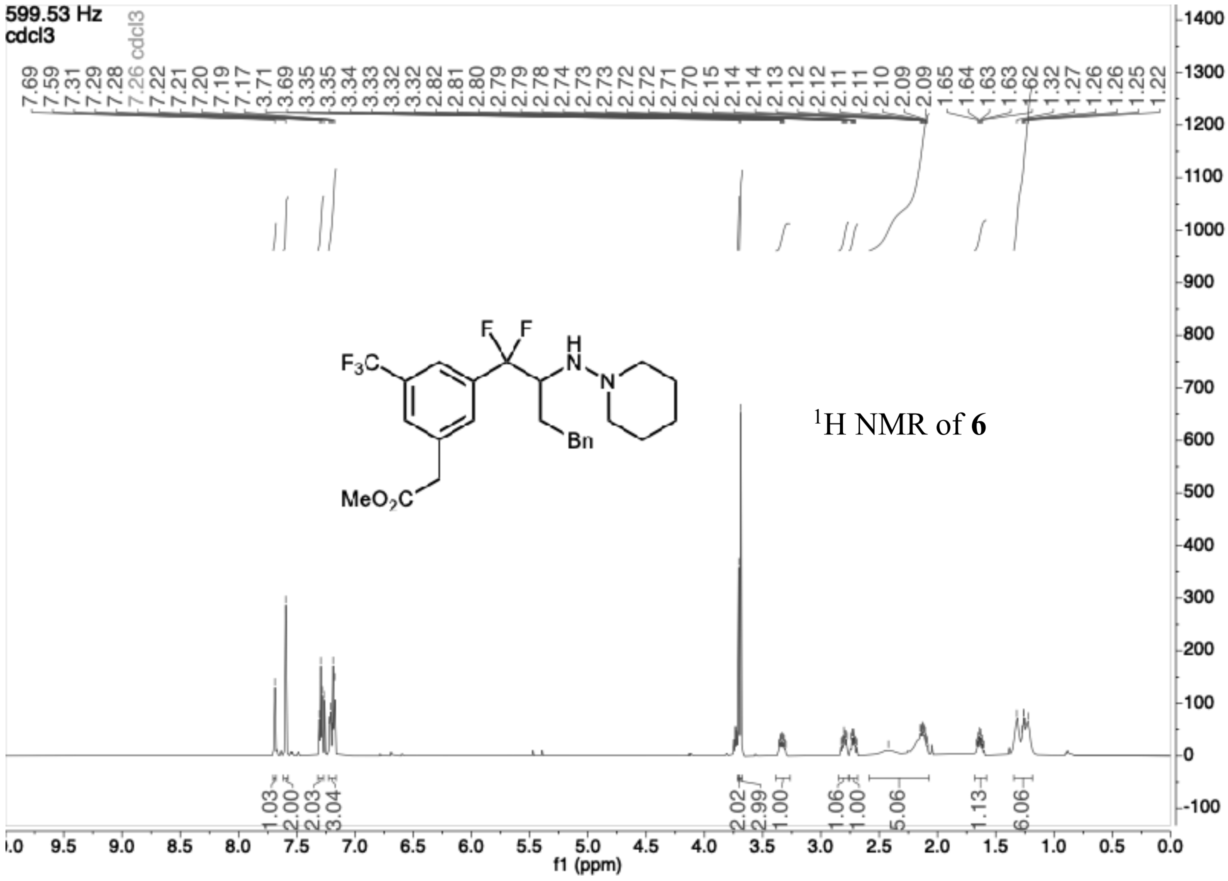
599.53 Hz
cdcl3
^{1}H NMR of **6**
f1 (ppm)

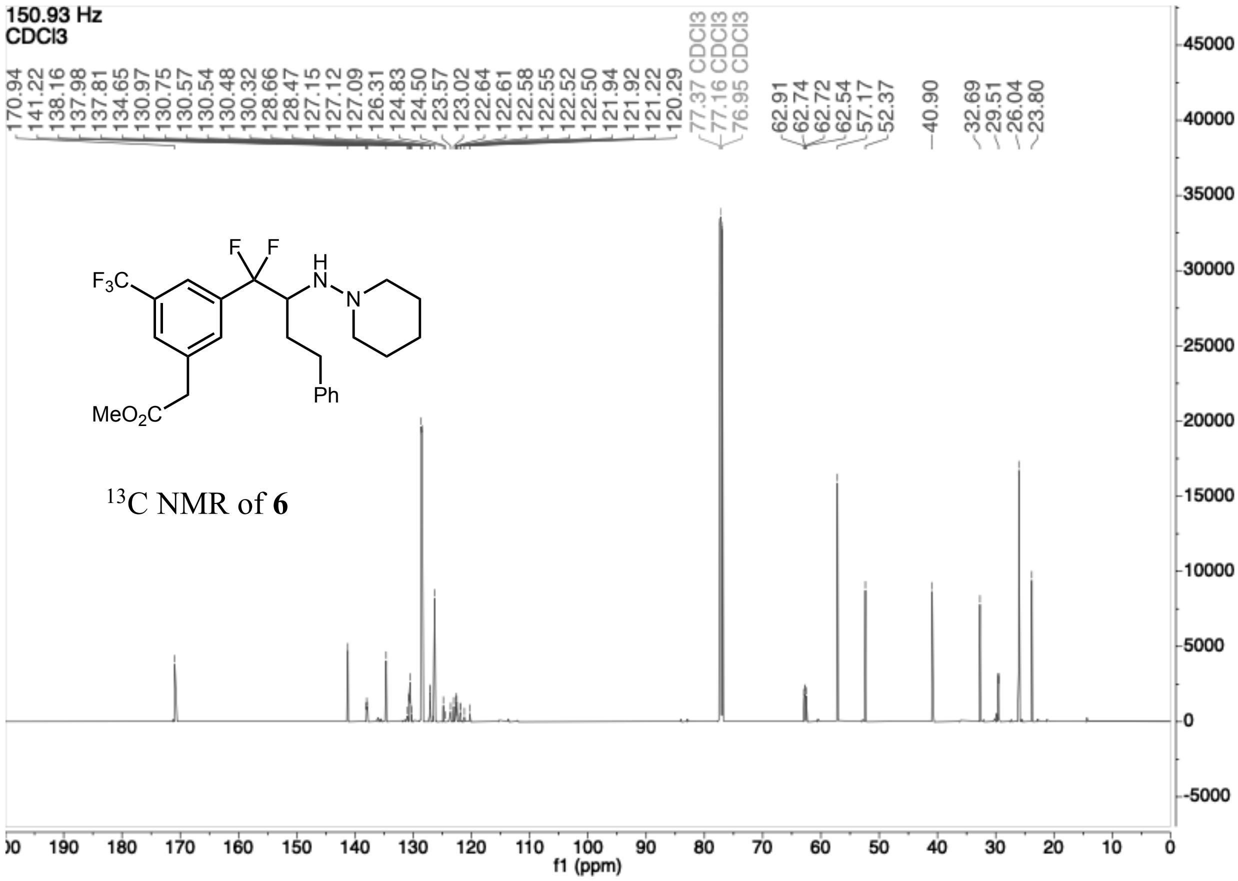
150.93 Hz
CDCl3
^{13}C NMR of **6**
f1 (ppm)

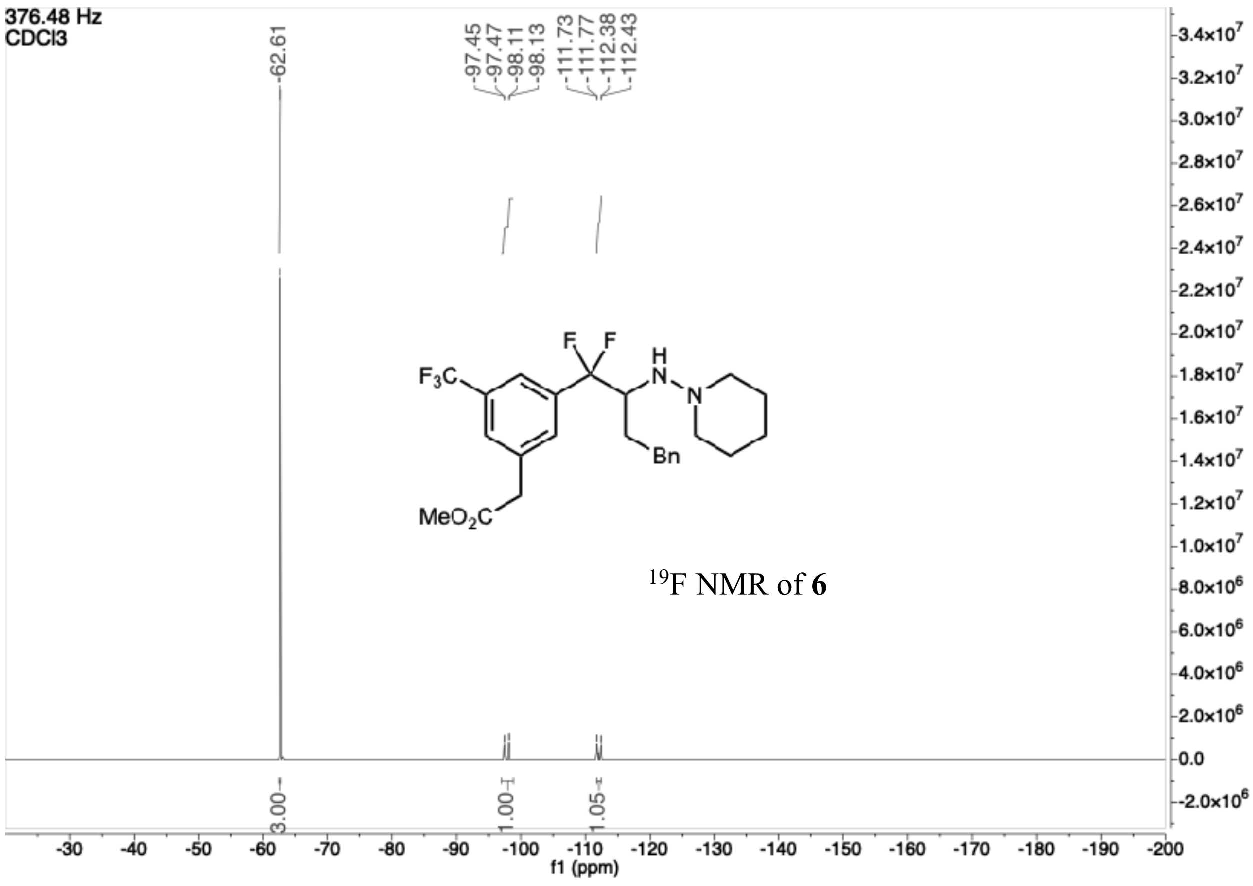

376.48 Hz
CDCl3
-62.61
-97.45
-97.47
-98.11
-98.13
-111.73
-111.77
-112.38
-112.43
F3C
F
F
H
N
N
Bn
MeO2C
3.00
1.00
1.05
f1 (ppm)

^{19}F NMR of **6**

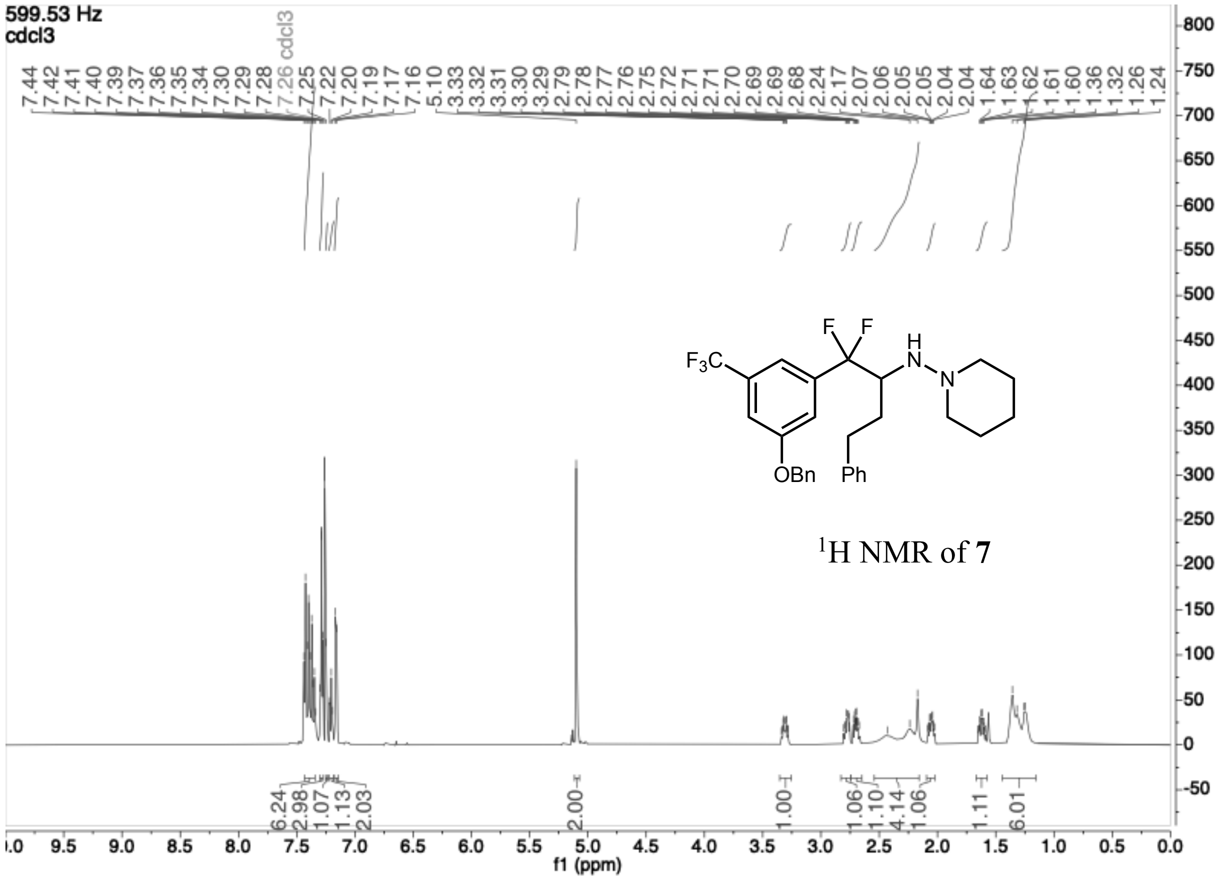
599.53 Hz
cdcl3
7.44 7.42 7.41 7.40 7.39 7.37 7.36 7.35 7.34 7.30 7.29 7.28 7.26 cdcl3 7.25 7.22 7.20 7.19 7.17 7.16 5.10 3.33 3.32 3.31 3.30 3.29 2.79 2.78 2.77 2.76 2.75 2.72 2.71 2.70 2.70 2.69 2.69 2.68 2.24 2.17 2.07 2.06 2.05 2.05 2.04 2.04 1.64 1.63 1.62 1.61 1.60 1.36 1.32 1.26 1.24
F
F
H
N
N
F_3C
OBn
Ph
^{1}H NMR of **7**
6.24 2.98 1.07 1.13 2.03 2.00 1.00 1.06 1.10 4.14 1.06 1.11 6.01
f1 (ppm)

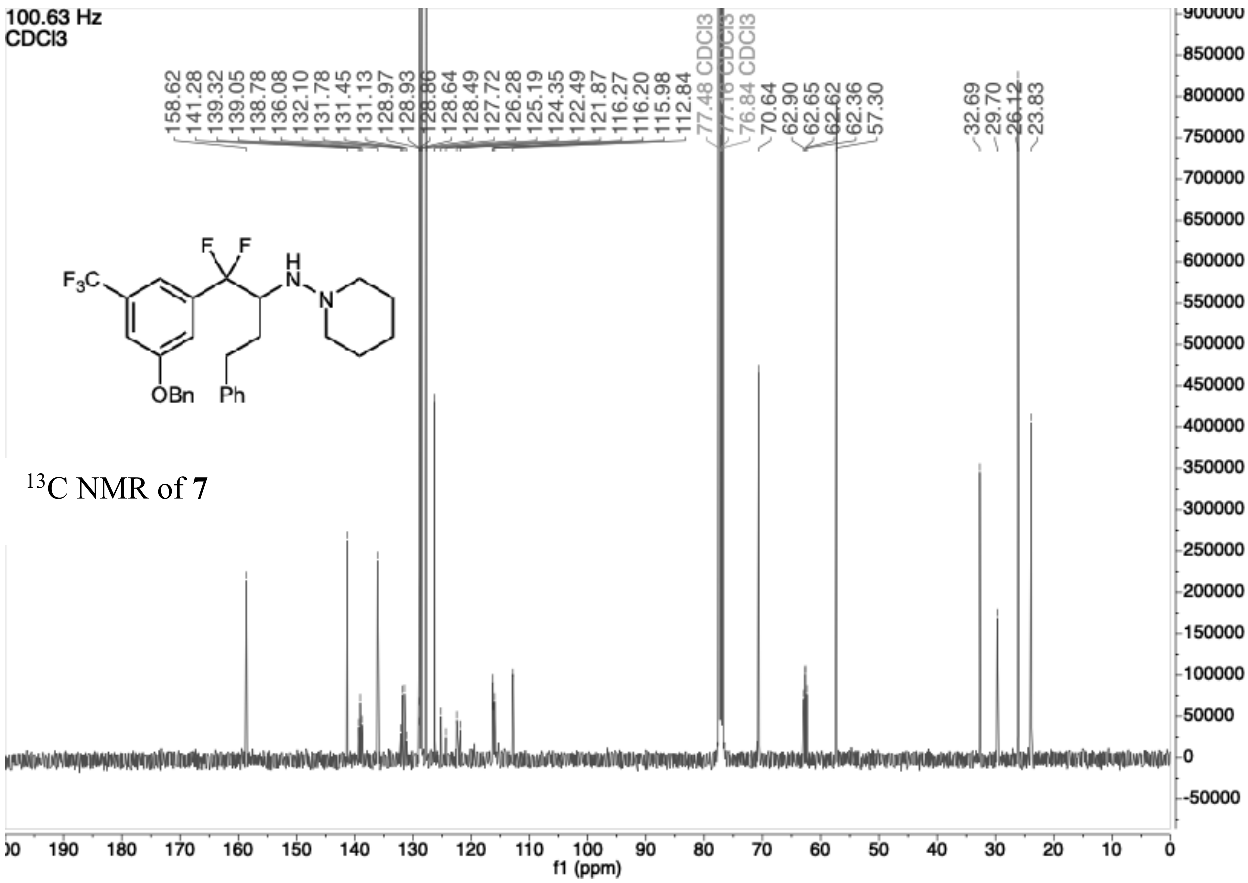
100.63 Hz
CDCl3
158.62 141.28 139.32 139.05 138.78 136.08 132.10 131.78 131.45 131.13 128.97 128.93 128.86 128.64 128.49 127.72 126.28 125.19 124.35 122.49 121.87 116.27 116.20 115.98 112.84 77.48 CDCl3 77.16 CDCl3 76.84 CDCl3 70.64 62.90 62.65 62.62 62.36 57.30 32.69 29.70 26.12 23.83
F
F
H
N
N
F_3C
OBn
Ph
^{13}C NMR of **7**
f1 (ppm)

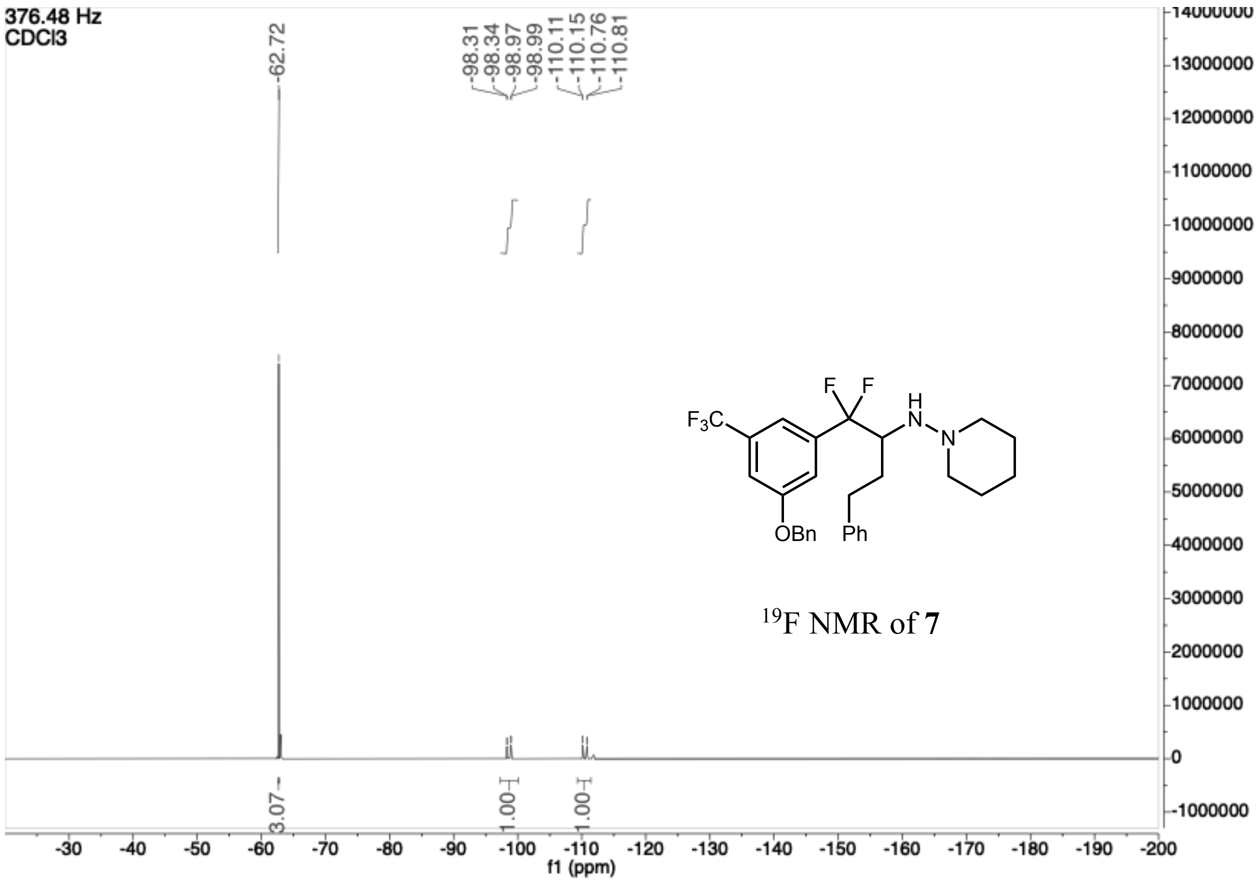
376.48 Hz
CDCl3
-62.72
-98.31
-98.34
-98.97
-98.99
-110.11
-110.15
-110.76
-110.81
3.07
1.00
1.00
f1 (ppm)
F$_3$C
F
F
H
N
N
OBn
Ph

^{19}F NMR of **7**

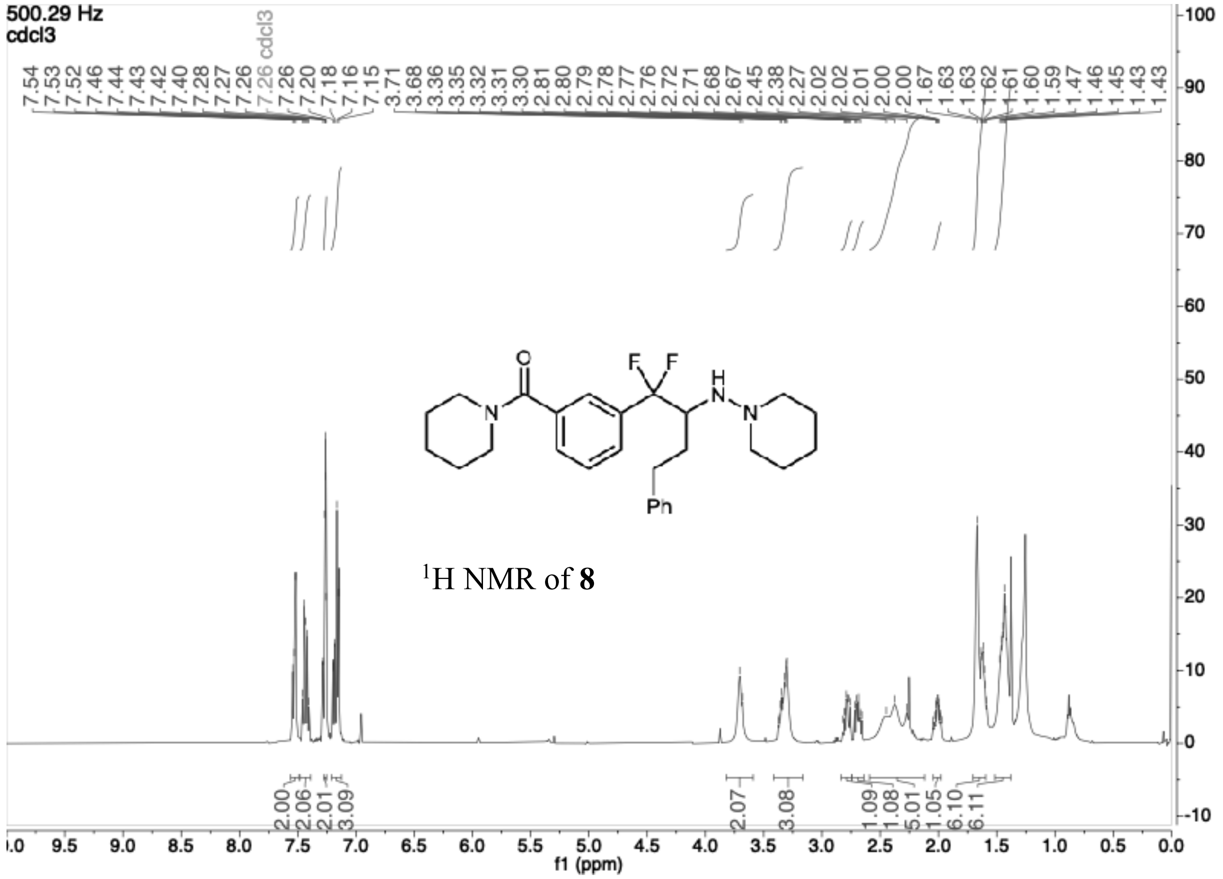

^{1}H NMR of **8**

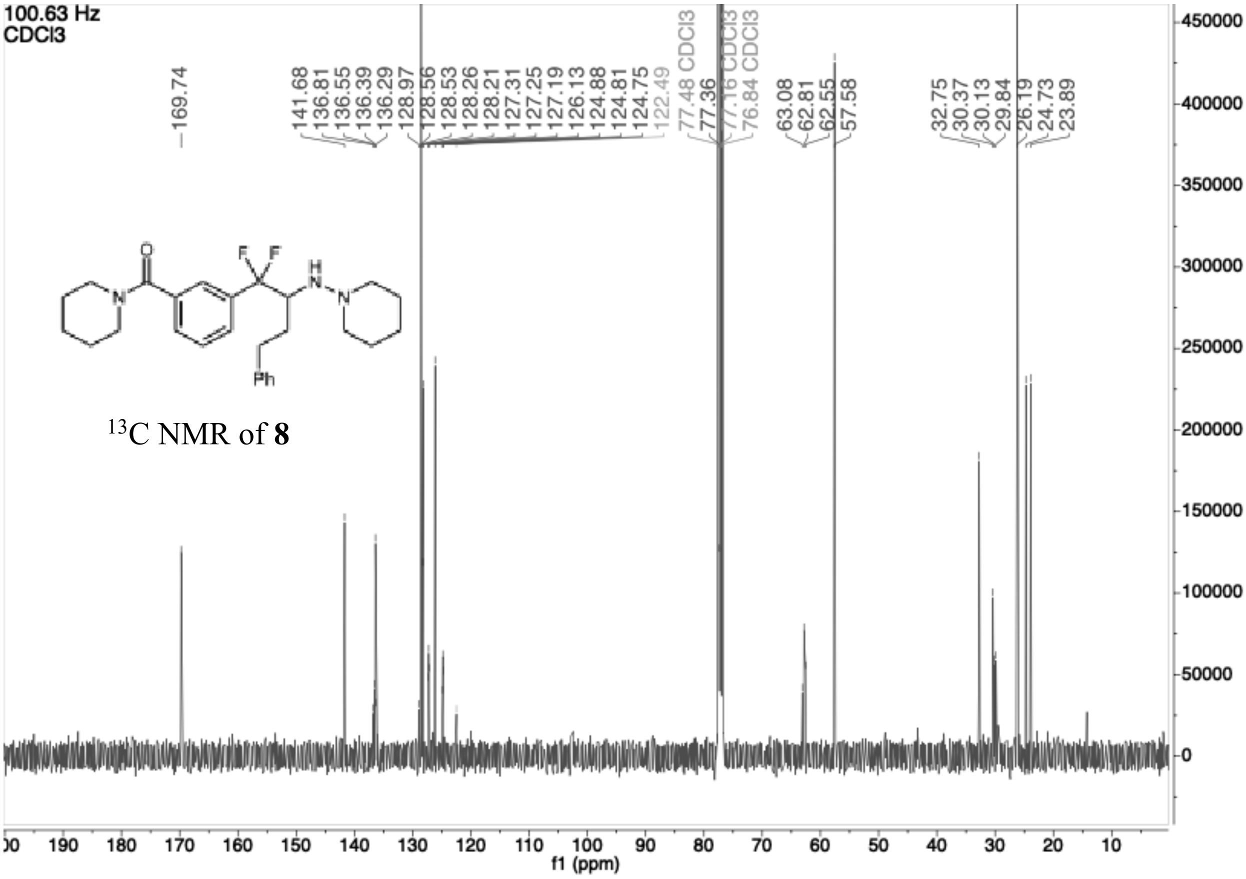

^{13}C NMR of **8**

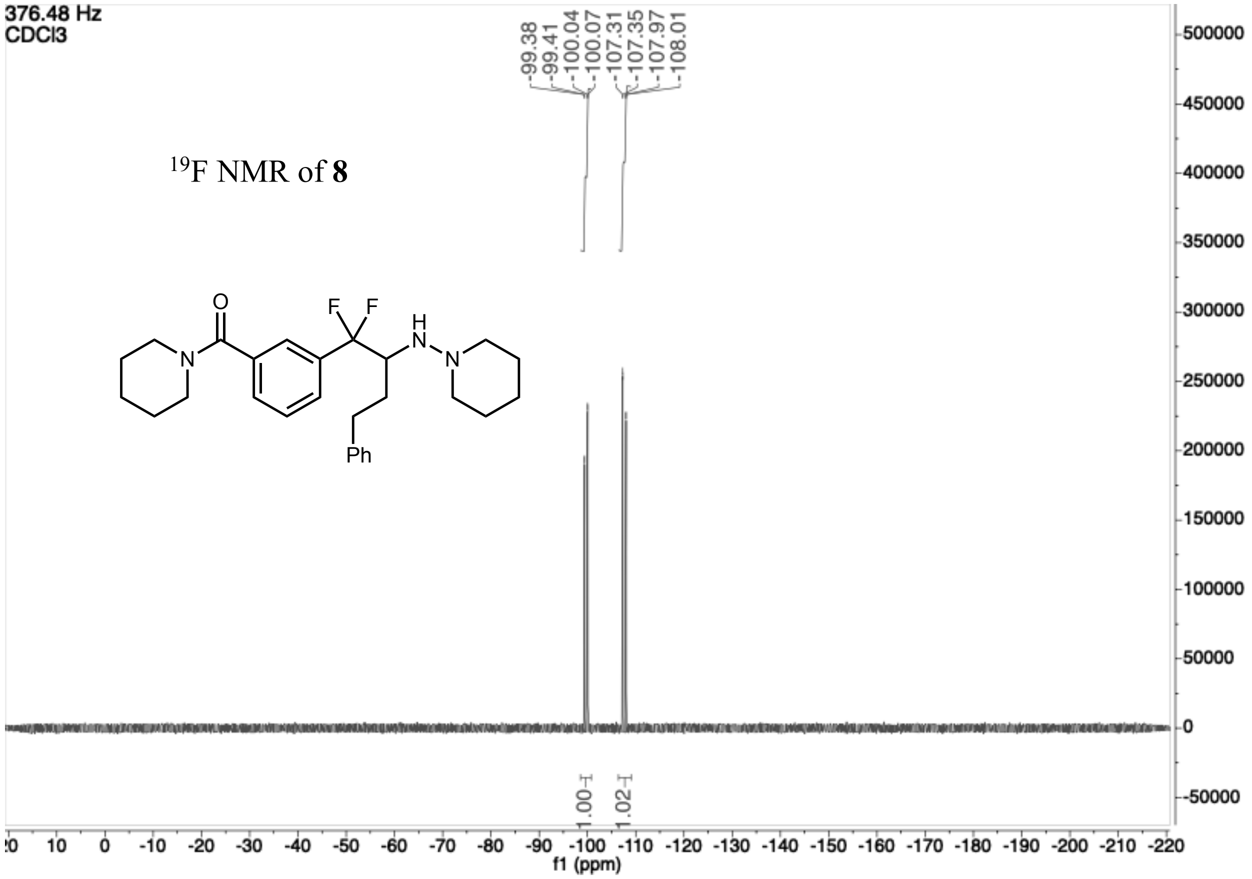

376.48 Hz
CDCl3
-99.38
-99.41
-100.04
-100.07
-107.31
-107.35
-107.97
-108.01
19F NMR of 8
1.00
1.02
f1 (ppm)

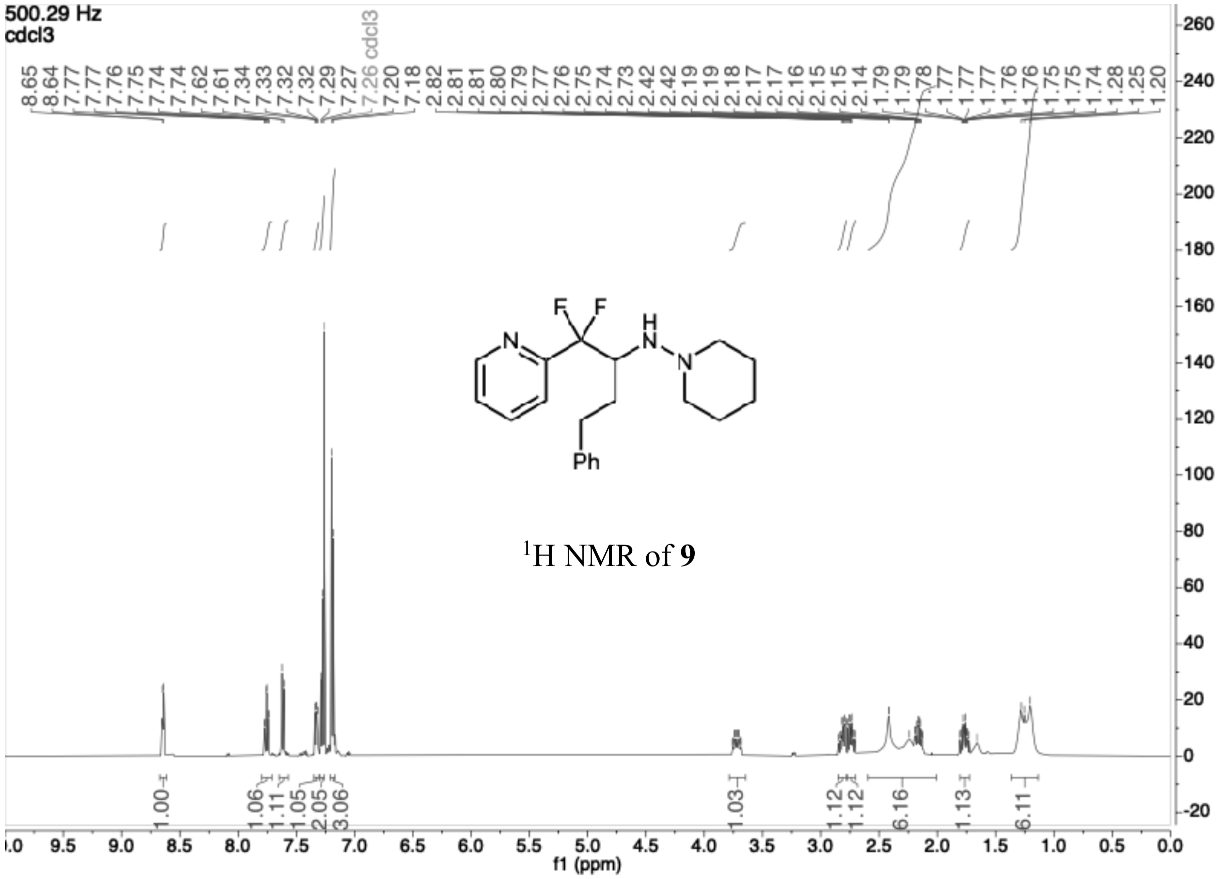
500.29 Hz
cdcl3
^{1}H NMR of **9**
f1 (ppm)

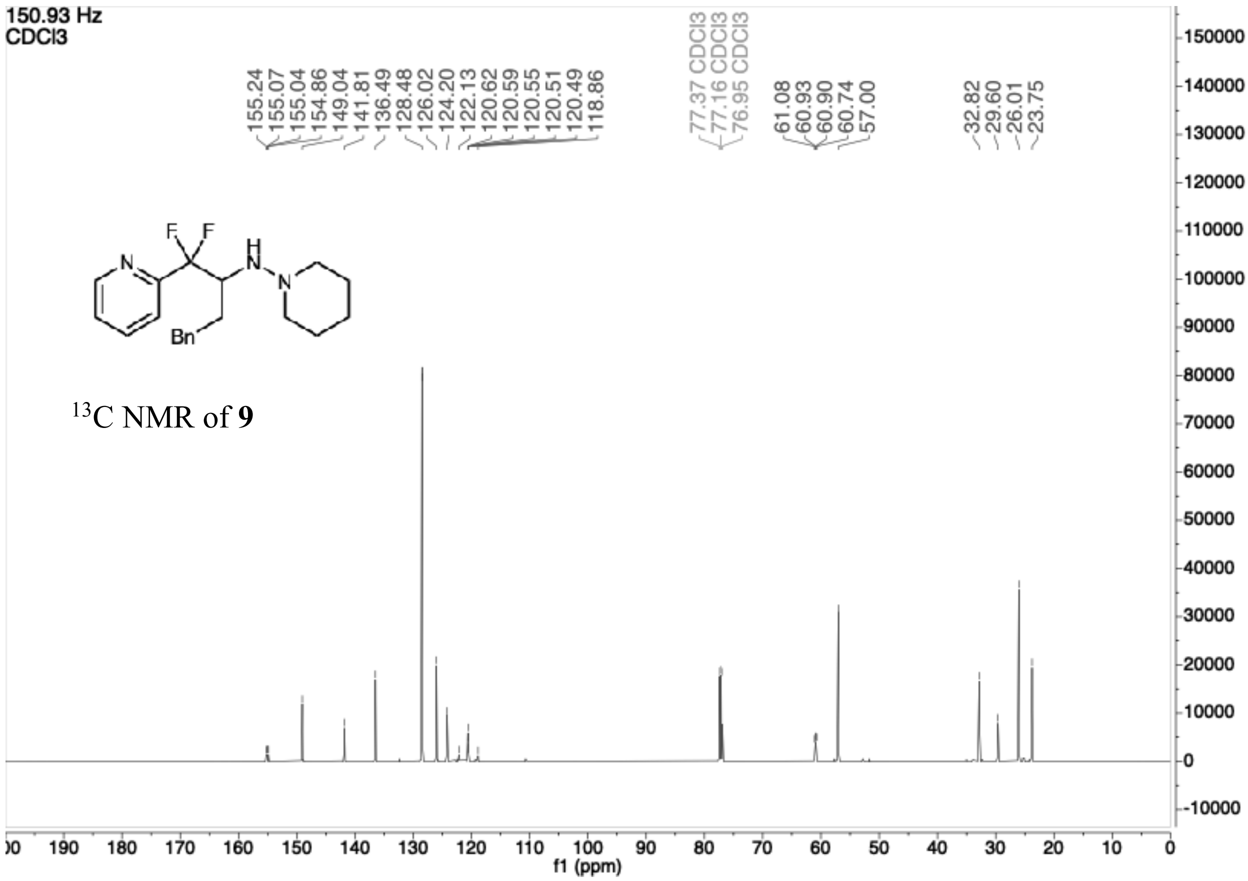
150.93 Hz
CDCl3
^{13}C NMR of **9**
f1 (ppm)

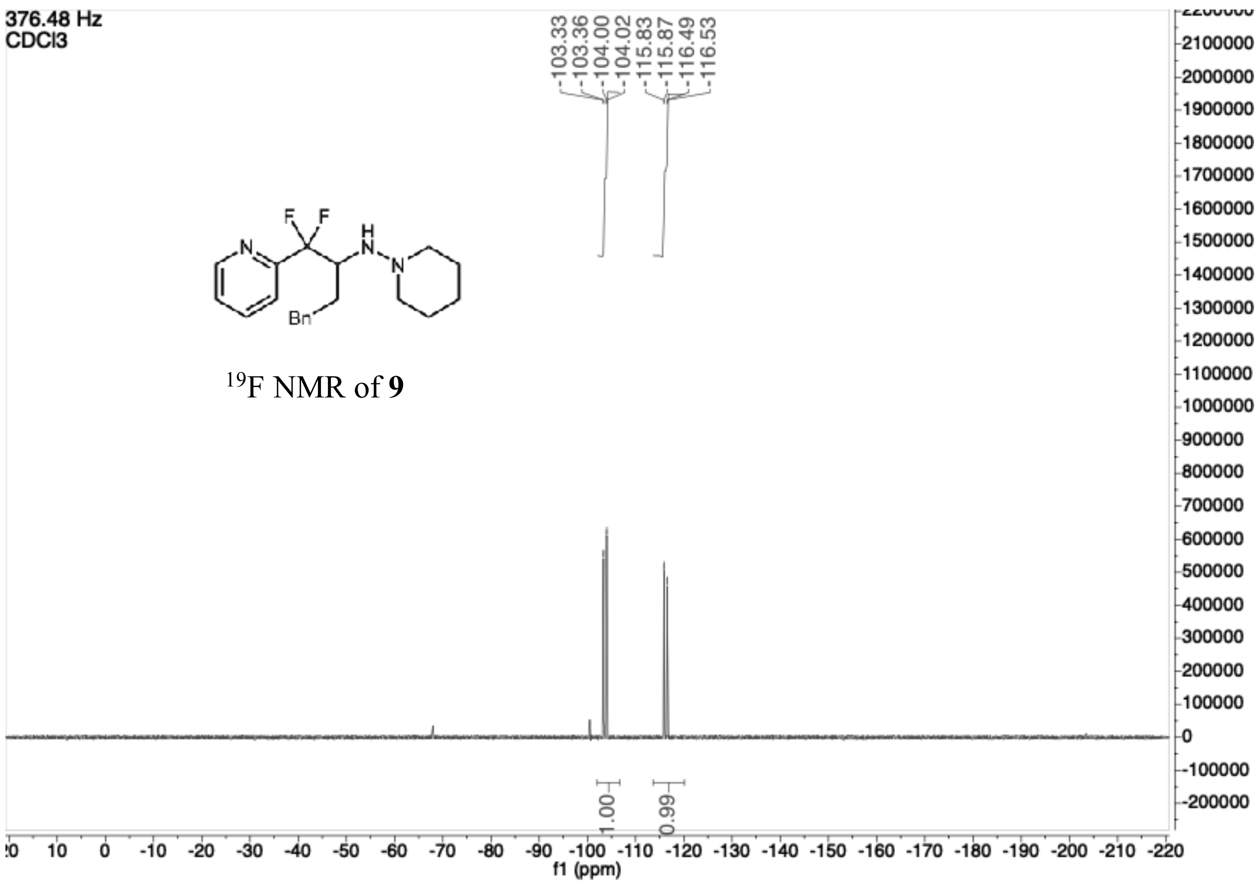
376.48 Hz
CDCl3
-103.33
-103.36
-104.00
-104.02
-115.83
-115.87
-116.49
-116.53
Bn
^{19}F NMR of **9**
1.00
0.99
f1 (ppm)

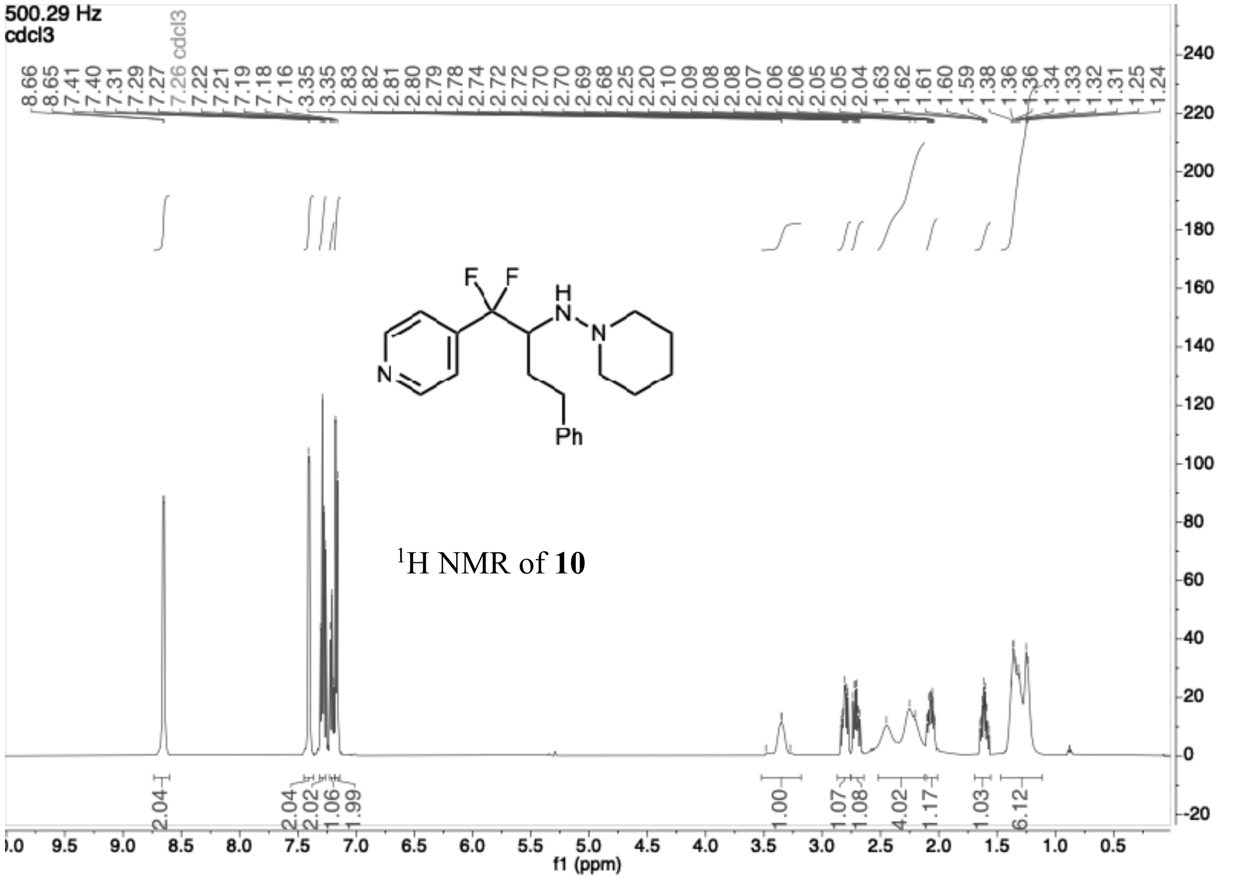
500.29 Hz
cdcl3
^{1}H NMR of **10**
f1 (ppm)

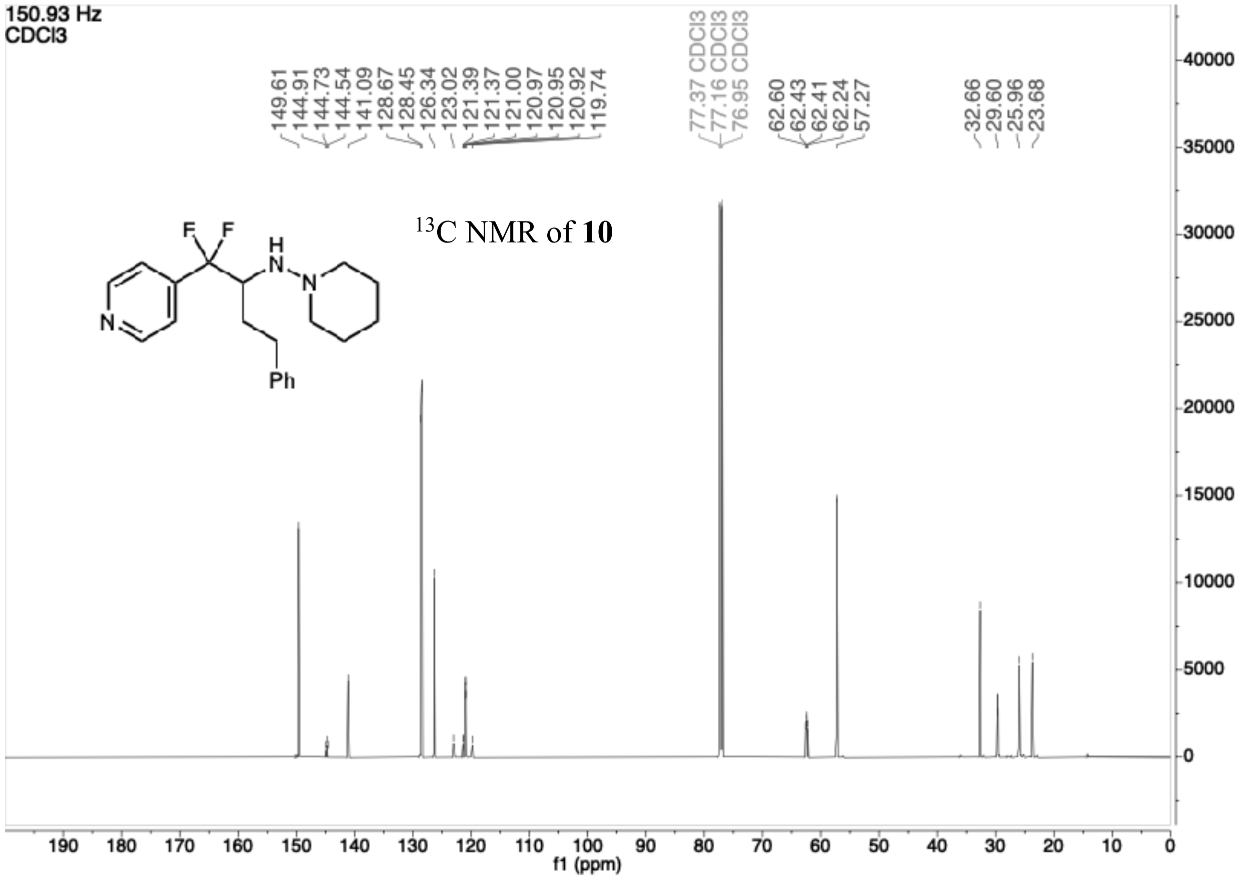
150.93 Hz
CDCl3
^{13}C NMR of **10**
f1 (ppm)

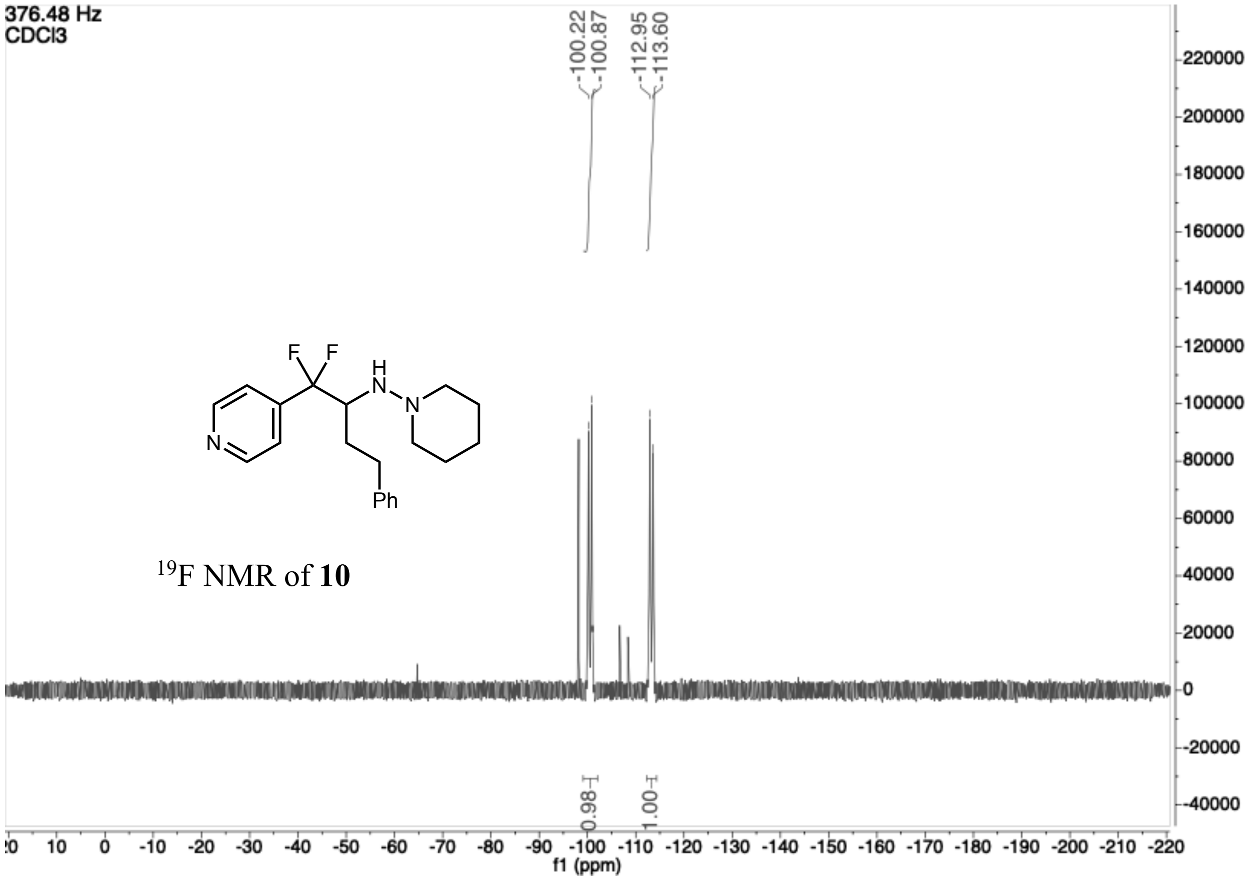

^{19}F NMR of **10**

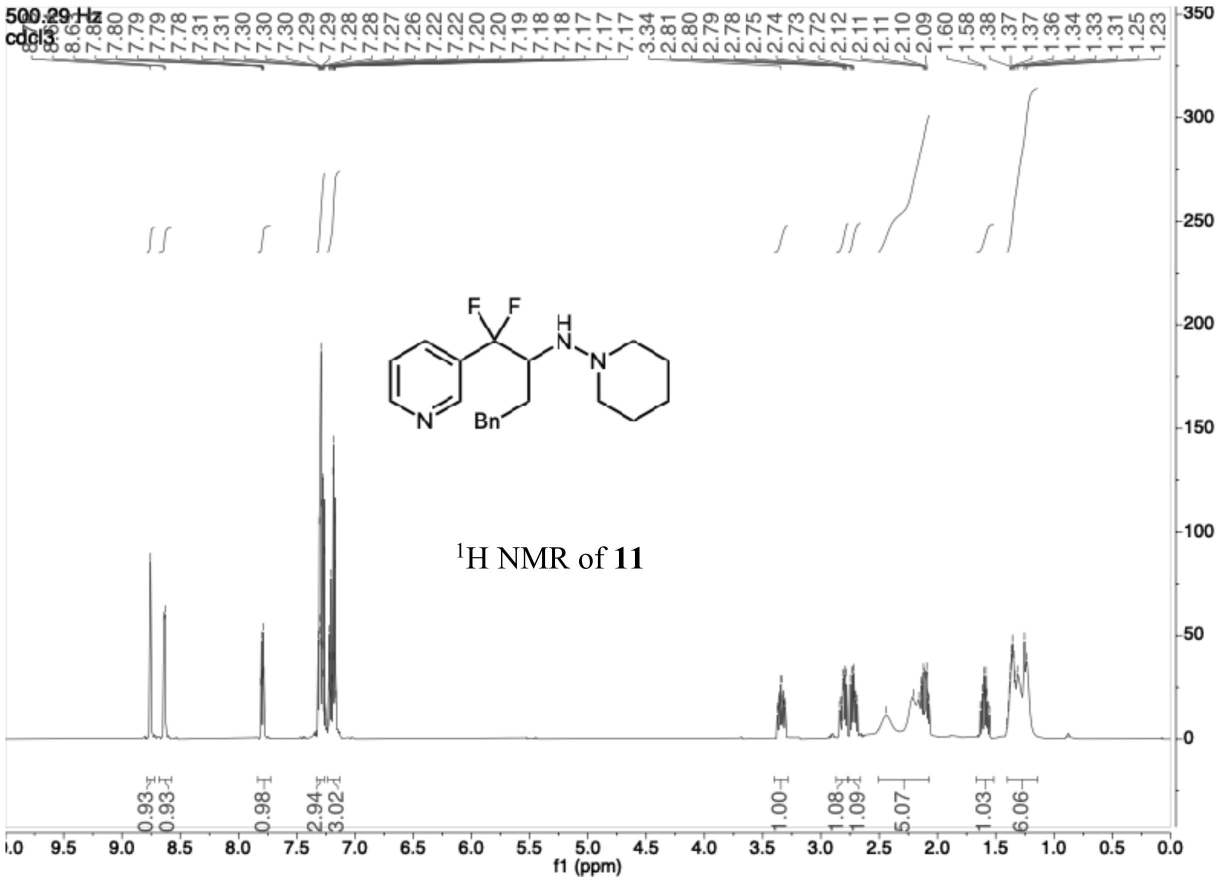

¹H NMR of **11**

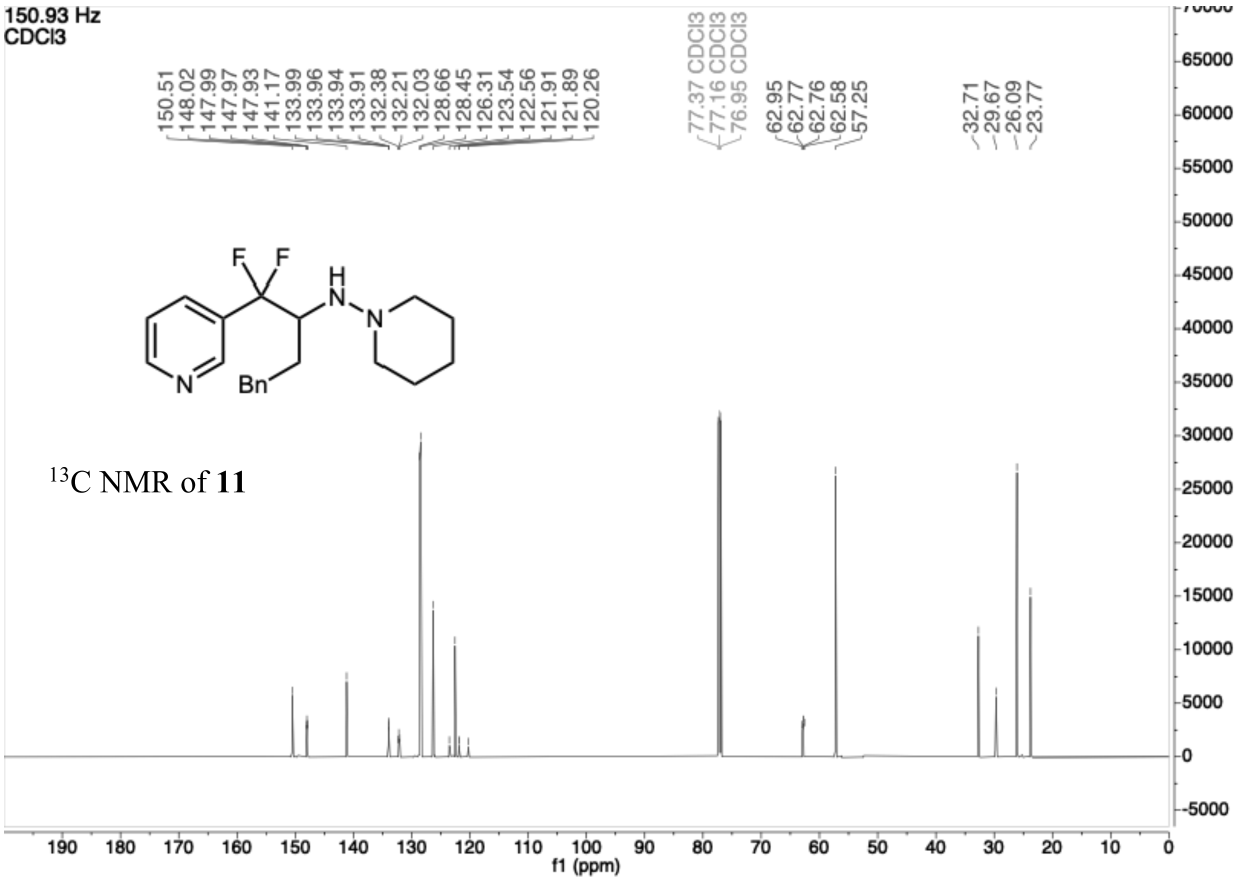

¹³C NMR of **11**

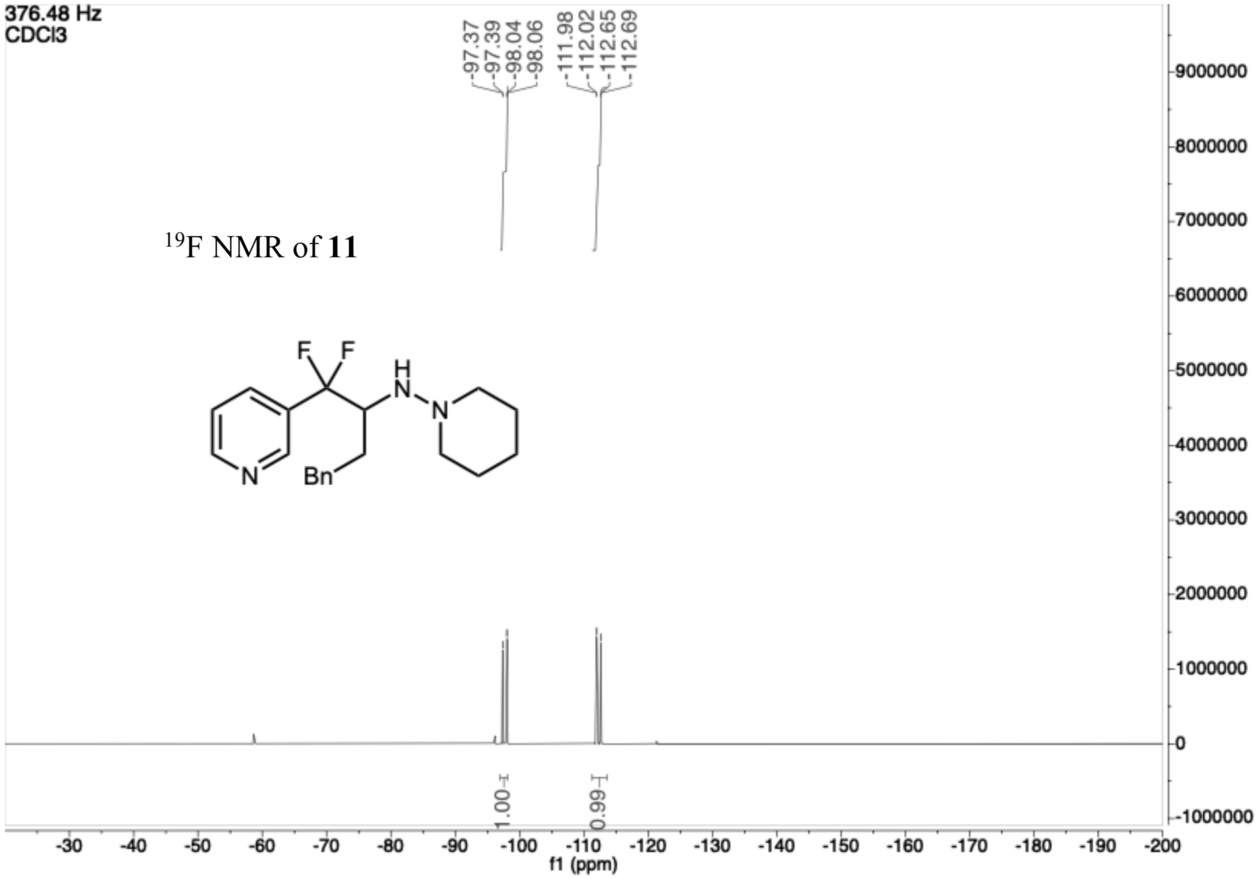
376.48 Hz
CDCl3
19F NMR of 11
-97.37
-97.39
-98.04
-98.06
-111.98
-112.02
-112.65
-112.69
1.00
0.99
f1 (ppm)
F
F
H
N
N
N
Bn

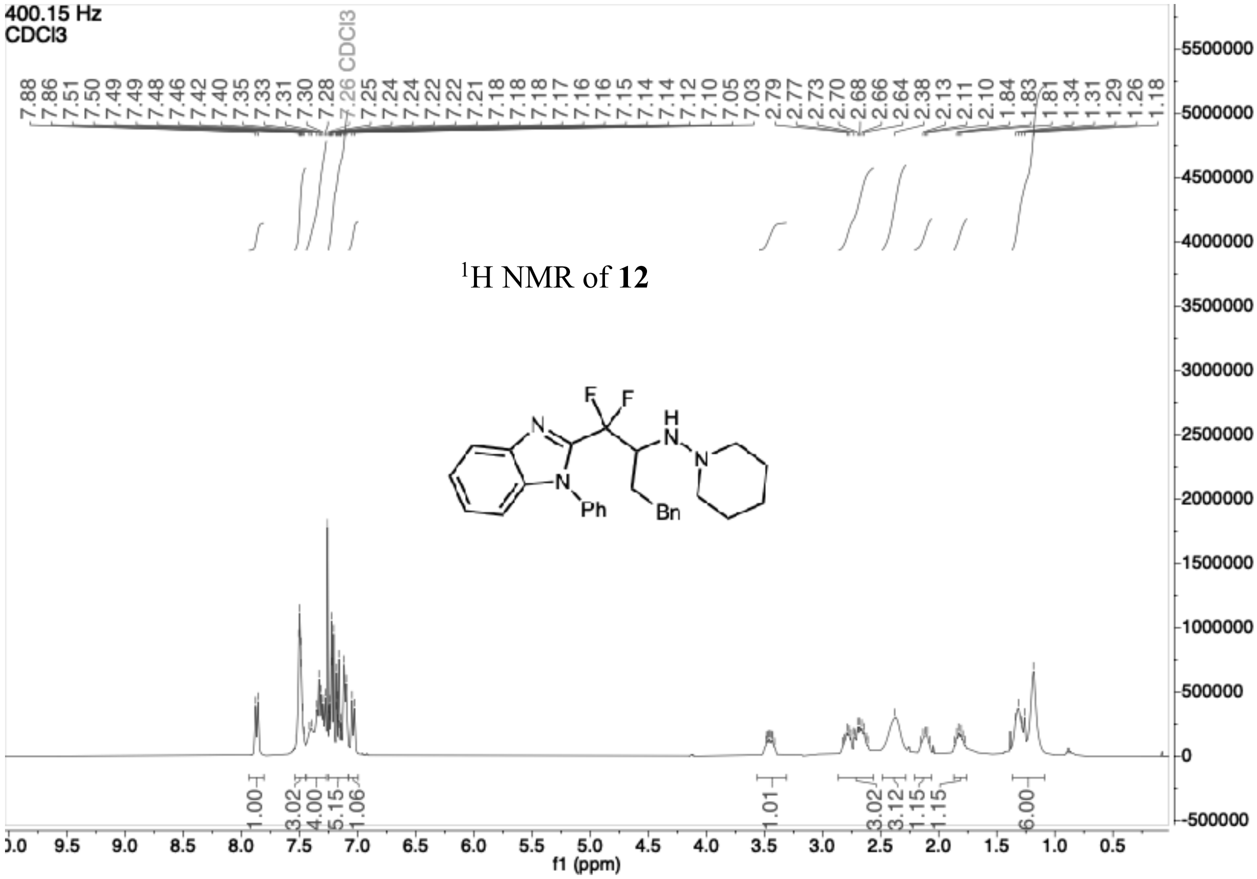
400.15 Hz
CDCl3
7.88 7.86 7.51 7.50 7.49 7.49 7.48 7.46 7.42 7.40 7.35 7.33 7.31 7.30 7.28 7.26 CDCl3 7.25 7.24 7.24 7.22 7.22 7.21 7.18 7.18 7.18 7.17 7.16 7.16 7.15 7.14 7.14 7.12 7.10 7.05 7.03 2.79 2.77 2.73 2.70 2.68 2.66 2.64 2.38 2.13 2.11 2.10 1.84 1.83 1.81 1.34 1.31 1.29 1.26 1.18
1H NMR of 12
1.00 3.02 4.00 5.15 1.06 1.01 3.02 3.12 1.15 1.15 6.00
f1 (ppm)
F F H N N N Ph Bn

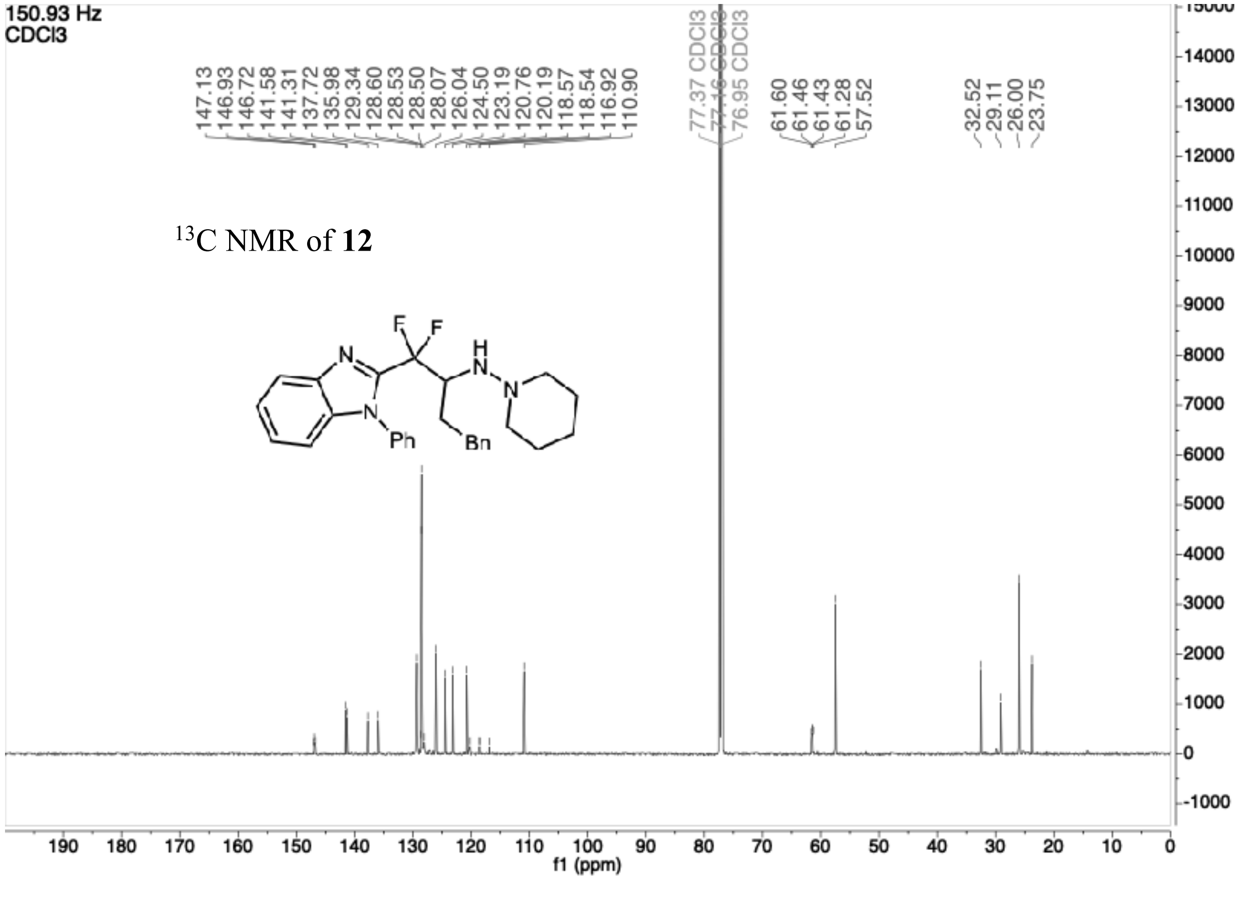
150.93 Hz
CDCl3
147.13 146.93 146.72 141.58 141.31 137.72 135.98 129.34 128.60 128.53 128.50 128.07 126.04 124.50 123.19 120.76 120.19 118.57 118.54 116.92 110.90 77.37 CDCl3 77.16 CDCl3 76.95 CDCl3 61.60 61.46 61.43 61.28 57.52 32.52 29.11 26.00 23.75
13C NMR of 12
f1 (ppm)
F F H N N N Ph Bn

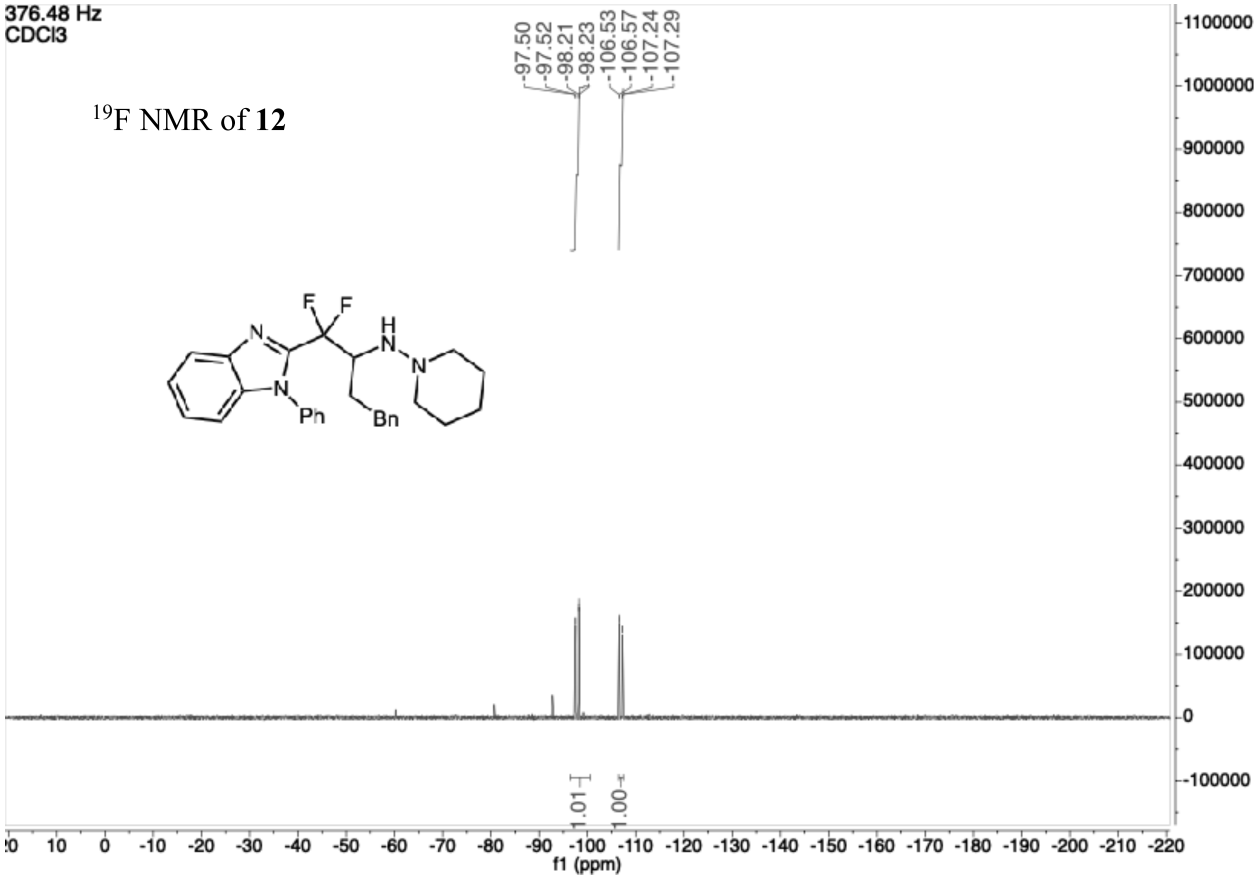
376.48 Hz
CDCl3
-97.50
-97.52
-98.21
-98.23
-106.53
-106.57
-107.24
-107.29
^{19}F NMR of **12**
N
F
F
H
N
N
N
Ph
Bn
1.01
1.00
f1 (ppm)

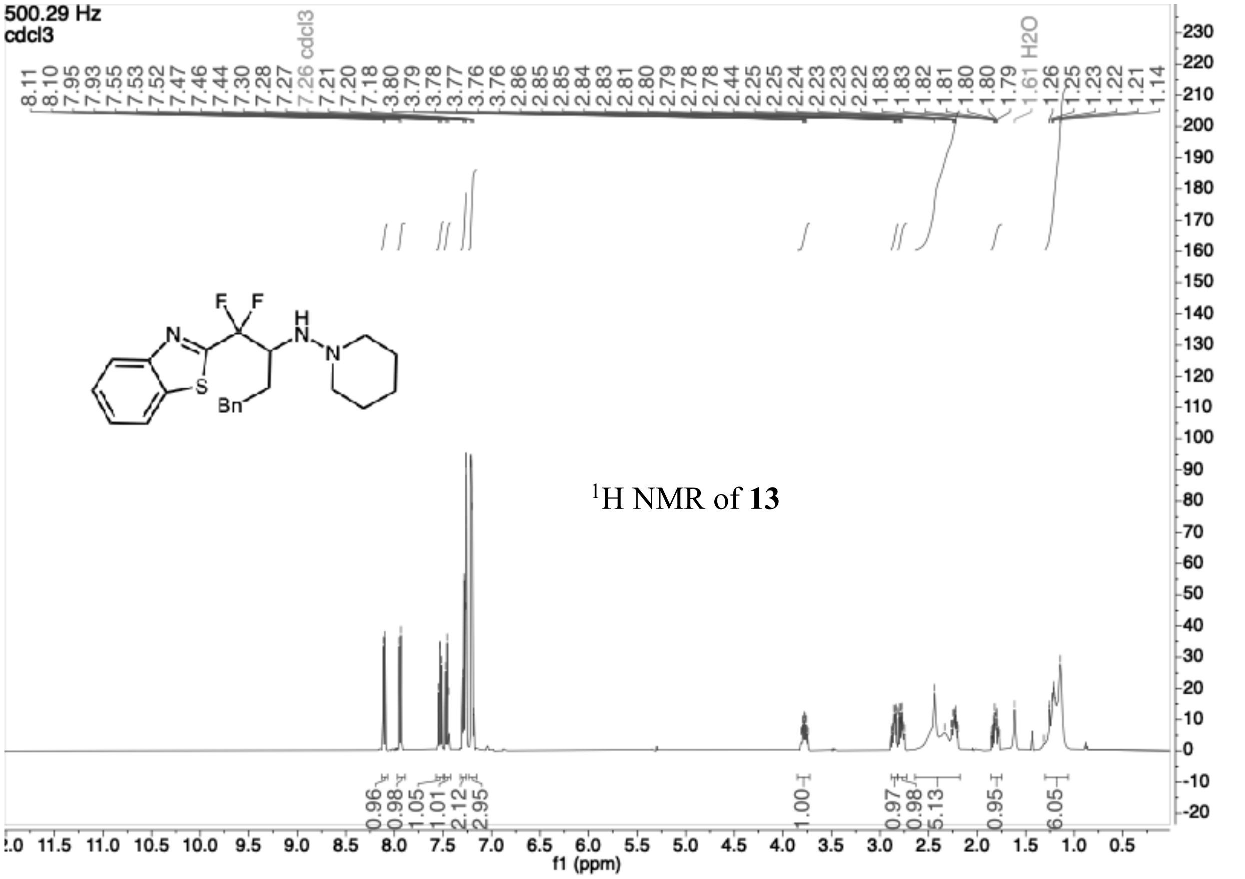
500.29 Hz
cdcl3
8.11 8.10 7.95 7.93 7.55 7.53 7.52 7.47 7.46 7.44 7.30 7.28 7.27 7.26 cdcl3 7.21 7.20 7.18 3.80 3.79 3.78 3.77 3.76 3.76 2.86 2.85 2.85 2.84 2.83 2.81 2.80 2.79 2.78 2.78 2.44 2.25 2.25 2.24 2.23 2.23 2.22 1.83 1.83 1.82 1.81 1.80 1.80 1.79 1.61 H2O 1.26 1.25 1.23 1.22 1.21 1.14
F F H N S N N Bn
^{1}H NMR of **13**
0.96 0.98 1.05 1.01 2.12 2.95 1.00 0.97 0.98 5.13 0.95 6.05
f1 (ppm)

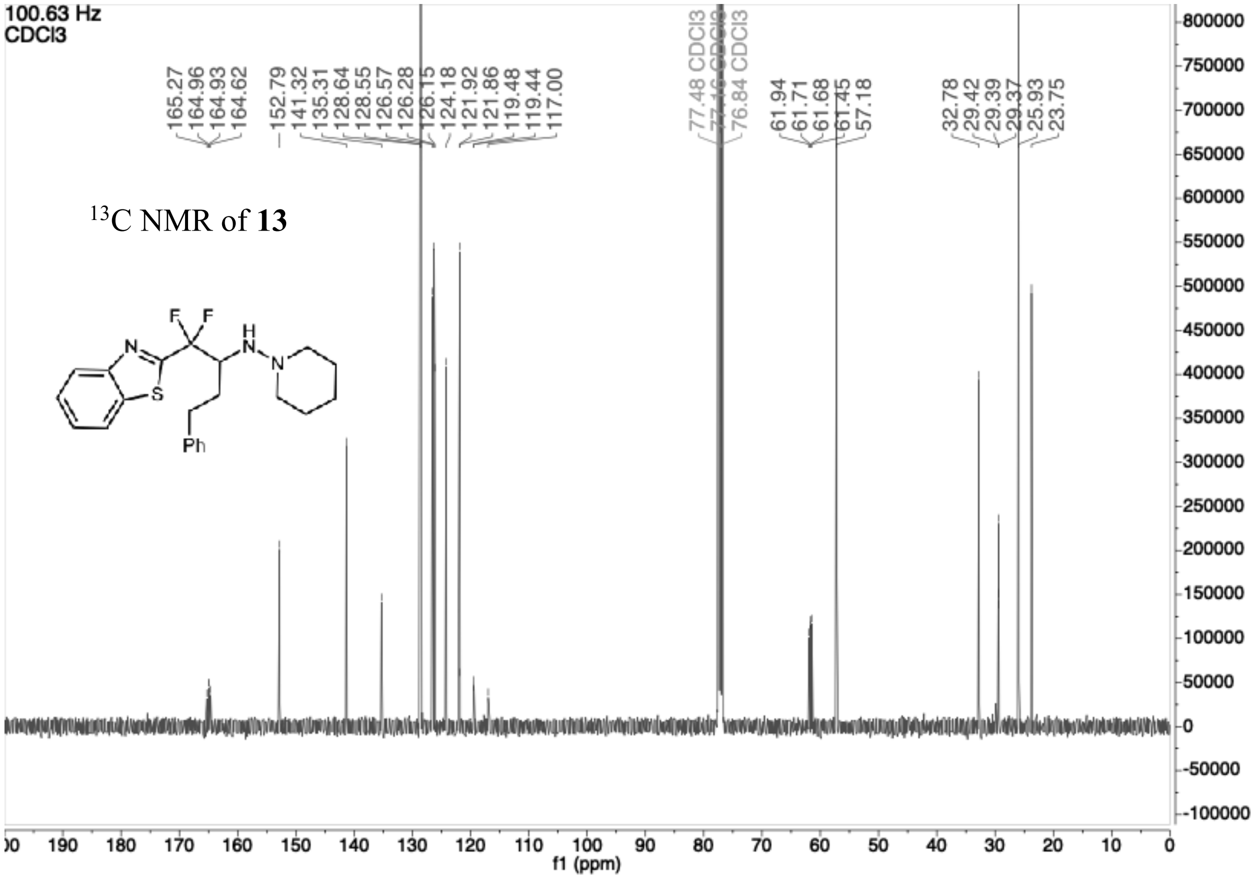
100.63 Hz
CDCl3
165.27 164.96 164.93 164.62 152.79 141.32 135.31 128.64 128.55 126.57 126.28 126.15 124.18 121.92 121.86 119.48 119.44 117.00 77.48 CDCl3 77.16 CDCl3 76.84 CDCl3 61.94 61.71 61.68 61.45 57.18 32.78 29.42 29.39 29.37 25.93 23.75
^{13}C NMR of **13**
F F H N S N N Ph
f1 (ppm)

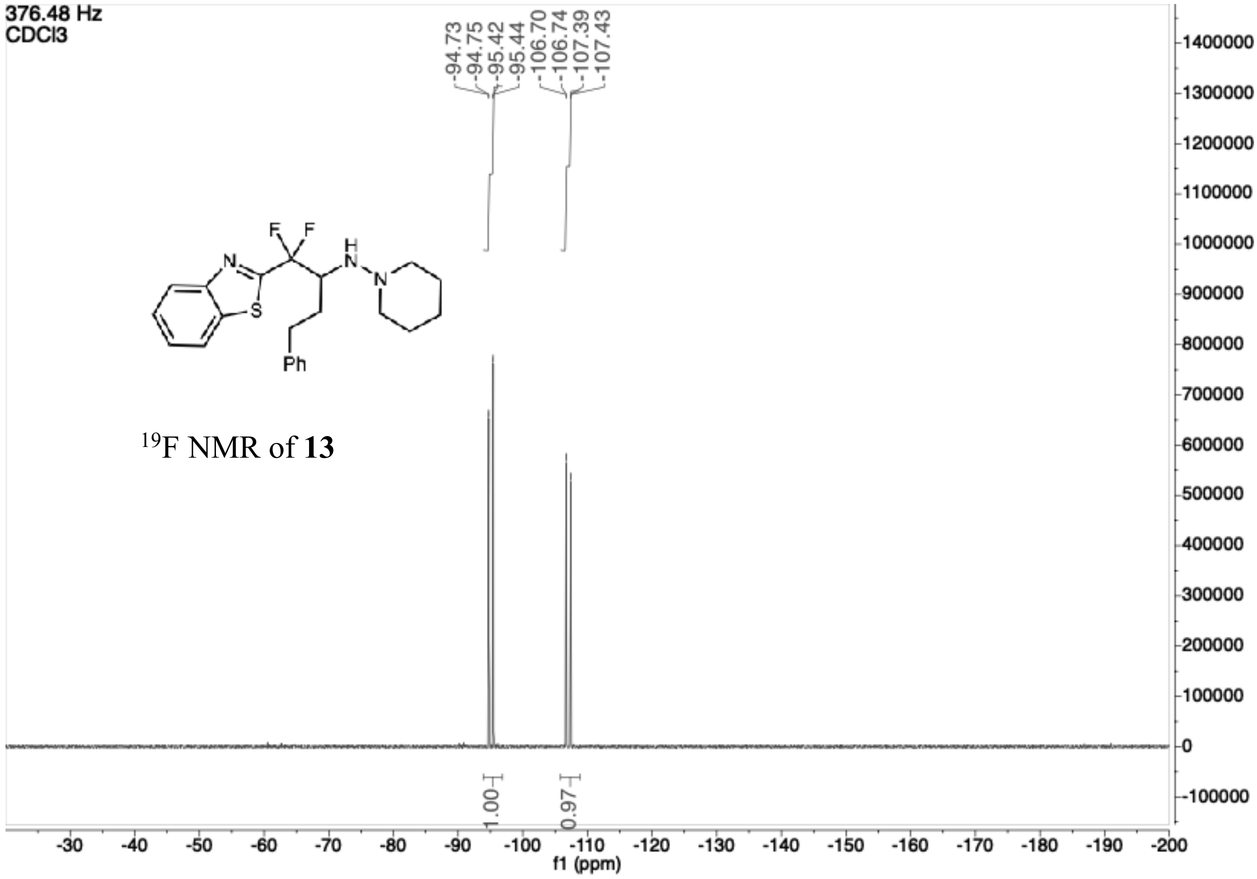
376.48 Hz
CDCl3
-94.73
-94.75
-95.42
-95.44
-106.70
-106.74
-107.39
-107.43
1.00
0.97
f1 (ppm)
F
F
N
H
N
S
Ph
¹⁹F NMR of 13

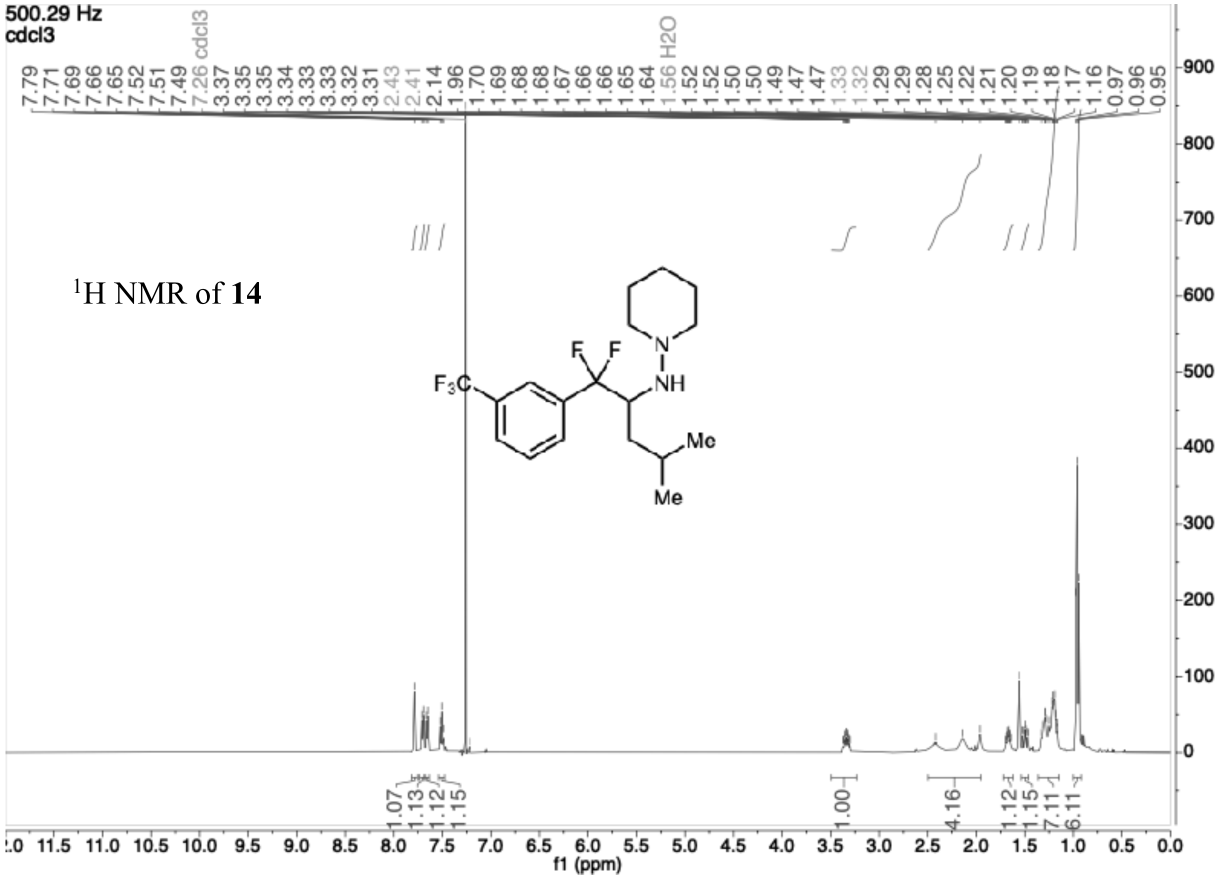

500.29 Hz
cdcl3
^{1}H NMR of 14
f1 (ppm)

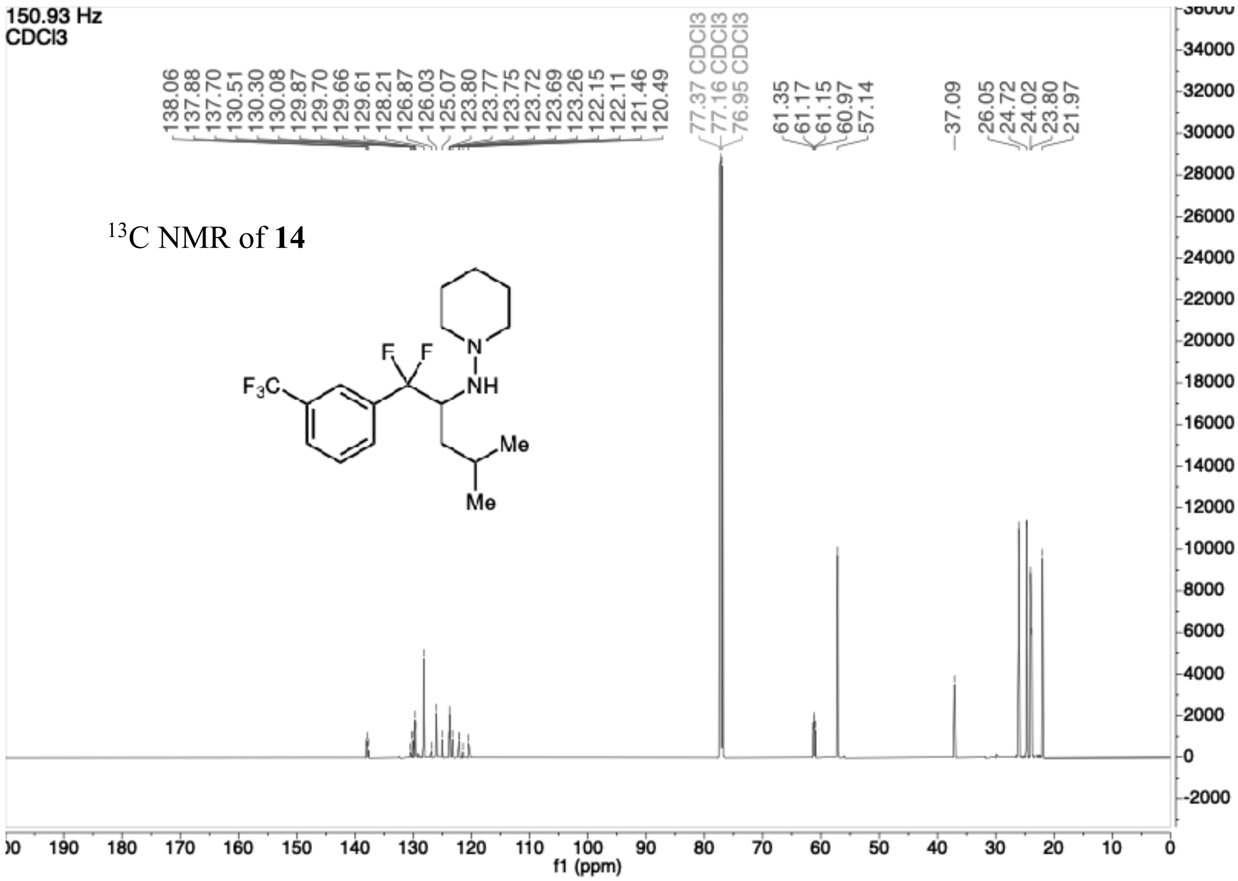

150.93 Hz
CDCl3
^{13}C NMR of 14
f1 (ppm)

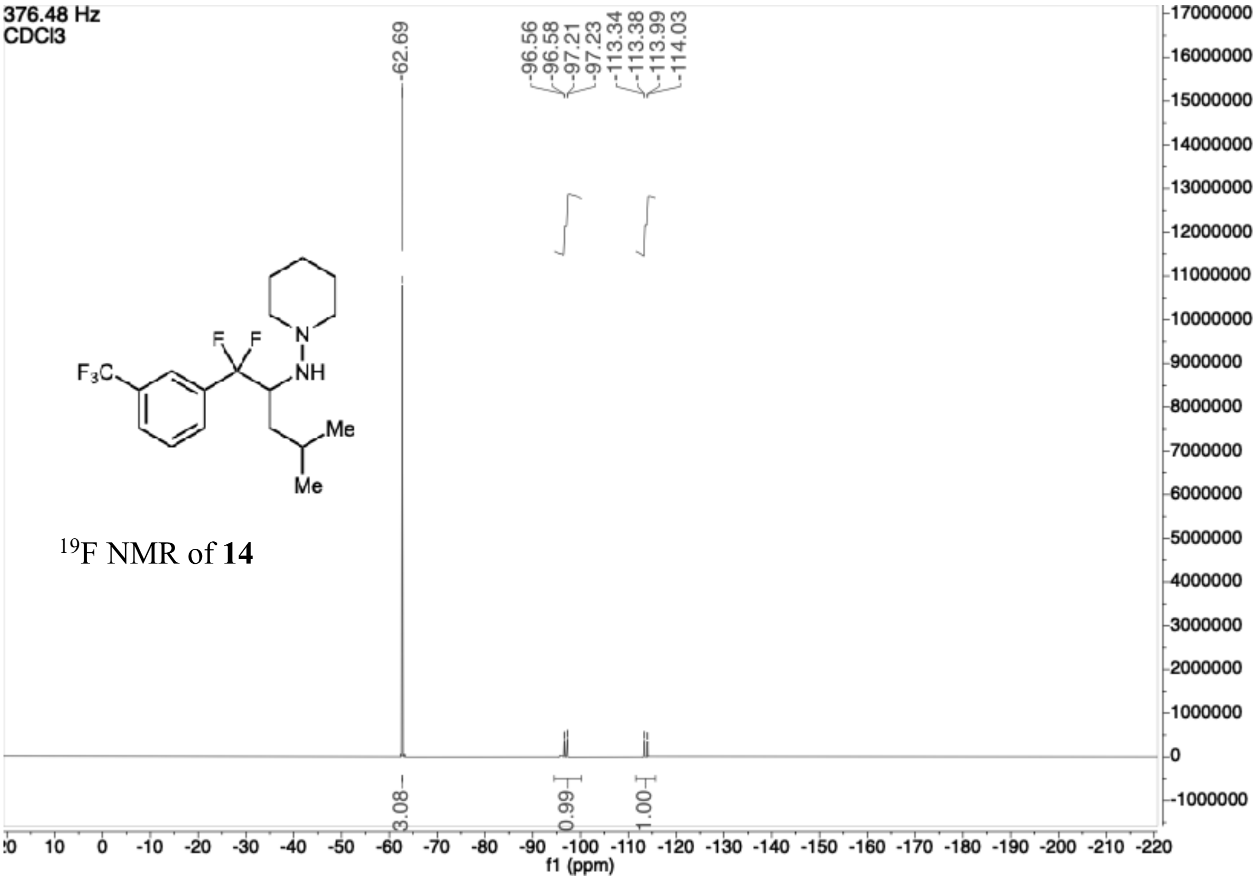
376.48 Hz
CDCl3
-62.69
-96.56
-96.58
-97.21
-97.23
-113.34
-113.38
-113.99
-114.03
3.08
0.99
1.00
f1 (ppm)
F3C
F
F
N
NH
Me
Me

^{19}F NMR of **14**

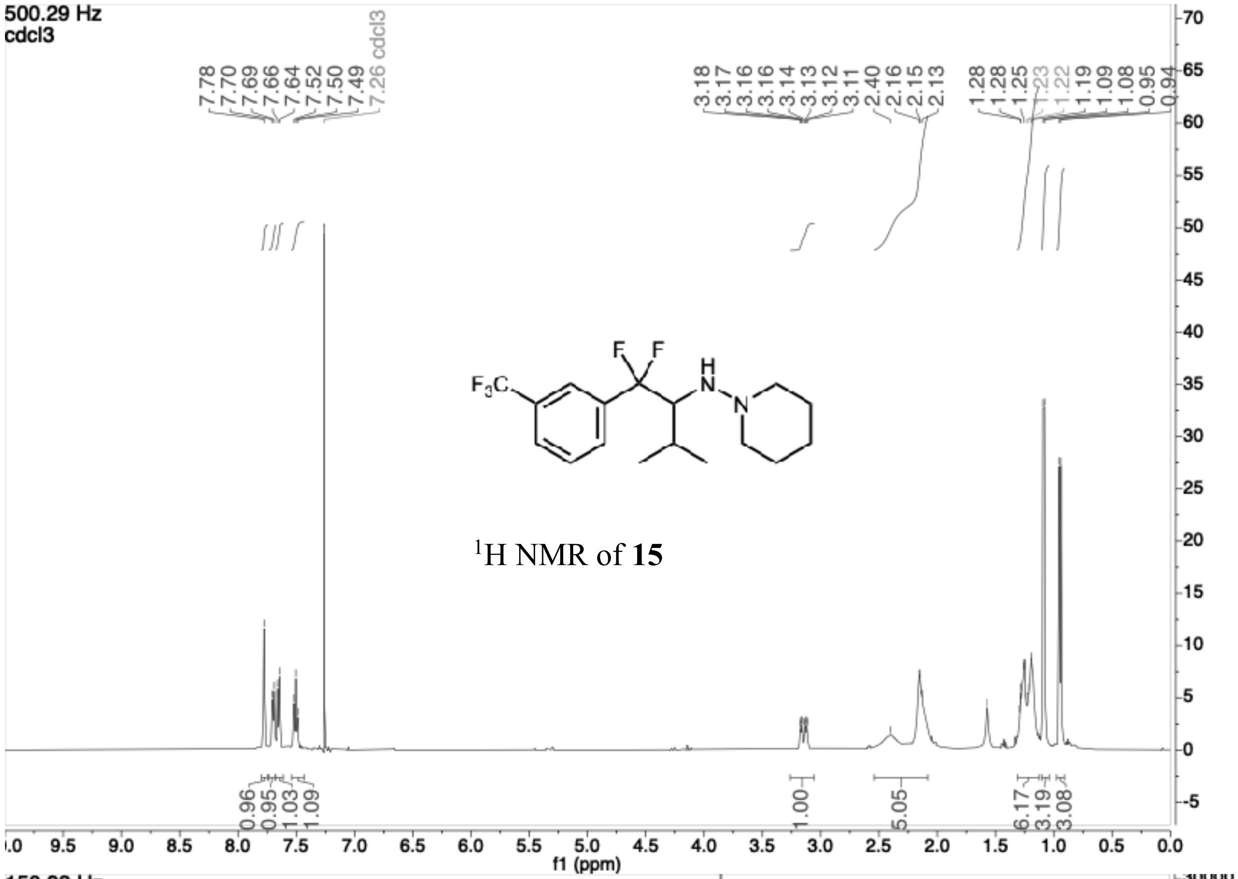
500.29 Hz
cdcl3
^{1}H NMR of **15**
f1 (ppm)

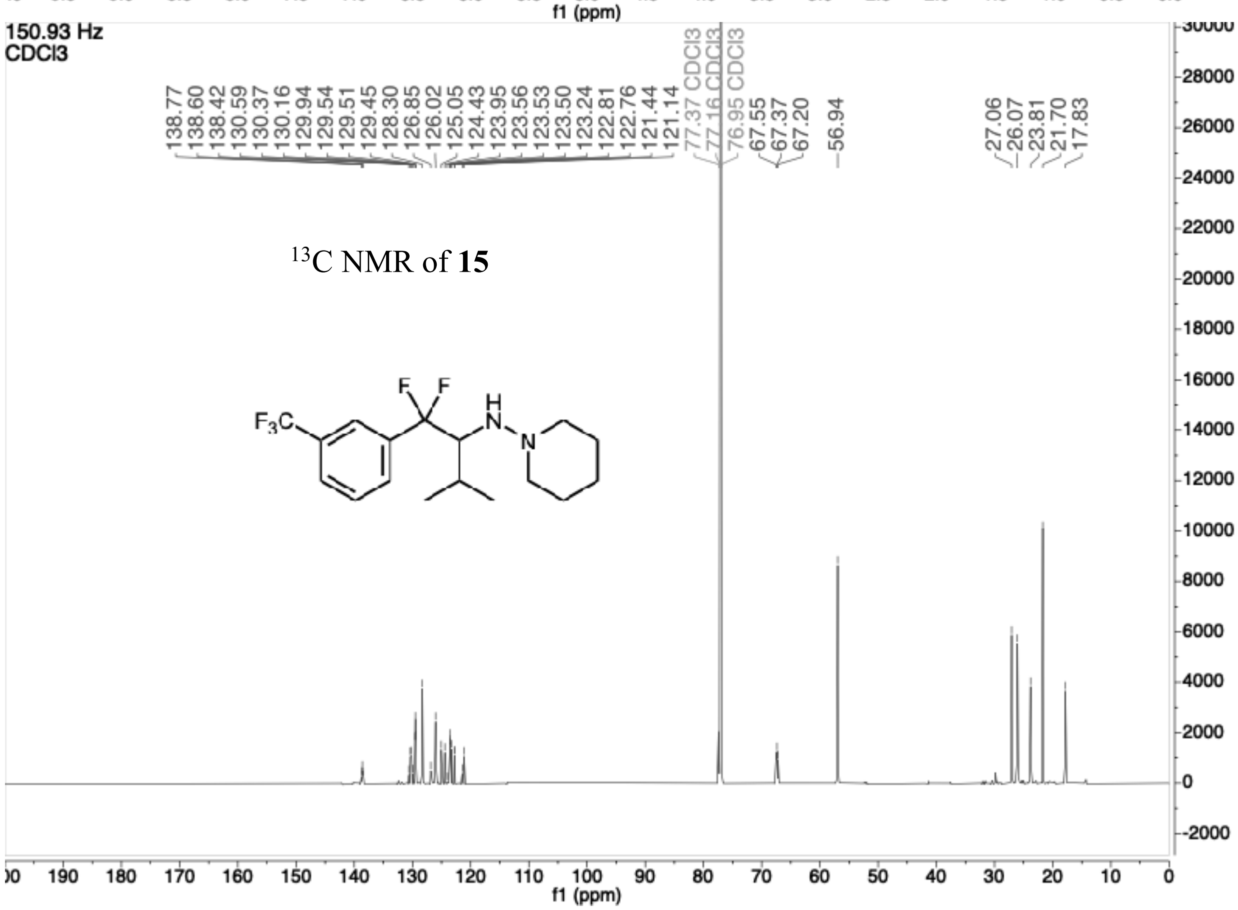
150.93 Hz
CDCl3
^{13}C NMR of **15**
f1 (ppm)

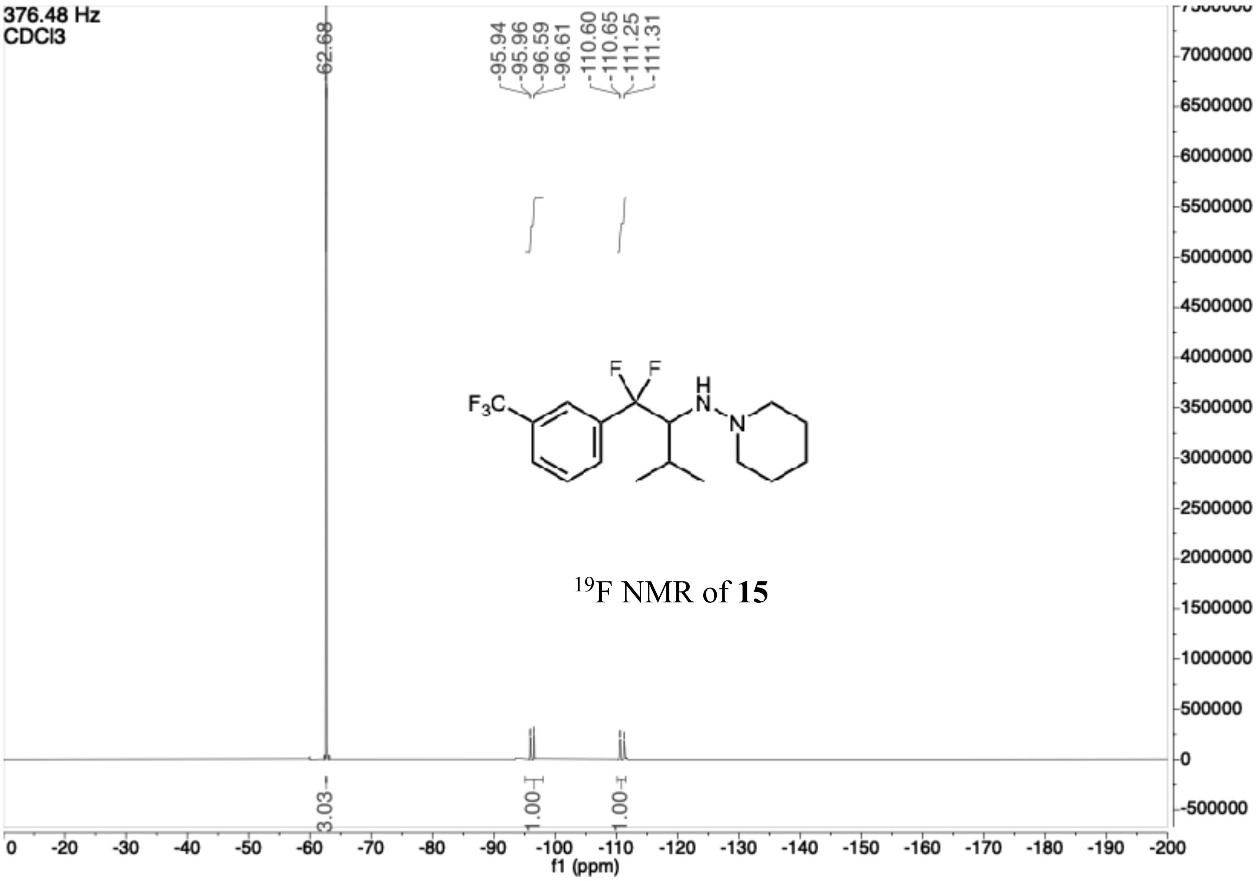
376.48 Hz
CDCl3
-62.68
-95.94
-95.96
-96.59
-96.61
-110.60
-110.65
-111.25
-111.31
3.03
1.00
1.00
f1 (ppm)

^{19}F NMR of **15**

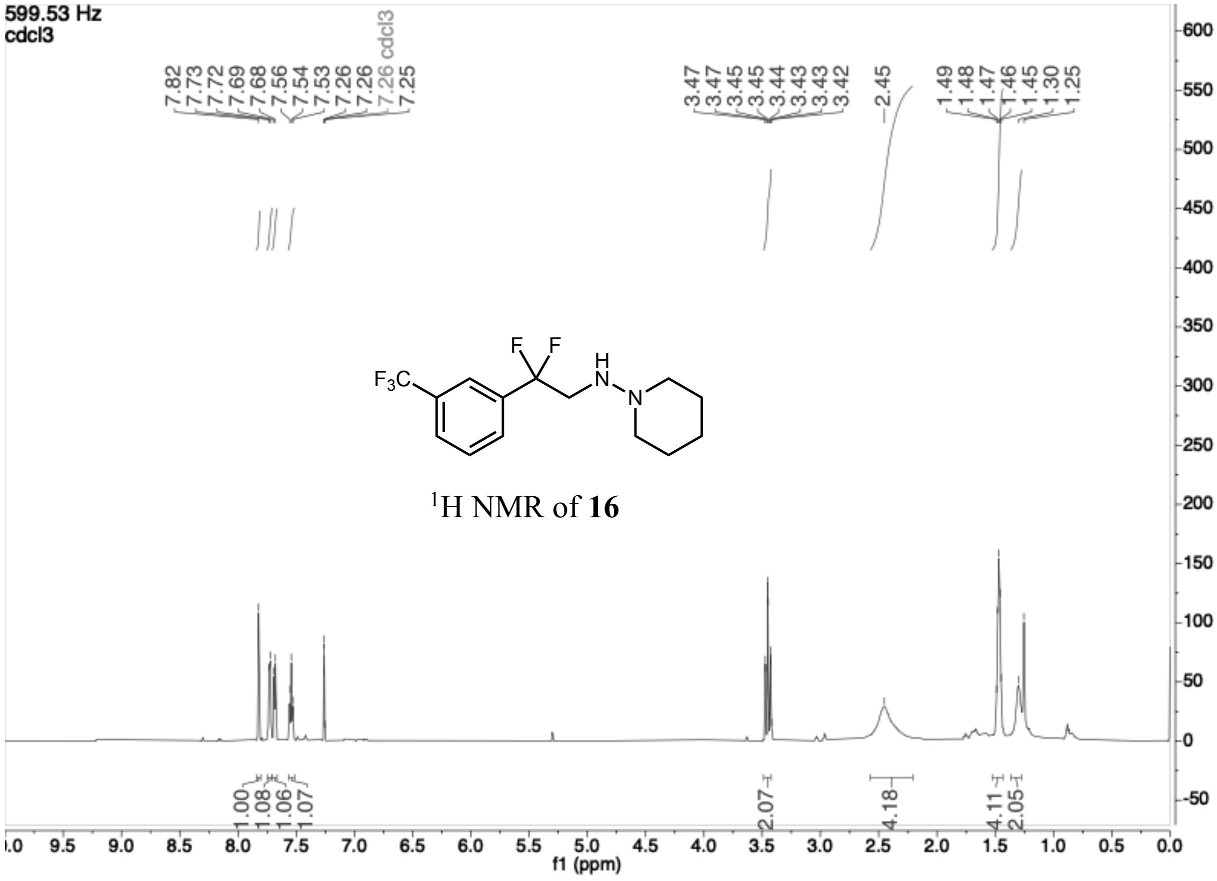

^{1}H NMR of **16**

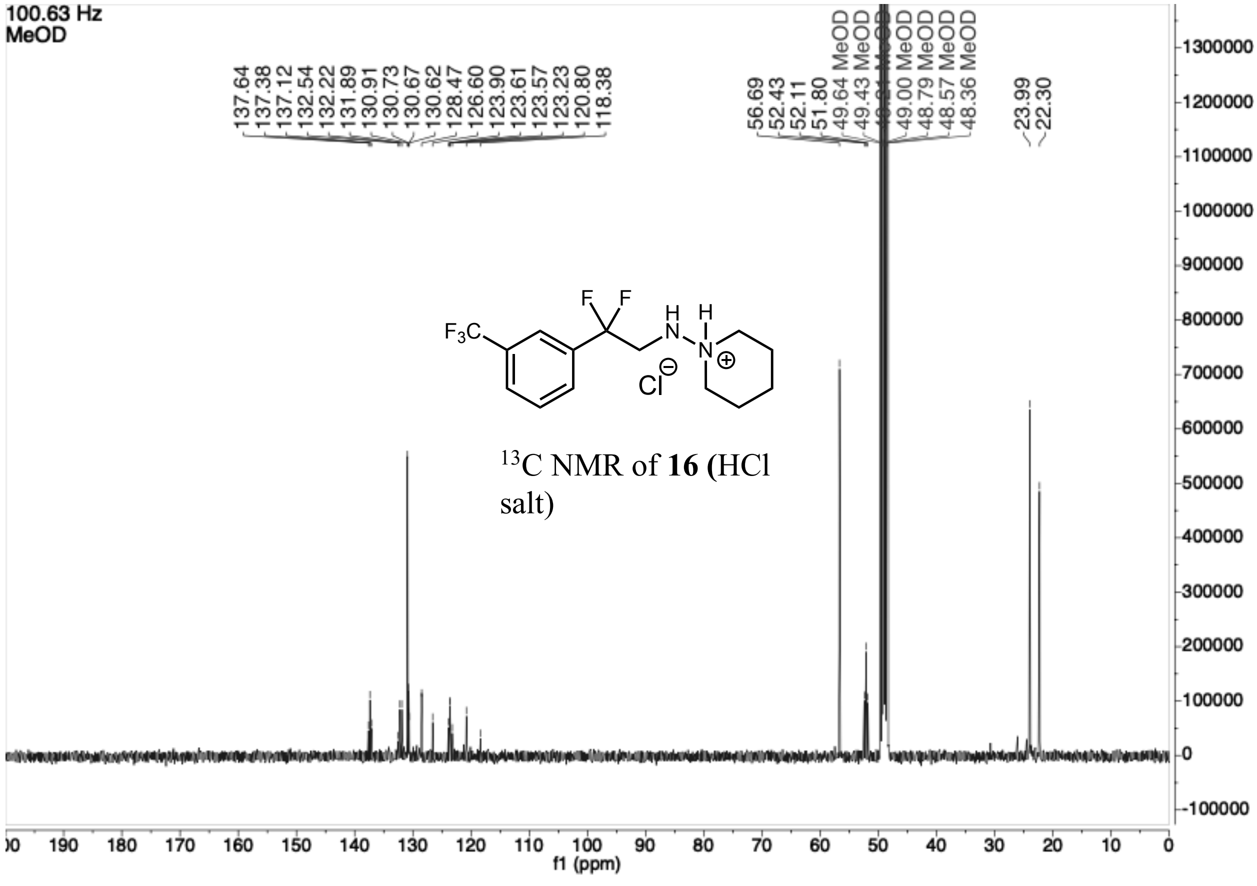

^{13}C NMR of **16 (**HCl salt)

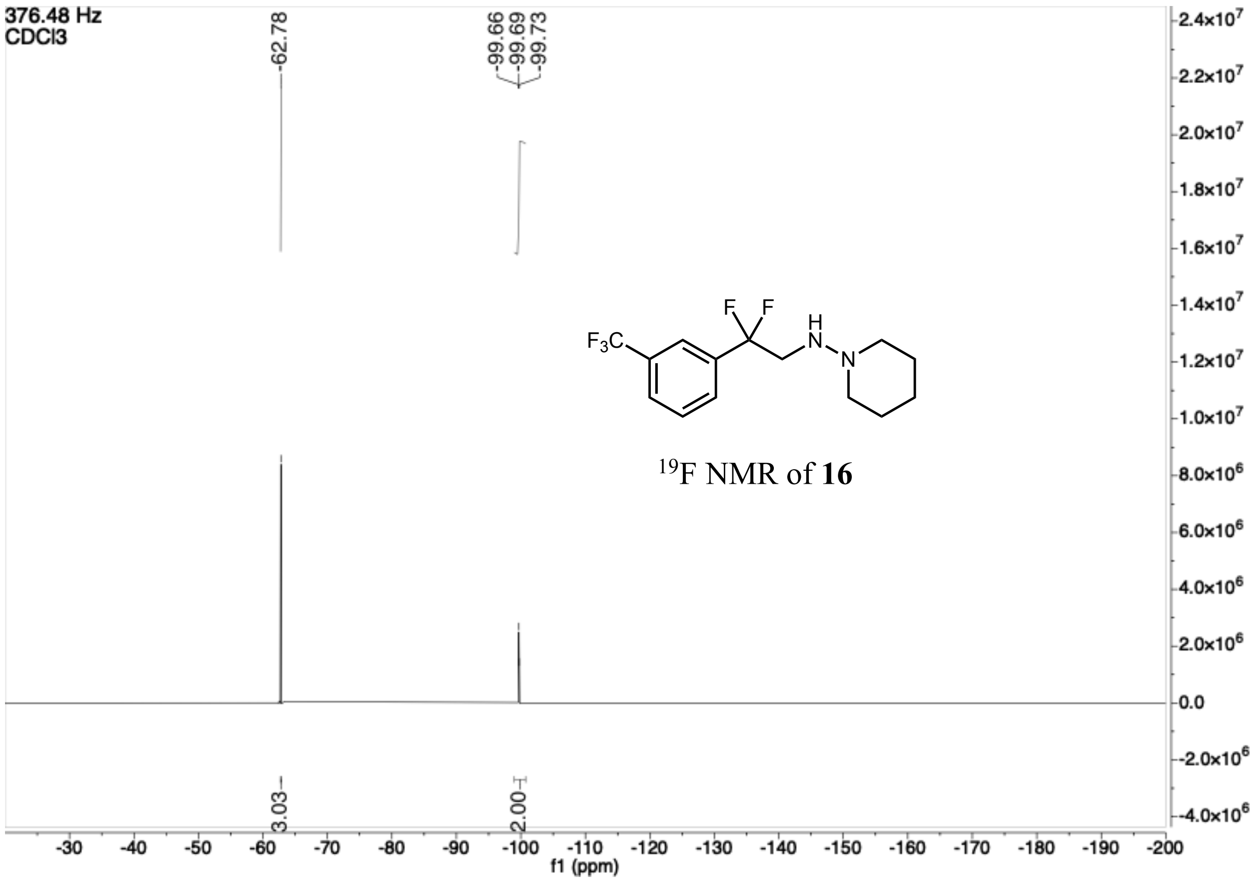
376.48 Hz
CDCl3
-62.78
-99.66
-99.69
-99.73
3.03
2.00
f1 (ppm)
F F
F3C
H
N
N

^{19}F NMR of **16**

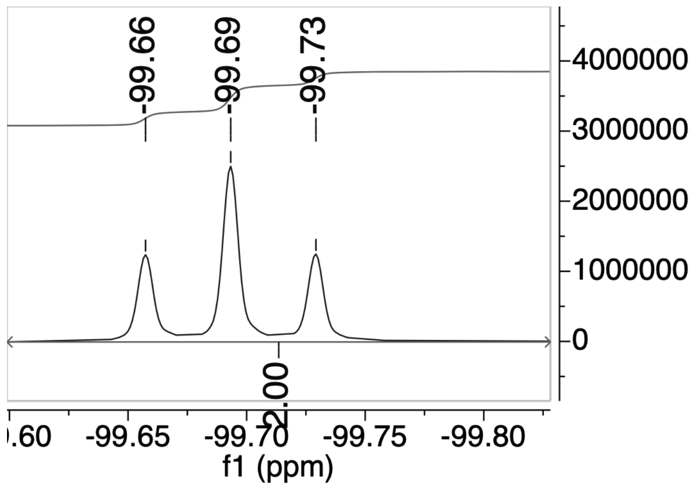
-99.66
-99.69
-99.73
2.00
f1 (ppm)

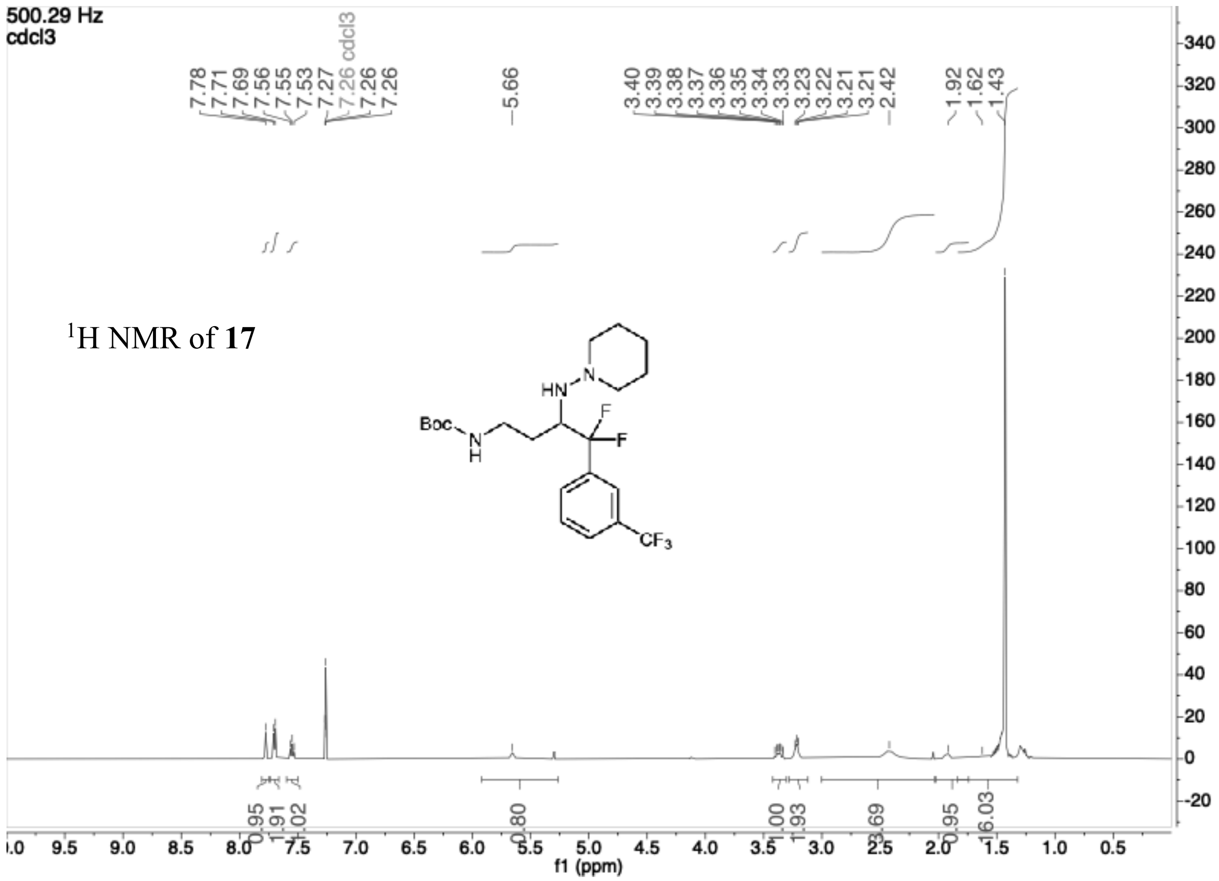

^{1}H NMR of **17**

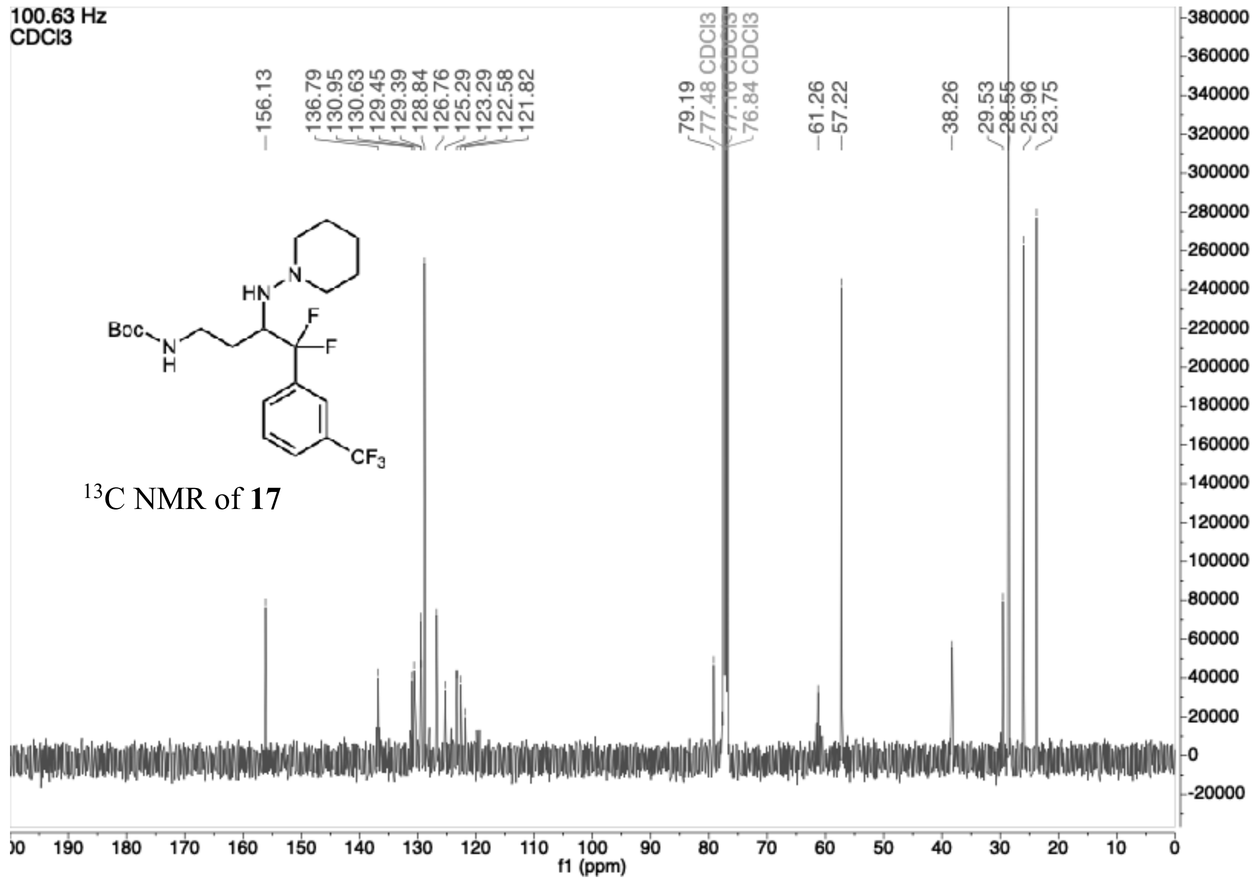

^{13}C NMR of **17**

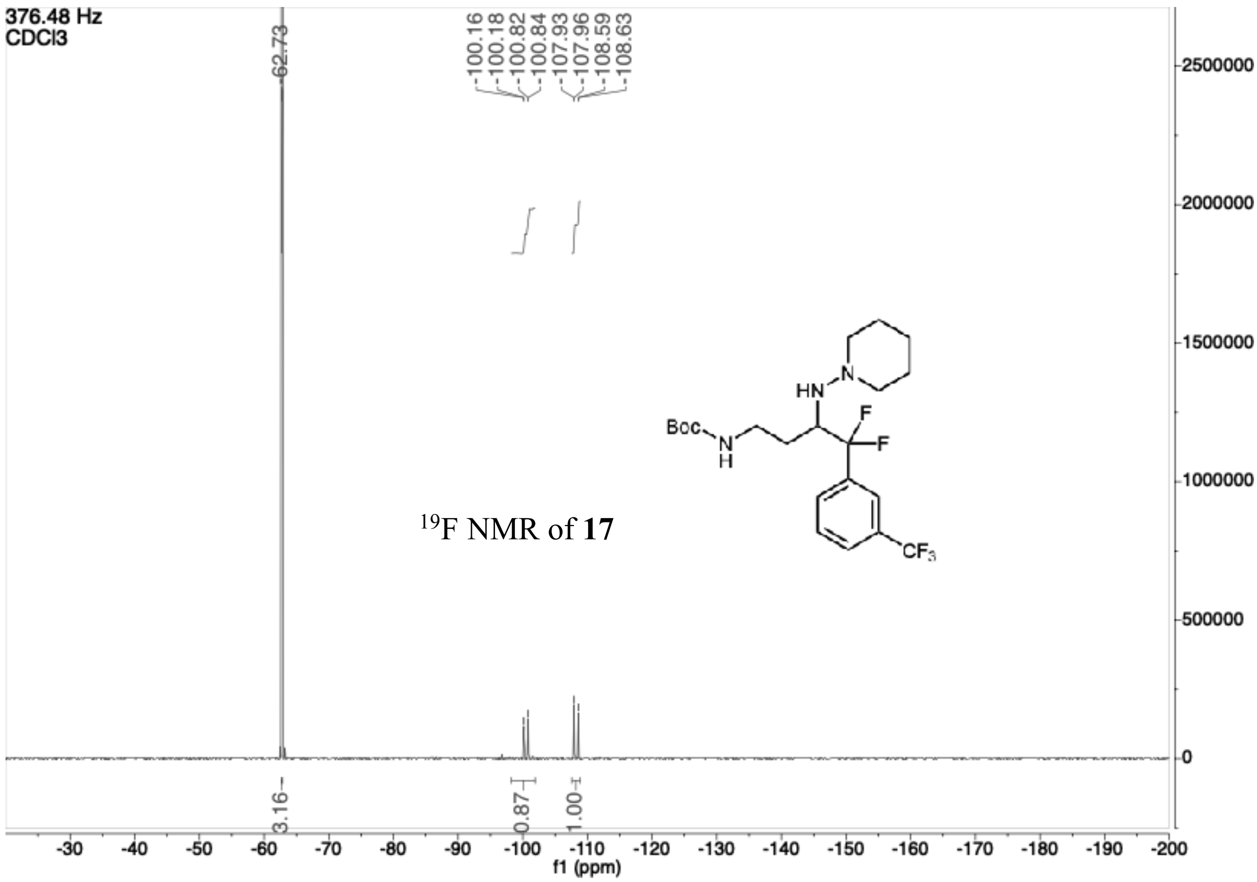
376.48 Hz
CDCl3
-62.73
-100.16
-100.18
-100.82
-100.84
-107.93
-107.96
-108.59
-108.63
Boc
N
H
HN
N
F
F
CF3
3.16
0.87
1.00
f1 (ppm)

^{19}F NMR of **17**

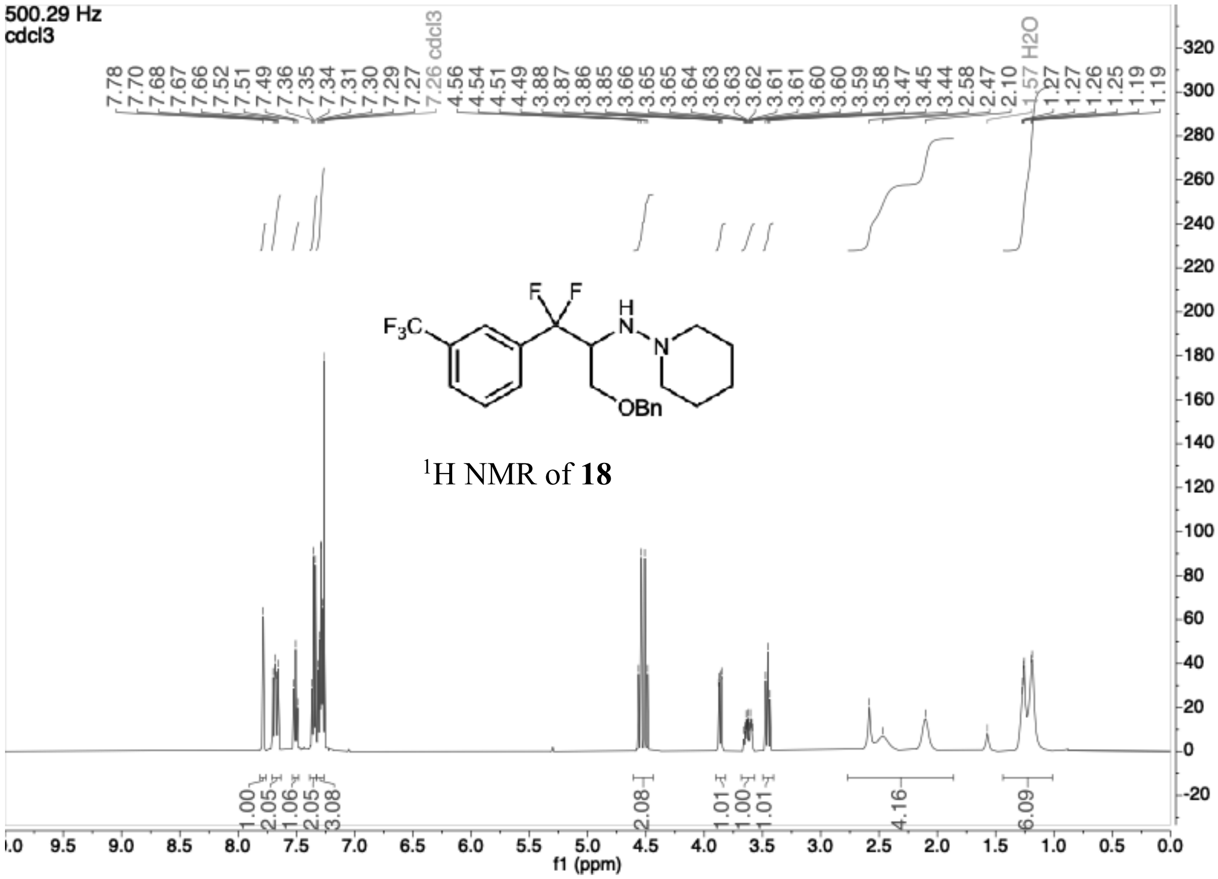

^{1}H NMR of **18**

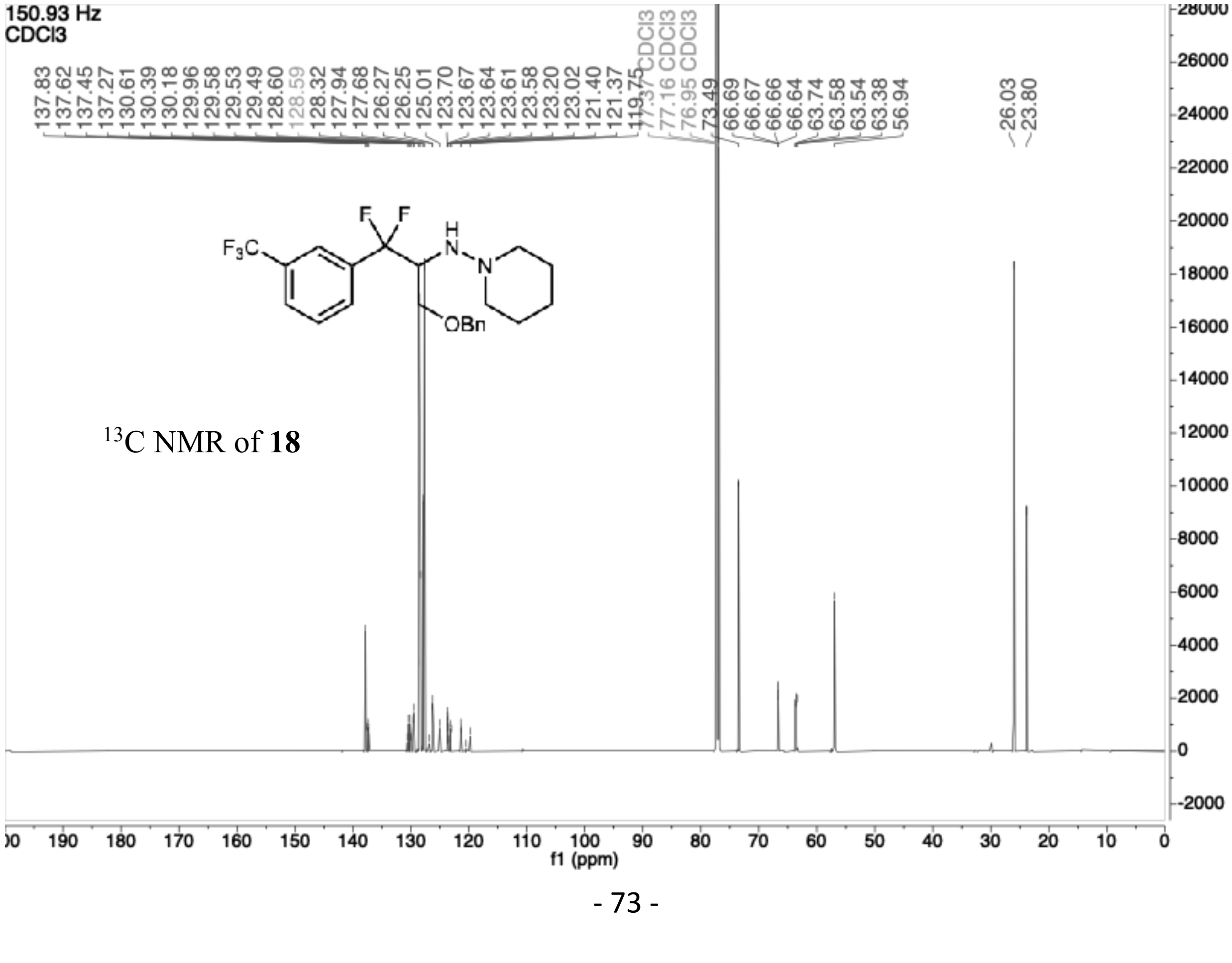

^{13}C NMR of **18**

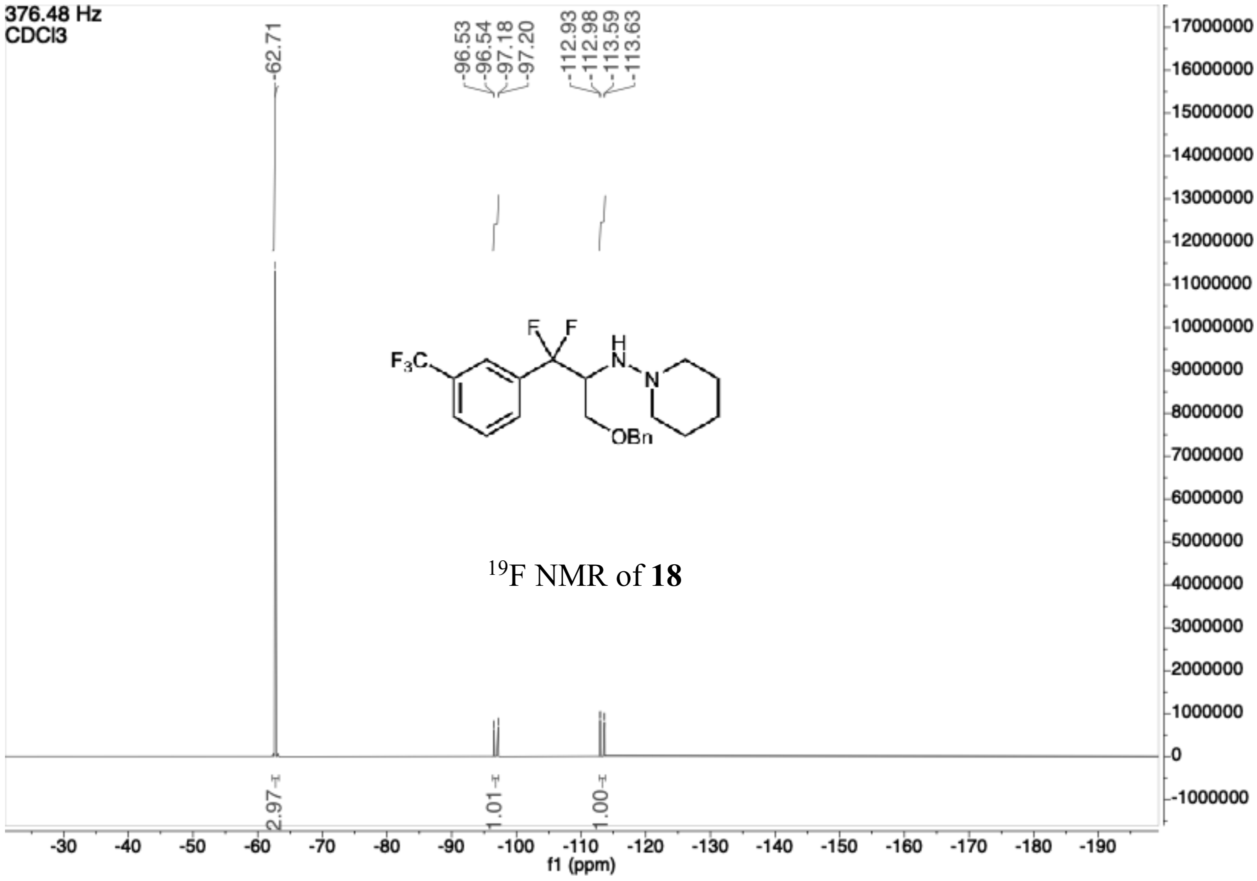

376.48 Hz
CDCl3
-62.71
-96.53
-96.54
-97.18
-97.20
-112.93
-112.98
-113.59
-113.63
2.97
1.01
1.00
f1 (ppm)
F3C
F F
H
N
N
OBn

^{19}F NMR of **18**

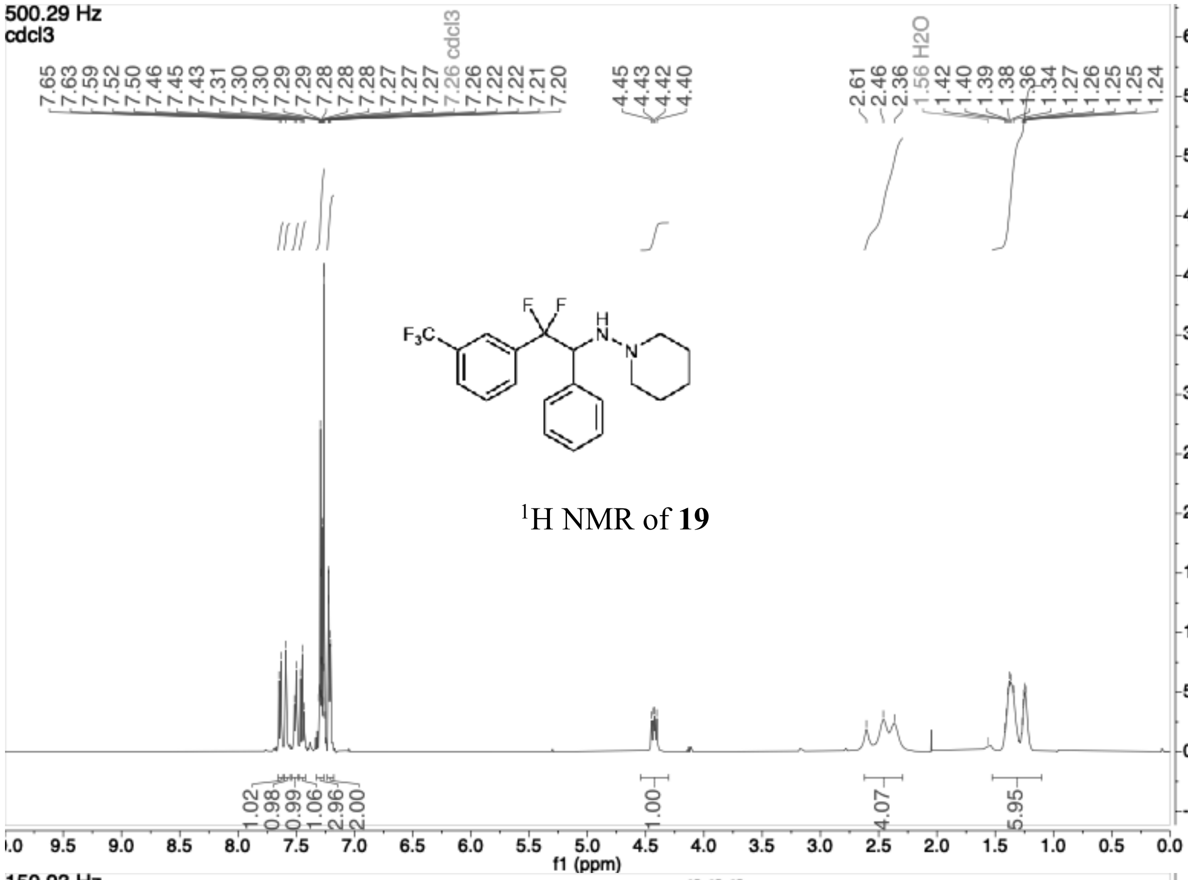

^{1}H NMR of **19**

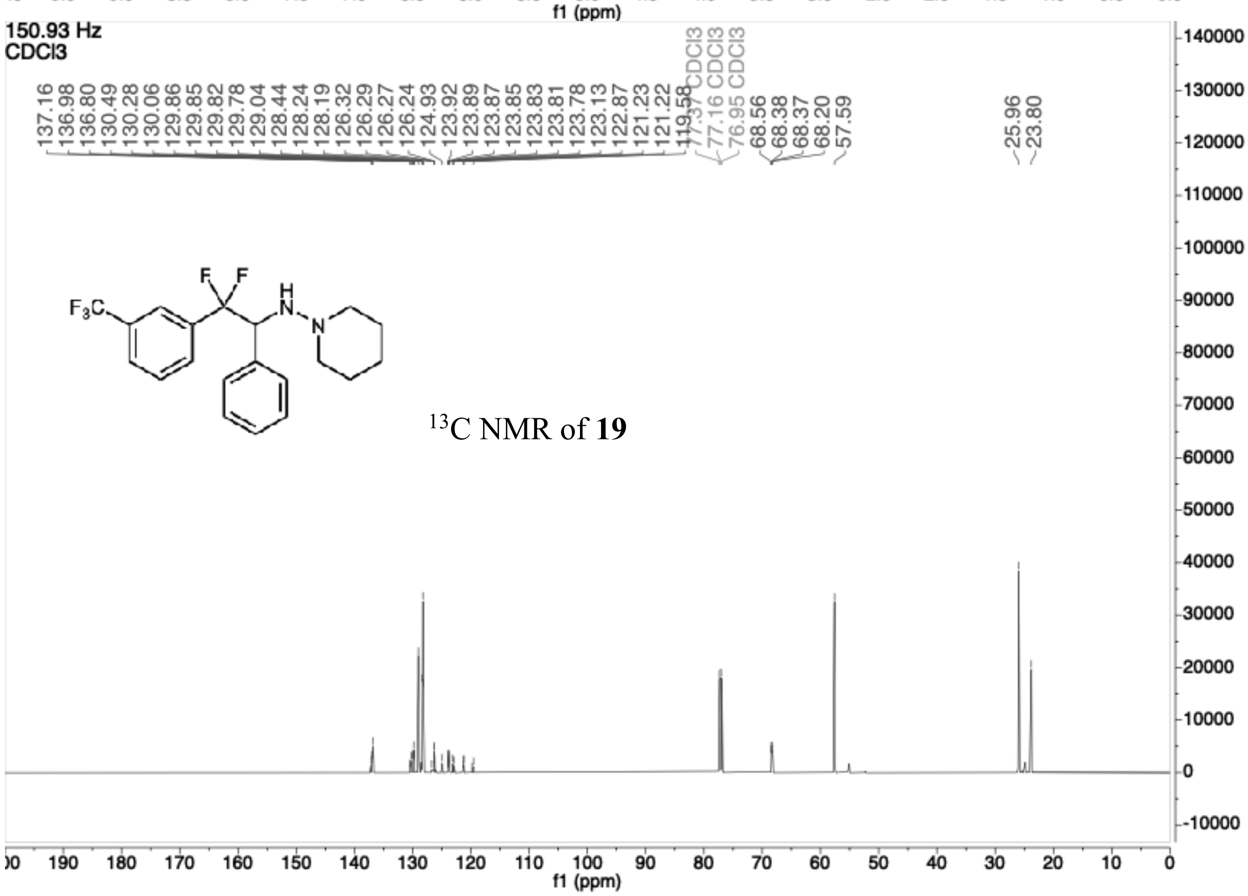

^{13}C NMR of **19**

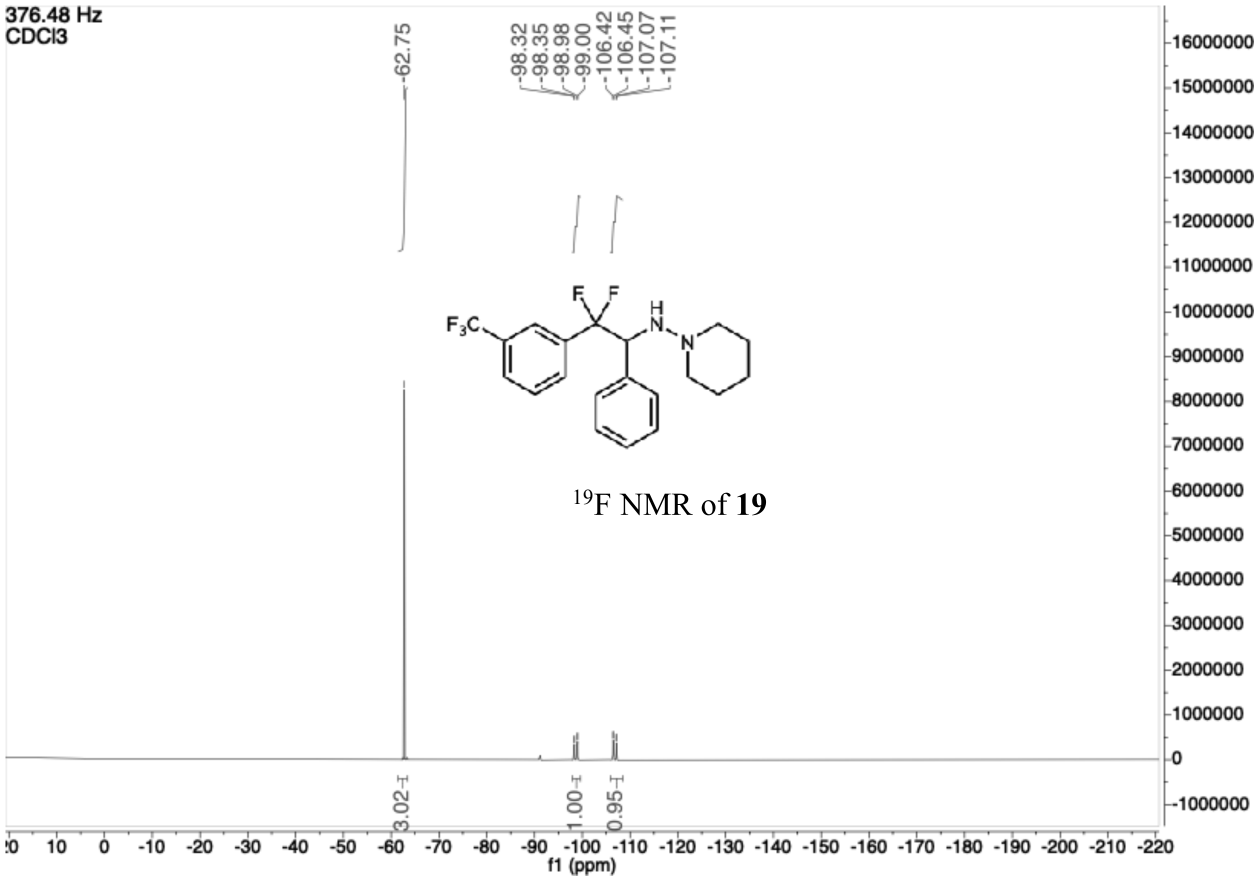
376.48 Hz
CDCl3
-62.75
-98.32
-98.35
-98.98
-99.00
-106.42
-106.45
-107.07
-107.11
3.02
1.00
0.95
f1 (ppm)

^{19}F NMR of **19**

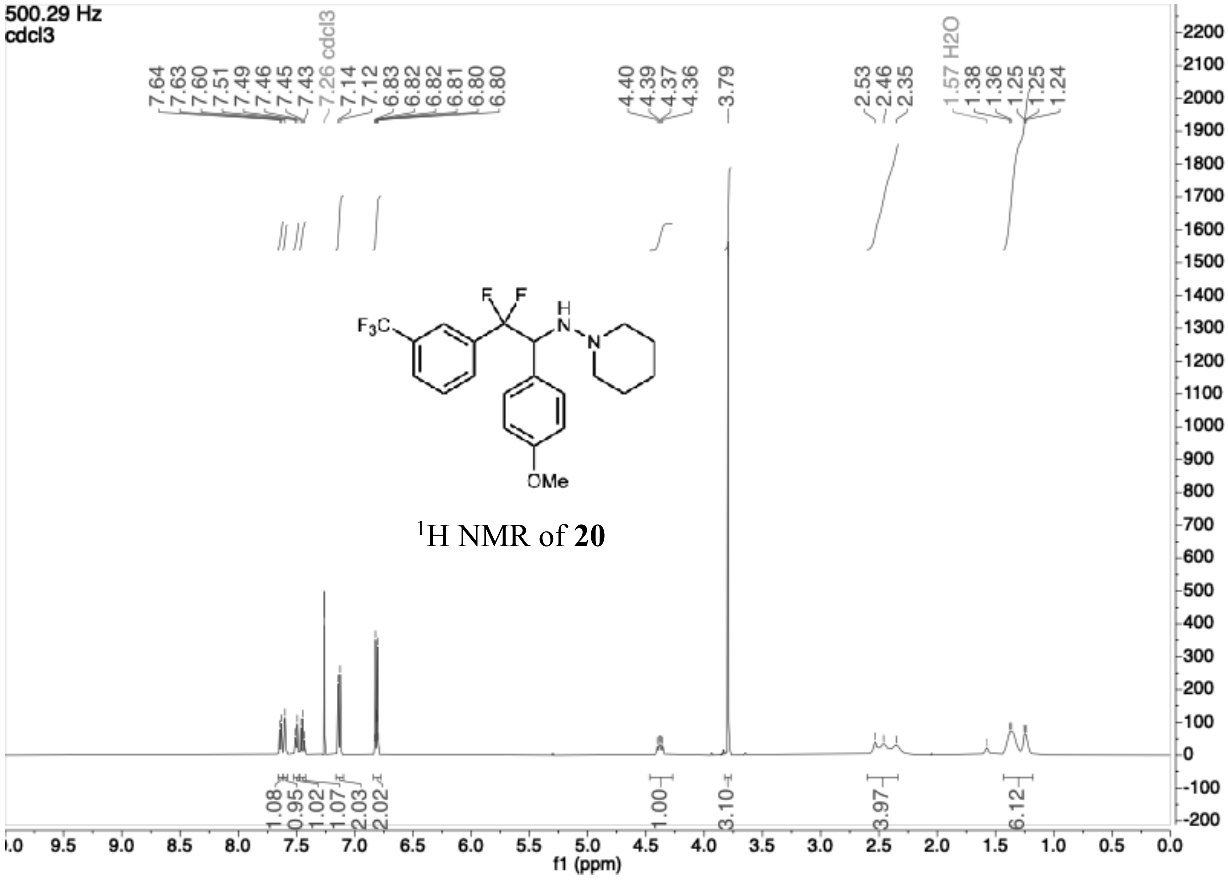

^{1}H NMR of **20**

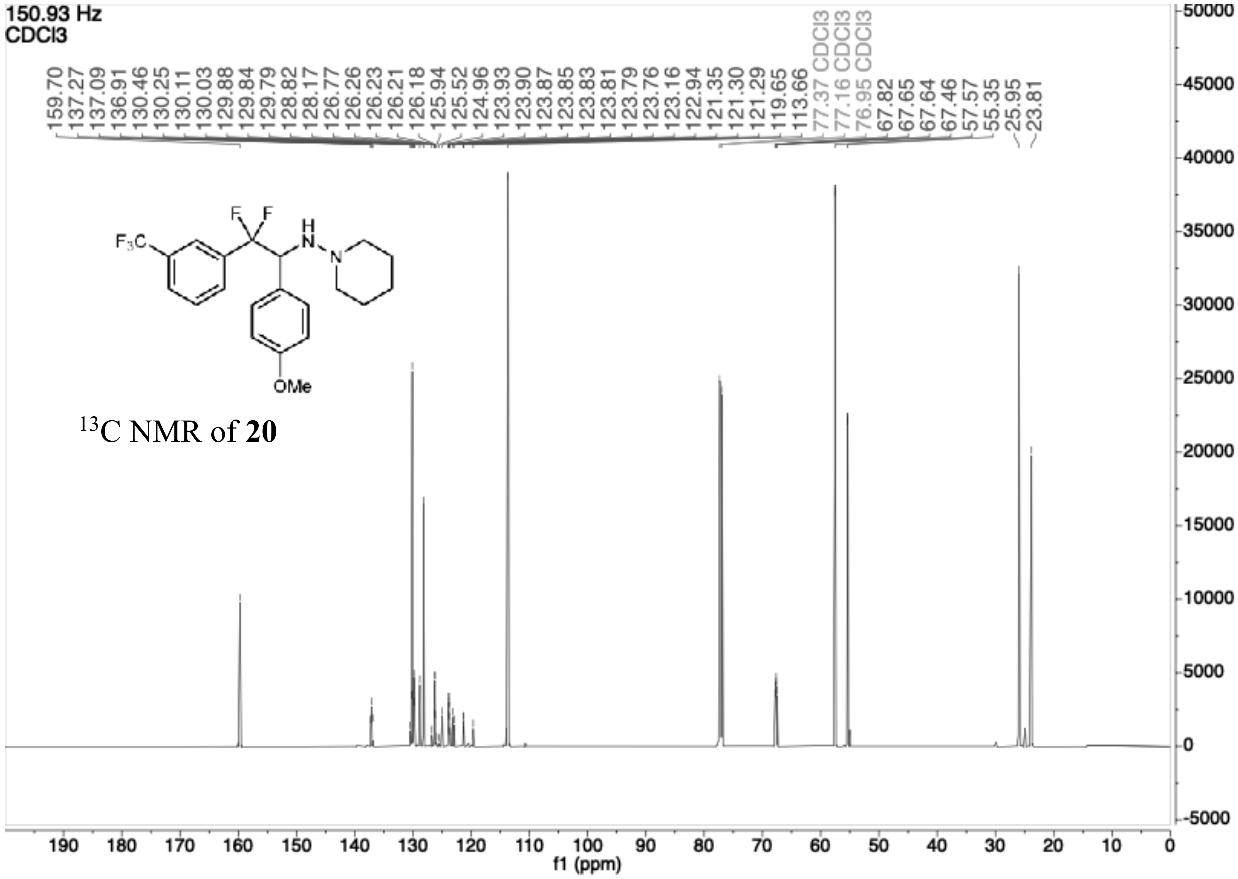

^{13}C NMR of **20**

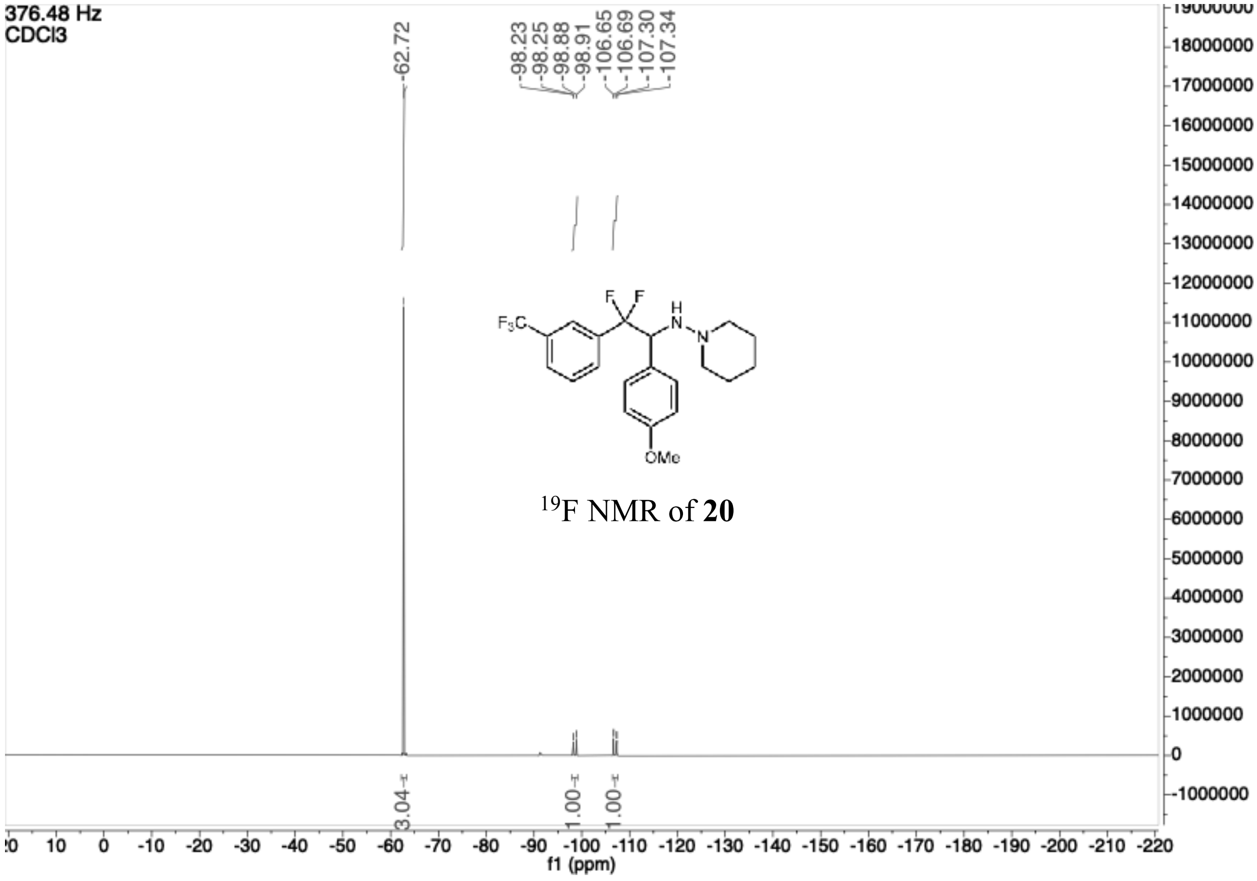
376.48 Hz
CDCl3
-62.72
-98.23
-98.25
-98.88
-98.91
-106.65
-106.69
-107.30
-107.34
3.04
1.00
1.00
f1 (ppm)
F3C
F F
H
N
N
OMe

^{19}F NMR of **20**

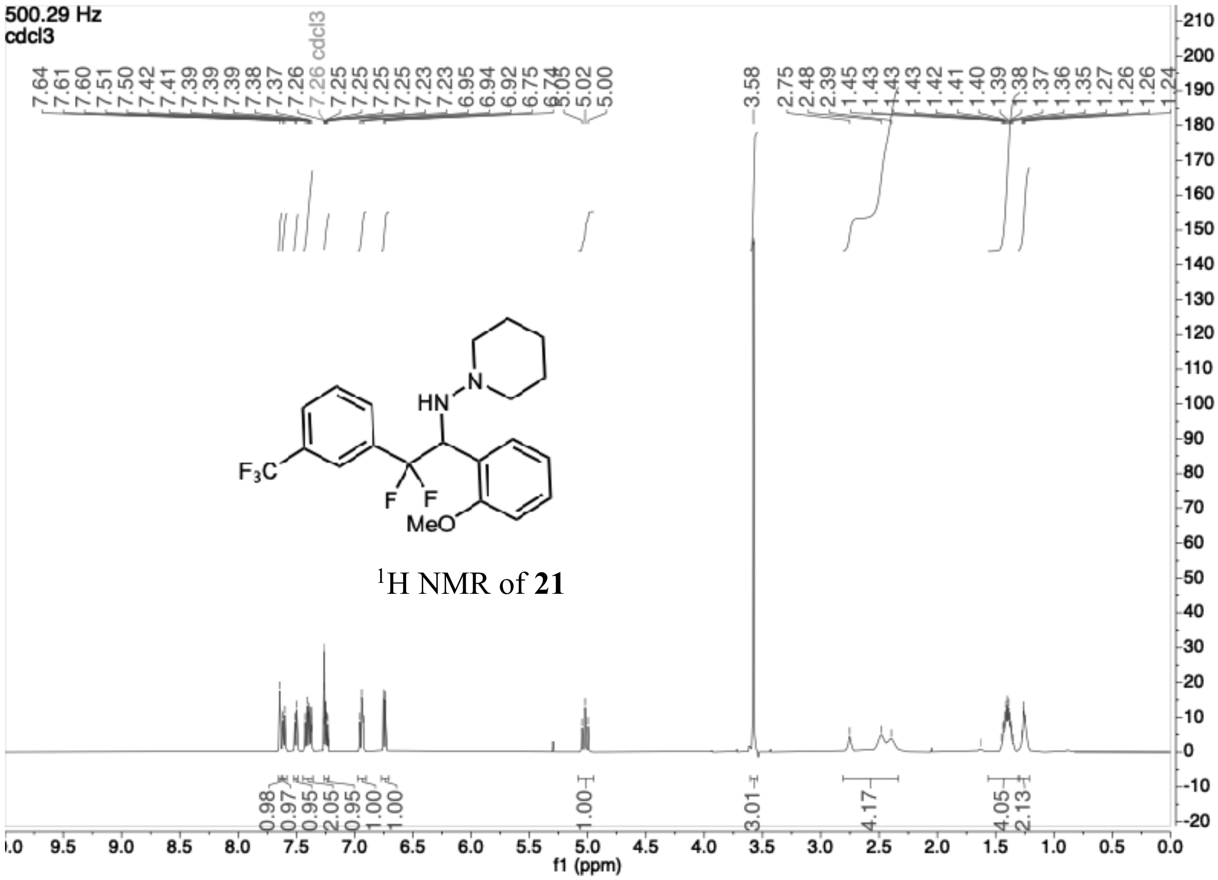

^{1}H NMR of **21**

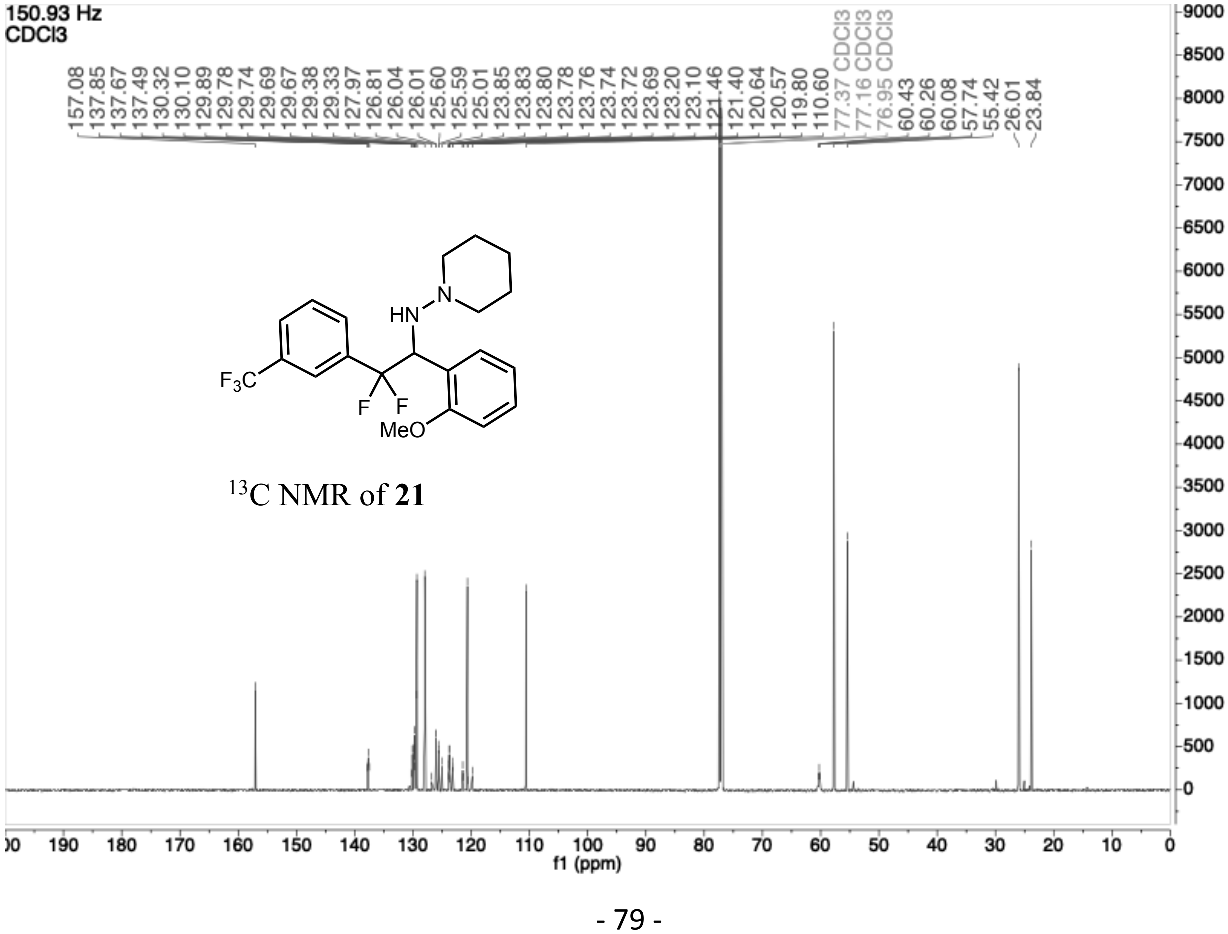

^{13}C NMR of **21**

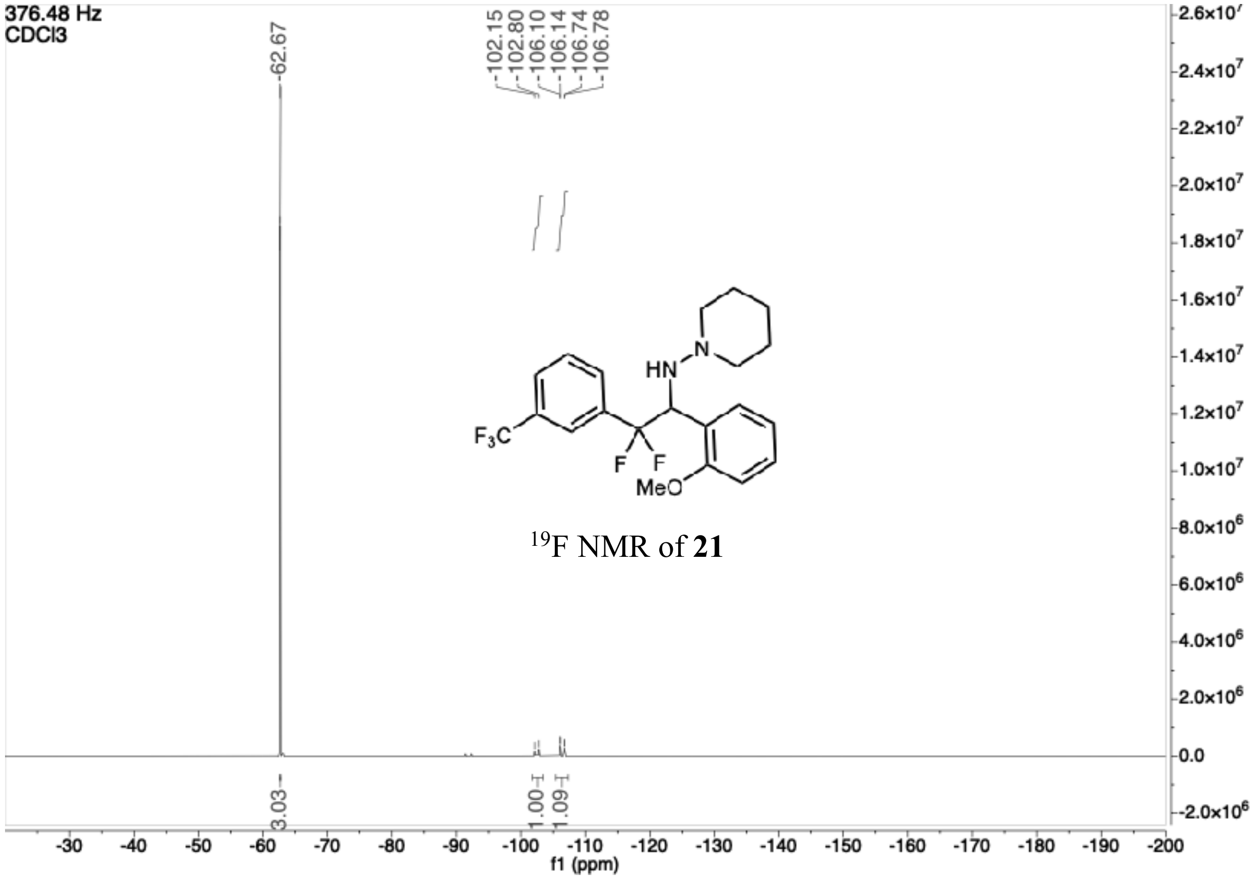
376.48 Hz
CDCl3
-62.67
-102.15
-102.80
-106.10
-106.14
-106.74
-106.78
HN
N
F3C
F F
MeO
3.03
1.00
1.09
f1 (ppm)

^{19}F NMR of **21**

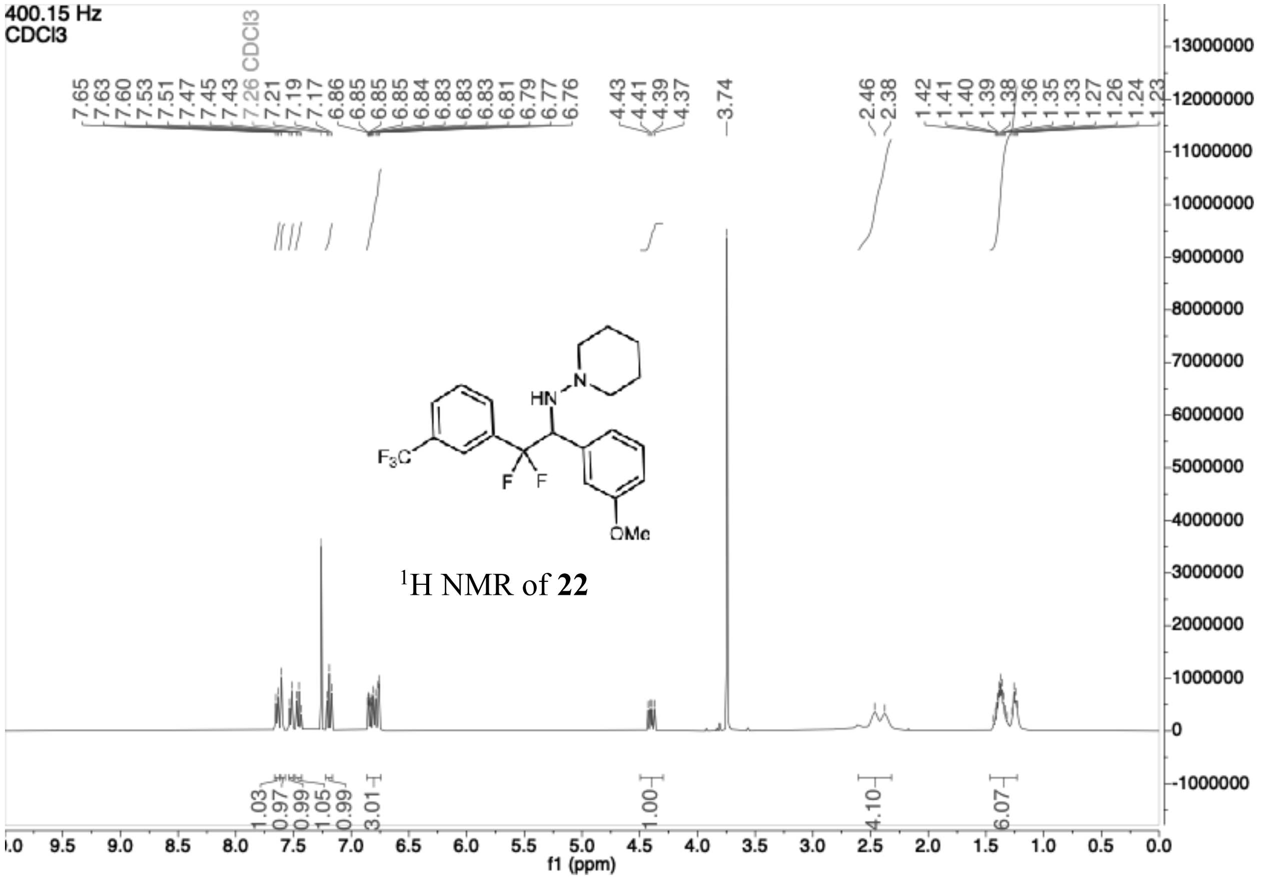

^{1}H NMR of **22**

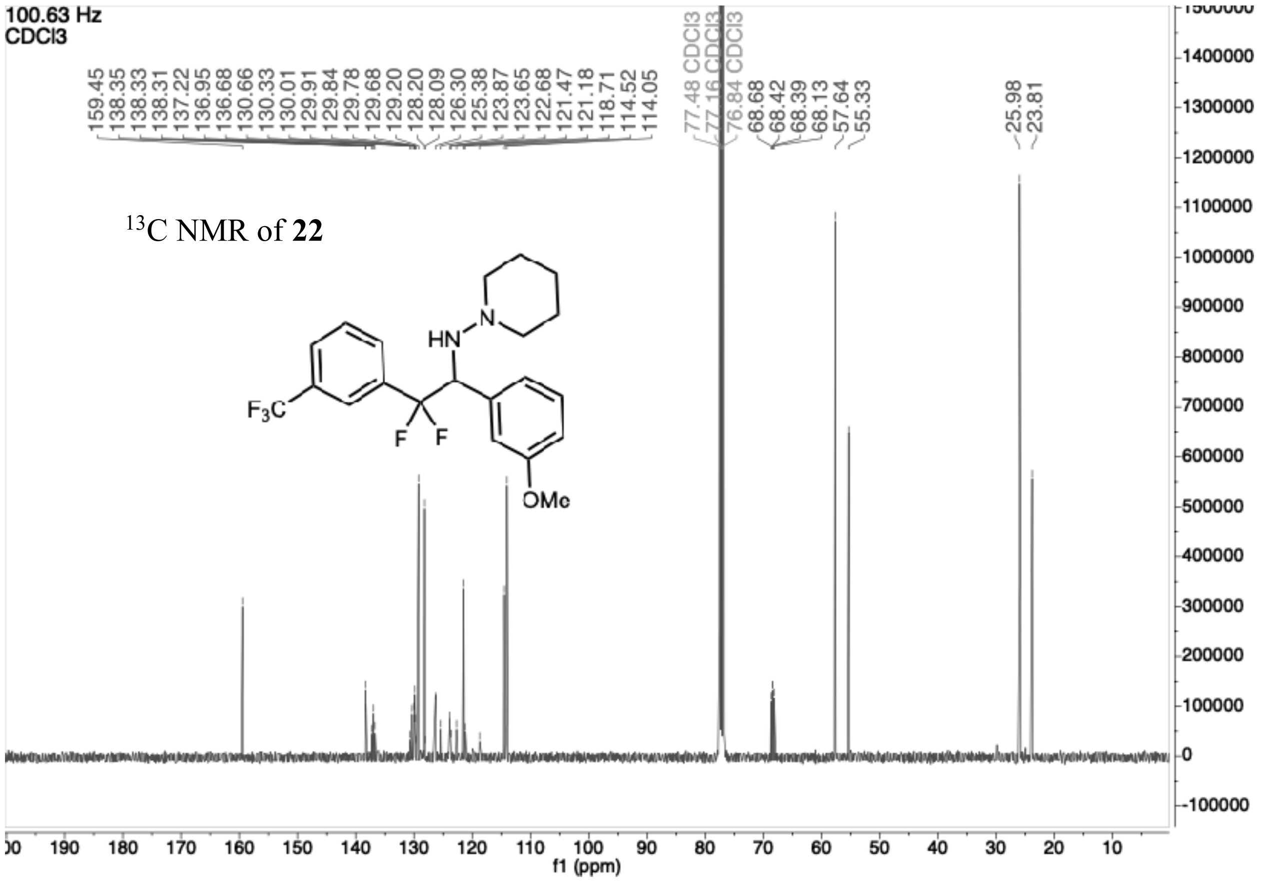

^{13}C NMR of **22**

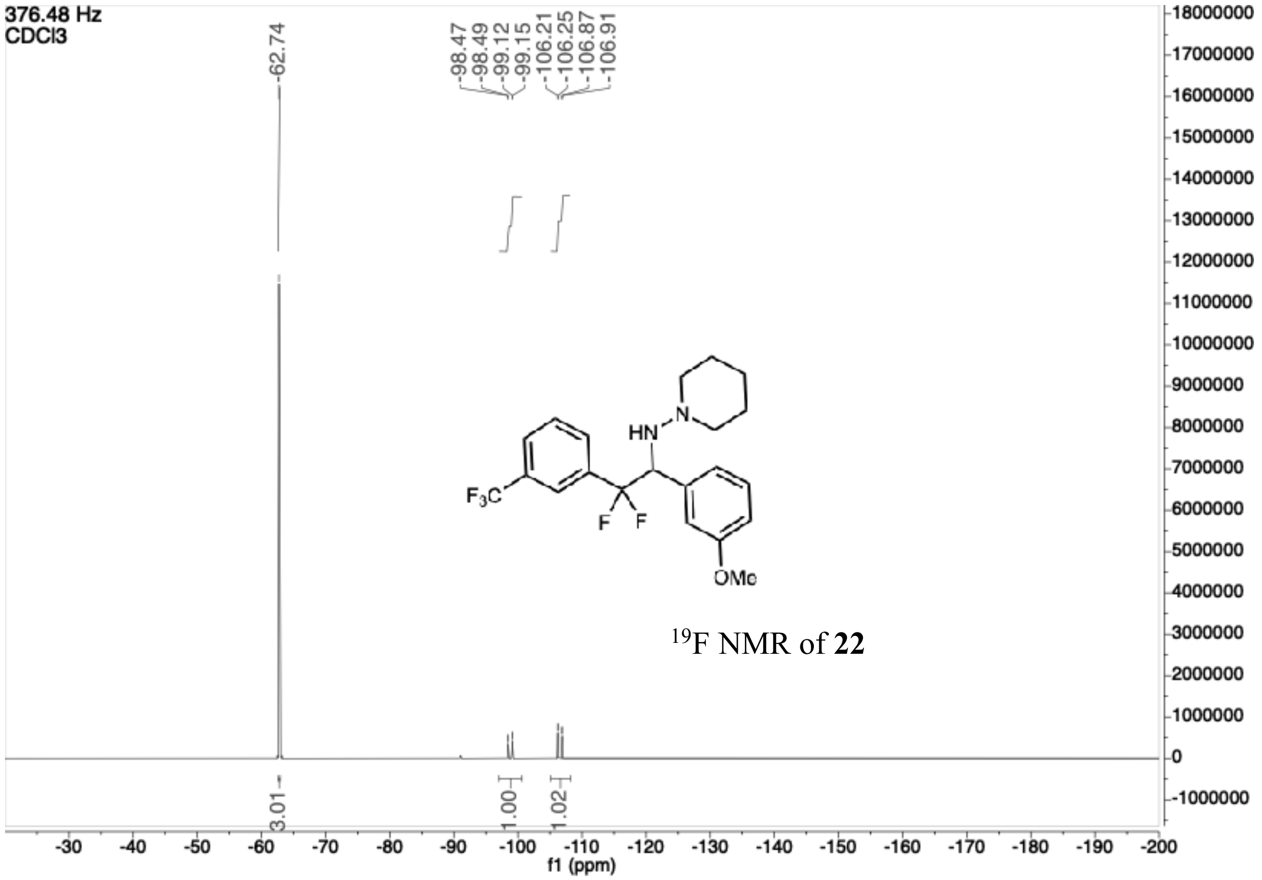
376.48 Hz
CDCl3
-62.74
-98.47
-98.49
-99.12
-99.15
-106.21
-106.25
-106.87
-106.91
3.01
1.00
1.02
f1 (ppm)
HN
N
F3C
F
F
OMe

^{19}F NMR of **22**

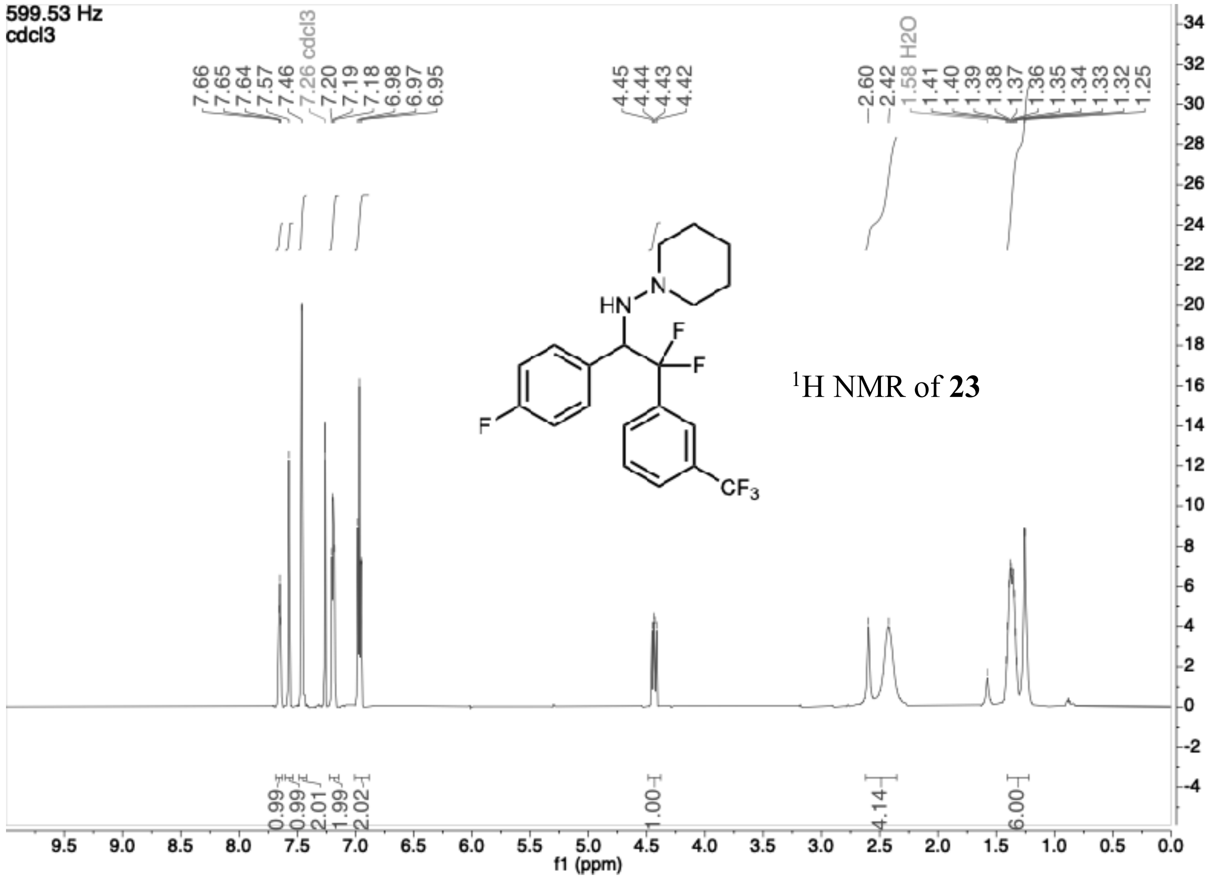

^{1}H NMR of **23**

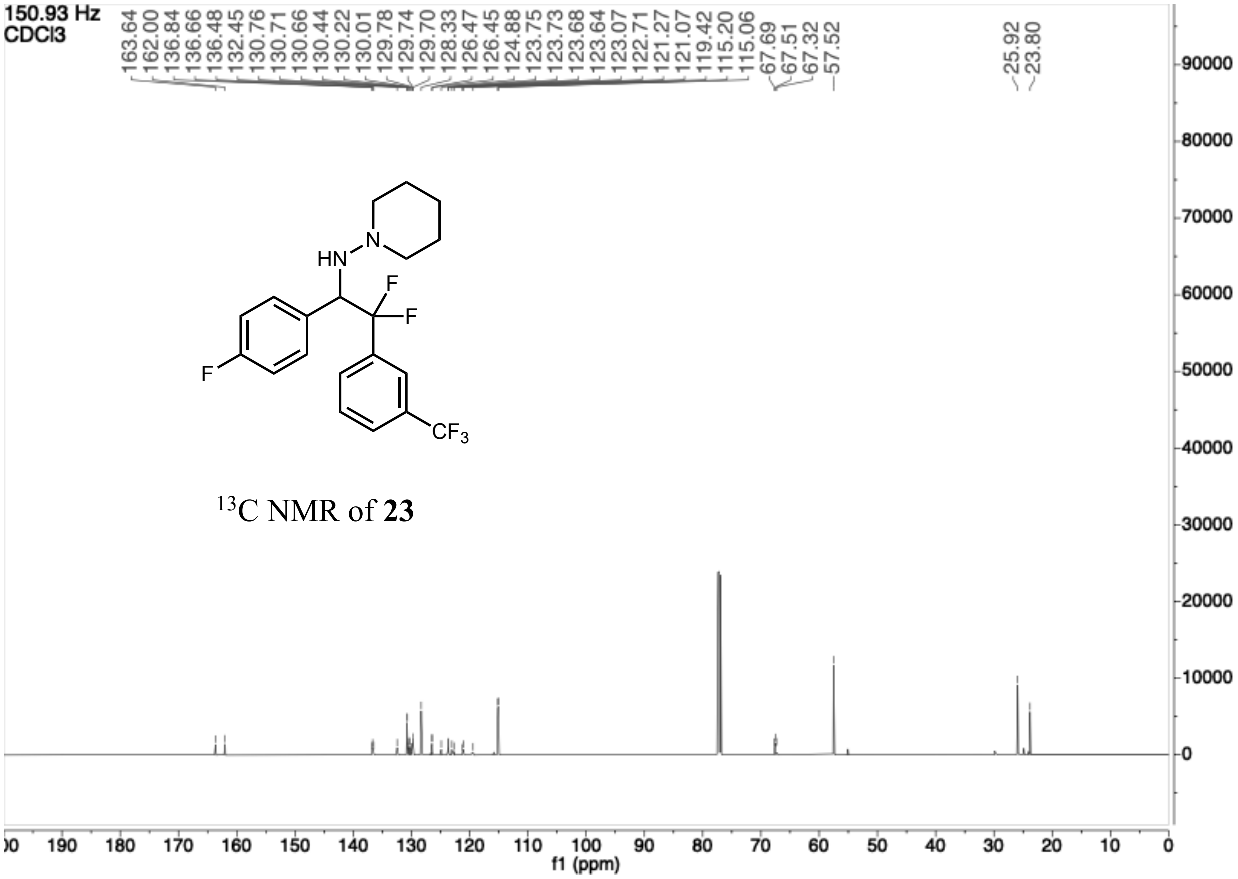

^{13}C NMR of **23**

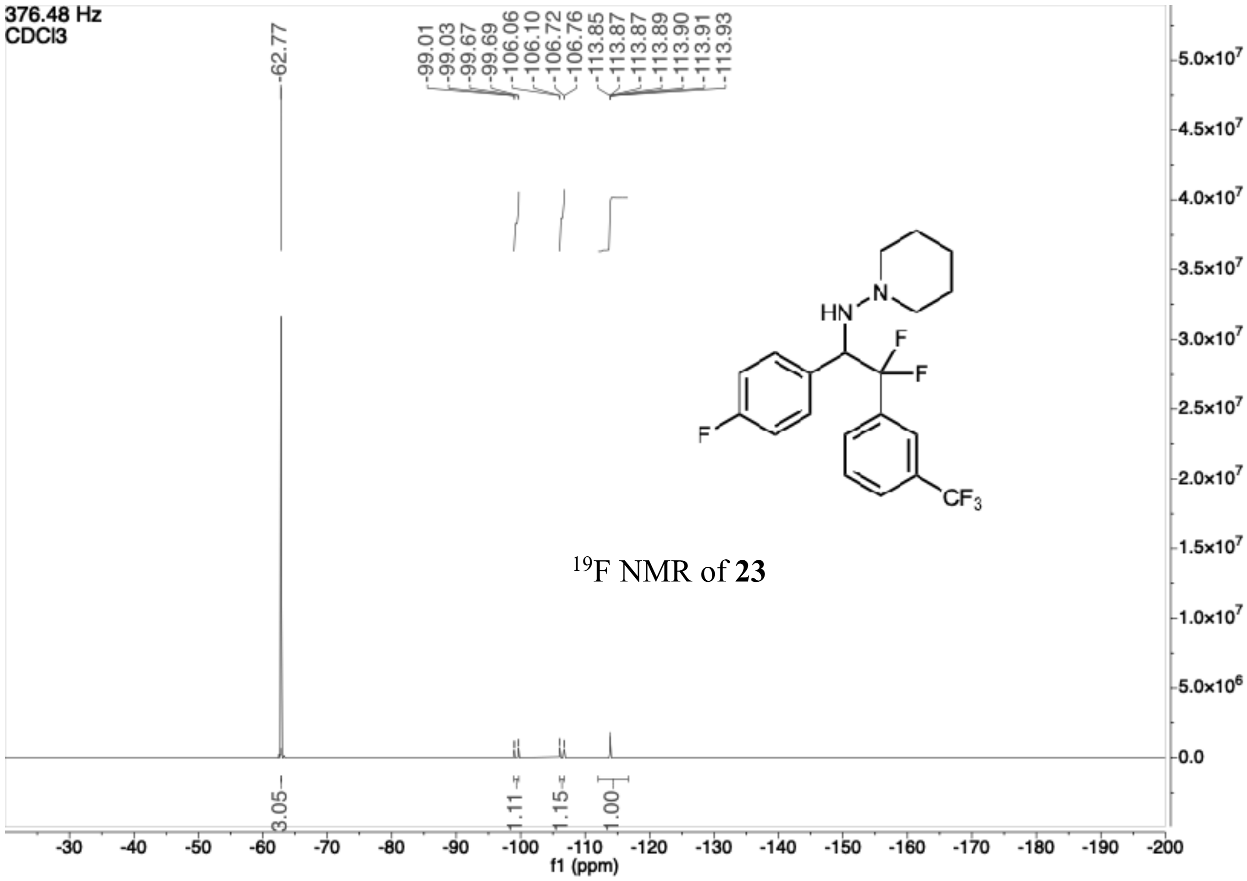

376.48 Hz
CDCl3
-62.77
-99.01
-99.03
-99.67
-99.69
-106.06
-106.10
-106.72
-106.76
-113.85
-113.87
-113.87
-113.89
-113.90
-113.91
-113.93
3.05
1.11
1.15
1.00
f1 (ppm)

^{19}F NMR of **23**

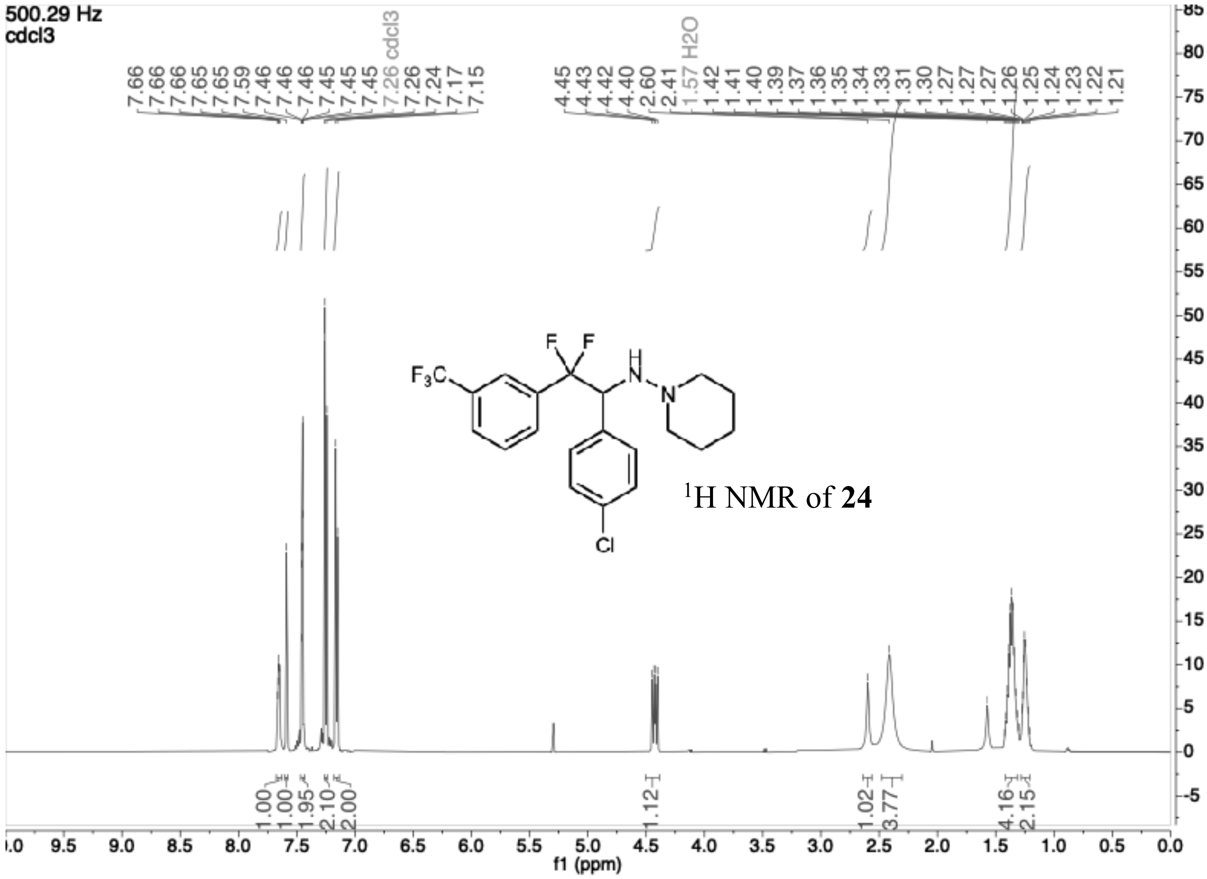

^{1}H NMR of **24**

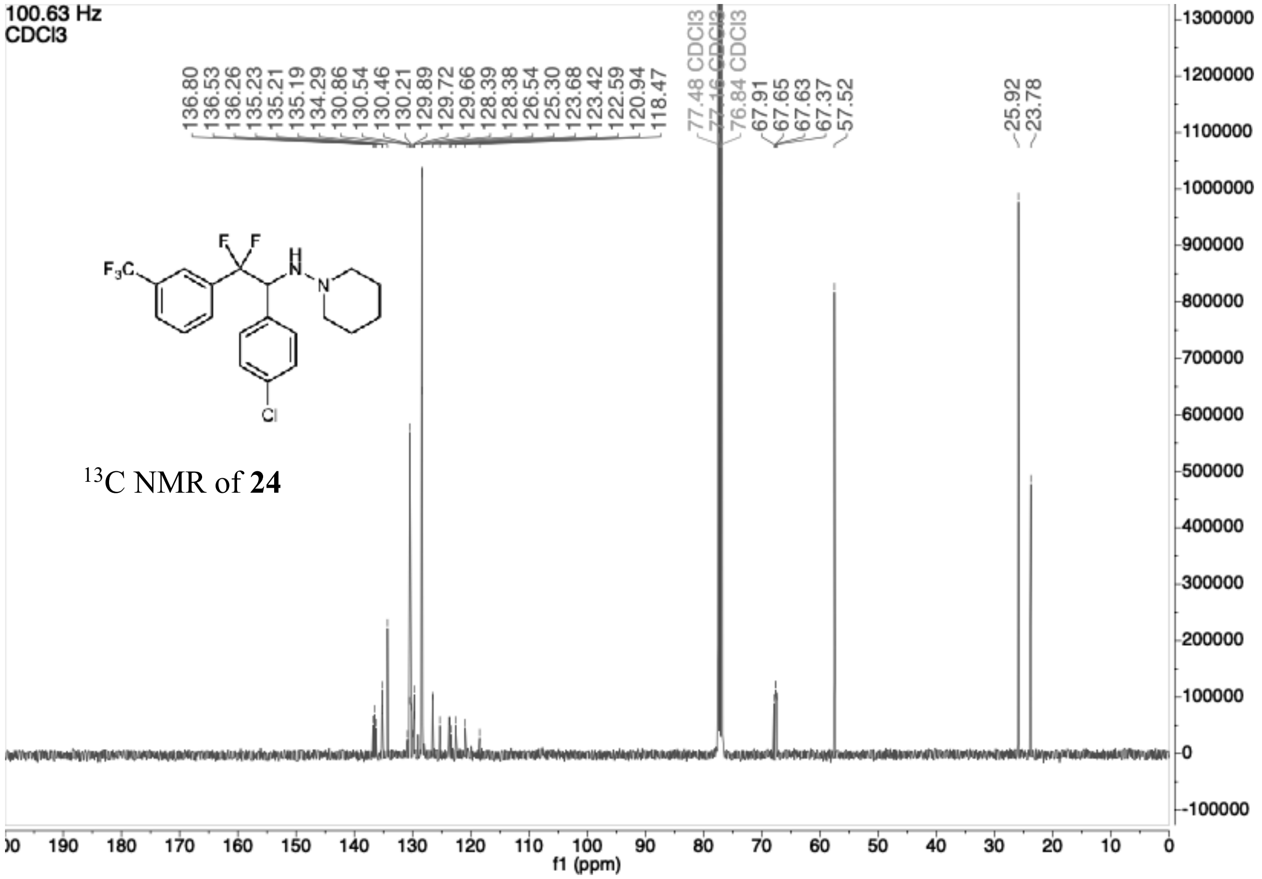

^{13}C NMR of **24**

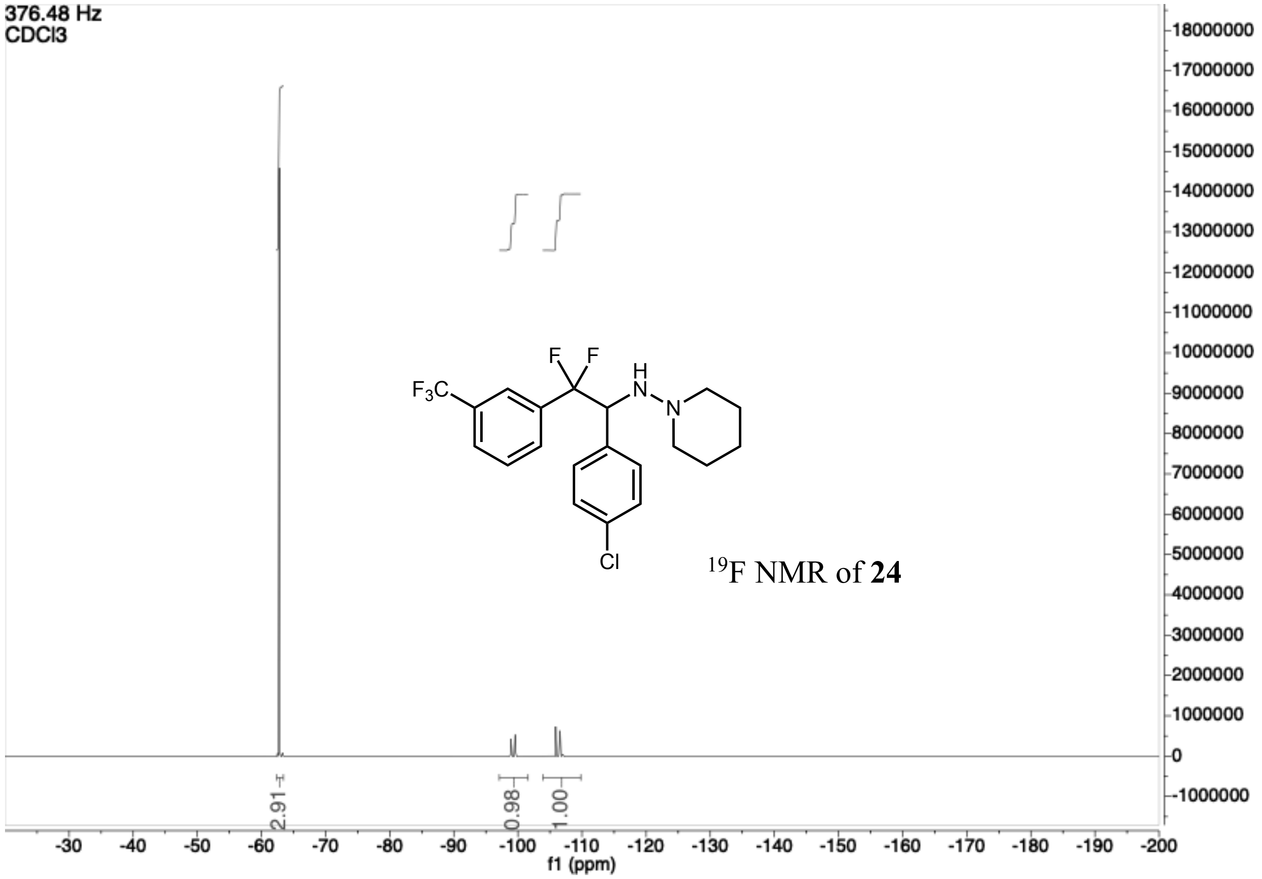

^{19}F NMR of **24**

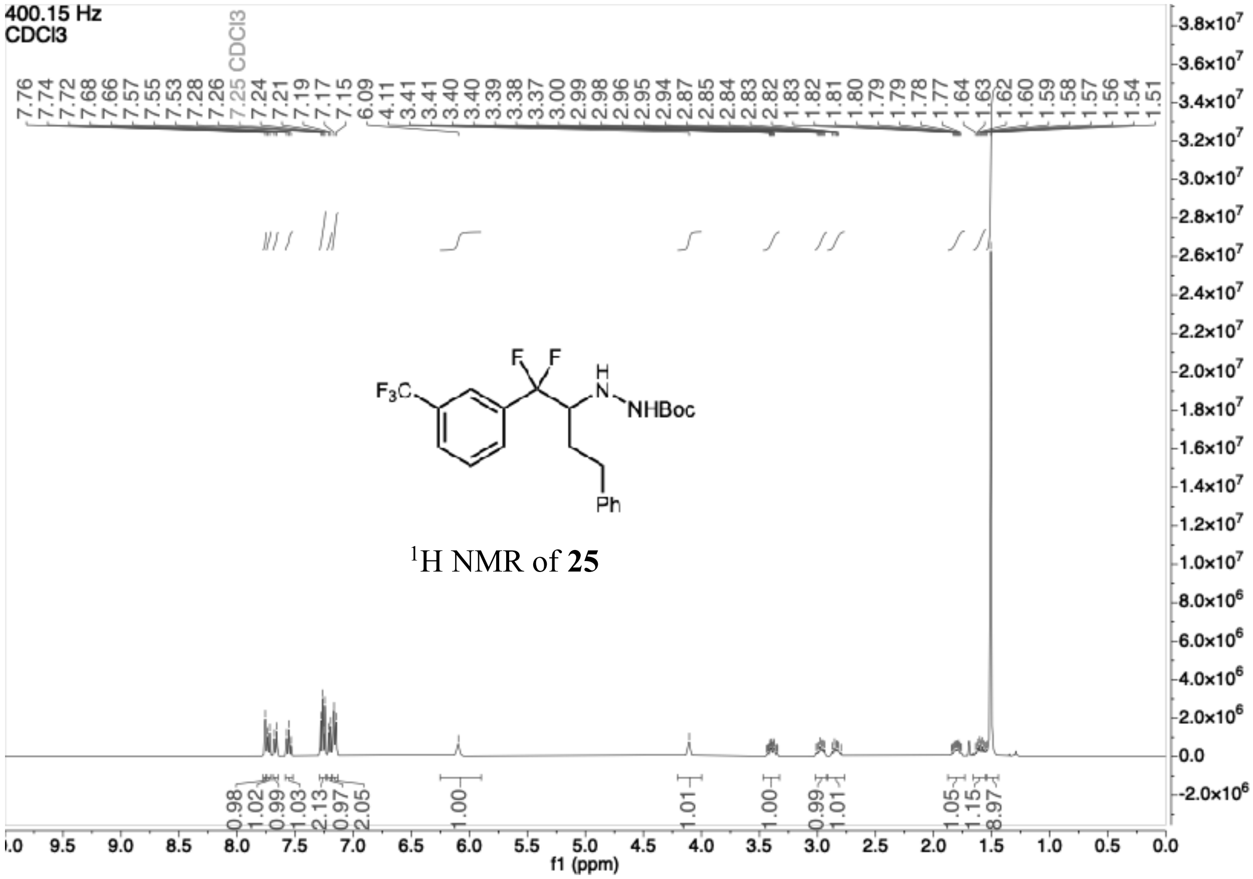

^{1}H NMR of **25**

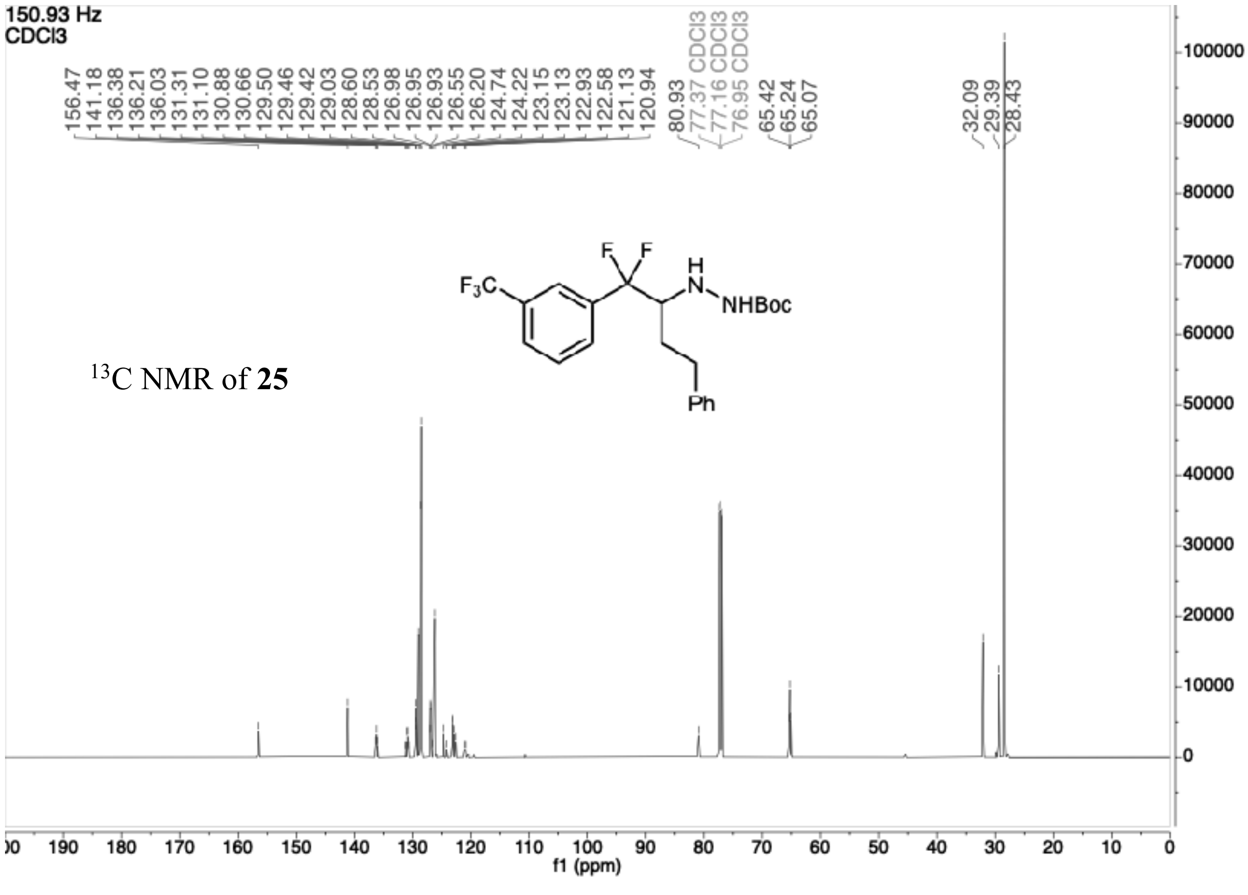

^{13}C NMR of **25**

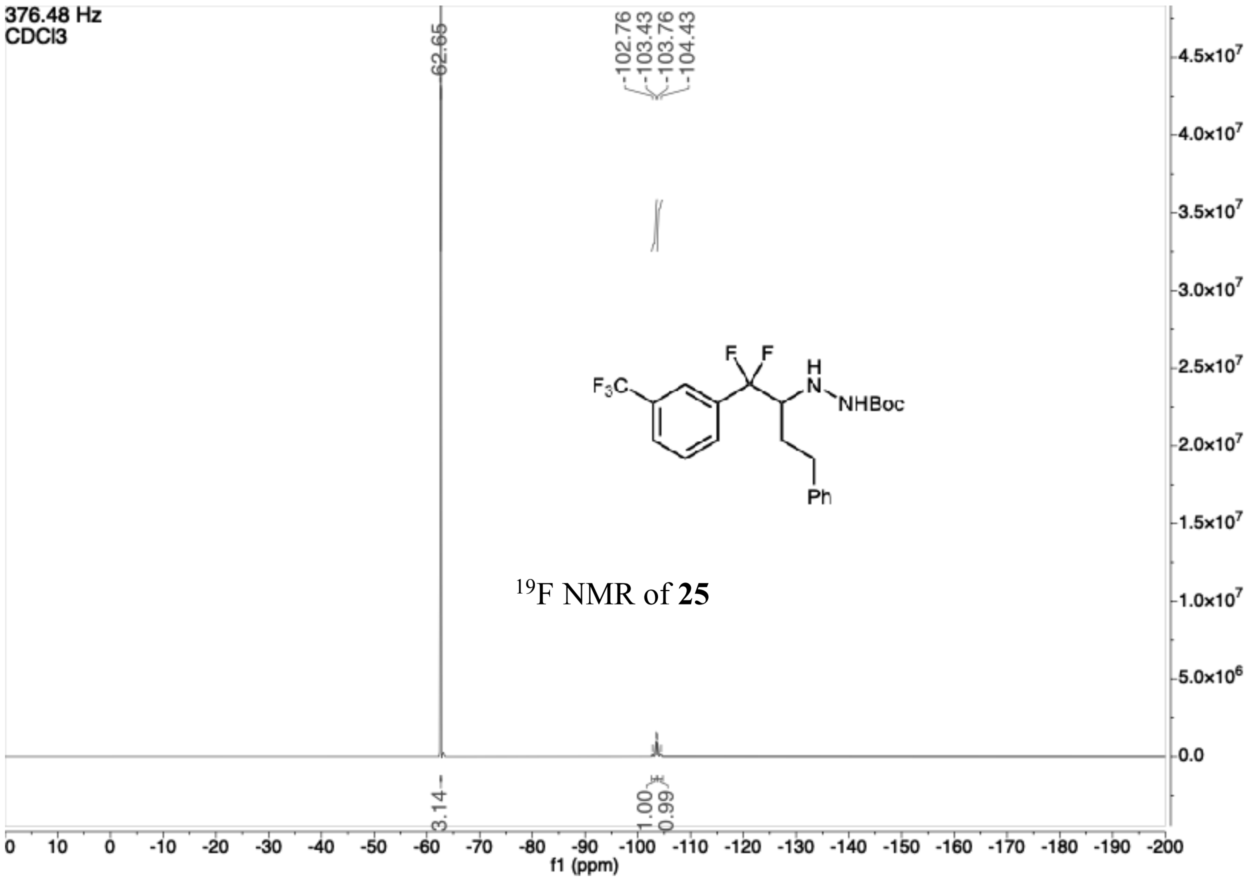
376.48 Hz
CDCl3
-62.65
-102.76
-103.43
-103.76
-104.43
F3C
F F
H
N
NHBoc
Ph
19F NMR of 25
3.14
1.00
0.99
f1 (ppm)

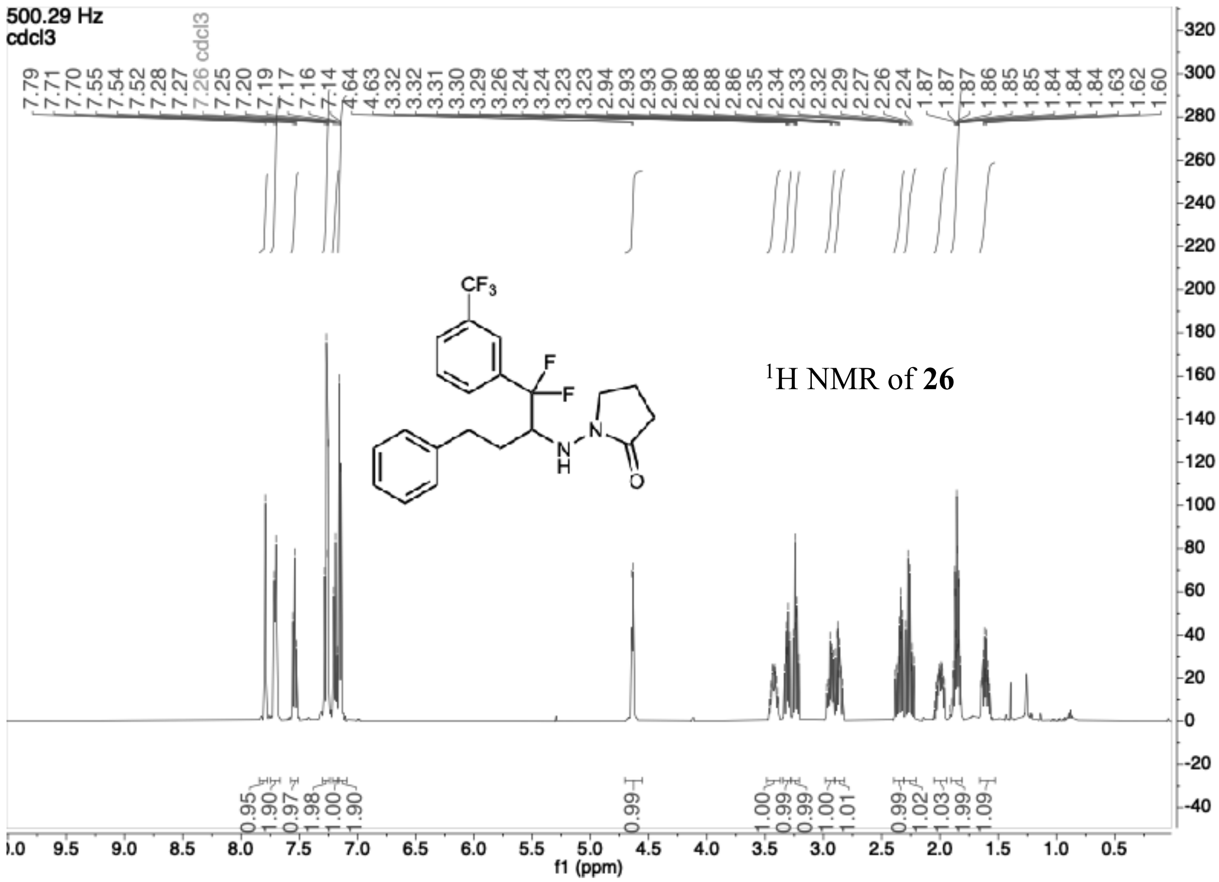
500.29 Hz
cdcl3
7.79 7.71 7.70 7.55 7.54 7.52 7.28 7.27 7.26 cdcl3 7.25 7.20 7.19 7.17 7.16 7.14 4.64 4.63 3.32 3.32 3.31 3.30 3.29 3.26 3.24 3.24 3.23 3.23 2.94 2.93 2.93 2.90 2.88 2.88 2.86 2.35 2.34 2.33 2.32 2.29 2.27 2.26 2.24 1.87 1.87 1.87 1.86 1.85 1.85 1.84 1.84 1.84 1.63 1.62 1.60
CF3
F
F
N
N
H
O
1H NMR of 26
0.95 1.90 0.97 1.98 1.00 1.90 0.99 1.00 0.99 0.99 1.00 1.01 0.99 1.02 1.03 1.99 1.09
f1 (ppm)

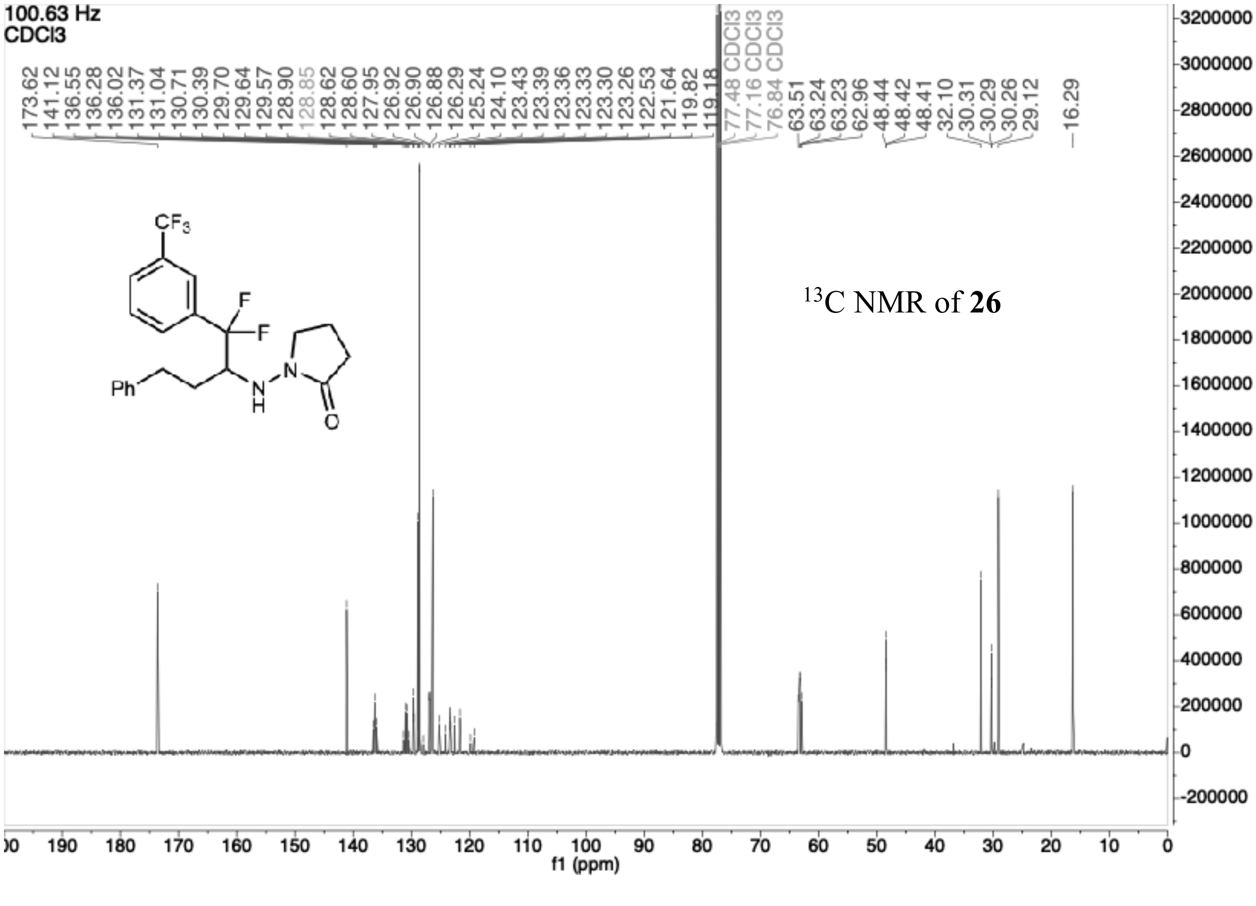
100.63 Hz
CDCl3
173.62 141.12 136.55 136.28 136.02 131.37 131.04 130.71 130.39 129.70 129.64 129.57 128.90 128.85 128.62 128.60 127.95 126.92 126.90 126.88 126.29 125.24 124.10 123.43 123.39 123.36 123.33 123.30 123.26 122.53 121.64 119.82 119.18 77.48 CDCl3 77.16 CDCl3 76.84 CDCl3 63.51 63.24 63.23 62.96 48.44 48.42 48.41 32.10 30.31 30.29 30.26 29.12 16.29
CF3
F
F
N
Ph
N
H
O
13C NMR of 26
f1 (ppm)

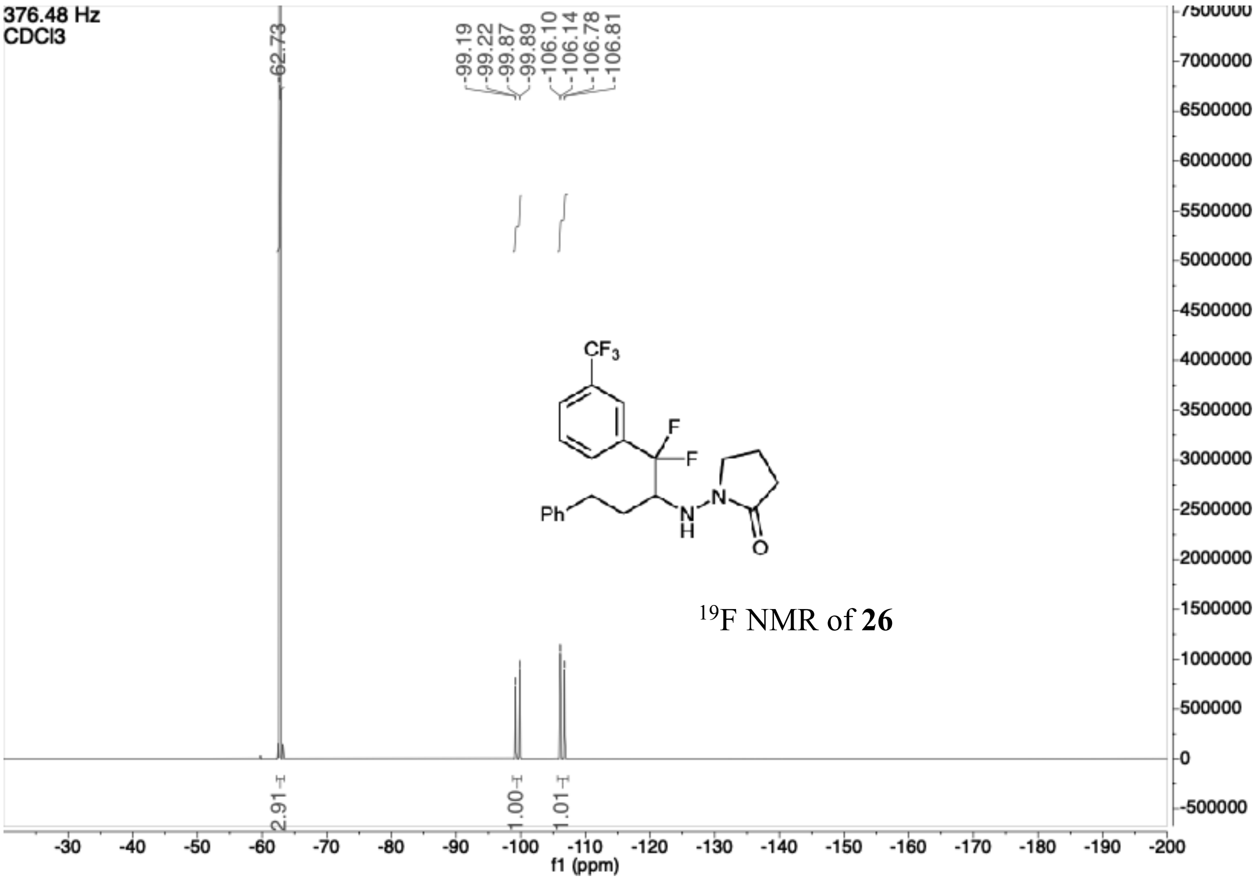
376.48 Hz
CDCl3
-62.73
-99.19
-99.22
-99.87
-99.89
-106.10
-106.14
-106.78
-106.81
CF3
F
F
Ph
N
H
N
O
2.91
1.00
1.01
f1 (ppm)

^{19}F NMR of **26**

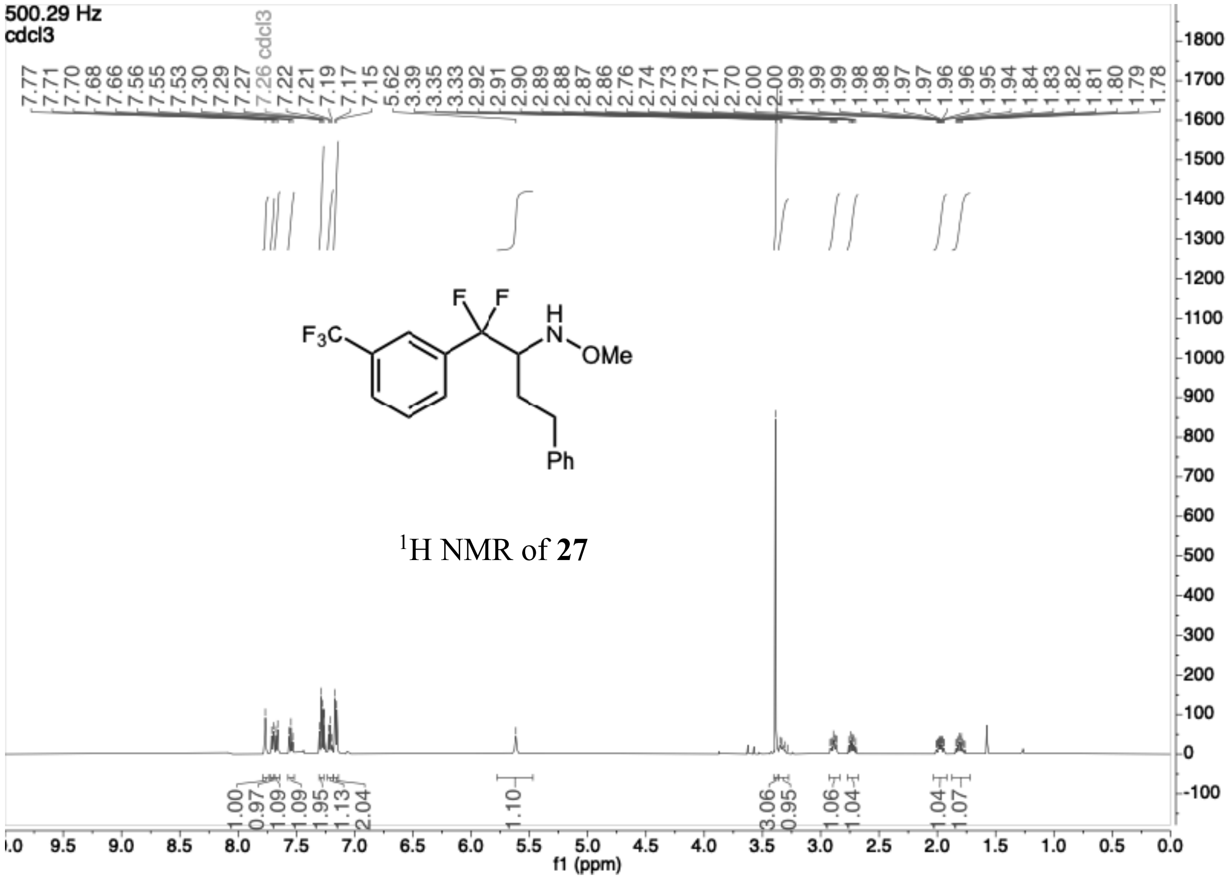

^{1}H NMR of **27**

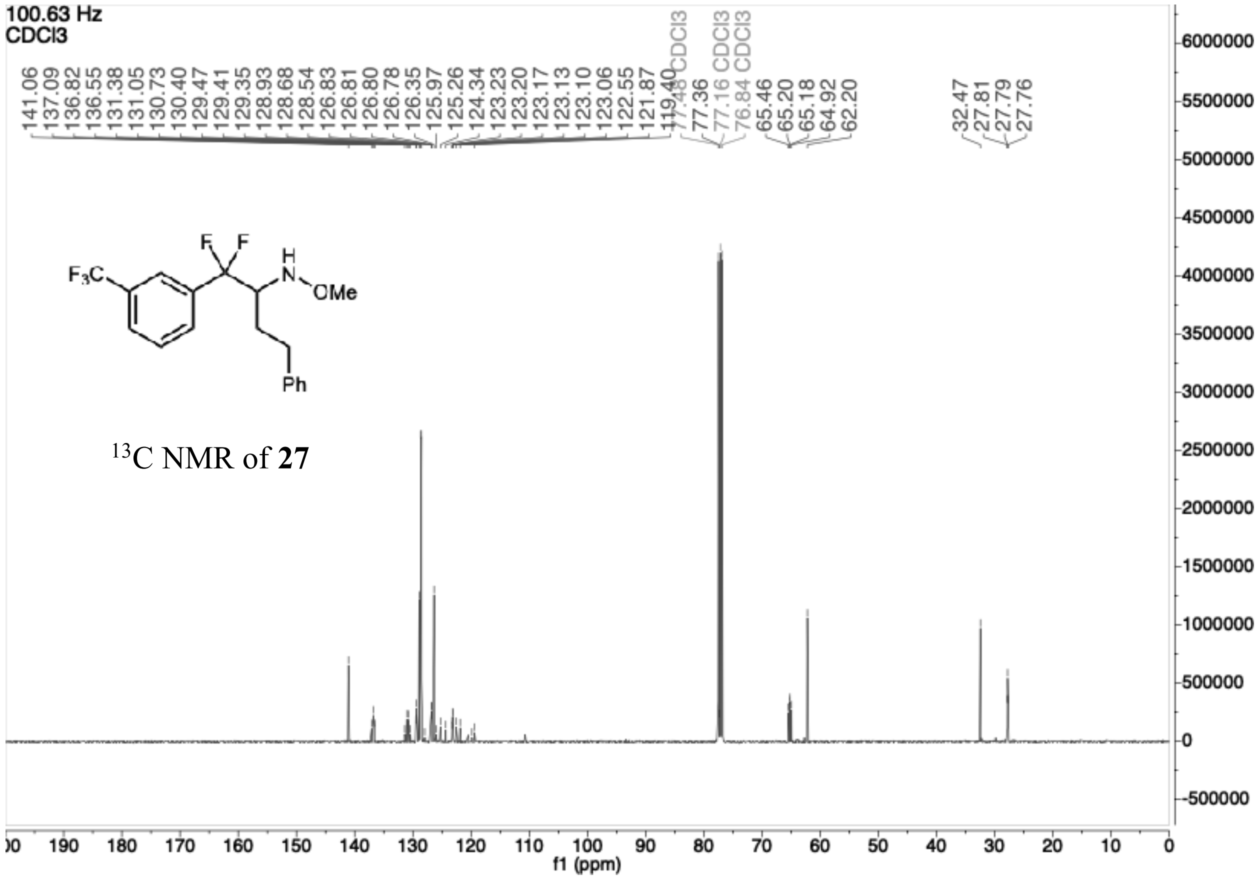

^{13}C NMR of **27**

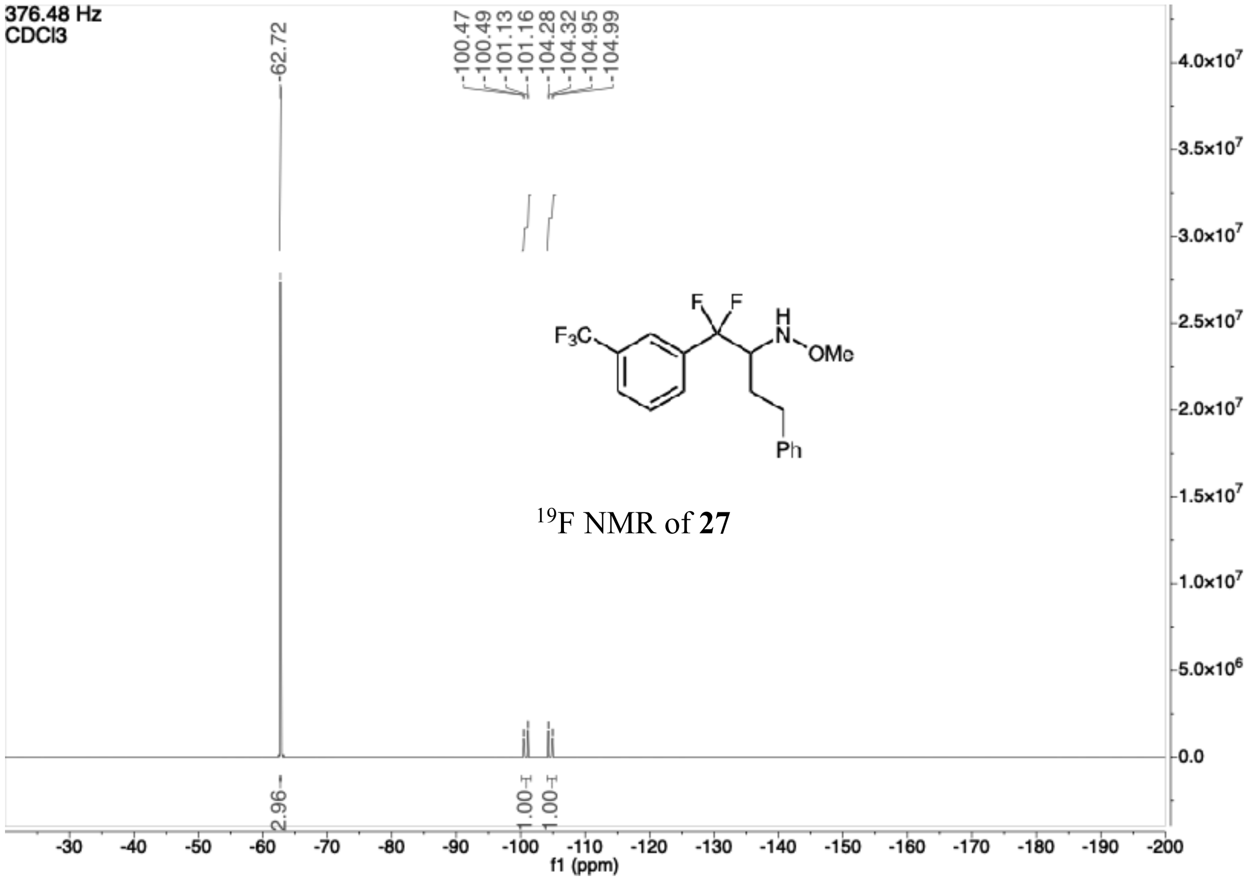
376.48 Hz
CDCl3
-62.72
-100.47
-100.49
-101.13
-101.16
-104.28
-104.32
-104.95
-104.99
F3C
F F
H
N
OMe
Ph
2.96
1.00
1.00
f1 (ppm)

^{19}F NMR of **27**

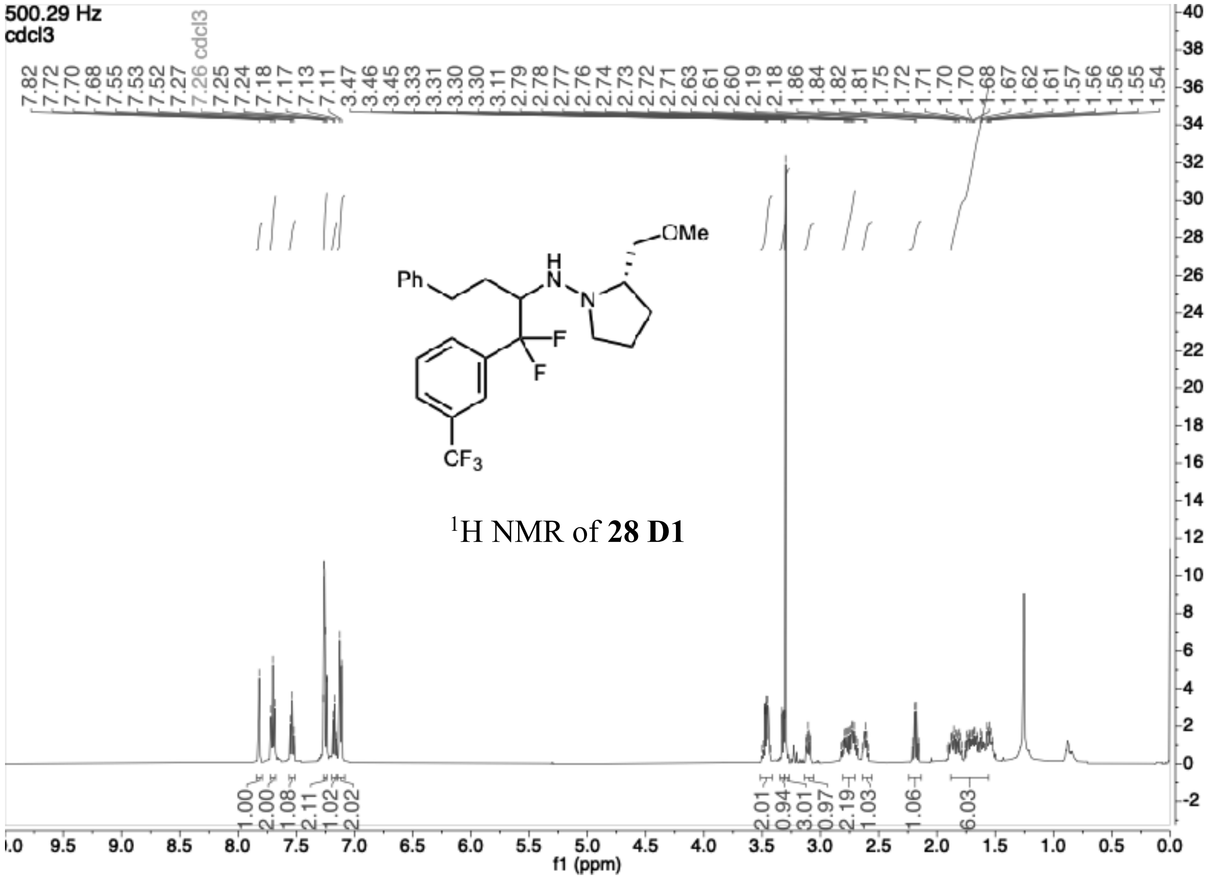
500.29 Hz
cdcl3
OMe
Ph
CF3
¹H NMR of **28 D1**
f1 (ppm)

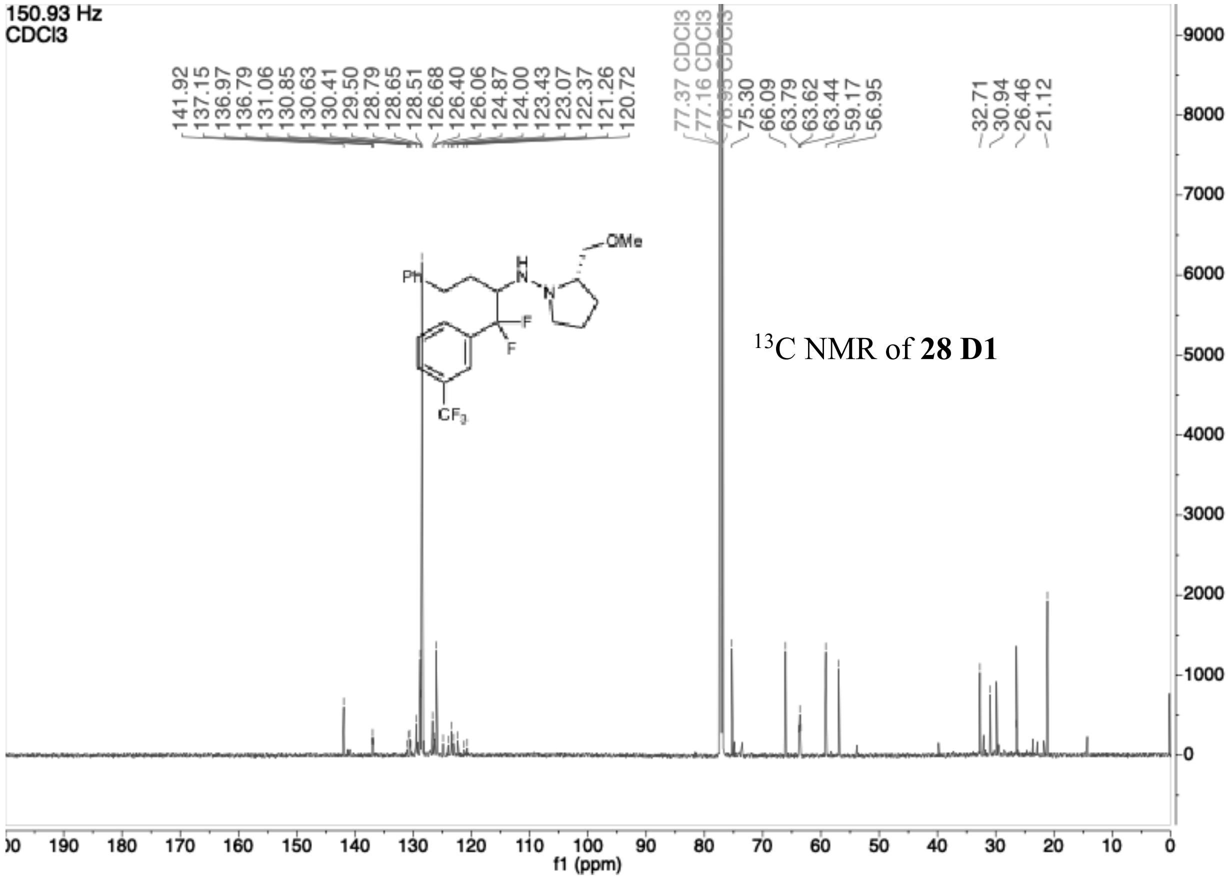
150.93 Hz
CDCl3
OMe
Ph
CF3
¹³C NMR of **28 D1**
f1 (ppm)

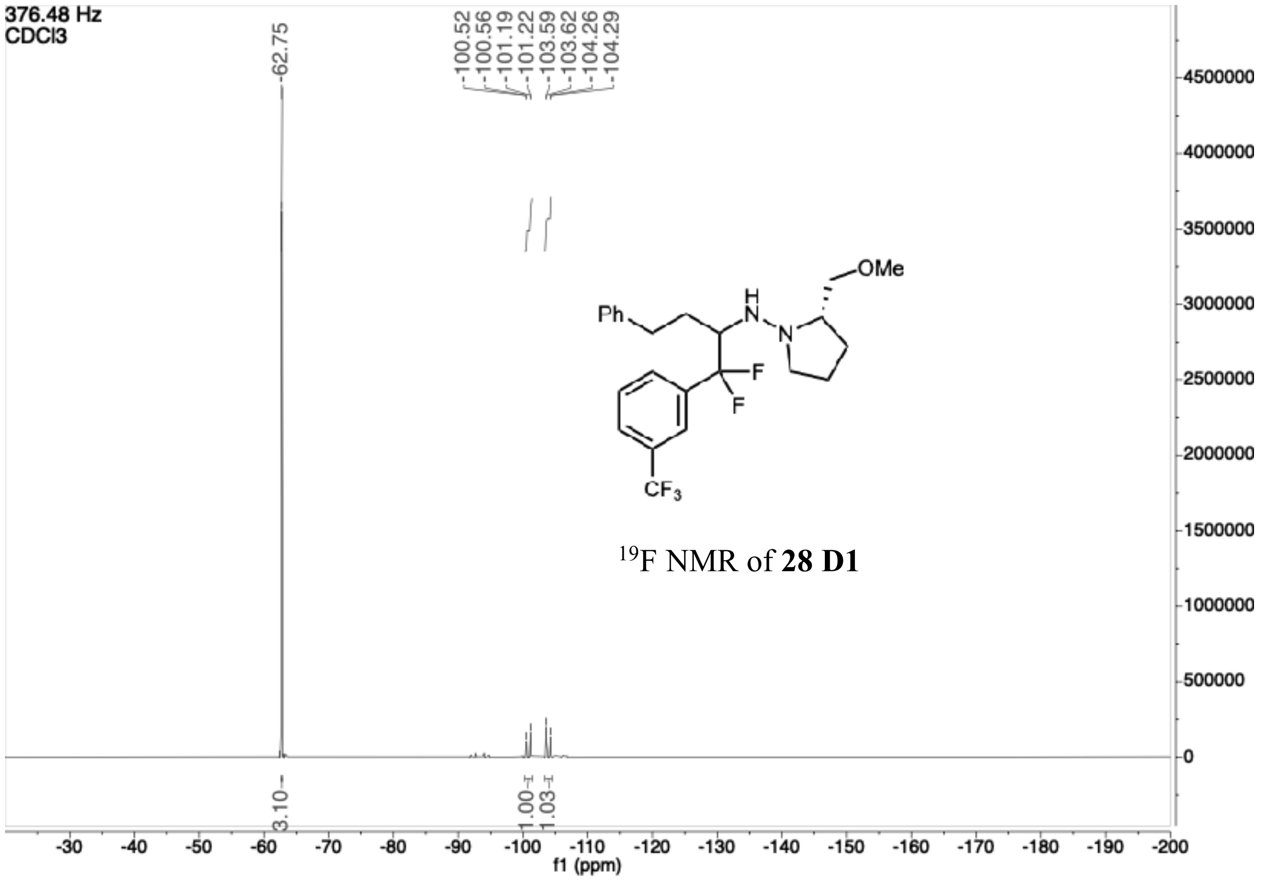

^{19}F NMR of **28 D1**

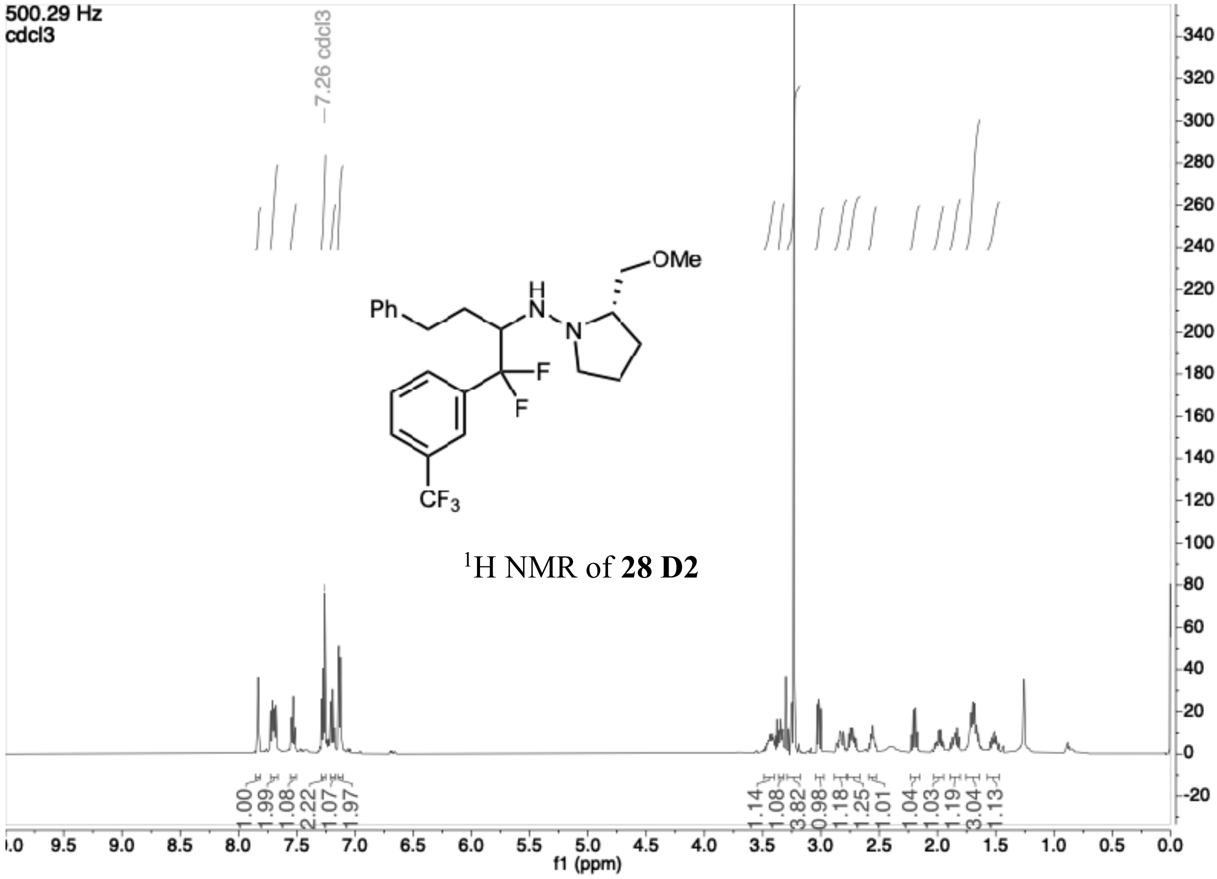

^1H NMR of **28 D2**

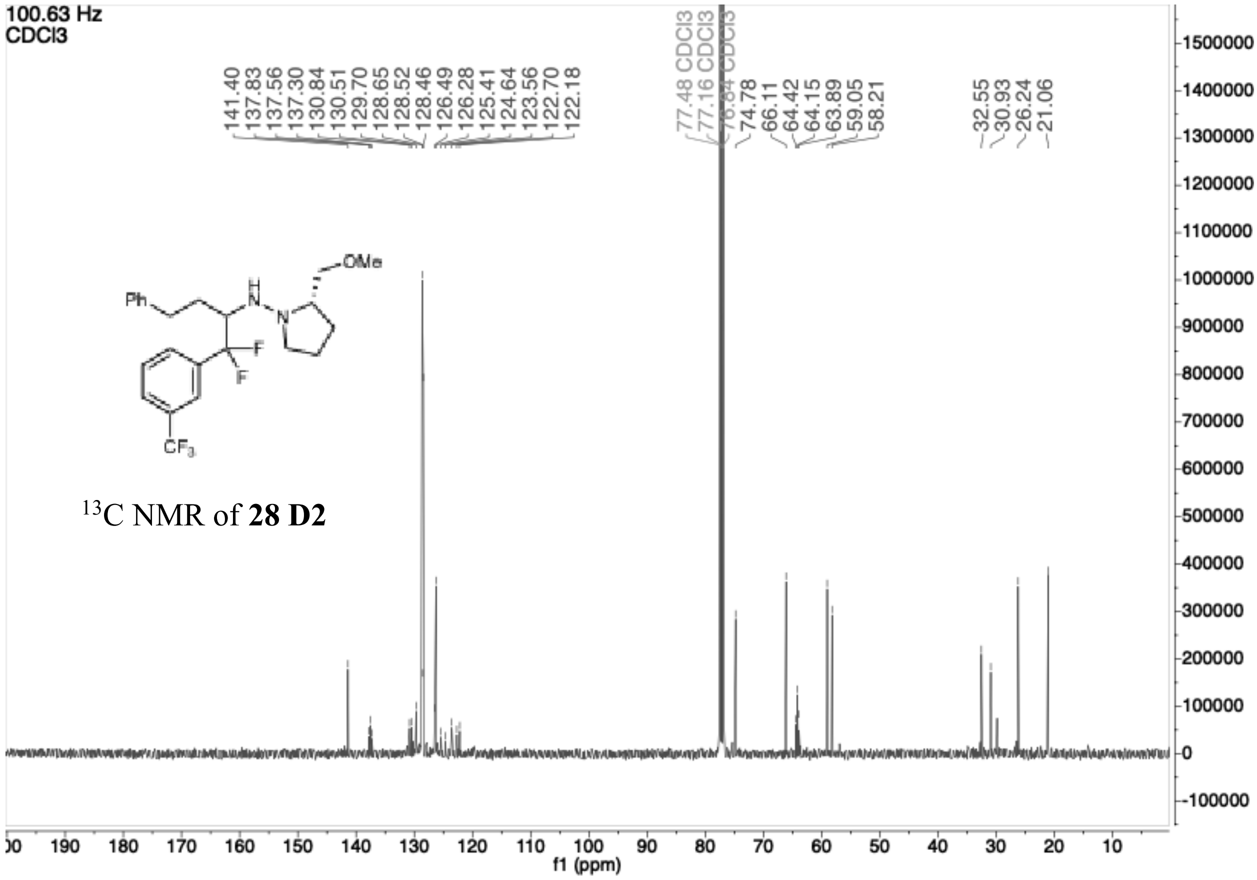

^{13}C NMR of **28 D2**

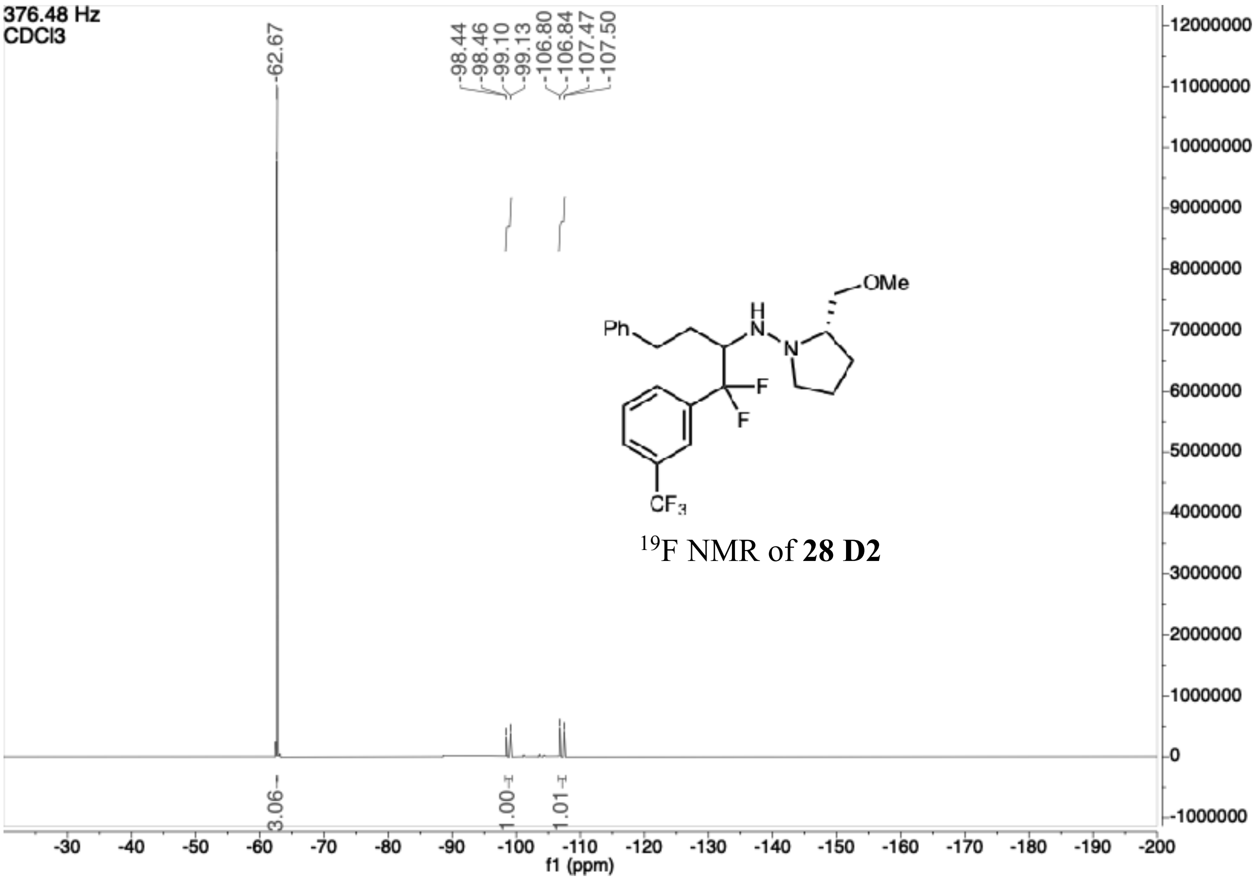

^{19}F NMR of **28 D2**

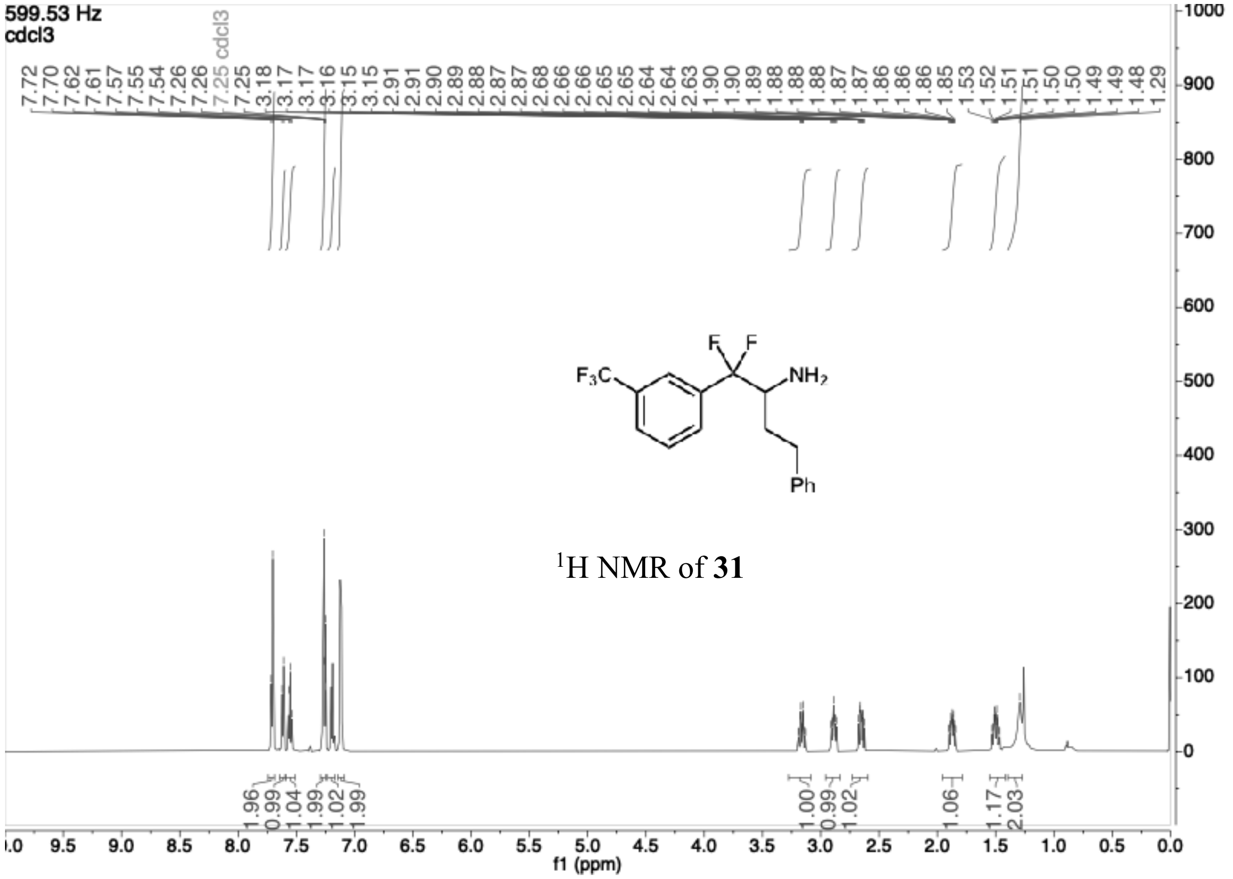
599.53 Hz
cdcl3
F F
F_3C
NH_2
Ph
^{1}H NMR of **31**
f1 (ppm)

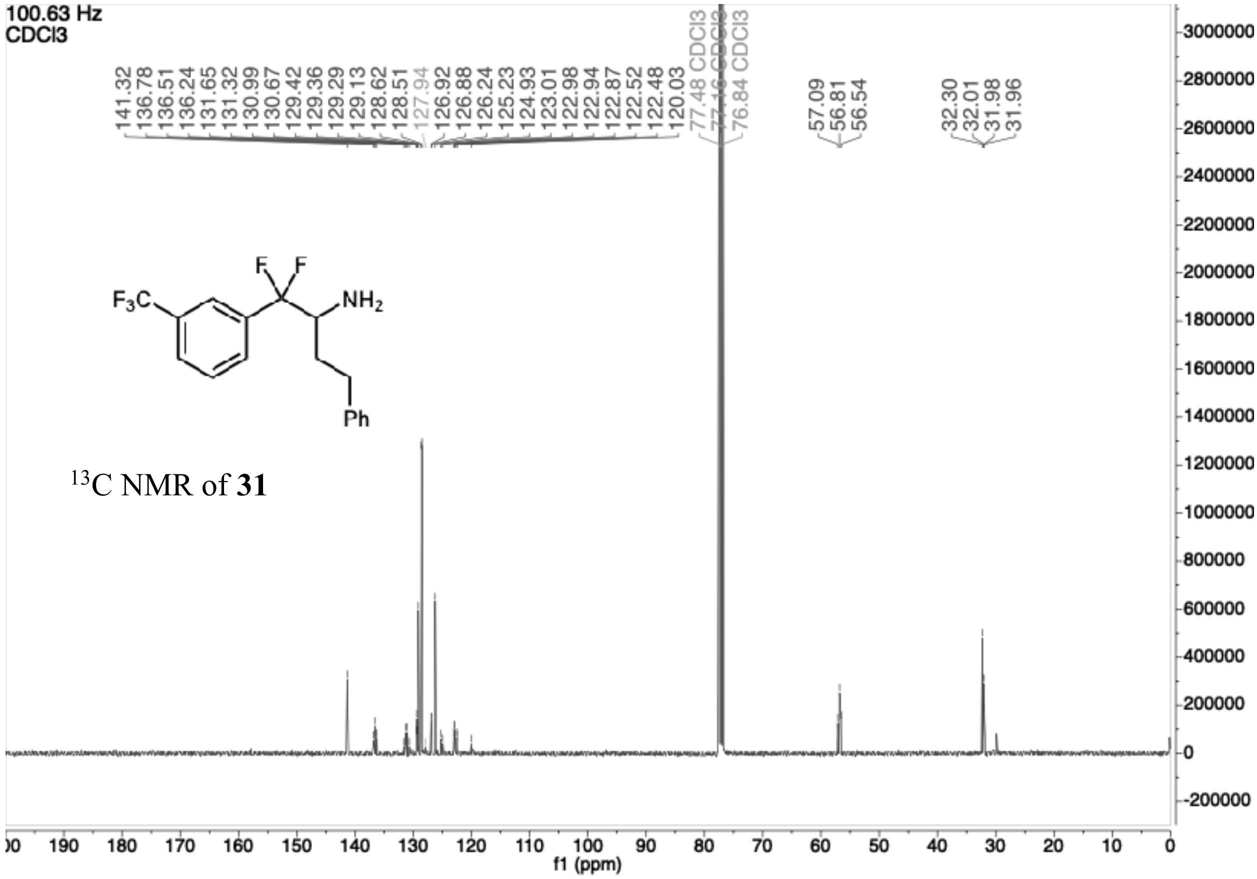
100.63 Hz
CDCl3
F F
F_3C
NH_2
Ph
^{13}C NMR of **31**
f1 (ppm)

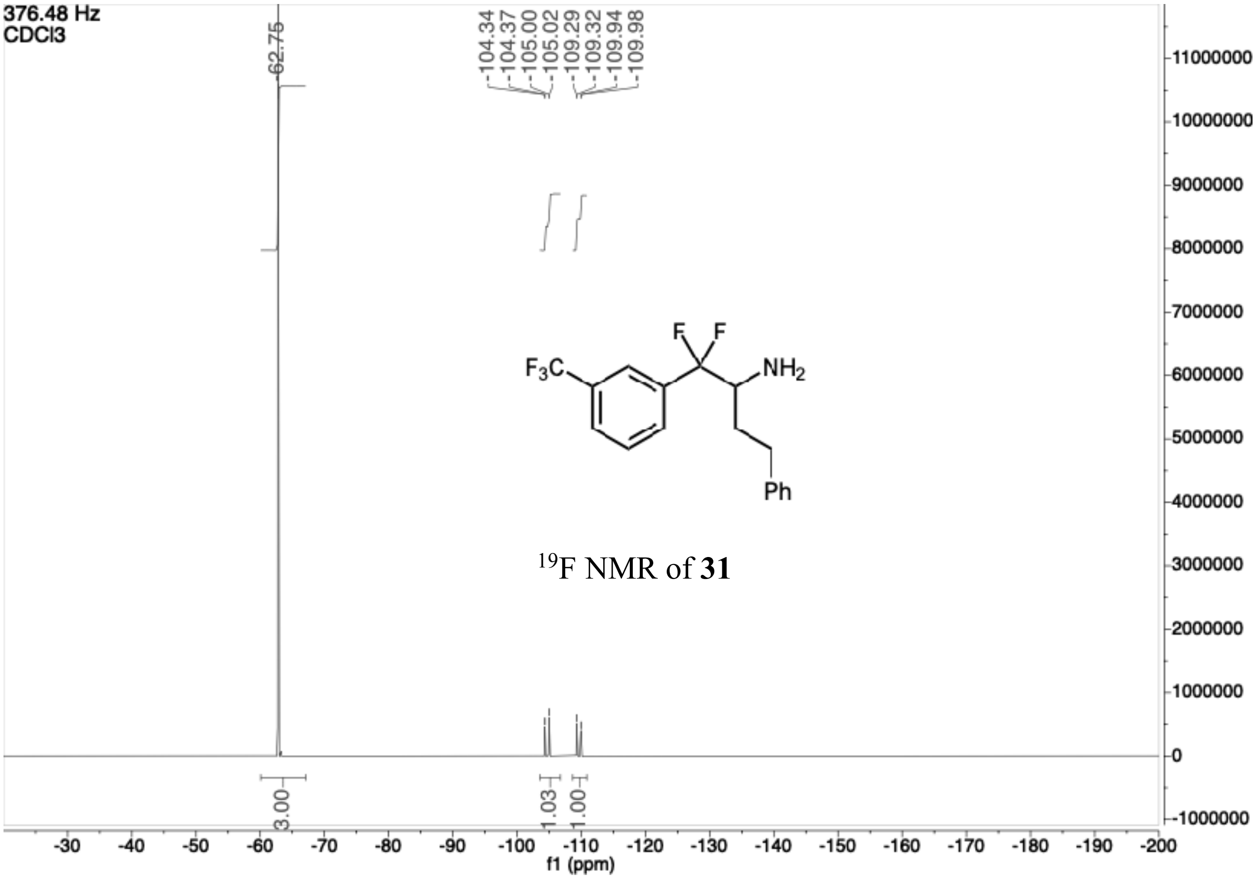
376.48 Hz
CDCl3
-62.75
-104.34
-104.37
-105.00
-105.02
-109.29
-109.32
-109.94
-109.98
F3C
F
F
NH2
Ph
3.00
1.03
1.00
f1 (ppm)

^{19}F NMR of **31**
